# Synthesis of highly substituted alkenes by sulfur-mediated olefination of *N*-tosylhydrazones

**DOI:** 10.1038/s42004-023-01058-2

**Published:** 2023-11-18

**Authors:** Peter Conen, Roman Nickisch, Michael A. R. Meier

**Affiliations:** 1 Institute of Organic Chemistry (IOC), Karlsruhe Institute of Technology (KIT), Fritz-Haber-Weg 6, 76131 Karlsruhe, Germany; 2 Institute of Biological and Chemical Systems – Functional Molecular Systems (IBCS-FMS), Karlsruhe Institute of Technology (KIT), Hermann-von-Helmholtz-Platz 1, 76344 Eggenstein-Leopoldshafen, Germany

**Keywords:** Synthetic chemistry methodology, Reaction mechanisms, Synthetic chemistry methodology, Organocatalysis, Sustainability

## Abstract

Tetraphenylethylenes (TPEs) are well-known for their aggregation-induced emission properties. The synthesis of TPE derivatives, as well as other highly substituted olefins, generally requires the use of hazardous reagents, such as metalorganic compounds, to overcome the high activation energies caused by the sterically congested double bond. Herein, we present an efficient and metal-free procedure for the synthesis of tetraarylethylenes *via* alkylidene-homocoupling of *N*-tosylhydrazones, derived from readily available benzophenones, in excellent yields. The method relies only on cheap and benign additives, *i.e*. elemental sulfur and potassium carbonate, and easily competes with other established procedures in terms of scope, yield and practicability. A mechanistic study revealed a diazo compound, a thioketone and a thiirane as key intermediates in the pathway of the reaction. Based on this, a modified method, which allows for selective alkylidene-cross-coupling, generating a broader scope of tri- and tetrasubstituted olefins in good yields, is showcased as well.

## Abstract (web summary)

Tetraphenylethylenes (TPEs) are known for their aggregation-induced emission and electrochemical properties of value for optical sensors, however, established synthetic routes suffer from several limitations. Here, the authors develop a metal-free route to TPEs and other alkenes via a sulfur-mediated coupling of *N*-tosylhydrazones, achieving good yields and a broad substrate scope.

## Introduction

Highly substituted carbon-carbon double bonds are ubiquitous structural motifs in a wide range of compounds used for instance in optical applications, as pharmaceuticals or in organic synthesis^1–7^. Tetraphenylethylenes (TPEs) are a subclass of highly substituted alkenes, which have attracted substantial interest in the past years due to their aggregation-induced emission (AIE) behavior and interesting electrochemical properties^3,8–10^. As such, TPEs have been used as templates for the design of numerous optical sensors for a large variety of different physical and chemical stimuli, and have shown promise in various optoelectronical applications such as organic light-emitting diodes (OLEDs)^11–16^.

The synthesis of tetrasubstituted alkenes (TSAs), such as TPEs, is met with challenges due to the substantial steric demand of the substitutents surrounding the double bond. The resulting high activation barriers for the formation of TSAs often render common methods for olefin synthesis, such as the Wittig and Horner-Wadsworth-Emmons olefinations, as well as olefin metathesis, highly ineffective^17,18^. Instead, TSA synthesis has to resort to harsh conditions and strong thermodynamic driving forces, which is often achieved by using metallic or metalorganic reagents.

**Fig. 1:**
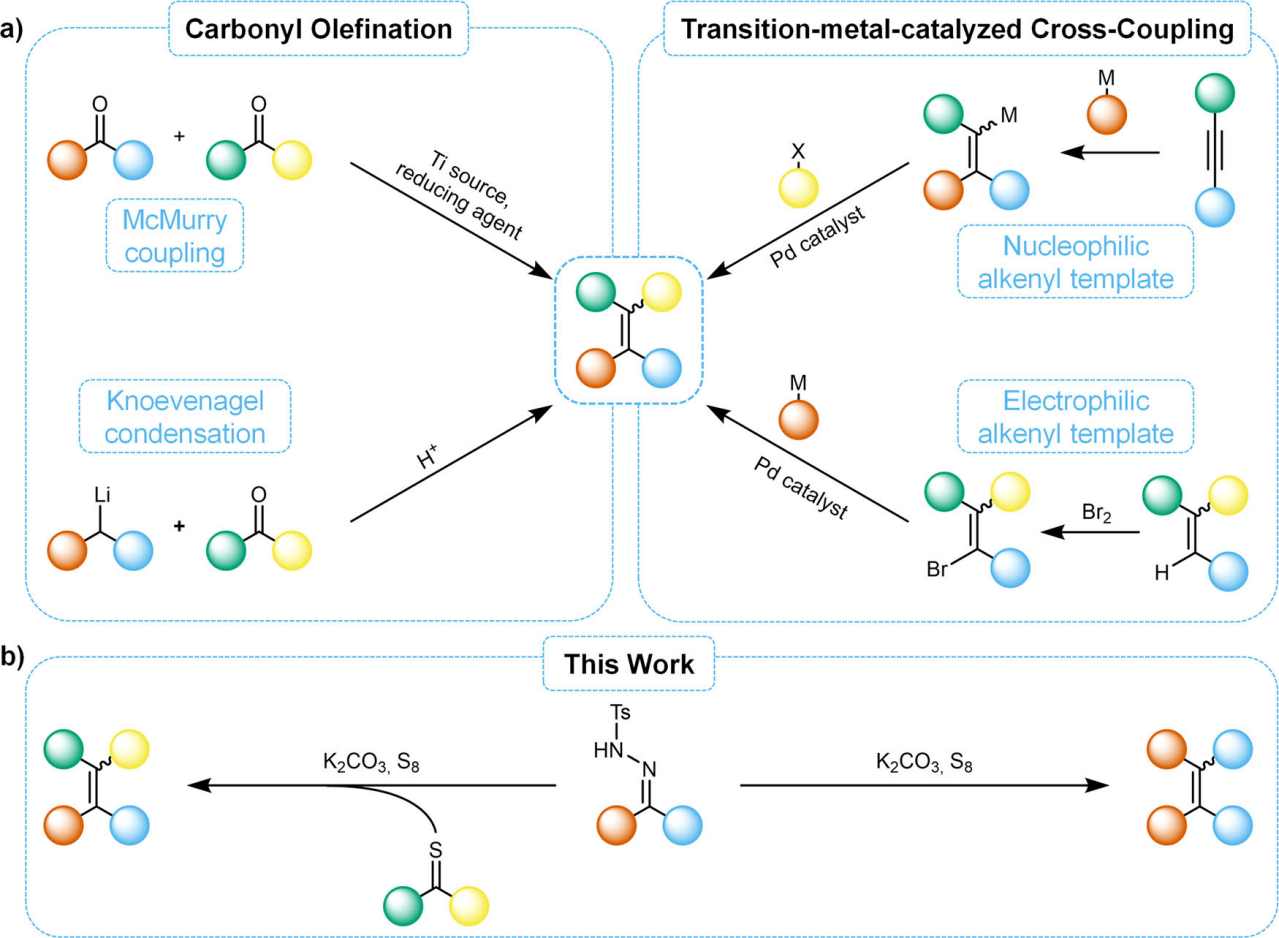
Synthetic routes towards tetraarylethylenes. **a** Schematic representation of previsouly described methods for the synthesis of TPEs based on carbonyl olefination reactions and transition-metal-catalyzed cross couplings. The colored spheres represent aryl moieties. **b** Sulfur-mediated alkylidene-homo- and cross-couplings of *N*-tosylhydrazones presented in this work.

The McMurry coupling of carbonyl compounds (Fig. 1a) offers a powerful strategy for the synthesis of sterically demanding alkenes and is therefore the most prevalent method for the construction of TPE derivatives^9,14,19–23^. However, the need for overstoichiometric amounts of a titanium source as well as often metallic reducing agents renders the procedure highly waste-intensive and moisture-sensitive. Further drawbacks are its incompatibility with reducible functional groups and its generally low selectivity for the cross-coupling of different alkylidene moieties^19^. Another carbonyl-based method is the Knoevenagel condensation (Fig. 1a) of benzophenones with lithiated diarylmethanes, which is superior to the McMurry reaction in synthesizing cross-coupled alkenes, but lacks general applicability due to the incompatibility of the organolithium reagents with numerous functional groups^24,25^.

Transition-metal-catalyzed cross-coupling reactions on existing alkene scaffolds are another effective route to access TPE derivatives and other TSAs (Fig. 1a)^6,7,26–31^. The alkenyl component can serve as either the electrophilic or the nucleophilic component in these reactions, which allows for great flexibility and potentially very high control over regio- and stereoselectivity of the synthesis. Despite these advantages, these methods suffer from the need for expensive catalysts and inert conditions as well as high excesses of the starting materials.

Due to the shortcomings of the above-mentioned synthetic protocols and the great utility of TPE derivatives, a cheap, efficient and easy to operate alternative procedure for their preparation is highly desirable. In this report, we present an effective methodology for the synthesis of tetraarylethylenes that uses *N*-tosylhydrazones (NTHs), which are easily accessible in high yields from the respective carbonyl compounds, as the substrates for a sulfur-mediated olefination under air atmosphere. Herein, the TPEs are formed *via* homocoupling of the NTH alkylidene moities. Furthermore, after understanding the underlying reaction mechanism, a variation of the method using an additional thioketone to enable a selective synthesis of alkylidene-cross-coupled TPEs and TSAs is showcased (Fig. 1b). The presented procedures can easily compete with the already established and above-described methods for the synthesis of TPEs in terms of yield, scope and practicability.

## Results

### Reaction optimization

NTH **1a** was initially reacted with 1.2 equivalents of sodium hydride along with 0.28 equivalents of elemental sulfur (corresponds to 2.24 atomic equivalents of sulfur) in DMSO-*d*_*6*_ at 130 °C (Table 1, entry 1). The reaction conditions were chosen anticipating the in situ formation of a carbene from the NTH in a similar fashion to Bamford-Stevens reactions, as well as the activation of elemental sulfur under basic conditions in aprotic polar solvents (*vide infra)*. Regarding the known reactivity of activated elemental sulfur with carbenes to form thiocarbonyl moieties, the formation of a thioketone was expected. The progress of the reaction was followed *via* ^1^H-NMR spectroscopy. After 1 h, the two methoxy signals of **1a** had given way to a single new signal in the methoxy region of the crude ^1^H-NMR spectrum, indicating the selective formation of one product, which was later identified as TPE **2a** instead of the expected thioketone. **2a** was isolated by direct column chromatography of the crude reaction mixture in an excellent yield of 92%. The selective formation of a TPE served as motivation for further investigations.

**Table 1:** Optimization of the reaction conditions for the homocoupling of 1a to 2a.


Entry	Base (eq.)	S_8_ / (eq.)	Solvent (conc.)	T / °C	t / min	Conversion of 1a / %
1	NaH (1.20)	0.28	DMSO-d_6_ (0.5 M)	130	60	100
2	NaH (1.20)	0.28	DMSO-d_6_ (0.5 M)	**80**	**300**	66
3	NaH (1.20)	0.28	DMSO-d_6_ (0.5 M)	**90**	**300**	86
4	NaH (1.20)	0.28	DMSO-d_6_ (0.5 M)	**100**	**30**	82
5	**DBU (1.20)**	0.28	DMSO-d_6_ (0.5 M)	100	30	66
6	**K**_**2**_**CO**_**3**_ **(1.20)**	0.28	DMSO-d_6_ (0.5 M)	100	**20**	100
7	**Cs**_**2**_**CO**_**3**_ **(1.20)**	0.28	DMSO-d_6_ (0.5 M)	100	20	100
8	K_2_CO_3_ (1.20)	0.28	**GBL (0.5** **M)**	100	20	n.d. ^a)^
9	K_2_CO_3_ (1.20)	0,28	**DMF (0.5** **M)**	100	20	76
10	K_2_CO_3_ (1.20)	0.28	**NMP (0.5** **M)**	100	20	88
11	K_2_CO_3_ (1.20)	0.28	**DMSO-d**_**6**_ **(1** **M)**	100	20	100
12	K_2_CO_3_ (1.20)	0.28	**DMSO-d**_**6**_ **(0.25** **M)**	100	20	100
13	K_2_CO_3_ (1.20)	**0.1875**	DMSO-d_6_ (0.5 M)	100	20	100 ^a)^
14	K_2_CO_3_ (1.20)	**0.1375**	DMSO-d_6_ (0.5 M)	100	20	100 ^a)^
**15**	**K**_**2**_**CO**_**3**_ **(1.20)**	**0.25**	**DMSO (0.5** **M)**	**100**	30	**100**

a) Significant formation of side products was observed.

The respective altered conditions are highlighted in bold.

Optimization of the reaction conditions was conducted *via* ^1^H-NMR monitoring (Table 1). Lowering the reaction temperature to below 100 °C was found to significantly slow down the reaction, yielding incomplete conversion even after 5 h (Table 1, entries 2 and 3). At 100 °C, 82% conversion was observed already after 30 min (Table 1, entry 4). Potassium carbonate and cesium carbonate were found to be superior bases to NaH, yielding full conversion of **1a** at 100 °C after already 20 min, while 1,8-diaza[5.4.0]bicycloundec-7-ene (DBU) showed lower efficacy (Table 1, entries 5-7). Potassium carbonate was thus selected as the optimal base due to its lower price in comparison to cesium carbonate.

Different polar-aprotic solvents, namely *N,N*-dimethylformamide (DMF), *γ-*butyrolactone (GBL) and *N*-methyl-2-pyrrolidone (NMP), all proved to be less viable than DMSO due to lower conversion of **1a** and the formation of side products (Table 1, entries 8-10). Altering the concentration of the reaction mixture as well as decreasing the amount of elemental sulfur was found to have no impact on the full conversion of **1a** after 20 min (Table 1, entries 11-14). However, a significant increase in side product formation was observed in the reactions with lower sulfur equivalents (Table 1, entries 13-14). Furthermore, carrying out the reaction in non-deuterated DMSO was found to also yield full conversion after 30 min using 0.25 equivalents of sulfur and was thus determined to be the optimized reaction conditions, leading to an isolated yield of 99% of TPE **2a** in a fast and simple one step procedure from NTH **1a**.

### Homocoupling scope

**Fig. 2:**
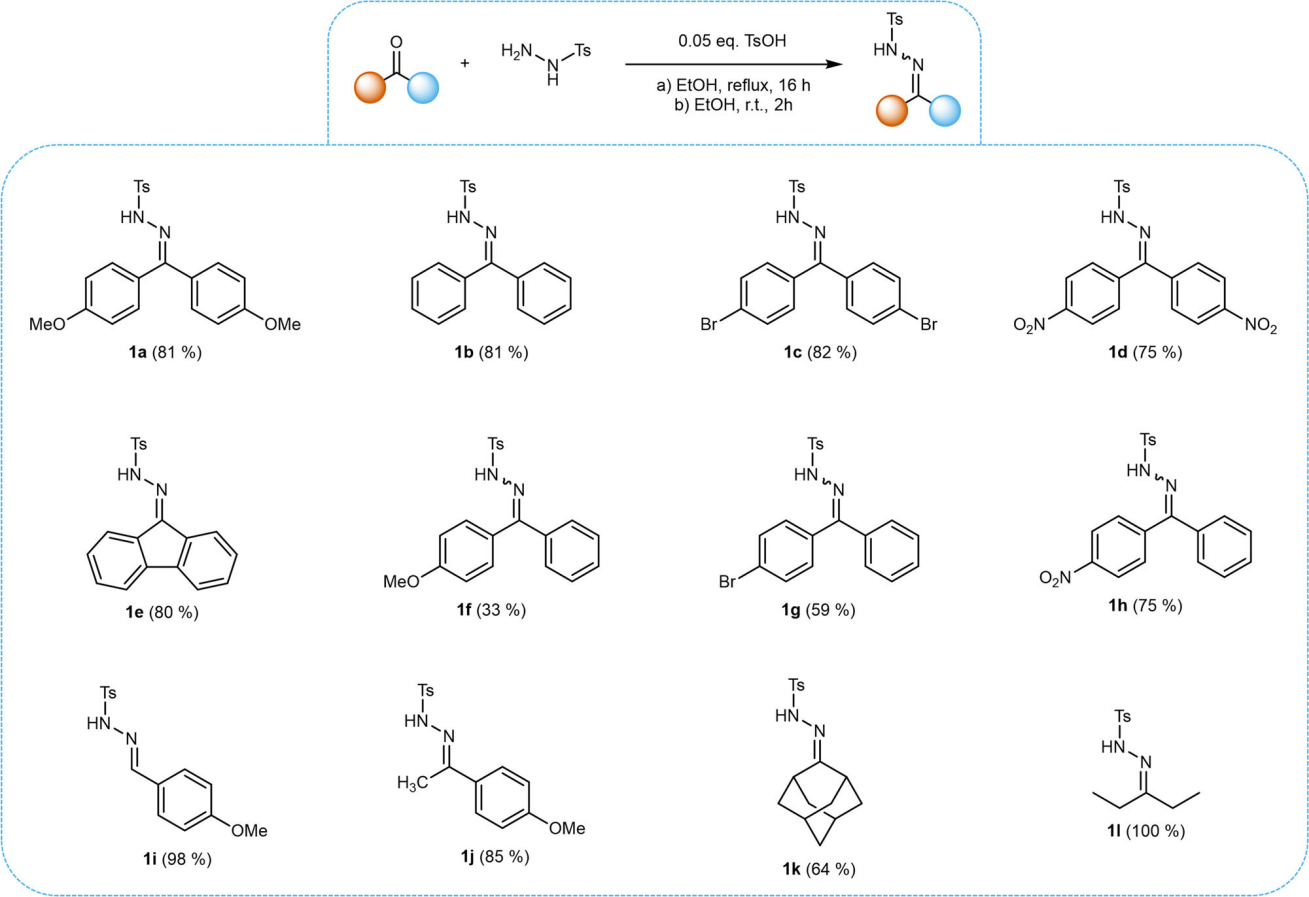
Scope of examined NTHs. NTHs were prepared from their respective carbonyl compounds. **1i,** **1j** and **1** **l** were prepared according to conditions b, all others were prepared using conditions a.

**Fig. 3:**
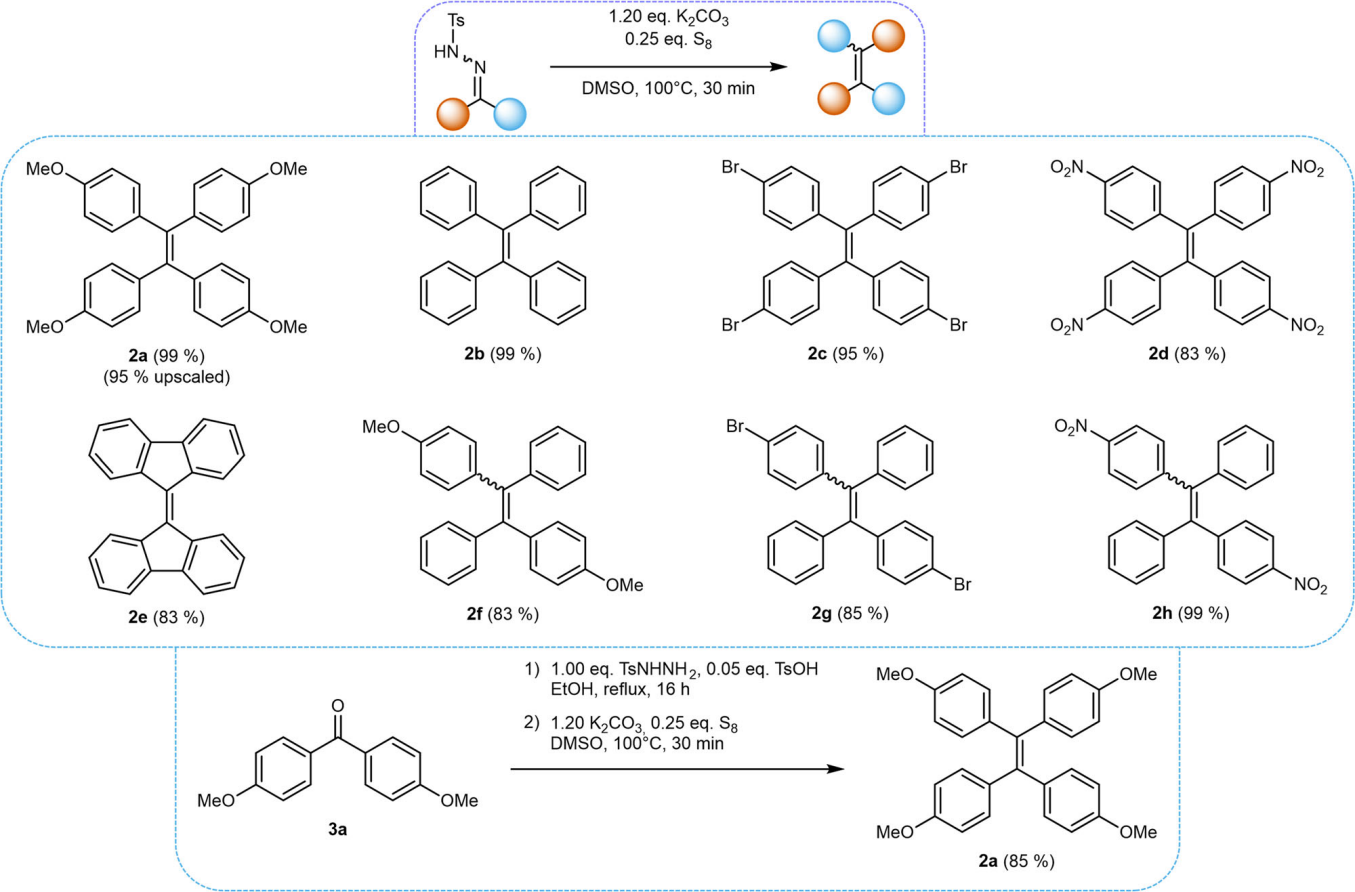
Homocoupling scope and one-pot synthesis of 2a. Homocouplings were conducted on 1 mmol scale respective to NTH. The NTHs **1i** – **1l** did not undergo selective homocoupling reactions. In the cases of **2f-h** the reported yield corresponds to a near-statistical mixture of *(E)-* and *(Z)-*isomers (50:50). The synthesis of **2a** was further upscaled to 5 mmol scale. The one-pot synthesis of **2a** from **3a** was also conducted on a 2 mmol scale.

In order to examine the feasibility of the alkylidene-homocoupling for a wider scope of substrates, a range of NTHs **1b-1i** were prepared from their respective carbonyl compounds **3b-3i** in yields ranging from 33 to 100% (Fig. 2). Homocoupling reactions were performed on a 1 mmol scale using the optimized reaction conditions described above (Fig. 3). Workup of the reaction mixtures was performed *via* direct column chromatography of the crude reaction mixture without the need for washing or extraction steps.

The benzophenone-derived NTHs **1a**-**1h** were all found to undergo smooth homocoupling, yielding their corresponding TPE derivatives in yields ranging from 80 to 99% (Fig. 3). This demonstrates a high efficiency of the method for generating both electron-rich and electron-poor, condensed cyclic, as well as synthetically useful di- or tetrahalogenated and -nitrated TPE derivatives.

Similarly to the McMurry reaction, the homocoupling of unsymmetrically substituted substrates **1f**-**1h** resulted in a near-statistical mixture of the respective *(E)-* and *(Z)-*isomers.

Aldehyde-based NTH **1i** and alkyl-group-bearing NTHs **1j-1l** did not undergo selective alkylidene-homocoupling, instead producing mixtures consisting of numerous different products as apparent by the appearance of various signals in the methoxy-region of the crude ^1^H-NMR spectra of **1i** and **1j**. In the cases of **1i** and **1j**, the homocoupling products could be qualitatively detected via GC-MS. However, due to the complicated nature of the product mixtures, no further workup and analysis was undertaken and the homocoupling method was deemed not feasible for NTHs derived from aldehydes and aliphatic compounds. Interestingly, all prepared TPE derivatives, except for **2e**, exhibited their characteristic solid-state-fluorescence behavior known as aggregation-induced emission (AIE) (see Supplementary Methods 1.3).

The homocoupling of **1a** to **2a** could be successfully upscaled to a gram scale (5 mmol), with the product **2a** being obtained in a yield of 95% in this case. Furthermore, a one-pot-procedure, combining the NTH synthesis and the homocoupling, was attempted. Herein, the crude NTH **1a** was used in the subsequent homocoupling without purification, allowing the isolation of TPE **2a** in a good yield of 85% in relation to ketone **3a**. This is slightly better compared to the two-step approach with intermediate isolation of NTH **1a**, which exhibits a combined yield of 81%. Considering NTH substrates that are more challenging to isolate, this one-pot approach can potentially be advantageous, while being also more feasible.

In comparison, the synthesis of the TPE derivatives **2a**–**2h** from their respective ketones **3a**-**3h** *via* a McMurry coupling has been reported several times with yields generally ranging from 70-99%^32–36^. The typically applied reaction conditions employ overstoichiometric amounts of titanium reagents and a reducing agent such as zinc under inert atmosphere. Workup is generally performed *via* extraction and subsequent column chromatography.

When considering a combination of both the NTH synthesis as well as the homocoupling, the herein presented method achieves combined yields of 28 to 85% in relation to the respective ketones, and was further shown to be feasible without the purification of the NTH. While these yields are generally slightly lower than those of the McMurry reaction, the showcased homocoupling comes with the advantages of less waste, lower cost and easier operatibility due to not requiring excessive amounts metallic reagents, extensive workup and inert conditions. Furthermore, the synthesis of nitro-group-bearing TPEs via homocoupling, which is out of scope of the McMurry reaction, was successfully performed. These advantages illustrate that the presented procedure can potentially serve as a viable addition to the repertoire of synthetic methods to access TPE derivatives.

### Mechanistic Study

In order to shed light upon the mechanism of this reaction, plausible pathways were considered and control experiments were developed. The mechanistic study was performed using the alkylidene-homocoupling of **1a** to **2a** under the optimized reaction conditions as the model reaction. First, the necessity of the used reagents, *i.e*. potassium carbonate and elemental sulfur, for the formation of **2a** was investigated. Thus, in addition to the unchanged homocoupling conditions (1.2 equivalents of base and 0.25 equivalents of sulfur, conditions A), control experiments were conducted in the absence of sulfur (conditions B), base (conditions C), or both sulfur and base (conditions D) from the reaction mixture. The use of DMSO-*d*_*6*_ as the solvent allowed for simple monitoring by following the changes in the crude ^1^H-NMR spectra.

**Fig. 4:**
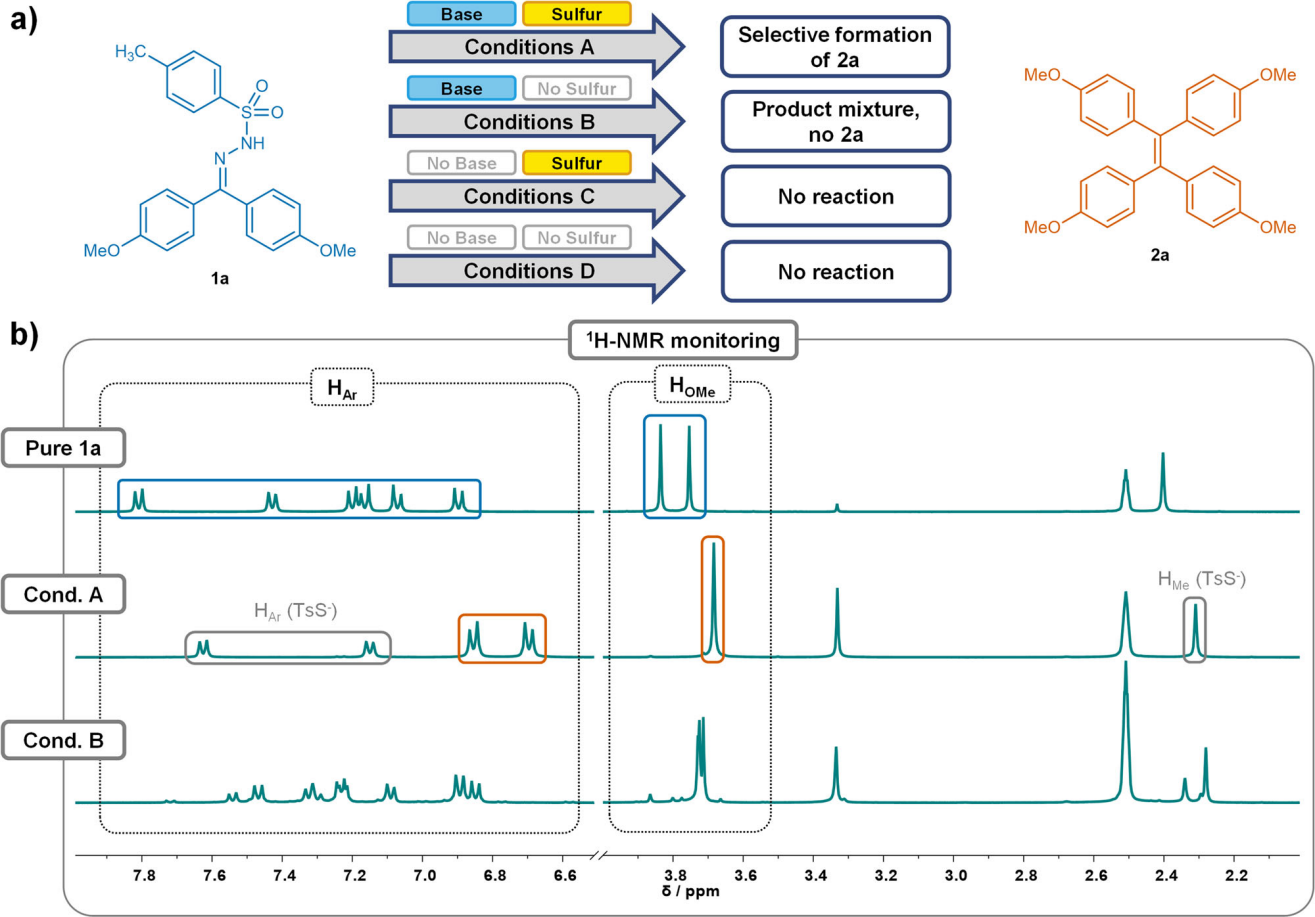
Mechanistic Control Experiments involving NTH 1a. **a** Schematic overview of the results of mechanistic control experiments using NTH **1a** under conditions A-D in DMSO-d_6_. **b** Excerpts of the ^1^H-NMR spectra of pure starting material **1a** (for comparison), and the crude ^1^H-NMR spectra obtained after 30 min under conditions A and B after 30 min. The aromatic and methoxy signals of **1a** (blue) and **2a** (orange) are assigned, along with the signals of *p*-toluenesulfinate formed under conditions **A** (gray).

In the presence of both sulfur and base (conditions A), **1a** was fully converted to **2a** after 30 min (Fig. 4). The remaining signals in the crude ^1^H-NMR spectrum can be attributed to *p*-toluenethiosulfonate, which can be formed by a side reaction of *p*-toluenesulfinate extruded upon NTH decomposition with elemental sulfur^37–39^. The absence of potassium carbonate in the experiments under conditions C and D leaves **1a** completely unreacted after 30 min, confirming the necessity of base to initiate the decomposition of the NTH. Under conditions B, full conversion of **1a** was observed due to the presence of the base after 30 min. However, no TPE **2a** could be detected in the resulting product mixture, indicating that the presence of elemental sulfur is vital for the success of the homocoupling starting from an NTH (Fig. 4).

Hence, as the presence of base was necessary for the conversion of NTH **1a**, it was assumed that the decomposition of the NTH takes place *via* elimination of *p*-toluenesulfinate under basic conditions, forming a diazo compound. The latter are known to be able to undergo further decomposition into carbenes under extrusion of N_2_ in aprotic media. This reactivity of NTHs is well-known for instance from Bamford-Stevens or Shapiro reactions^40–43^. At this point, it is worthwhile to mention that a possible Wanzlick dimerization of the supposed carbene would result in the formation of TPE **2a**. However, this pathway was ruled out for the present homocoupling reaction due to the necessity of sulfur for the formation of **2a**.

**Fig. 5:**
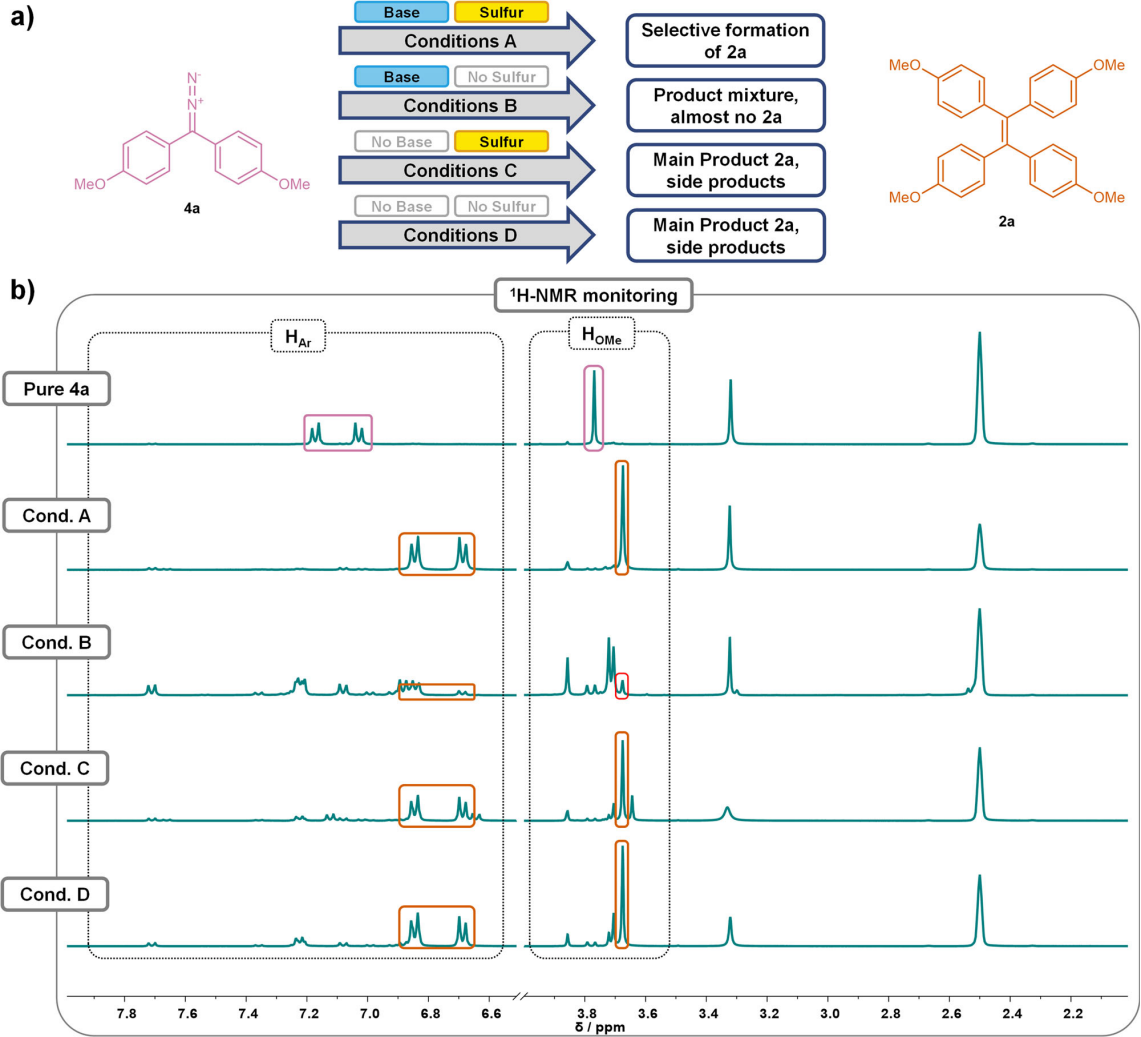
Mechanistic Control Experiments involving diazo compound 4a. **a** Schematic overview of the results of mechanistic control experiments using diazo compound **4a** under conditions **A**-**D** in DMSO-d_6_. Bottom: Excerpts of the ^1^H-NMR spectra of pure diazo compound **4a** (for comparison), and the crude ^1^H-NMR spectra obtained after 30 min under conditions **A**-**D** after 30 min. The aromatic and methoxy signals of **4a** (pink) and **2a** (orange) are assigned.

For further confirmation of these results, the corresponding diazo compound **4a** was synthesized and subjected to the same set of control experiments (Fig. 5). Similarly to the results obtained from the test reactions using NTH **1a**, almost exclusive formation of TPE **2a** from **4a** was observed in the presence of both sulfur and base (conditions A), supporting the assumption of an intermediate diazo compound in the mechanism of the homocoupling, which can be expected to further decompose into a carbene under nitrogen extrusion. Likewise, conditions B also resulted in the formation of a product mixture, further reinforcing the notion that sulfur is essential for a selective formation of TPE **2a**, if potassium carbonate is also present. In the remaining two test reactions under conditions C and D, TPE **2a** was found to be the main product, albeit among a larger amount of side products than under conditions A. This is presumably due to the higher initial concentration of the diazo compound **4a** in these test reactions in comparison to the actual homocoupling reaction. This could lead to a significantly higher carbene concentration in the mixture, possibly favoring a Wanzlick dimerization as a pathway for TPE **2a** formation under these changed conditions. However, since almost no TPE is formed from **4a** when potassium carbonate is present (conditions **A** and **B**), Wanzlick dimerization is clearly not the main pathway for the synthesis of **2a** under homocoupling conditions.

Since sulfur is obviously essential for the occurrence of homocoupling, other plausible pathways were considered. Elemental sulfur is well-known to be activated under basic conditions (e.g. potassium carbonate) in polar aprotic solvents like DMSO, which has been attributed to a nucleoplilic attack of the base on the cyclooctasulfur ring, resulting in the formation of a highly nucleophilic polysulfide chain, which exhibits a highly versatile reactivity profile^44–46^.

**Fig. 6:**
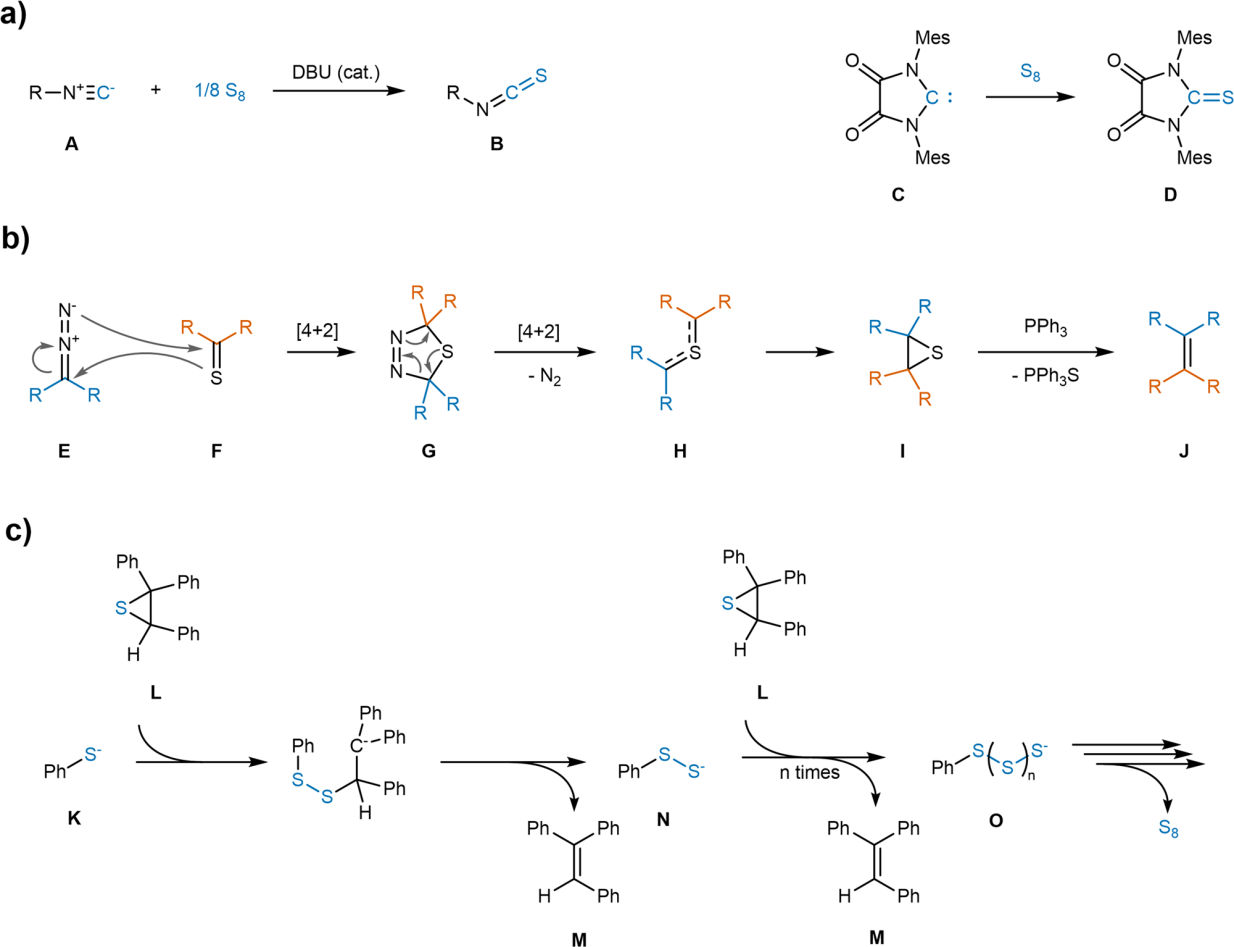
Relevant reactions and pathways for the elucidation of the homocoupling mechanism. **a** Reactions of isocyanides **A** and *N*-heterocyclic carbenes **C** with elemental sulfur under formation of C = S double bonds. **b** Pathway of the Barton-Kellogg reaction of a diazo compound **E** with a thioketone **F**, yielding an alkene **J** after thiirane **I** desulfurization with triphenylphosphine. **c** Desulfurization of 2,2,3-triphenylthiirane **L** into 1,1,2-triphenylethylene **M** catalyzed by thiophenolate **K**.

Recently, the reactivity of base-activated elemental sulfur with carbenes and carbenoids under formation of thiocarbonyl compounds (e.g. isothiocyanates **B** from isocyanides **A**, thioureas **D** from *N-*heterocyclic carbenes **C**) has been studied (Fig. 6a)^47–49^. In the present case, a reaction of the assumed intermediate carbene resulting from decompostion of the diazo compound with base-activated elemental sulfur would result in a thioketone.

Thioketones **F** and diazo compounds **E** are known to react in a [4 + 2]cycloaddition known as the Barton-Kellogg reaction, forming a 1,3,4-thiadiazoline **G**, which readily decomposes into a thiirane **I** upon nitrogen extrusion *via* an intermediate sulfur ylide **H** (Fig. 6b)^50–52^. Furthermore, direct reactions of carbenes with thioketones to form thiiranes are also known, making the formation of a thiirane under the alkylidene-homocoupling conditions conceivable^53^.

Barton-Kellogg reactions are themselves widely used reactions for the synthesis of highly congested alkenes, have however not seen widespread application in the synthesis of TPE derivatives. In such reactions, the use of a desulfurization agent is often necesssary to achieve the synthesis of alkene **J** from thiirane **I**. This is usually achieved using a stoichiometric reagent such as triphenylphosphine, however a catalytic desulfurization of 2,2,3-triphenylthiirane **L** using thiophenolate **K** as a strong nucleophilic catalyst has been reported as well^54^. The proposed mechanism of this catalytic desulfurization entails a nucleophilic attack of the thiophenolate **K** on the thiirane sulfur, followed by alkene **M** formation under elimination of a phenyl disulfide anion **N**. The latter can effect further desulfurizations, leading to a gradual buildup of a polysulfide chain **O** attached to the phenyl ring until the eventual extrusion of elemental sulfur (Fig. 6c). Furthermore, there are also several known instances of thiiranes spontaneously desulfurizing into their respective alkenes without any additional reagents required^54–56^.

The system of phenyl polysulfide species **O** bears high similarity to the supposed formation of polysulfide chains upon base-activation of elemental sulfur. Hence, while to our knowledge no report exists about the direct use of activated elemental sulfur to desulfurize thiiranes, the assumption was made that base-activated sulfur could potentially mediate thiirane desulfurization in the present homocoupling.

The viability of this newly assumed pathway was examined by synthesizing thioketone **5a** and thiirane **6a** in order to perform further control experiments using these substrates in similar manner as with NTH **1a** and diazo compound **4a**.

**Fig. 7:**
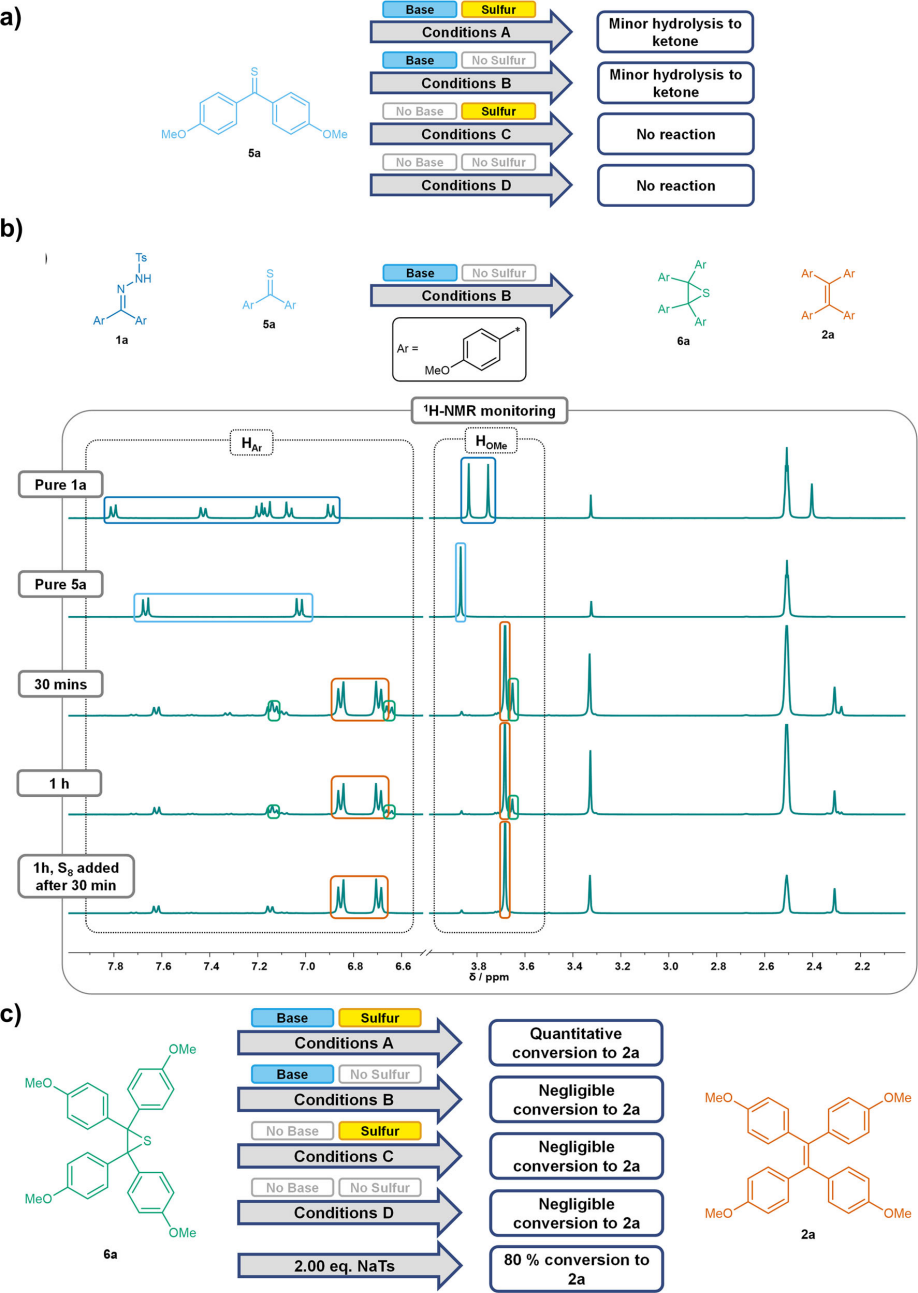
Mechanistic Control Experiments involving thioketone 5a and thiirane 6a. **a** Schematic overview of the results of mechanistic control experiments using thioketone **5a** under conditions A-D in DMSO-d_6_. **b** Schematic overview of mechanistic control experiments using NTH **1a** and thioketone **5a** under conditions **B** in DMSO-d_6_, along with ^1^H-NMR spectra of pure NTH **1a** and thioketone **5a** (for comparison), and the crude ^1^H-NMR spectra obtained after 30 min, 1 h, and 1 h with elemental sulfur having been added after 30 min. The aromatic and methoxy signals of **1a** (blue), **2a** (orange), **5a** (light blue) and **6a** (green) are assigned. **c** Schematic overview of the results of mechanistic control experiments using thiirane **6a** under conditions **A**-**D** and in a test reaction with 2.00 eq. of sodium *p-*toluenesulfinate in DMSO-d_6_.

When thioketone **5a** alone was subjected to the different control experiment conditions A-D, no reaction except minor formation of the corresponding ketone **3a** in the reactions involving potassium carbonate was detected (conditions A and B), which is presumably due to hydrolysis caused by the presence of small amounts of water or bicarbonate (Fig. 7a). This suggests that the formation of a thioketone alone does not yield a suitable reaction pathway towards the product TPE.

Hence, an additional test reaction using an equal stoichiometry of NTH **1a** and thioketone **5a** was conducted in the presence of base (conditions **B**) in order to examine if a reaction between the decomposition products of the NTH and the presynthesized thioketone would occur. Indeed, both the NTH and the thioketone were completely converted after 30 min, with TPE **2a** being the main product, along with a significant amount of thiirane **6a** (Fig. 7b). Interestingly, it was found that the tosyl species formed upon decomposition of the NTH was mostly present as p-toluenethiosulfonate, despite the absence of elemental sulfur, leading to the assumption that *p*-toluenesulfinate acts as a stoichiometric desulfurizing agent to generate TPE **2a** from thiirane **6a**. However, even after elongated reaction times, quantitative conversion of thiirane **6a** into TPE **2a** could not be achieved. Therefore, in another control experiment, the same reaction was conducted with sulfur being added after 30 min. In this case, quantitative TPE formation was observed, suggesting that base-activated sulfur can mediate the thiirane desulfurization more efficiently (Fig. 7b). These results were further validated by exposing thiirane **6a** to the various control experiment conditions (Fig. 7c). Quantitative conversion of **6a** to **2a** was only observed when both sulfur and base were used, further validating the assumption that base-activated sulfur can mediate thiirane desulfurization in similar fashion as in the previous report using thiophenolate^54^. Accordingly, in all other cases of control experiments, only negligible desulfurization was observed, indicating that in the case of thiirane **6a**, no spontaneous desulfurization can occur under the reaction conditions. Additionally, a reaction of **6a** with two equivalents of sodium *p*-toluenesulfinate resulted in only 80% conversion of **6a** to **2a** after 30 min, further demonstrating the lower effectiveness of thiirane desulfurization by sulfinate ions.

**Fig. 8:**
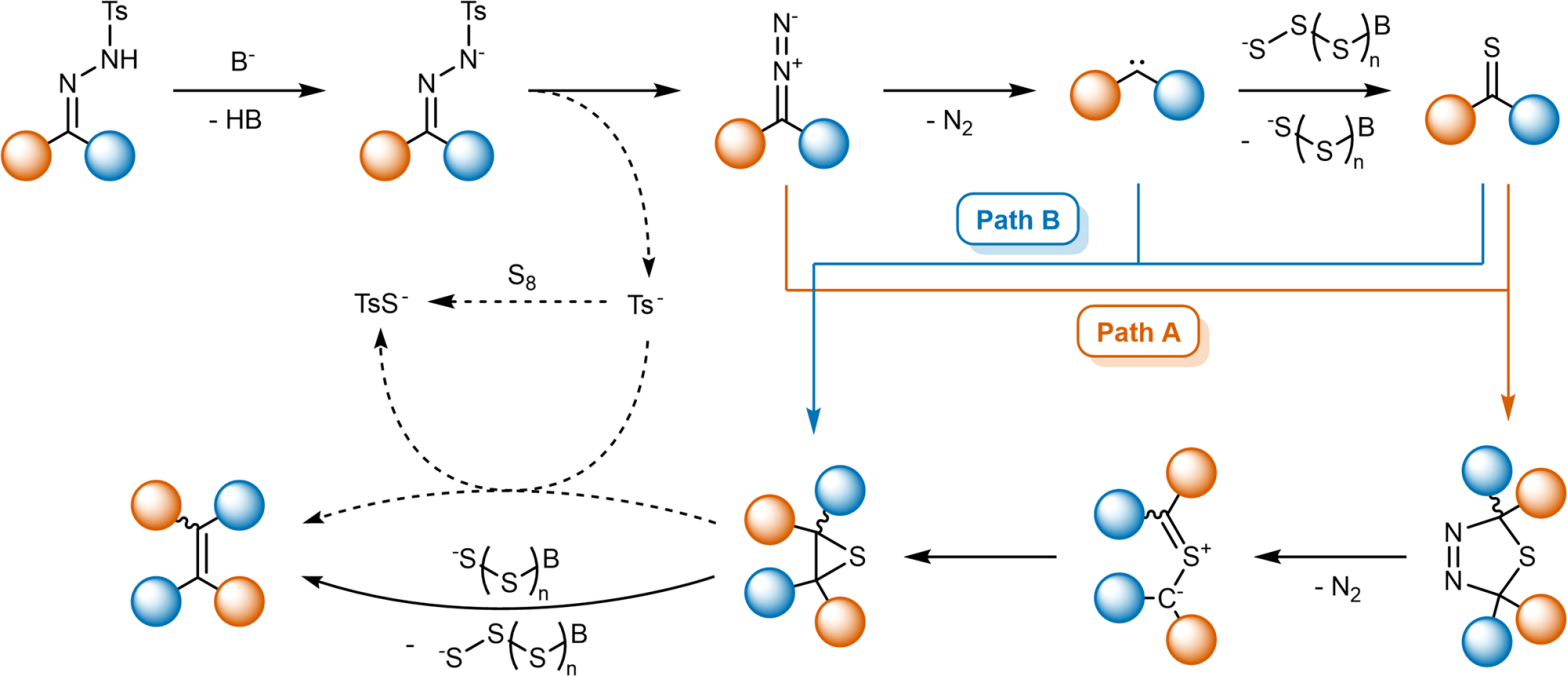
Proposed mechanism of the herein presented alkylidene-homocoupling reaction. Path A (red arrow) entails a Barton-Kellogg reaction and path B (blue arrow) a direct thiirane formation from carbene and thioketone. The desulfurization is mainly mediated by a polysulfide chain, while eliminated p-toluenesulfinate can also enable product formation (dashed reaction sequence). The colored spheres represent aryl moieties.

Based on these findings, we propose a plausible reaction mechanism for the alkylidene-homocoupling of NTHs with base-activated sulfur (Fig. 8). The reaction initiates *via* a base-induced decomposition of the reactant NTH in similar fashion to literature known reactions, such as the Bamford-Stevens reaction, resulting in the formation of a carbene, which then reacts with activated elemental sulfur to yield a thioketone. This is followed by the formation of a thiirane, either by a Barton-Kellogg reaction between diazo compound and thioketone followed by nitrogen extrusion and recyclization (path A), or *via* direct reaction of carbene and thioketone (path B). The final alkene product is obtained by desulfurization of the thiirane, mainly mediated by activated elemental sulfur. As a parallel reaction, the sulfinate extruded upon NTH decomposition further reacts to a thiosulfonate ion, either by direct reaction with elemental sulfur, or by desulfurizing the thiirane.

This proposed mechanism may also explain the low selectivity of the homocoupling reaction for aliphatic and aldehyde-derived substrates, since their respective carbenes experience significantly less stabilization due to the smaller degree of π-conjugation at the carbene center, facilitating the occurrence of undesirable side reactions^43,57^. Furthermore, thioaldehydes and aliphatic thioketones are also known to be more reactive than aromatic thioketones, being significantly more prone to polymerization reactions^58–60^. Overall, the higher degree of instability in these intermediates presumably impedes a selective homocoupling for these substrates.

### Alkylidene-cross-coupling

In the previous section, the mechanism of the alkylidene-homocoupling reaction was shown to include the in situ formation of a thioketone. Furthermore, the homocoupling was also shown to proceed when using the thioketone as an additional reactant. Therefore, using thioketones and NTHs bearing different substituents, the synthesis of alkylidene-cross-coupled TPEs was attempted.

The cross-coupling conditions were adapted from the corresponding test reaction conducted in the course of the mechanistic study (Fig. 7b), i.e. thioketone and NTH were reacted only with potassium carbonate in order to prevent the competing NTH homocoupling. The thioketone was used in a very slight excess of 1.02 equivalents. Sulfur was added after 30 min of reaction time and the reaction was continued for another 30 min in order to ensure complete thiirane desulfurization. Thus, sulfur was only used in its capacity as desulfurization reagent in this cross-coupling approach. Essentially, this kind of reaction protocol can be considered an upgraded Barton-Kellogg reaction, since it does not require the handling of a diazo compound.

**Fig. 9:**
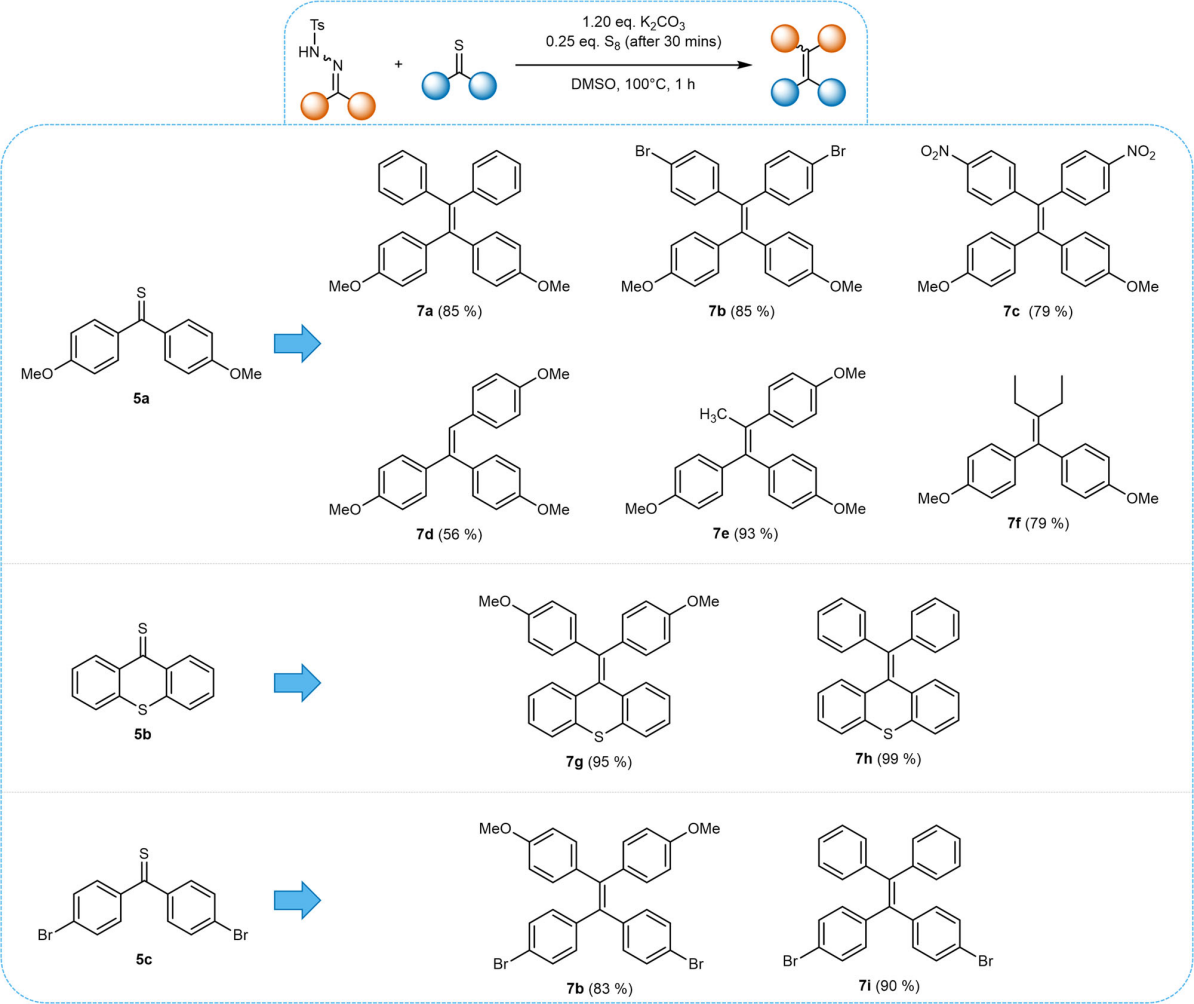
Scope of the herein presented alkylidene-cross-coupling. Cross couplings were condcuted on 0.5 mmol scale (according to NTH) using 1.02 equivalents of thioketone.

The cross-coupling method also proved to be highly viable for the synthesis of TPE derivatives, as shown by successful coupling of thioketone **5a** with a range of benzophenone-derived NTHs **1b**-**1d**, yielding the cross-coupled TPEs **7a**–**7c** in good yields ranging from 79 to 85% (Fig. 9). Similarly to the homocoupling method, the compatibility of the procedure with NTHs bearing electron-donating and electron-withdrawing functional groups, particularly the easily reducible nitro group, was confirmed. Furthermore, TPE derivatives **7g** – **7i** were synthesized in good yields *via* cross-coupling of thioketones **5b** and **5c** with NTHs **1a** and **1b** (82-95%). Notably, TPE derivative **7a** could be successfully synthesized both from **5a** and **1c** as well as **5c** and **1a**, illustrating the flexibility of the method from both the NTH and thioketone sides. Similarly to the TPE derivatives accessed *via* the homocoupling route, the compounds **7a**-**c** and **7g**-**I** exhibited AIE behavior (see Supplementary Methods 1.4a for characterization).

Additionally, it was found that the aliphatic and aldehyde-derived NTHs **1i,** **1j** and **1l**, which did not undergo selective alkylidene-homocoupling, could be successfully cross-coupled with thioketone **5a**, yielding compounds **7d**–**7f** in moderate to excellent yields (56–93%). Thus, the successful introduction of alkyl substituents expands the scope of the alkylidene-cross-coupling to a wider range of TSAs beyond TPEs. It has to be noted that the thioketones used in this work were synthesized under an inert atmosphere, however, they were found to be sufficiently stable towards air and moisture to perform the cross-coupling reactions without an inert atmosphere, mantaining the feasbile and simple reaction set-up.

In comparison to other prevalent synthetic routes toward alkylidene-cross-coupled TSAs, the herein described method can be more efficient and feasible. For instance, the utility of the McMurry coupling of two different benzophenone derivatives is greatly hindered by the competing homocoupling reaction. This results in the formation of statistical mixtures of all possible products, greatly complicating the workup and leading to low yields^19,34^.

Due to the infeasibility of the McMurry coupling, significantly more elaborate methods involving multiple reaction steps are required for a selective synthesis of alkylidene-cross-coupled alkenes, often relying on transition-metal-catalyzed cross-couplings (compare Fig. 1). The herein presented method for the synthesis of alkylidene-cross-coupled TSAs poses a similar synthetic effort, since the preparations of NTH and thioketone as well as the subsequent coupling require three reaction steps, all of which are carried out without the need for hazardous and expensive (organo)metallic reagents.

## Conclusion

Two methods for the synthesis of highly substituted alkenes *via* olefination of *N*-tosylhydrazones (NTHs) are presented. The first method entails a sulfur-mediated alkylidene-homocoupling of aromatic NTHs and was shown to effectively generate a range of TPE derivatives with different substituents. Compared to classic approaches like McMurry reactions or transition-metal-catalyzed cross-couplings, this two-step reaction can be advantageous in terms of feasibility (easy reaction setup, cheap and benign additives, less elaborate work-up, tolerance of reduction-labile functional groups). Additionally, the possibility to perform both reaction steps in a one-pot approach further adds to the viability of the procedure. The second method uses a thioketone as an additional substrate, enabling the synthesis of alkylidene cross-coupled alkenes with an additional tolerance for aliphatic substituents, while maintaining similar benefits as its homocoupling pendant in comparison to alternative procedures. Furthermore, a mechanistic study for the homocoupling reaction was conducted, confirming the formation of an intermediate thiirane from in situ generated diazo compounds / carbenes and thioketones. The product is formed via desulfurization of said thiirane mediated by base-activated sulfur. The cross-coupling method can hence be considered a variation to the already established Barton–Kellogg reaction, since it entails the in situ generation of the required diazo compounds. Both methods were shown to be viable alternatives to other established procedures often used to access TSAs, particularly TPE derivatives, which are interesting target molecules due to their aggregation-induced fluorescence properties. The synthetically useful scope of both methods paired with the high obtained yields and the operative simplicity potentially makes the showcased reactions a viable addition to the toolbox for the synthesis of highly substituted double bonds.

## Methods

### General procedures for the synthesis of NTHs from carbonyl compounds

#### Procedure A

931 mg tosyl hydrazide (5.00 mmol, 1.00 eq.) were suspended in 10 mL of ethanol and 5.00 mmol (1.00 eq.) of the carbonyl component along with 47.5 mg of *p*-toluenesulfonic acid monohydrate (0.25 mmol, 0.05 eq.) were added. The mixture was refluxed overnight. Afterwards, the mixture was cooled down to −20 °C, the precipitate was filtered off and washed with cold ethanol. The filtered solid was dried *in vacuo*.

#### Procedure B

931 mg tosyl hydrazide (5.00 mmol, 1.00 eq.) were suspended in 10 mL of ethanol and 5.00 mmol (1.00 eq.) of the carbonyl component were dissolved in 10 mL of ethanol. The mixture was stirred at room temperature for 2 h. Afterwards, the solvent was removed in vacuo.

### General procedure for alkylidene-homocouplings of NTHs

1.00 mmol of the *N-*tosylhydrazone was dispersed in 2.00 mL of dry DMSO and 64.1 mg of elemental sulfur (0.25 mmol, 0.25 eq.) was added. The mixture was heated to 100°C and 166 mg of anhydrous potassium carbonate (1.20 mmol, 1.20 eq.) were added. The mixture was stirred at 100°C for 30 min. Afterward, the crude mixture was transferred directly onto a column (packed in pure cyclohexane) and the product was isolated *via* column chromatography.

### General procedure for the alkylidene-cross-coupling of NTHs and thioketones

0.50 mmol (1.00 eq.) of the *N*-tosylhydrazone were dissolved in 2 mL of dry DMSO and 0.51 mmol of the thioketone (1.02 eq.) were added. The mixture was heated to 100 °C and 82.92 mg (0.60 mmol, 1.20 eq.) of potassium carbonate were added. After stirring at 100 °C for 30 min, 32.1 mg (0.125 mmol, 0.25 eq.) of elemental sulfur were added and stirring at 100°C was continued for another 30 min. Afterward, the crude mixture was transferred directly onto a column (packed in pure cyclohexane) and the product was isolated *via* column chromatography.

For all further procedures, see Supplementary Methods.

For NMR Spectra, see Supplementary Figs. 1–80.

For Fluorescence data, see Supplementary Table 1 and Supplementary Figs. 81–93.

For further schemes associated with the mechanistic study, see Supplementary Figs. 94–99.

### Supplementary information

Peer Review File

Supplementary Information

## Data Availability

Data are included within the article or its supplementary information and can be provided by the corresponding author upon reasonable request.
