# Supplementary material for: Synthesis of highly substituted alkenes by sulfur-mediated olefination of N-tosylhydrazones
Source: Commun Chem. 2023 Nov 18;6:255. doi: 10.1038/s42004-023-01058-2 (PMC10657425; doi:10.1038/s42004-023-01058-2)
Supplement: Supplementary file 2 — Supplementary Information [file 42004_2023_1058_MOESM2_ESM.pdf]

SUPPLEMENTARY INFORMATION

Synthesis of highly substituted olefins by sulfur-mediated olefination of *N*-tosylhydrazones

P. Conen, R. Nickisch, M. A. R. Meier *

Table of Contents

1 Supplementary Methods 2
1.1 Instrumentation and Practical Methodology 2
1.2 Starting Materials 5
1.3 Synthesis and Characterization 7
1.3.1 Carbonyl compounds.. 7
1.3.2 *N*-Tosylhydrazones.. 17
1.3.3 Thioketones 42
1.3.4 Mechanistic intermediates 48
1.3.5 Homocoupling products 54
1.3.6 Cross-coupling products 71
1.4 Fluorescence Measurements 90
1.5 Mechanistic Study 96
2. Supplementary References 103

1 Supplementary Methods

1.1 Instrumentation and Practical Methodology

Nuclear Magnetic Resonance Spectroscopy (NMR)

^{1}H and ^{13}C-NMR spectra were recorded on a *BRUKER Avance 400* NMR spectrometer at 400 MHz for ^{1}H and 101 MHz for ^{13}C-NMR. Unless otherwise stated, all spectra were recorded at ambient temperature. The chemical shift was reported in parts per million (ppm) and referenced to characteristic signals of deuterated solvents *e.g.*, DMSO-d_6 at 2.50 ppm for ^{1}H (39.52 ppm for ^{13}C) and chloroform-d_1 at 7.26 ppm for ^{1}H (77.16 ppm for ^{13}C). ^{13}C-NMR spectra were recorded with ^{1}H-decoupling. For centrosymmetrical signals, the centroid shift was given and for multiplets, the signal range was reported. The multiplets arising from spin-spin coupling were abbreviated as follows: s = singlet, d = doublet, t = triplet, q = quartet, quint. = quintet, sext. = sextet, m = multiplet, br = broad signal. Coupling constants J were reported in Hz. Furthermore, the 2D-NMR methods ^{1}H,^{1}H-correlated spectroscopy (COSY), ^{1}H,^{13}C-heteronuclear single quantum coherence spectroscopy (HSQC), ^{1}H-^{13}C-heteronuclear multiple bond correlation spectroscopy (HMBC) and ^{1}H-^{1}H-nuclear Overhauser enhancement spectroscopy (NOESY) were performed for signal assignment and structure elucidation.

Infrared Spectroscopy (IR)

IR measurements were performed on a *Bruker ALPHA attenuated total reflection (ATR)* IR spectrometer in a range 4000 to 500 cm^{-1} at ambient temperature. The bands were characterized according to their transmission (T) intensity as follows: vs = very strong (0 – 10% T), s = strong (10 – 40% T), m = medium (40 – 70% T), w = weak (70 – 90% T), vw = very weak (>90% T). Broad signals were labelled with br.

UV/Vis Spectroscopy

Absorption spectroscopy was performed on an *Agilent Cary 3500 UV-Vis* multicell spectrometer with a Peltier Element. Spectra were recorded at a cell temperature of 20 °C in a range of 200 nm to 800 nm in 1 nm intervals using a scanning speed of 600 nm/min. Samples were measured using 1 cm *STARNA* quartz cuvettes in a concentration of 100 µM in tetrahydrofuran.

Fluorescence Spectroscopy

Emission spectra were recorded on a *HORIBA FluoroMax Plus* spectrometer. Spectra were recorded at a cell temperature of 20°C in 1 nm intervals using an increment time of 200 ms and an integration time of 100 ms. Excitations wavelengths and slit sizes were adapted for each sample individually. Samples were prepared by injecting 10 µL of a 10 mM stock solution of the analyte in THF into 5 mL of water with 10 vol% THF under rapid stirring (corresponding to 20 µM of analyte in water with 10 vol% THF). For further details, see Supplementary Chapter 1.4.

High Resolution Mass Spectrometry (HRMS)

Fast Atom Bombardment (FAB) and electron impact ionization (EI) mass spectra were recorded using a *Finnigan MAT* mass spectrometer. A *Q Exactive* Orbitrap mass spectrometer (*Thermo Fisher Scientific*) equipped with a *HESI II* probe was employed to record high resolution electrospray ionization (ESI) mass spectra. Calibration was carried out in the *m/z* range 74 – 1822 using premixed calibration standards (*Thermo Fisher Scientific*). The spectra were interpreted by indicating the *m/z* of the molecular peak $[M]^+$ or the protonated molecular peak $[M+H]^+$ as well as characteristic fragments along with their relative intensities.

Gas Chromatography / Mass Spectrometry (GC-MS)

GCMS measurements were performed on a *Varian 431 GC* instrument with a capillary column *FactorFour VS-5 ms* (30 m × 0.25 mm × 0.25 mm) coupled with a *Varian 210* electron impact (EI) ion trap mass spectrometer. Scans were performed from 40 to 650 *m/z* at a rate of 1.0 scans per second. The oven temperature program was initial temperature 95°C, ramp at 15°C min^{-1} to 220°C, hold for 4 min, ramp at 15°C min^{-1} to 300°C, hold for 2 min. The injector transfer line temperature was set to 250°C. Measurements were performed in split-split mode (split ratio 50:1) using He as carrier gas with a flow rate of 1.0 mL min^{-1}.

Thin Layer Chromatography (TLC)

TLC analyses were performed using aluminium plates coated with silica and a fluorescence indicator (*Merck, Silica Gel 60, F 254, layer thickness 0.25 mm*). The spots were visualized by examining their fluorescence under UV light (λ = 365 nm) or their fluorescence quenching at a wavelength of 254 nm. If necessary, the TLC plates were stained with a potassium permanganate staining solution (1.50 g $KMnO_4$, 10.0 g K_2CO_3 and 0.50 g NaOH in 200 mL water).

Column Chromatography

Column Chromatography was performed as the primary workup method using *Silica Gel 60* (*Merck*, mesh size 40 – 63 µm) and quartz sand (*Bernd Kraft*) as column material and HPLC grade solvents as mobile phase.

1.2 Starting Materials

Unless otherwise specified, all chemicals were used as received.

Substance	Supplier	Purity
4,4'-Dimethoxybenzophenone	Acros Organics	97%
p-Toluenesulfonyl hydrazide	Sigma-Aldrich	97%
p-Toluenesulfonic acid monohydrate	Acros Organics	>99%, extra pure
Sodium hydride, dispersion in mineral oil	Sigma Aldrich	60%
1,8-Diazabicyclo[5.4.0]undec-7-ene (DBU)	TCI	>98%
Potassium carbonate, anhydrous	Fluka	99%, vacuum-oven-dried before use
Caesium carbonate	Sigma-Aldrich	99%, vacuum-oven-dried before use
γ-Butyrolactone (GBL)	Sigma-Aldrich	>99%
N,N-Dimethylformamide (DMF)	VWR	HPLC grade
N-Methyl-2-pyrrolidone (NMP)	Thermo Fisher	99%, extra pure
Sulfur	BASF	technical
Benzophenone	Sigma-Aldrich	99%
9-Fluorenone	Acros Organics	99%
4-Bromobenzophenone	Alfa Aesar	98%
4-Nitrobenzophenone	Sigma-Aldrich	99%, purified by flash column chromatography before use (cyclohexane/EtOAc 10:1)
4-Hydroxybenzophenone	Acros Organics	>98%
4-Methoxybenzaldehyde	abcr	98%
2-Adamantanone	Acros Organics	98%
3-Pentanone	Sigma-Aldrich	>99%
Thioxanthene-9-one	Sigma-Aldrich	98%
Bromobenzene	Acros Organics	99%
4-Bromobenzoyl chloride	TCI	98%
Aluminium chloride	Acros Organics	98.5%, anhydrous
Diphenylmethane	Sigma-Aldrich	For synthesis
Tetrabutylammonium hydrogensulfate	Fisher Scientific	99%
Potassium permanganate	Supelco	krist,, EMPLURA®
Methyl Iodide	Acros Organics	98%, for synthesis
Anisole	Sigma-Aldrich	99%
Acetyl Chloride	Sigma-Aldrich	99%, for synthesis
Hydrazine monohydrate	Sigma-Aldrich	99%
Lawesson's reagent	Acros Organics	99%
Sodium *p*-toluenesulfinate (NaTs)	Sigma-Aldrich	95%
Dichloromethane (DCM)	VWR	HPLC grade
Ethanol, absolute	VWR	HPLC grade
Methanol	VWR	HPLC grade
Toluene	Acros Organics	>99.85%, extra dry
Acetonitrile	Fisher Scientific	>99.9%, HPLC grade
Diethyl ether	Oqema	Pharmaceutical grade

Dimethyl sulfoxide (DMSO)	Acros Organics	>99.7%, extra dry
Ethyl acetate	VWR	HPLC grade
Cyclohexane	VWR	HPLC grade
Triethylamine	Fluorochem	anhydrous
Tetrahydrofuran (THF)	Sigma-Aldrich	>99.8%, with 2.5 ppm BHT as stabilizer, distilled before use
Dimethylsulfoxide-d_6	Eurisotop	>99.9 atom-% D
Chloroform-d	Eurisotop	>99.9 atom-% D
Sodium hydroxide	Bernd Kraft	For analysis
Sodium bicarbonate	Fisher Scientific	>99%
Hydrochloric acid, 37%	Fisher Scientific	For analysis
Sulfuric acid	Fisher Scientific	For analysis
Nitric acid, 65%	Fisher Scientific	For analysis
Acetic acid	Fluka	Puriss, p.a.
Sodium sulfate, anhydrous	Thermo	Extra pure, 99%
Sodium sulfite, anhydrous	Fisher Scientific	>97%
Manganese dioxide, activated	Fluka	90%, technical
Potassium hydroxide	Bernd Kraft	For analysis
Celite®	Acros Organics	Acid-washed

1.3 Synthesis and Characterization

1.3.1 Carbonyl compounds

***Synthesis of 4,4'-dibromobenzophenone* (3c)**

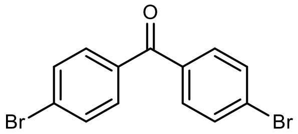

In a Schlenk flask under argon atmosphere, 2.00 g of aluminium chloride (15.0 mmol, 1.20 eq.) were dispersed in 10 mL of bromobenzene and a solution of 2.74 g *p*-bromobenzoyl chloride (12.5 mmol, 1.00 eq.) in 10 mL of bromobenzene was added dropwise. The mixture was stirred for 60 hours at 65 °C. Subsequently, the reaction mixture was poured onto 50 g of ice and 50 mL of DCM along with 200 mL of concentrated hydrochloric acid were added. The aqueous phase was extracted three times with 50 mL each of DCM and the combined extracts were washed once each with 1 M NaOH and water. The extracts were dried over sodium sulfate and the solvent was removed under reduced pressure. The product was obtained as a slightly yellow, crystalline solid in a yield of 82% (3.48 g, 10.2 mmol). Procedure adapted and modified from Supplementary Reference [1].

^{1}H NMR (400 MHz, DMSO-d_6) δ/ppm = 7.82 – 7.74 (m, 4H), 7.71 – 7.63 (m, 4H).

^{13}C NMR (101 MHz, DMSO-d_6) δ/ppm = 193.99, 135.65, 131.72, 131.60, 126.93.

IR (ATR platinum diamond): v/cm^{-1} = 1643 (vs), 1580 (vs), 1563 (m), 1479 (w), 1395 (m), 1378 (w), 1368 (w), 1312 (w), 1302 (m), 1284 (s), 1273 (s), 1174 (w), 1144 (w), 1111 (w), 1105 (w), 1070 (s), 1010 (s), 967 (w), 924 (s), 854 (vs), 825 (s), 749 (vs), 695 (w), 675 (w), 662 (s), 623 (w), 485 (m), 477 (s), 436 (m).

ESI-HRMS m/z: $[M+H]^+$ calculated for $C_{13}H_8O^{79}Br^{81}Br$ = 340.8994, found 340.8990.

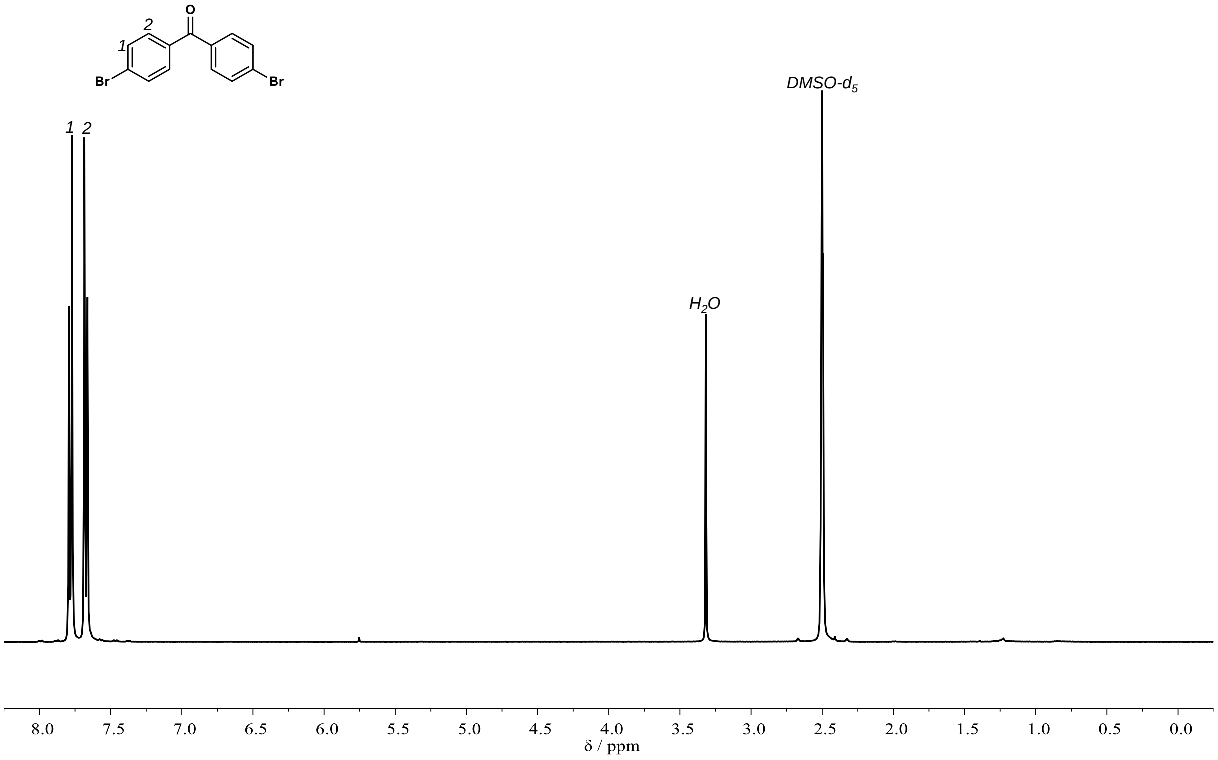

Supplementary Figure 1: ^{1}H-NMR spectrum of 3c.

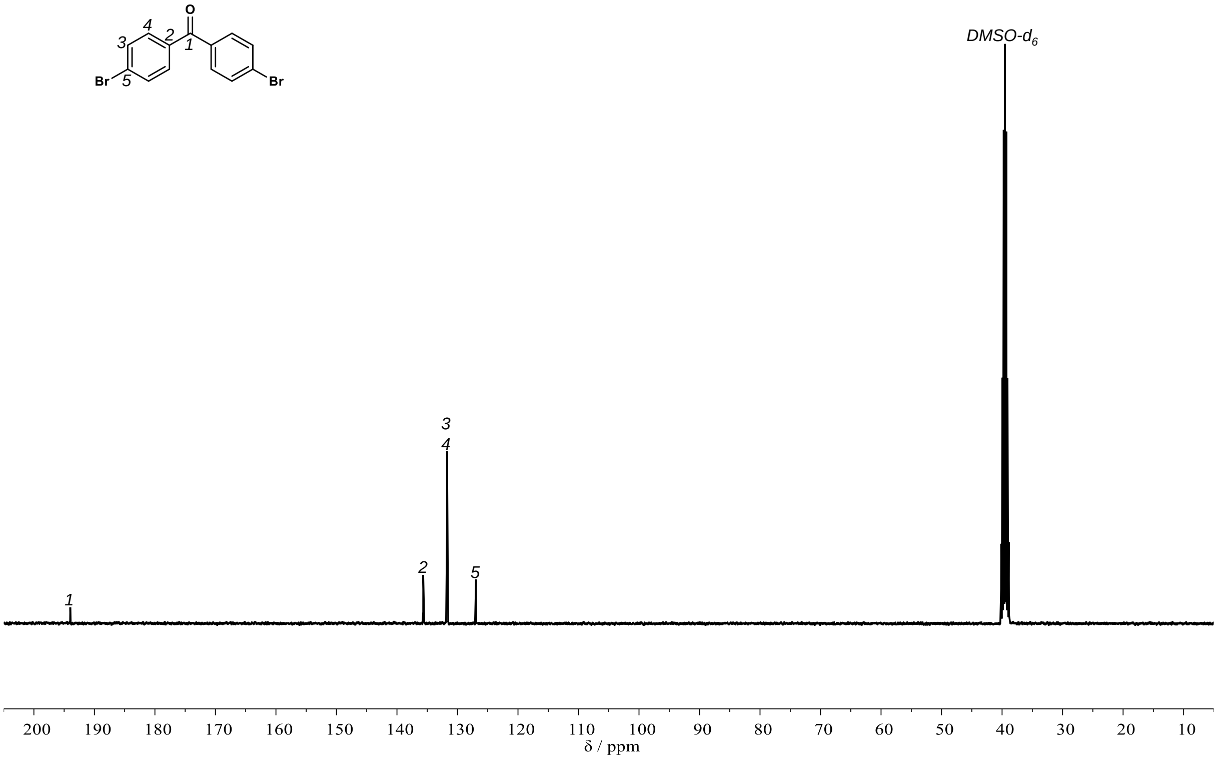

Supplementary Figure 2: ^{13}C-NMR spectrum of 3c.

***Synthesis of 4,4‘-dinitrodiphenylmethane* (8)**

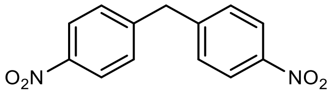

In a 1000 mL round bottom flask, 115 mL of 65% nitric acid were added dropwise to 105 mL of sulfuric acid. Afterwards, a solution of 30.0 g diphenylmethane (178 mmol, 1.00 eq.) in 70 mL of acetic acid was added dropwise while maintaining a temperature lower than 10°C. After complete addition, the mixture was stirred in an ice bath for 2 hours. Subsequently, the crude mixture was poured onto 500 g of ice, the precipitate was filtered off and washed three times each with water and ethanol. The crude product was recrystallized from 350 mL of toluene to yield 4,4’-dinitrodiphenylmethane as a slightly yellow, crystalline solid in a yield of 30% (13.7 g, 53.1 mmol). Procedure adapted from Supplementary reference [2].

^{1}H NMR (400 MHz, $CDCl_3$) δ/ppm = 8.15 – 8.02 (m, 4H), 7.31 – 7.24 (m, 2H), 4.12 (s, 2H).

^{13}C NMR (101 MHz, $CDCl_3$) δ/ppm = 146.96, 146.68, 129.78, 124.10, 41.38.

IR (ATR platinum diamond): ν/cm^{-1} = 3106 (vw), 3079 (vw), 2941 (vw), 2836 (vw), 2448 (vw), 1927 (vw), 1798 (vw), 1604 (w), 1592 (w), 1506 (vs), 1446 (w), 1337 (vs), 1318 (vs), 1290 (m), 1174 (w), 1107 (s), 1014 (w), 973 (vw), 882 (w), 858 (m), 841 (w), 817 (w), 802 (m), 743 (s), 706 (s), 685 (w), 640 (w), 634 (w), 527 (w), 508 (m), 469 (w).

ESI-HRMS m/z: $[M+H]^+$ calculated for $C_{13}H_{10}N_2O_4$ = 259.0714, found 259.0712.

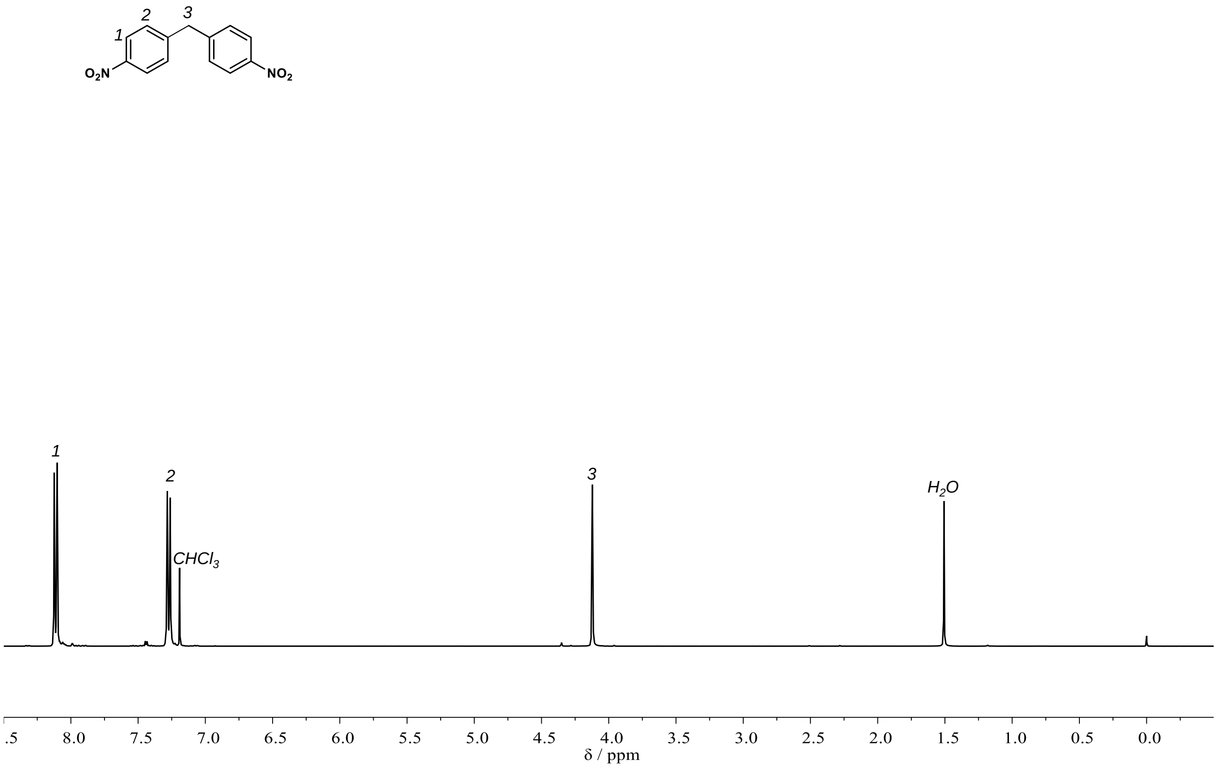

Supplementary Figure 3: ^{1}H-NMR spectrum of 8.

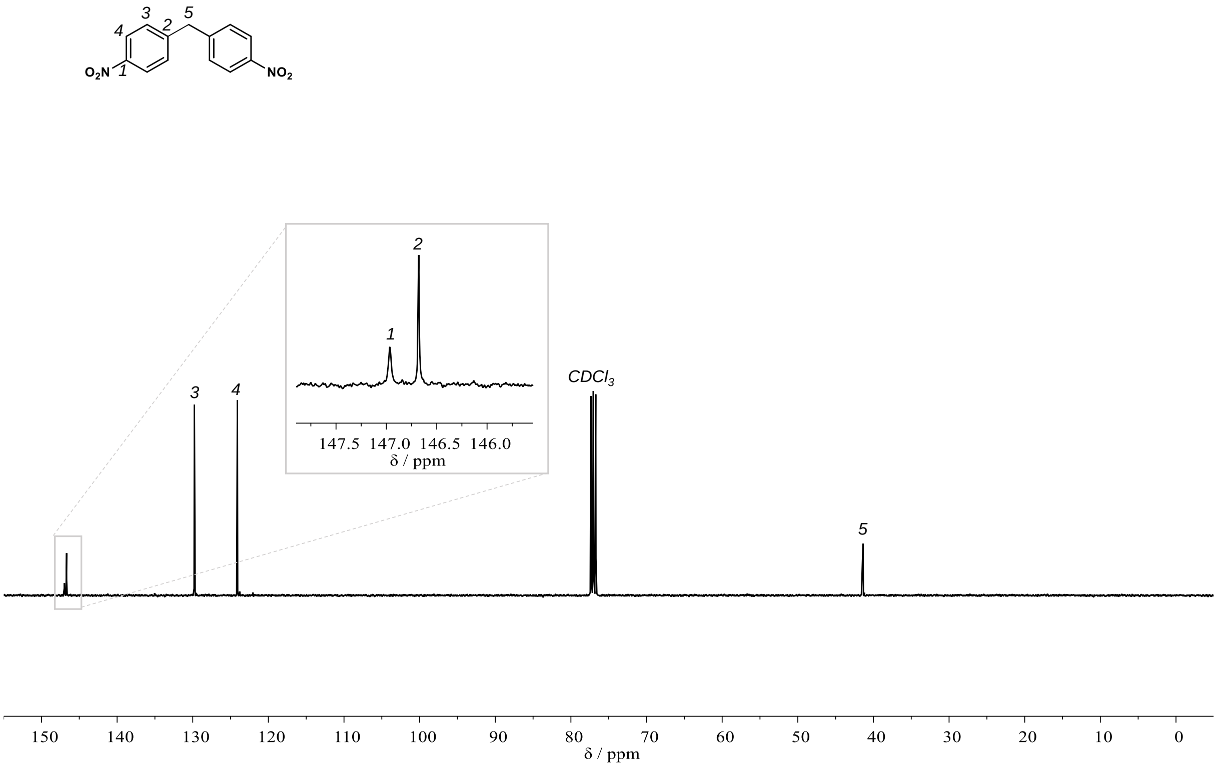

Supplementary Figure 4: ^{13}C-NMR spectrum of 8.

***Synthesis of 4,4'-dinitrobenzophenone* (3d)**

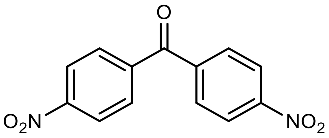

In a 500 mL Erlenmeyer flask, 2.58 g of 4,4'-dinitrodiphenylmethane (**8**) (10.0 mmol, 1.00 eq.) were dissolved in 50 mL of DCM. Afterwards, a solution of 2.37 g potassium permanganate (15.0 mmol, 1.50 eq.), 340 mg of tetrabutylammonium hydrogen sulfate (1.00 mmol, 0.10 mmol) and 281 mg of potassium hydroxide (5.00 mmol, 0.50 eq.) in 50 mL of water was added. The mixture was stirred vigorously at room temperature for 5 hours. Subsequently, 5 mL of acetic acid were added and concentrated sodium sulfite solution was added until the disappearance of the brown manganese dioxide color. The organic phase was separated, dried over sodium sulfate and the solvent was removed under reduced pressure. The product was isolated as a white solid in a yield of 22% (596 mg ,2.19 mmol). The procedure was adapted from Supplementary Reference [3].

^{1}H NMR (400 MHz, DMSO-d_6) δ/ppm = 8.44 – 8.36 (m, 1H), 8.07 – 7.97 (m, 1H).

^{13}C NMR (101 MHz, DMSO-d_6) δ/ppm = 193.89, 150.33, 141.73, 131.57, 124.28.

IR (ATR platinum diamond): ν/cm^{-1} = 3106 (vw), 3083 (vw), 2853 (vw), 1656 (m), 1600 (m), 1514 (vs), 1407 (w), 1343 (vs), 1312 (s), 1298 (s), 1271 (vs), 1172 (w), 1144 (w), 1107 (w), 1010 (w), 979 (w), 934 (s), 870 (m), 862 (m), 845 (vs), 788 (w), 738 (w), 708 (vs), 695 (vs), 654 (m), 621 (w), 494 (w), 432 (w).

EI-HRMS m/z: $[M]^+$ calculated for $C_{13}H_8N_2O_5$ = 272.0428, found 272.0426.

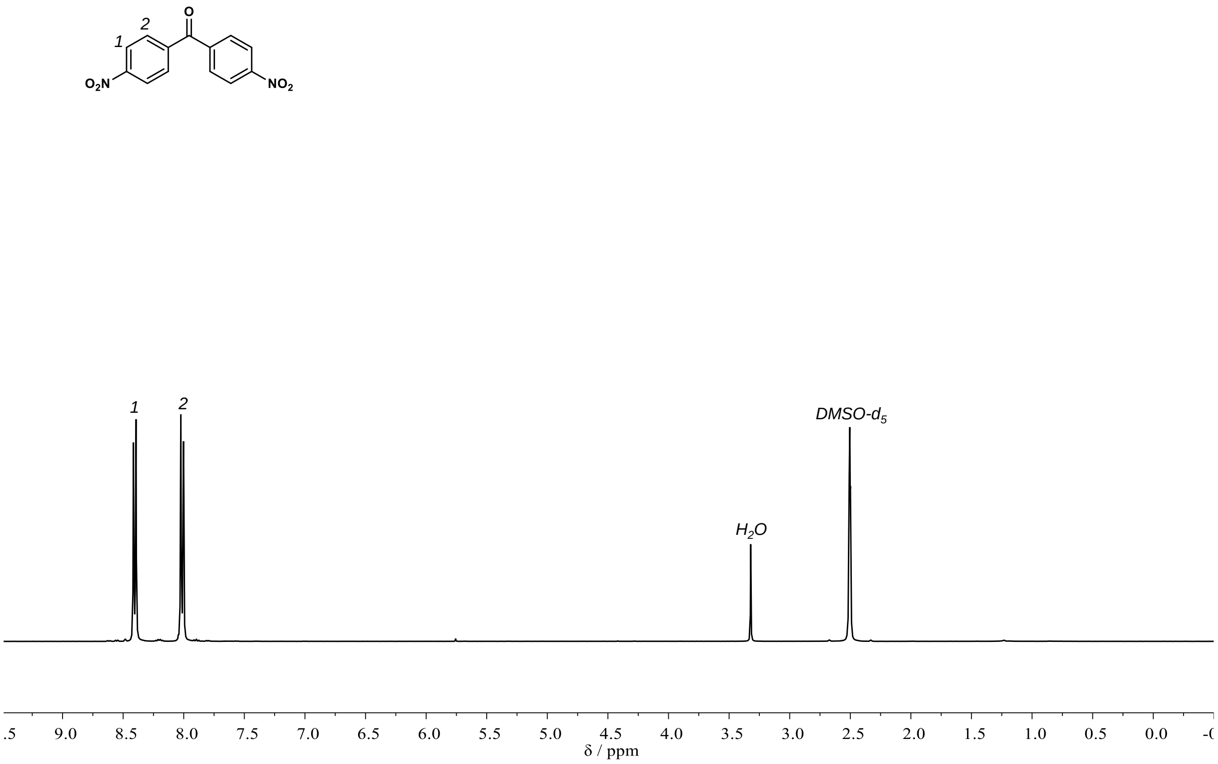

Supplementary Figure 5: ^{1}H-NMR spectrum of 3d.

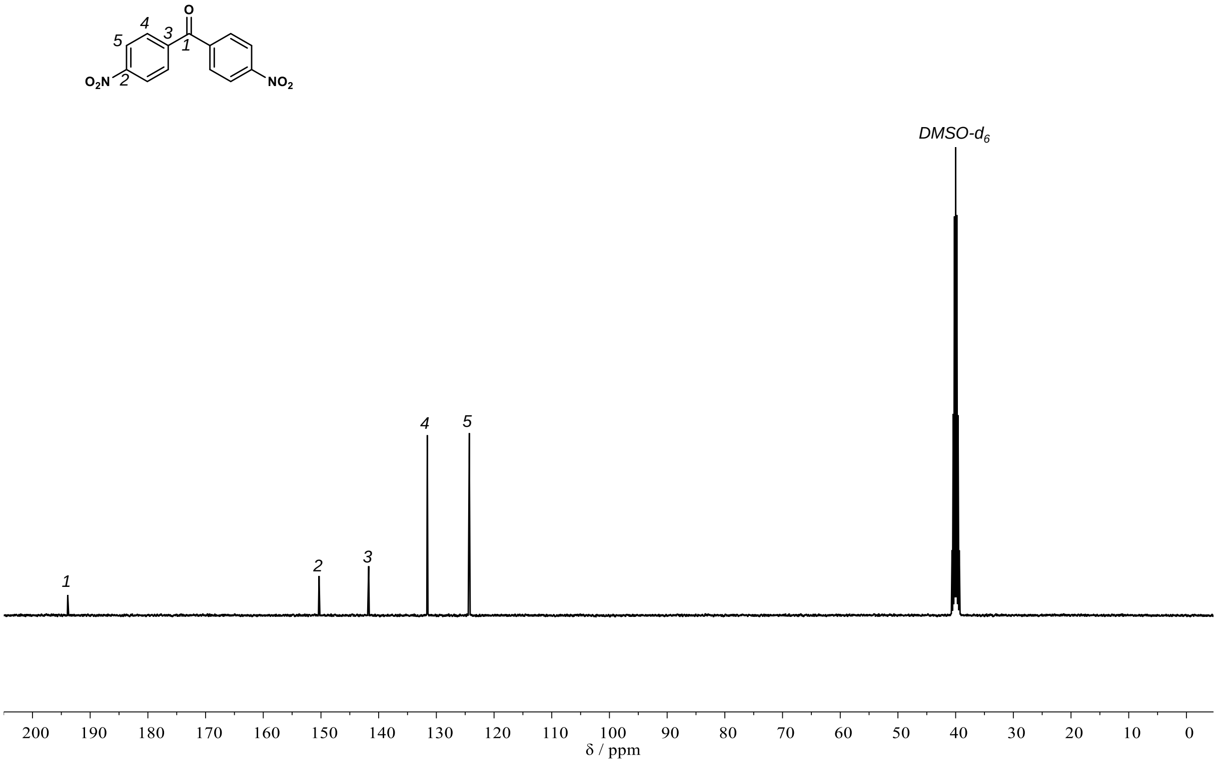

Supplementary Figure 6: ^{13}C-NMR spectrum of 3d.

***Synthesis of 4-methoxybenzophenone* (3f)**

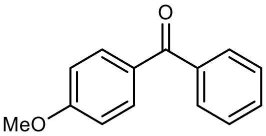

1.98 g (10.0 mmol, 1.05 eq.) of 4-hydroxybenzophenone were dissolved in 100 mL of MeCN and 5 mL of a 2 M aqueous potassium hydroxide solution (corresponding to 561 mg, 10.0 mmol, 1.00 eq. KOH) were added. The yellow solution was stirred at room temperature for 15 minutes. Afterwards, the solvent was removed under reduced pressure and the yellow residue was redissolved in 125 mL of MeCN. 591 µL of methyl iodide (1.35 g, 9.50 mmol, 1.00 eq.) were added and the mixture was refluxed for 2 hours. Subsequently, the solvent was removed under reduced pressure and 100 mL of DCM followed by 100 mL of 1 M aqueous NaOH solution were added to the residue. The organic phase was separated and washed once more with 1 M NaOH and twice with water, then dried over sodium sulfate. The solvent was removed *in vacuo* to yield the product as a white solid in a yield of 84% (1.69 g, 7.98 mmol). Procedure was adapted and modified from Supplementary Reference [4].

^{1}H NMR (400 MHz, DMSO-d_6) δ/ppm = 7.79 – 7.71 (m, 2H), 7.75 – 7.65 (m, 2H), 7.69 – 7.60 (m, 1H), 7.59 – 7.50 (m, 2H), 7.13 – 7.05 (m, 2H), 3.86 (s, 3H).

^{13}C NMR (101 MHz, DMSO-d_6) δ/ppm = 194.43, 162.97, 137.77, 132.17, 132.08, 129.36, 129.24, 128.44, 113.91, 55.57.

IR (ATR platinum diamond): ν/cm^{-1} = 3059 (vw), 3013 (vw), 2970 (vw), 2845 (vw), 1648 (s), 1596 (vs), 1576 (m), 1506 (w), 1456 (w), 1444 (m), 1316 (s), 1279 (s), 1259 (vs), 1181 (w), 1168 (s), 1150 (s), 1113 (m), 1090 (w), 1074 (w), 1022 (s), 1000 (w), 936 (s), 917 (m), 841 (vs), 792 (s), 738 (vs), 695 (vs), 677 (s), 631 (w), 599 (vs), 564 (w), 512 (w).

ESI-HRMS m/z: $[M+H]^+$ calculated for $C_{14}H_{12}O_2$ = 213.0910, found 213.0906.

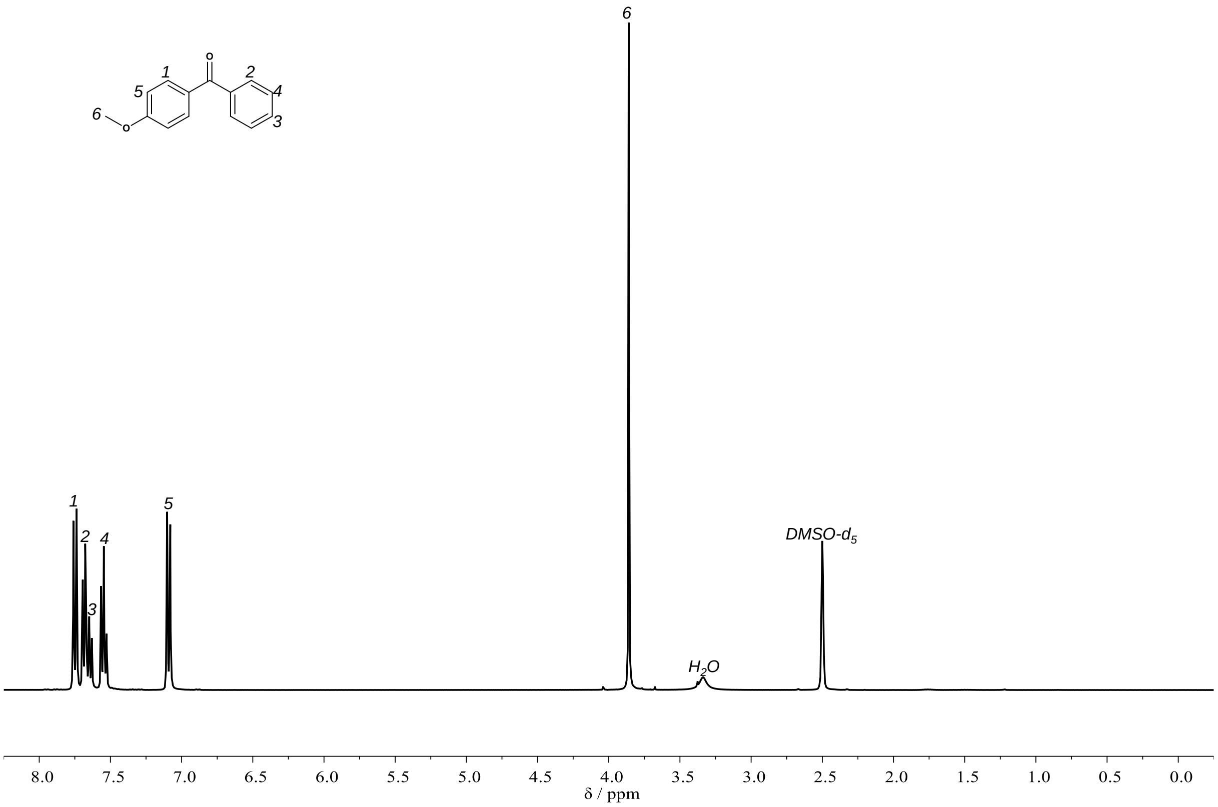

Supplementary Figure 7: ^{1}H-NMR spectrum of 3f.

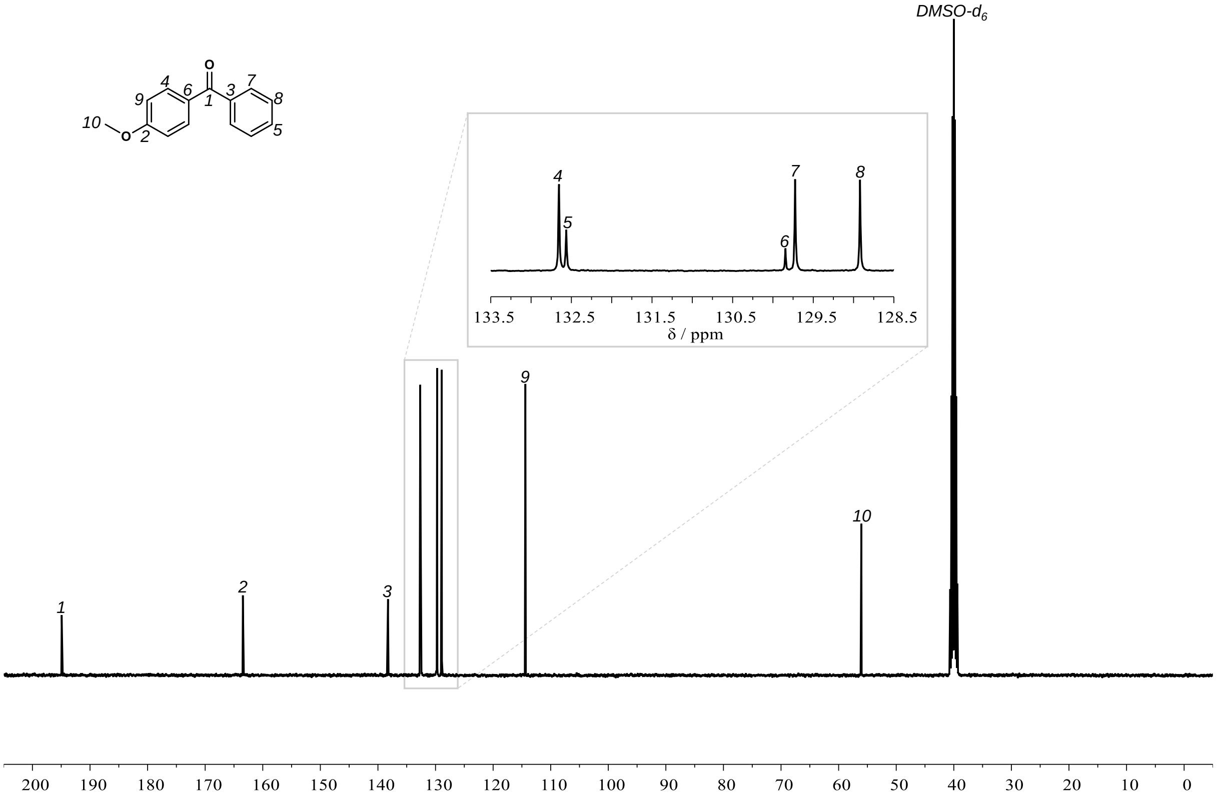

Supplementary Figure 8: ^{13}C-NMR spectrum of 3f.

***Synthesis of 4-methoxybenzophenone* (3j)**

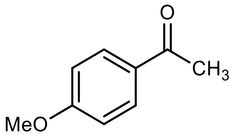

In a three-necked round bottom flask under argon atmosphere, 26.7 g of aluminium chloride (200 mmol, 1.20 eq.) were suspended in 66 mL of DCM and 12.4 mL of acetyl chloride (13.7 g, 175 mmol, 1.05 eq.) was added dropwise at 0 °C. Subsequently, 18.2 mL of anisole (18.1 g, 167 mmol, 1.00 eq.) was added dropwise while maintaining a temperature below 20°C. After stirring at room temperature for 2 hours, the mixture was left to stand overnight at room temperature. Afterwards, the crude mixture was poured onto 100 g of ice and 50 mL of DCM along with 300 mL of concentrated hydrochloric acid were added. The phases were separated and the aqueous phase was extracted twice more with dichloromethane. The combined extracts were washed once each with 1 M sodium hydroxide solution and water, then dried over sodium sulfate. The solvent was removed under reduced pressure and the crude product was purified *via* vacuum distillation at 20 mbar (bp = 139 °C). The product was obtained as a white solid in a yield of 68% (17.0 g, 113 mmol).

^{1}H NMR (400 MHz, Chloroform-*d*) δ/ppm = 7.96 – 7.89 (m, 2H), 6.96 – 6.88 (m, 2H), 3.85 (s, 3H), 2.54 (d, *J* = 0.7 Hz, 3H).

^{13}C NMR (101 MHz, Chloroform-*d*) δ/ppm = 196.85, 163.57, 130.67, 130.42, 113.76, 55.54, 26.42.

IR (ATR platinum diamond): ν/cm^{-1} = 2999 (vw), 2962 (vw), 2937 (vw), 2845 (vw), 1668 (vs), 1643 (w), 1598 (vs), 1574 (s), 1508 (m), 1460 (w), 1446 (w), 1417 (m), 1356 (s), 1304 (w), 1273 (s), 1246 (vs), 1170 (vs), 1113 (m), 1074 (w), 1020 (vs), 954 (m), 913 (w), 831 (vs), 819 (s), 806 (s), 759 (w), 738 (vw), 629 (vw), 590 (s), 576 (s), 564 (s), 522 (vw), 498 (w), 461 (w).

ESI-HRMS m/z: $[M+H]^+$ calculated for $C_9H_{10}O_2$ = 151.0754, found 151.0747.

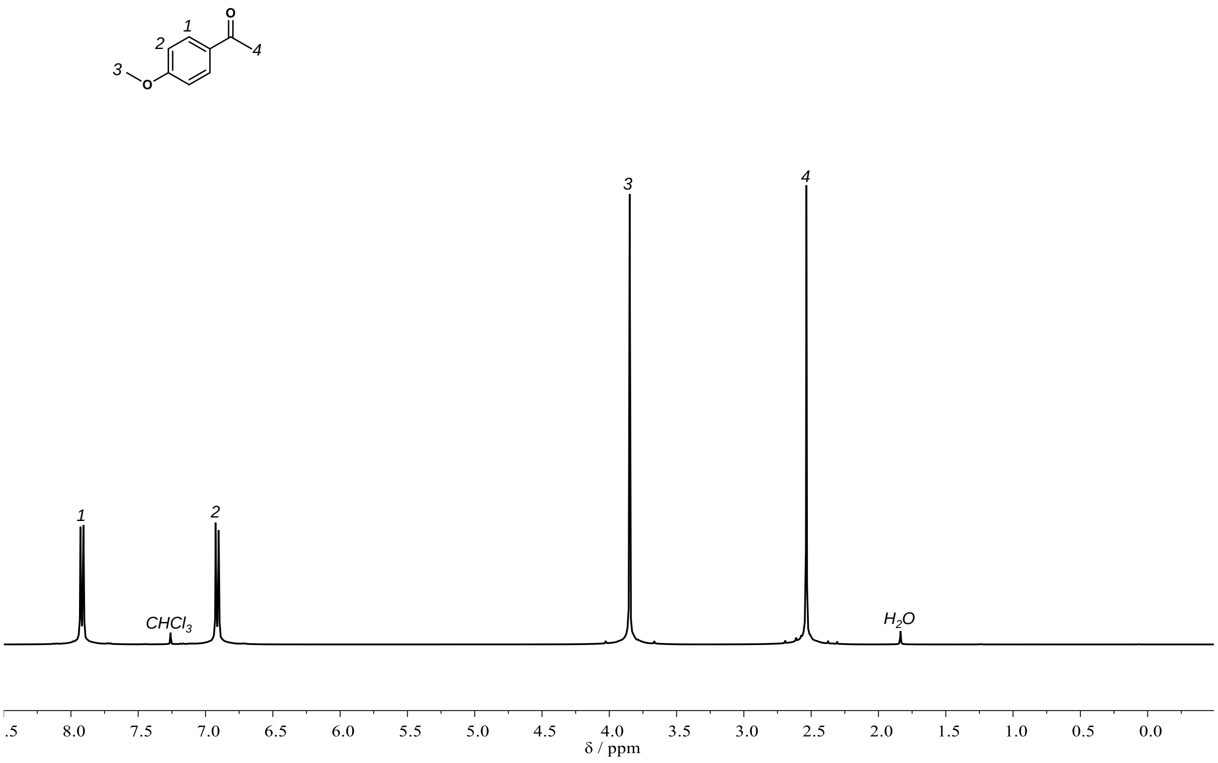

Supplementary Figure 9: ^{1}H-NMR spectrum of 3j.

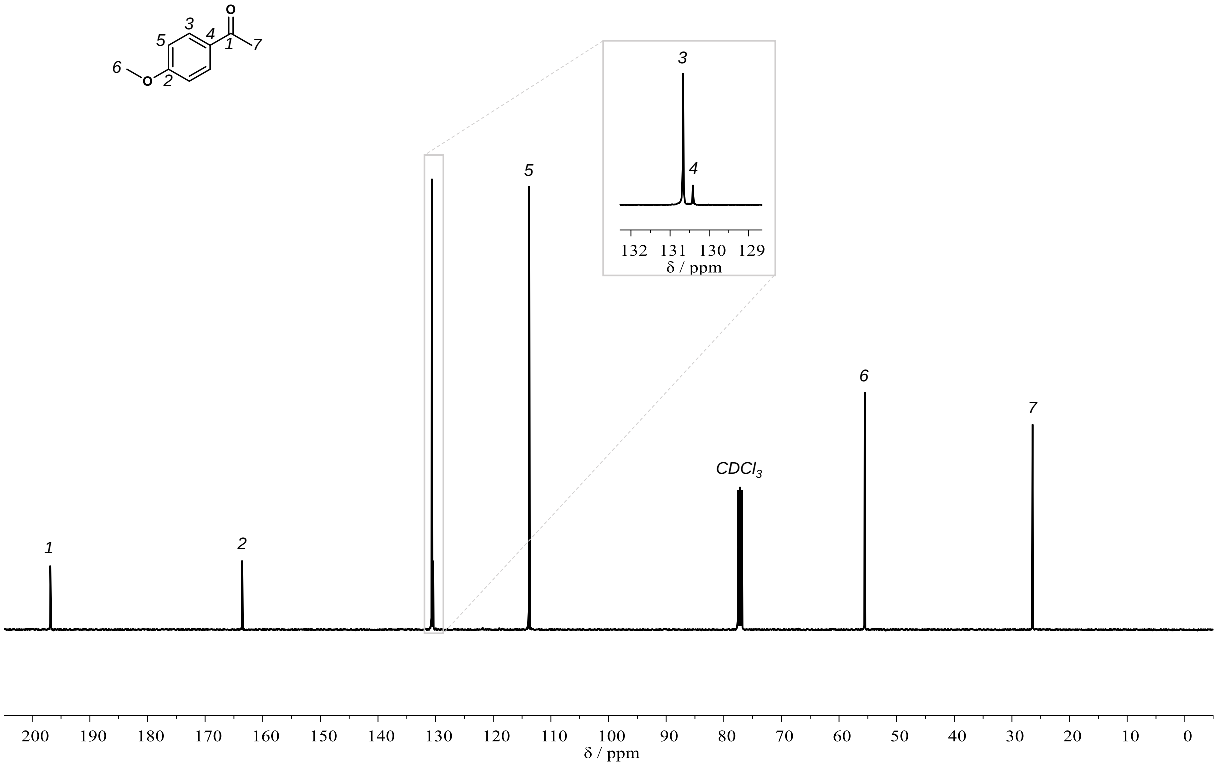

Supplementary Figure 10: ^{13}C-NMR spectrum of 3j.

1.3.2 *N*-Tosylhydrazones

General procedures for the synthesis of N-tosylhydrazones from carbonyl compounds

Procedure A:
931 mg tosyl hydrazide (5.00 mmol, 1.00 eq.) were suspended in 10 mL of ethanol and 5.00 mmol (1.00 eq.) of the carbonyl component along with 47.5 mg of *p*-toluenesulfonic acid monohydrate (0.25 mmol, 0.05 eq.) were added. The mixture was refluxed overnight. Afterwards, the mixture was cooled down to -20 °C, the precipitate was filtered off and washed with cold ethanol. The filtered solid was dried *in vacuo*.

Procedure B:
931 mg tosyl hydrazide (5.00 mmol, 1.00 eq.) were suspended in 10 mL of ethanol and 5.00 mmol (1.00 eq.) of the carbonyl component were dissolved in 10 mL of ethanol. The mixture was stirred at room temperature for 2 hours. Afterwards, the solvent was removed *in vacuo*.

Synthesis of N'-(4,4'-dimethoxybenzhydrylidene)-p-toluenesulfonylhydrazide (*1a*)

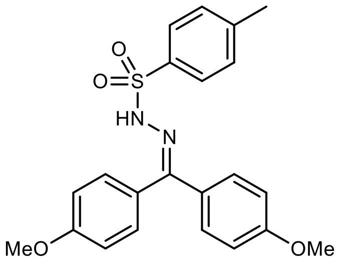

Prepared from **3a** according to general procedure procedure A. Obtained as a white, crystalline solid in a yield of 81% (1.66 g, 4.04 mmol).

^{1}H NMR (400 MHz, DMSO-*d*6) δ/ppm = 10.19 (s, 1H), 7.83 – 7.76 (m, 2H), 7.45 – 7.39 (m, 2H), 7.23 – 7.11 (m, 4H), 7.10 – 7.02 (m, 2H), 6.93 – 6.85 (m, 2H), 3.83 (s, 3H), 3.74 (s, 3H), 2.39 (s, 3H).

^{13}C NMR (101 MHz, DMSO-*d*6) δ/ppm = 160.46, 159.84, 154.47, 143.18, 136.13, 130.48, 130.00, 129.35, 128.91, 127.77, 124.77, 114.10, 113.69, 55.23, 21.05.

IR (ATR platinum diamond): v/cm^{-1} = 3201 (w), 3065 (vw), 3028 (vw), 2956 (vw), 2925 (vw), 2832 (vw), 1606 (s), 1578 (w), 1510 (s), 1460 (w), 1440 (w), 1419 (w), 1386 (m), 1341 (m), 1310 (s), 1300 (m), 1249 (vs), 1187 (w), 1174 (s), 1158 (vs), 1113 (w), 1092 (w), 1053 (m), 1031 (vs), 1012 (m), 983 (s), 954 (w), 887 (m), 831 (vs), 810 (s), 782 (w), 734 (w), 706 (m), 689 (m), 664 (vs), 597 (w), 578 (w), 545 (vs), 518 (m), 498 (m), 481 (w), 444 (w).

ESI-HRMS m/z: $[M+H]^+$ calculated for $C_{22}H_{22}N_2O_4S$ = 411.1373, found 411.1370.

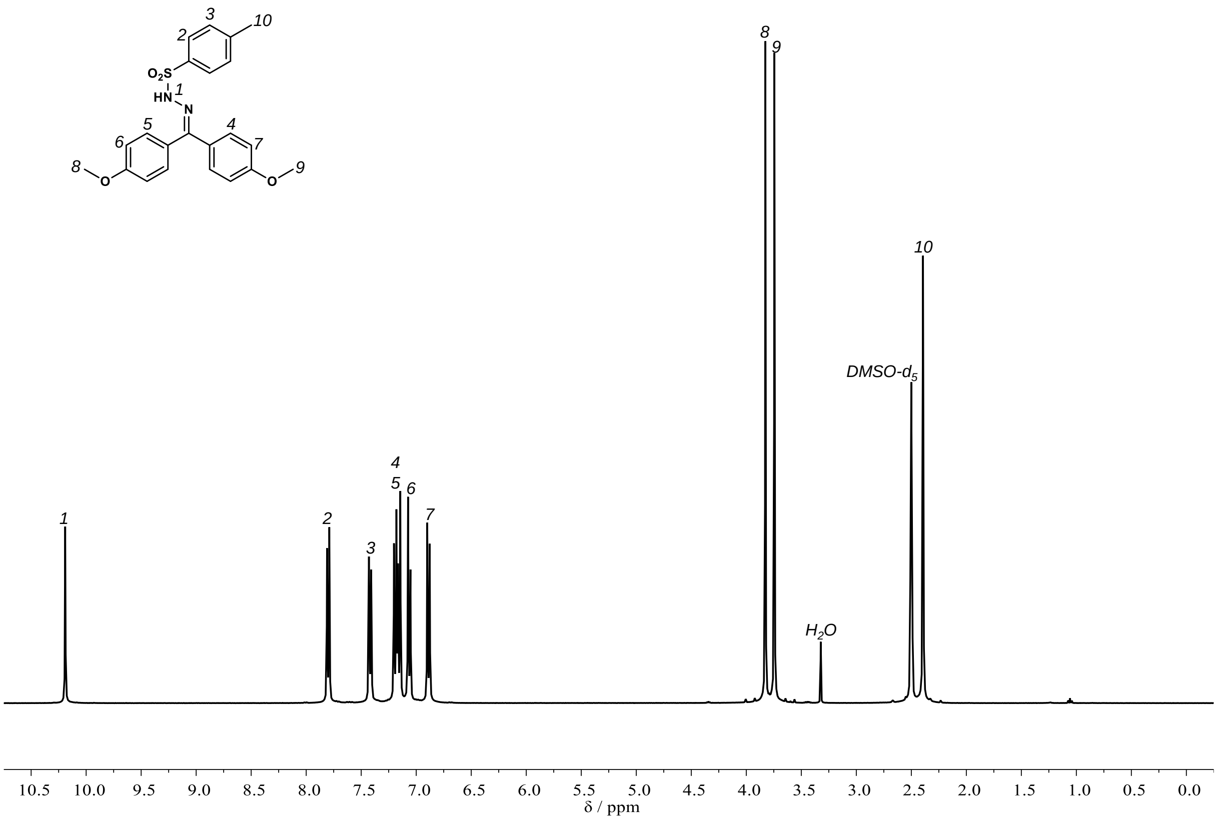

Supplementary Figure 11: ^{1}H-NMR spectrum of 1a.

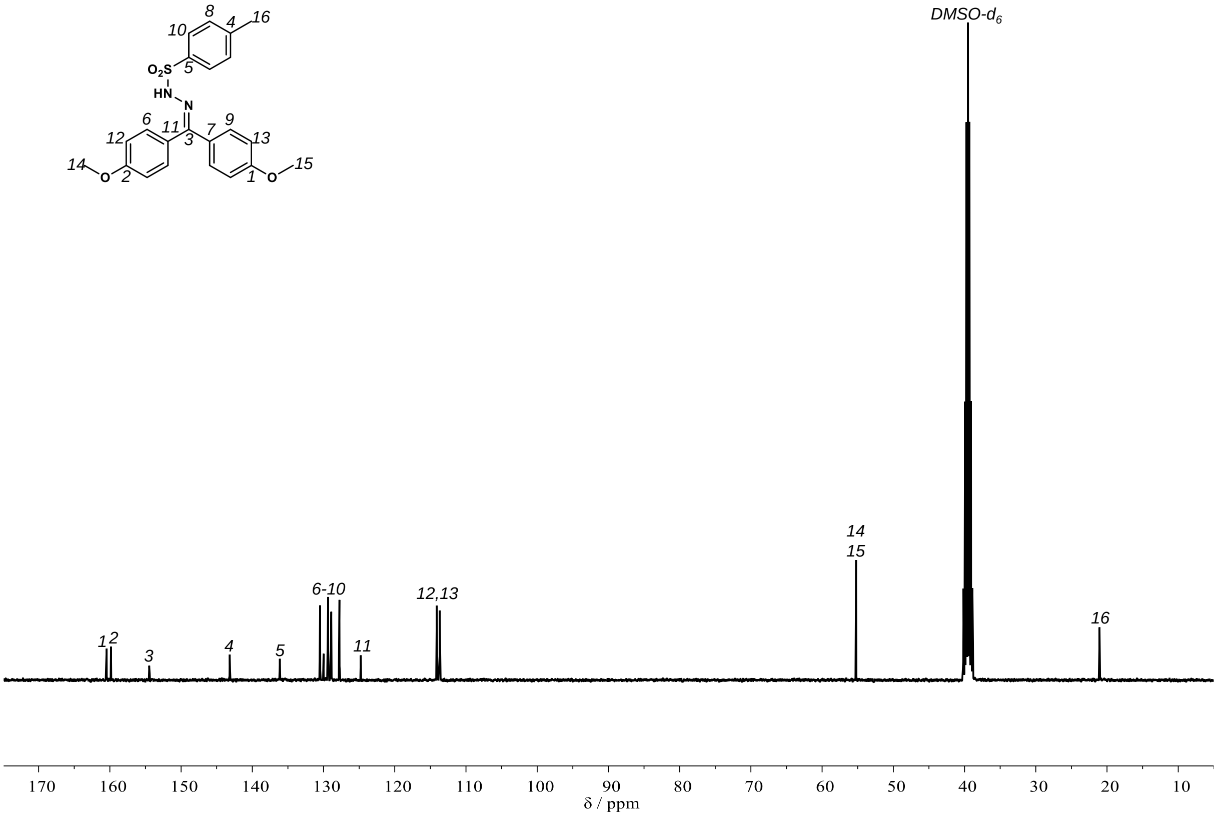

Supplementary Figure 12: ^{13}C-NMR spectrum of 1a.

Synthesis of N'-benzhydrylidene-p-toluenesulfonylhydrazide (*1b*)

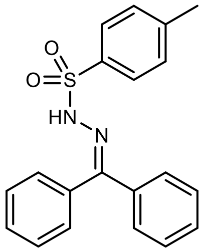

Prepared from **3b** according to general procedure A. Obtained as a white, crystalline solid in a yield of 81% (1,43 g, 4.07 mmol).

^{1}H NMR (400 MHz, DMSO-d_6) δ/ppm = 10.43 (s, 1H), 7.85 – 7.78 (m, 2H), 7.58 – 7.47 (m, 3H), 7.47 – 7.40 (m, 2H), 7.42 – 7.29 (m, 3H), 7.29 – 7.17 (m, 4H), 2.40 (s, 3H).

^{13}C NMR (101 MHz, DMSO-d_6) δ/ppm = 154.54, 143.34, 137.16, 136.07, 132.56, 129.69, 129.43, 129.37, 128.81, 128.35, 127.75, 127.22, 21.05.

IR (ATR platinum diamond): v/cm^{-1} = 3211 (w), 3061 (vw), 3028 (vw), 1594 (vw), 1571 (vw), 1446 (w), 1374 (m), 1347 (s), 1318 (m), 1302 (w), 1187 (w), 1168 (vs), 1094 (w), 1053 (m), 1026 (w), 1002 (w), 977 (m), 915 (w), 872 (m), 812 (m), 771 (vs), 732 (w), 697 (vs), 664 (vs), 640 (m), 609 (w), 553 (vs), 541 (vs), 508 (s), 455 (m).

ESI-HRMS m/z: $[M+H]^+$ calculated for $C_{20}H_{18}N_2O_2S$ = 351.1162, found 351.1157.

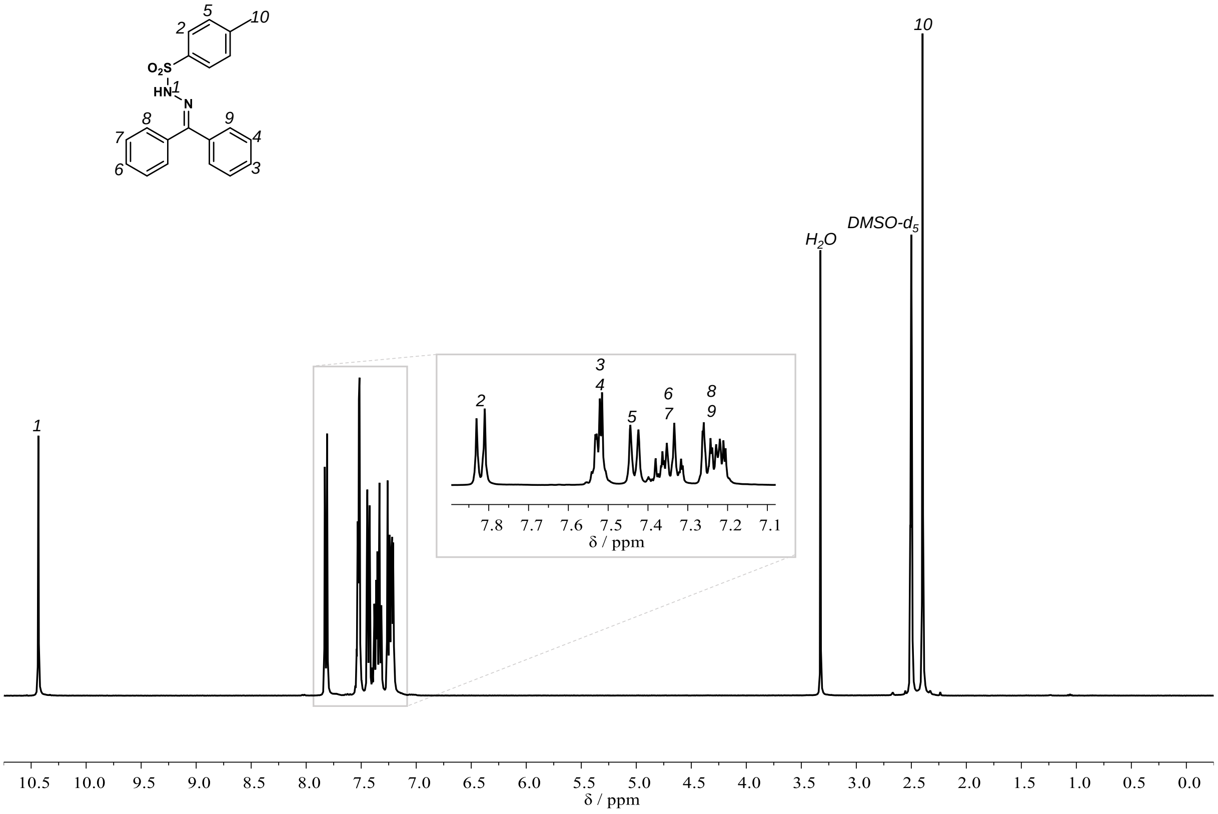

Supplementary Figure 13: ^{1}H-NMR spectrum of 1b.

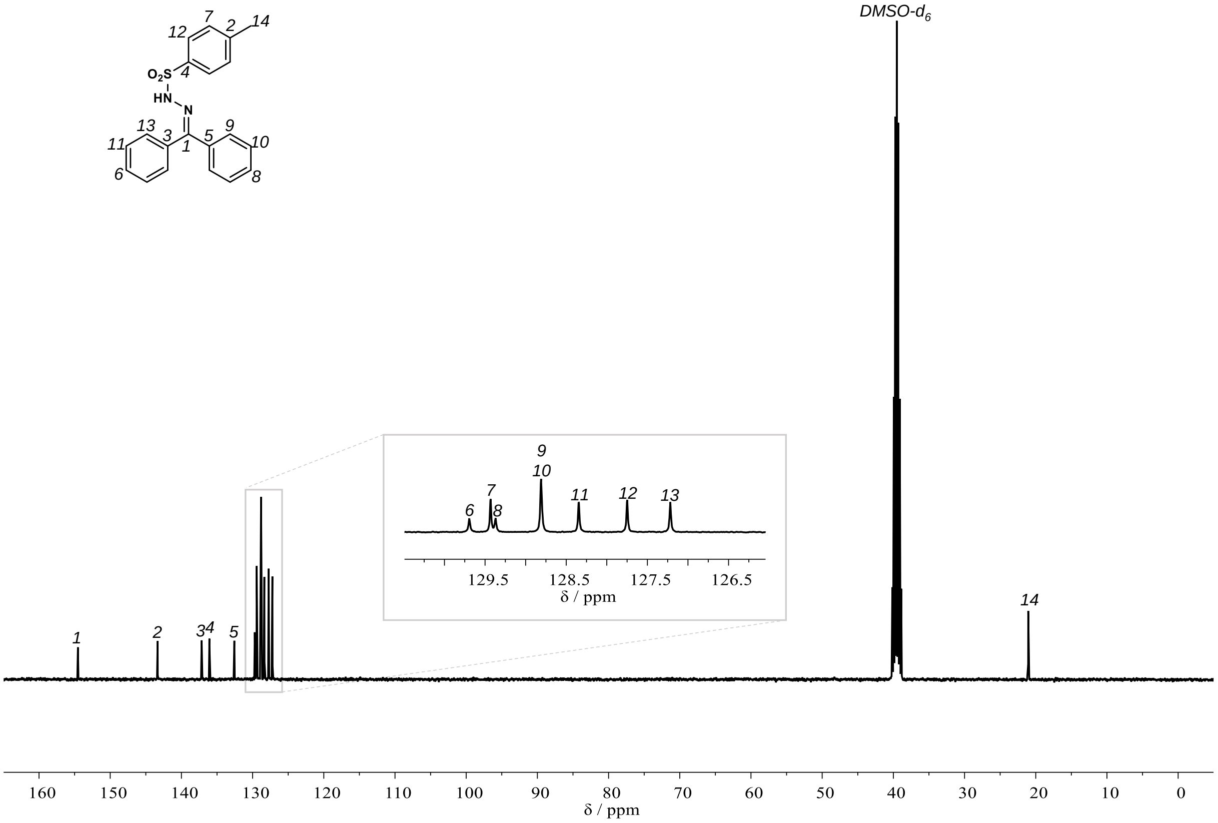

Supplementary Figure 14: ^{13}C-NMR spectrum of 1b.

Synthesis of N'-(4,4'-dibromobenzhydrylidene)-p-toluenesulfonylhydrazide (1c)

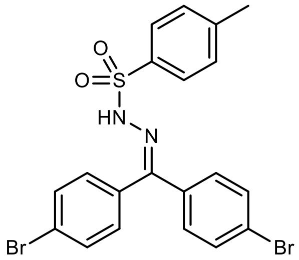

Prepared from **3c** according to general procedure A in a smaller scale scale of 2 mmol. Obtained as a light orange, crystalline solid in a yield of 82% (836 mg, 1.62 mmol).

^{1}H NMR (400 MHz, DMSO-d_6) δ/ppm = 10.68 (s, 1H), 7.82 – 7.76 (m, 2H), 7.76 – 7.69 (m, 2H), 7.59 – 7.51 (m, 2H), 7.46 – 7.39 (m, 2H), 7.23 – 7.12 (m, 4H), 2.39 (s, 3H).

^{13}C NMR (101 MHz, DMSO-d_6) δ/ppm = 152.29, 143.95, 136.49, 136.39, 132.48, 131.95, 131.66, 131.56, 130.03, 129.49, 128.05, 123.79, 123.58, 21.54.

IR (ATR platinum diamond): *v*/cm^{-1} = 3184 (w), 3059 (vw), 1596 (w), 1485 (w), 1405 (m), 1395 (w), 1347 (s), 1316 (m), 1294 (w), 1189 (w), 1168 (vs), 1098 (w), 1068 (s), 1057 (s), 1008 (s), 987 (m), 948 (w), 884 (m), 835 (m), 825 (m), 812 (s), 755 (vw), 716 (w), 706 (w), 681 (s), 664 (m), 638 (m), 617 (w), 603 (w), 551 (vs), 510 (w), 492 (m), 465 (w).

ESI-HRMS m/z: $[M+H]^+$ calculated for $C_{20}H_{16}N_2O_2S^{79}Br^{81}Br$ = 508.9352, found 508.9349.

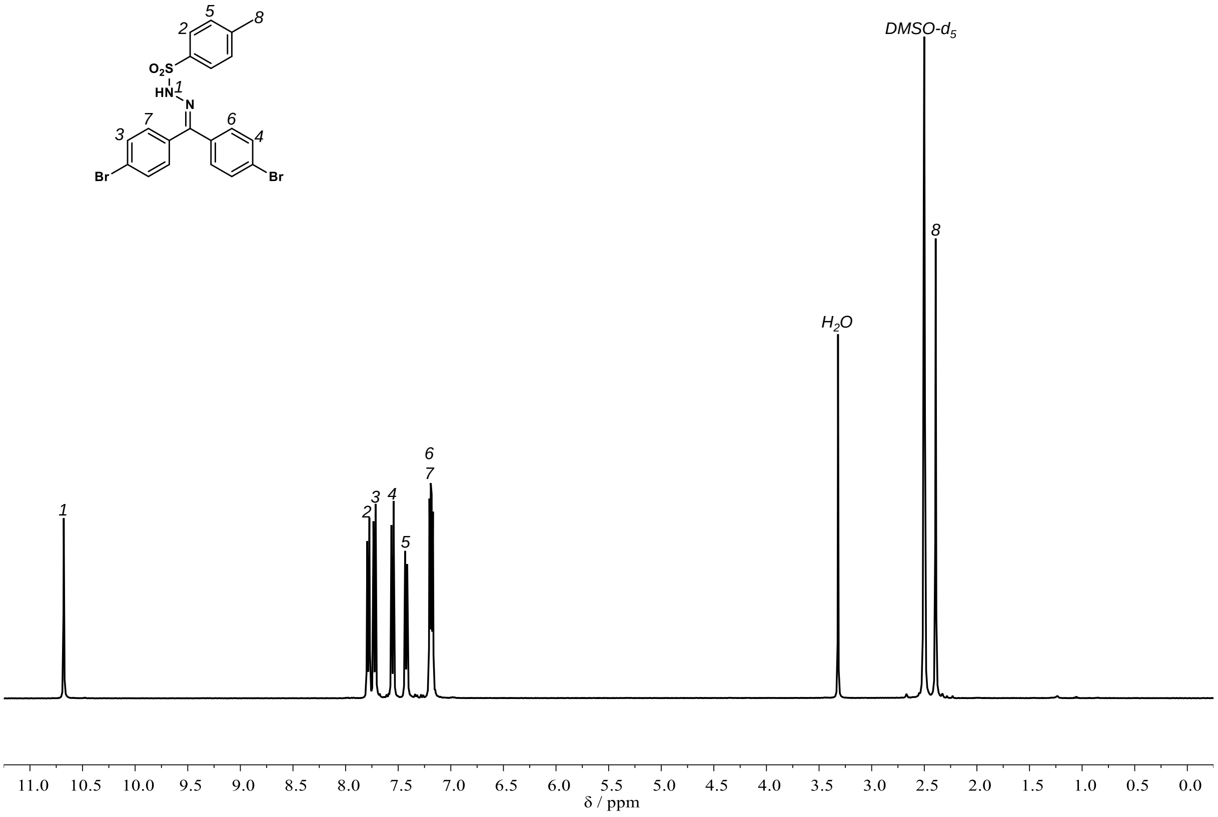

Supplementary Figure 15: ^{1}H-NMR spectrum of 1c.

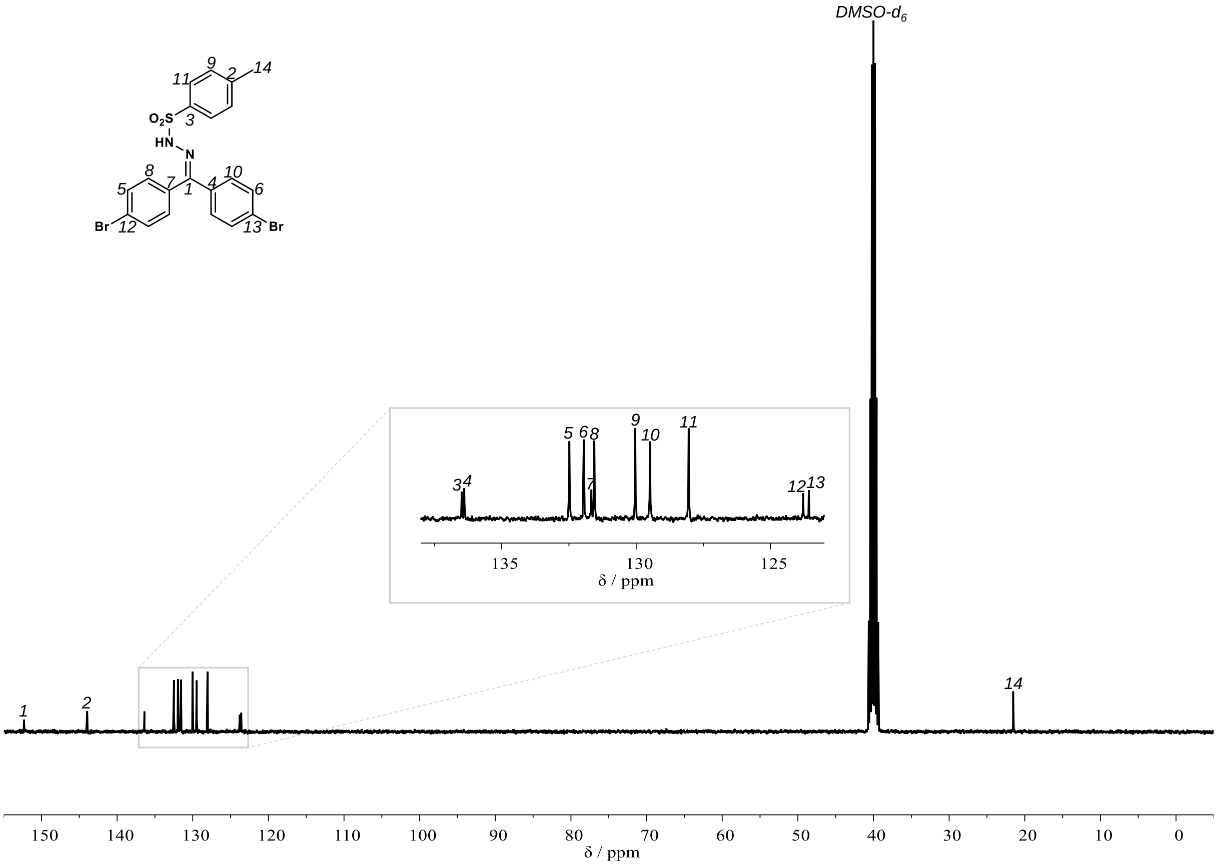

Supplementary Figure 16: ^{13}C-NMR spectrum of 1c.

***Synthesis of N'-(4,4'-dinitrobenzhydrylidene)-p-toluenesulfonylhydrazide* (1d)**

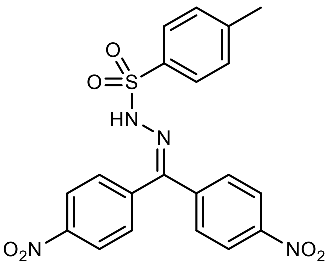

Prepared from **3d** according to general procedure A in a smaller scale of 2 mmol. The crude product was washed with hot ethanol. Obtained as an orange, crystalline solid in a yield of 75% (658 mg, 1.49 mmol).

^{1}H NMR (400 MHz, DMSO-*d*$_6$) δ/ppm = 11.09 (s, 1H), 8.46 – 8.33 (m, 2H), 8.30 – 8.16 (m, 2H), 7.81 (d, *J* = 8.2 Hz, 2H), 7.69 – 7.50 (m, 4H), 7.48 – 7.43 (m, 2H), 2.40 (s, 3H).

^{13}C NMR (101 MHz, DMSO-*d*$_6$) δ/ppm = 149.33, 148.28, 147.87, 143.80, 142.05, 138.28, 135.83, 130.78, 129.76, 128.04, 127.52, 124.34, 123.82, 21.08.

IR (ATR platinum diamond): v/cm^{-1} = 3190 (w), 1596 (w), 1567 (w), 1512 (vs), 1489 (w), 1403 (w), 1345 (vs), 1312 (s), 1286 (w), 1187 (w), 1166 (vs), 1105 (w), 1092 (w), 1057 (m), 1016 (w), 983 (m), 961 (w), 882 (w), 860 (s), 843 (s), 810 (m), 767 (w), 757 (w), 745 (w), 693 (m), 679 (s), 642 (w), 619 (m), 605 (w), 547 (vs), 520 (w), 490 (w), 426 (w) cm^{-1}.

FAB-HRMS m/z: $[M+H]^+$ calculated for $C_{20}H_{16}N_4O_6S$ = 441.0863, found 441.0863.

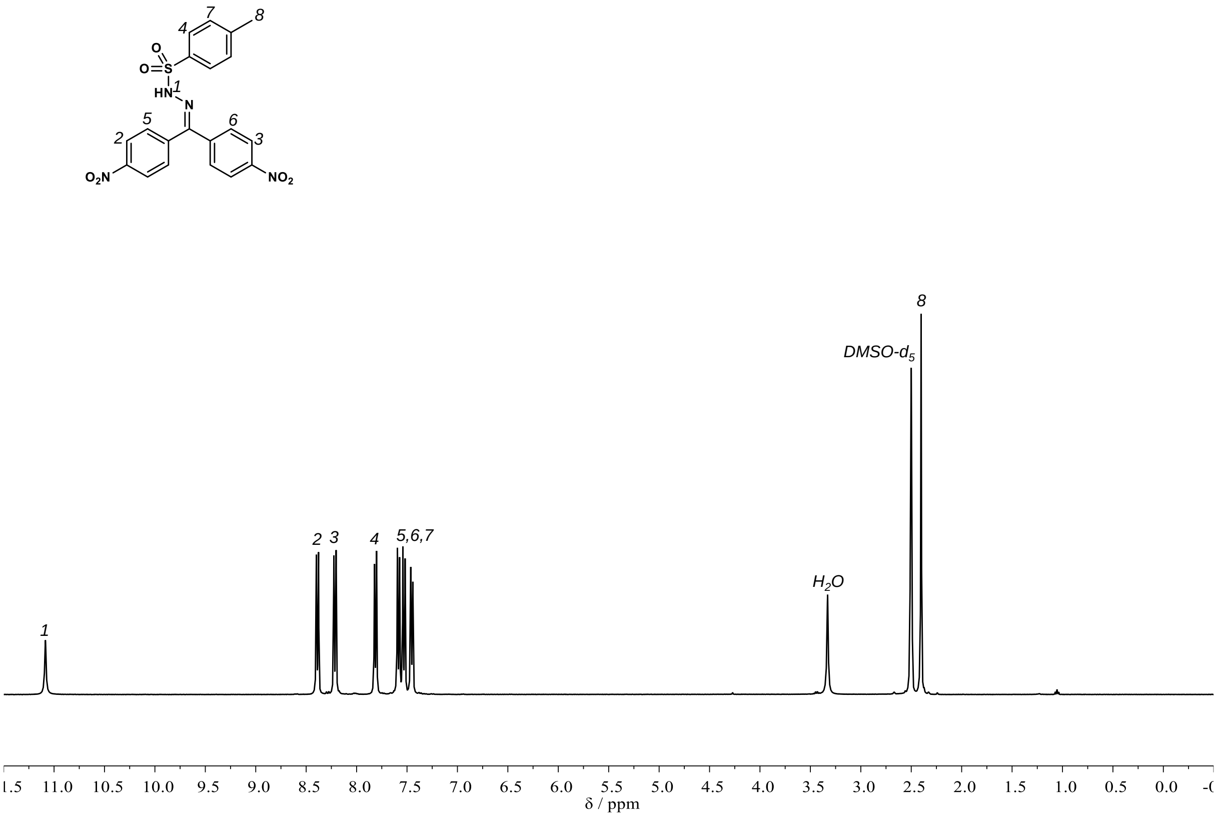

Supplementary Figure 17: ^{1}H-NMR spectrum of 1d.

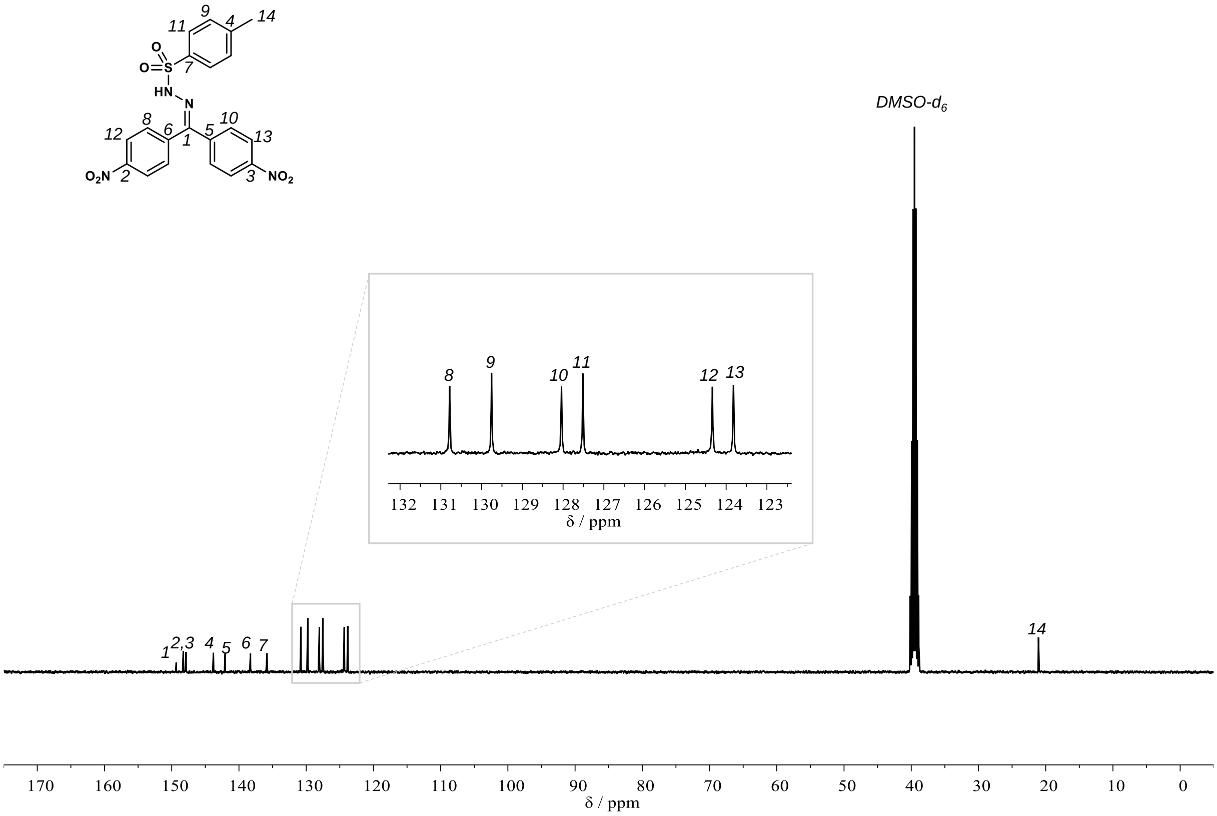

Supplementary Figure 18: ^{13}C-NMR spectrum of 1d.

***Synthesis of N'-(9-fluorenylidene)-p-toluenesulfonylhydrazide* (1e)**

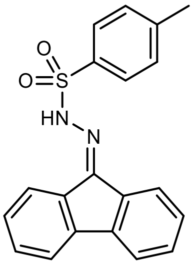

Prepared from **3e** according to general procedure A on a larger scale of 5.00 g (29.1 mmol). Obtained as a yellow, crystalline solid in a yield of 80% (7.86 g, 23.7 mmol).

^{1}H NMR (400 MHz, DMSO-d_6) δ/ppm = 11.39 (s, 1H), 8.14 – 8.08 (m, 1H), 7.97 – 7.91 (m, 2H), 7.90 – 7.83 (m, 1H), 7.83 – 7.76 (m, 1H), 7.62 – 7.55 (m, 1H), 7.58 – 7.50 (m, 1H), 7.49 – 7.39 (m, 4H), 7.36 – 7.28 (m, 1H), 2.39 (s, 3H).

^{13}C NMR (101 MHz, DMSO-d_6) δ/ppm = 152.08, 144.28, 141.95, 140.07, 136.28, 135.63, 132.33, 131.23, 129.88, 129.50, 128.82, 128.74, 128.64, 128.39, 121.90, 121.16, 120.91, 21.54.

IR (ATR platinum diamond): *v*/cm^{-1} = 3209 (w), 3061 (vw), 3028 (vw), 1596 (w), 1497 (vw), 1450 (m), 1382 (m), 1325 (s), 1310 (s), 1294 (m), 1216 (vw), 1195 (vw), 1150 (vs), 1092 (m), 1053 (w), 1026 (m), 1020 (m), 971 (s), 936 (w), 872 (m), 866 (m), 812 (s), 778 (vs), 722 (vs), 703 (w), 673 (vs), 656 (s), 642 (vs), 617 (m), 562 (w), 537 (vs), 500 (s), 479 (m), 428 (w).

ESI-HRMS m/z: [M+H]$^+$ calculated for $C_{20}H_{16}N_2O_2S$ = 349.1005, found 349.0998.

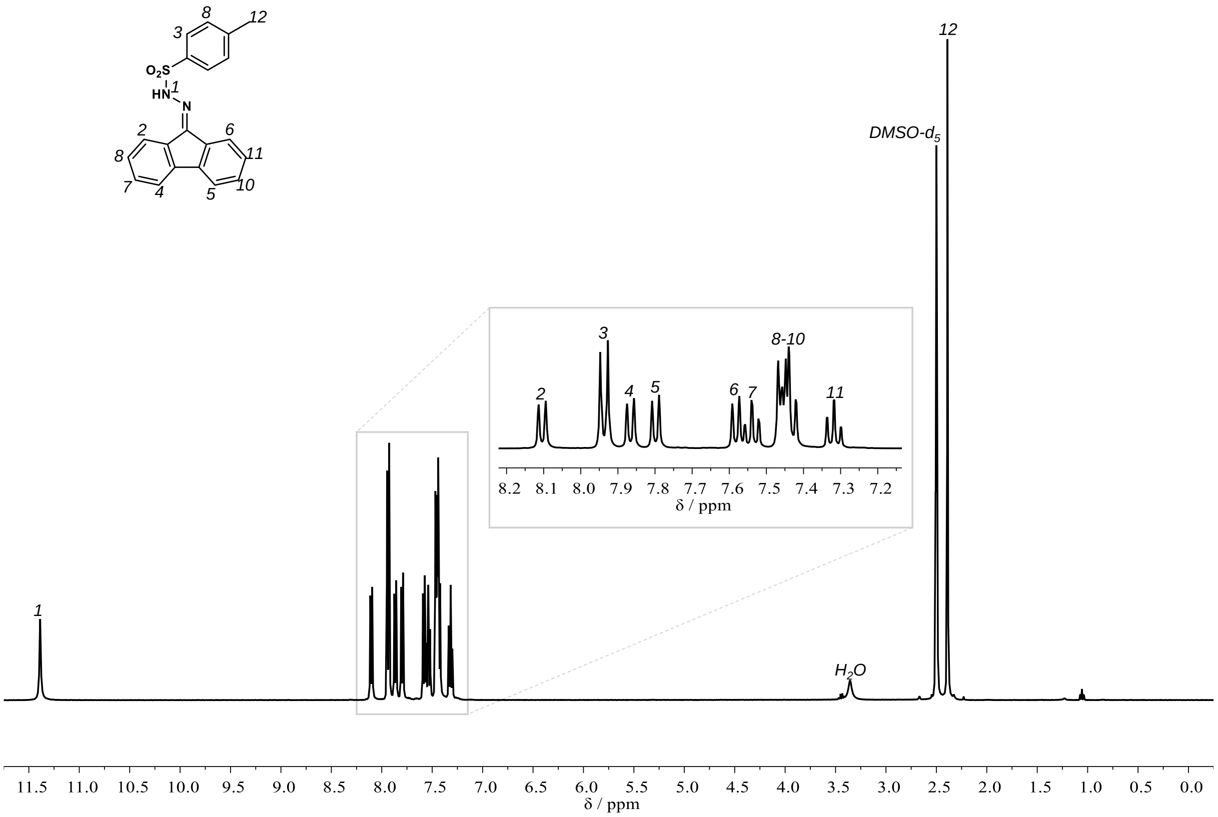

Supplementary Figure 19: ^{1}H-NMR spectrum of 1e.

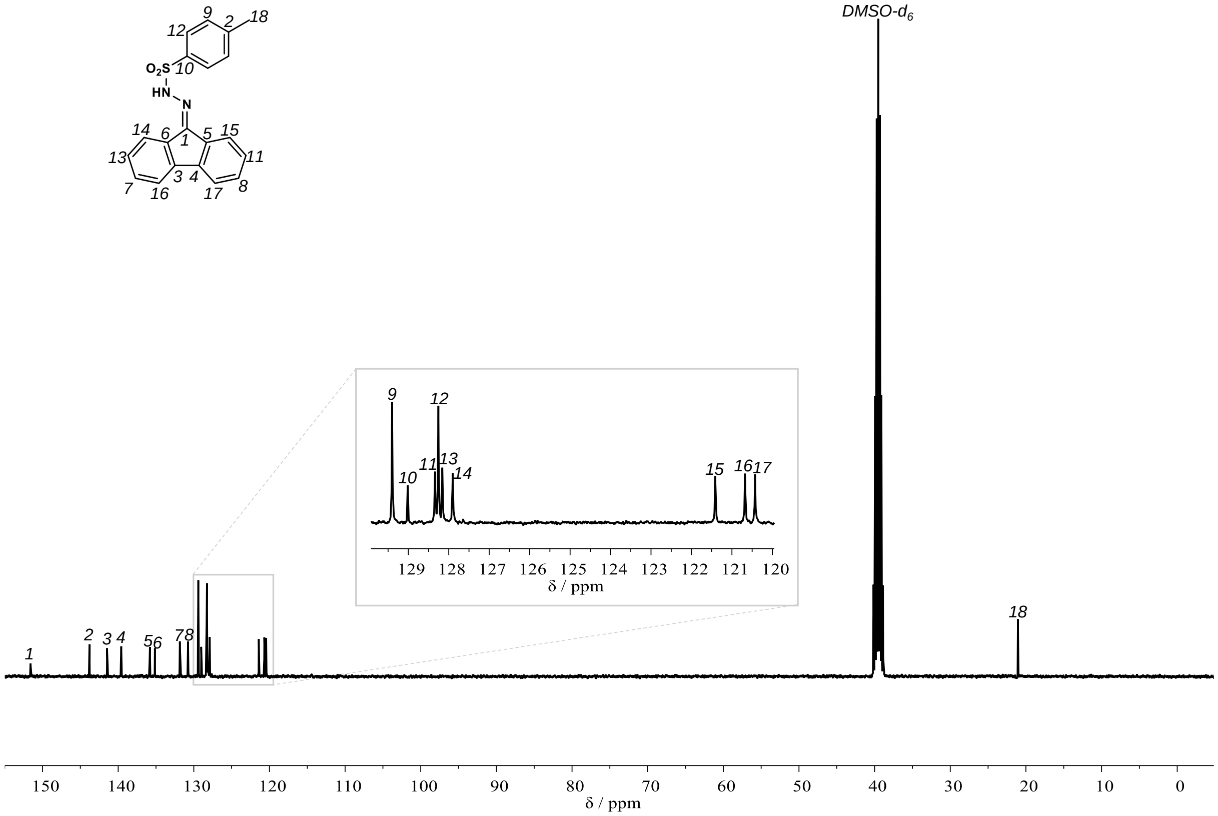

Supplementary Figure 20: ^{13}C-NMR spectrum of 1e.

***Synthesis of N'-(4-methoxybenzhydrylidene)-p-toluenesulfonylhydrazide* (1f)**

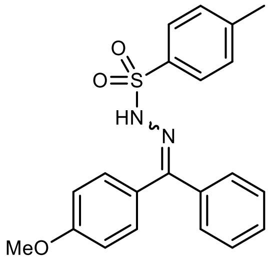

Prepared from **3f** according to general procedure A. Obtained as a white solid in a yield of 33% (629 mg, 1.66 mmol). The isolated product consisted of a mixture of the *(E)*- and *(Z)*-stereoisomers in a ratio of 91:9. In the ^{1}H-NMR spectrum, only the signals of the *(E)*-isomer are assigned.

^{1}H NMR (400 MHz, DMSO-d_6) δ/ppm = 10.22 (s, 1H), 7.85 – 7.77 (m, 2H), 7.57 – 7.46 (m, 3H), 7.46 – 7.39 (m, 2H), 7.25 – 7.13 (m, 4H), 6.93 – 6.85 (m, 2H), 3.74 (s, 3H), 2.40 (s, 3H).

^{13}C NMR (101 MHz, DMSO-d_6) δ/ppm = 160.52, 154.76, 143.24, 136.10, 132.80, 129.62, 129.38, 129.26, 128.80, 128.76, 128.73, 127.78, 113.77, 55.25, 21.05.

IR (ATR platinum diamond): v/cm^{-1} = 3277 (vw), 3201 (w), 3052 (vw), 3026 (vw), 2972 (vw), 1609 (w), 1592 (w), 1510 (m), 1456 (w), 1444 (w), 1390 (w), 1378 (s), 1345 (w), 1314 (s), 1300 (m), 1259 (s), 1185 (w), 1158 (vs), 1117 (w), 1092 (w), 1078 (w), 1051 (m), 1024 (s), 1000 (w), 973 (s), 928 (w), 872 (w), 833 (s), 812 (m), 778 (vs), 716 (m), 703 (vs), 660 (vs), 638 (w), 607 (w), 599 (w), 555 (vs), 543 (vs), 520 (m), 502 (w), 465 (w).

ESI-HRMS m/z: $[M+H]^+$ calculated for $C_{21}H_{20}N_2O_3S$ = 381.1267, found 381.1261.

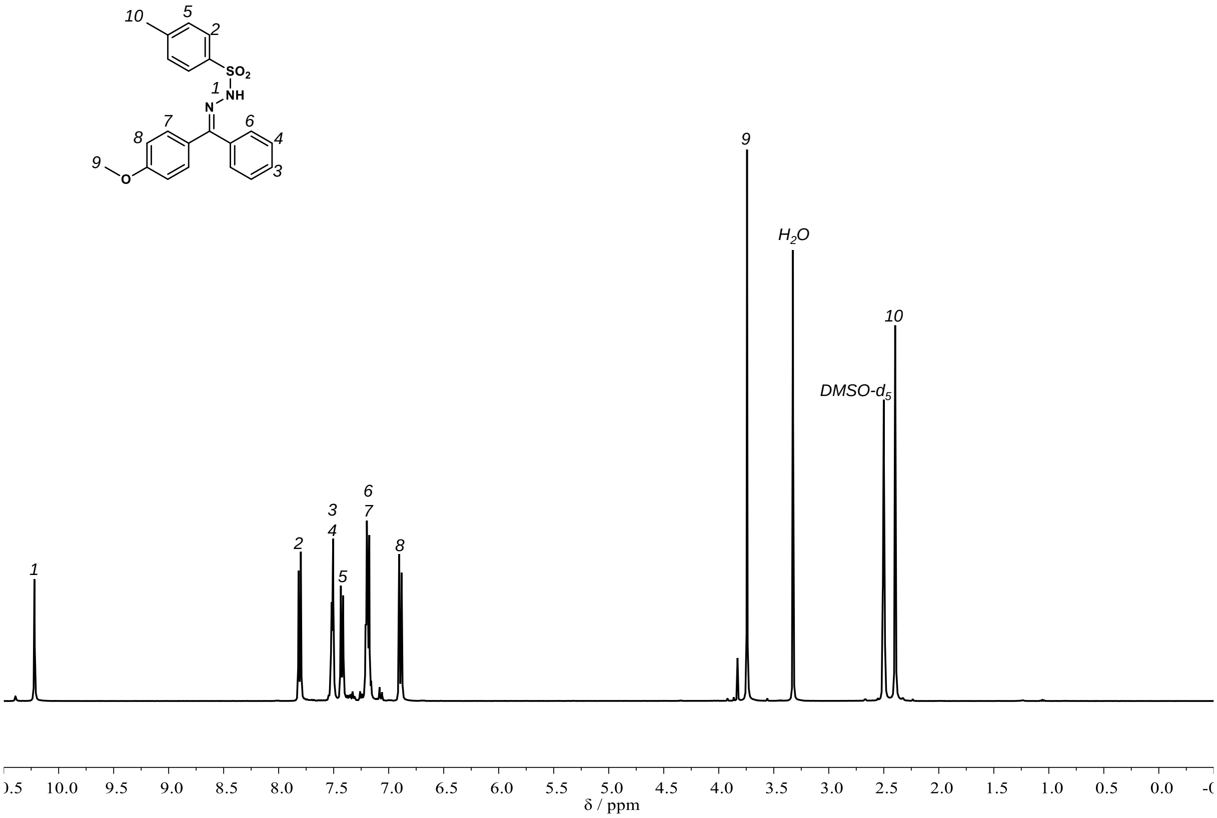

Supplementary Figure 21: ^{1}H-NMR spectrum of 1f.

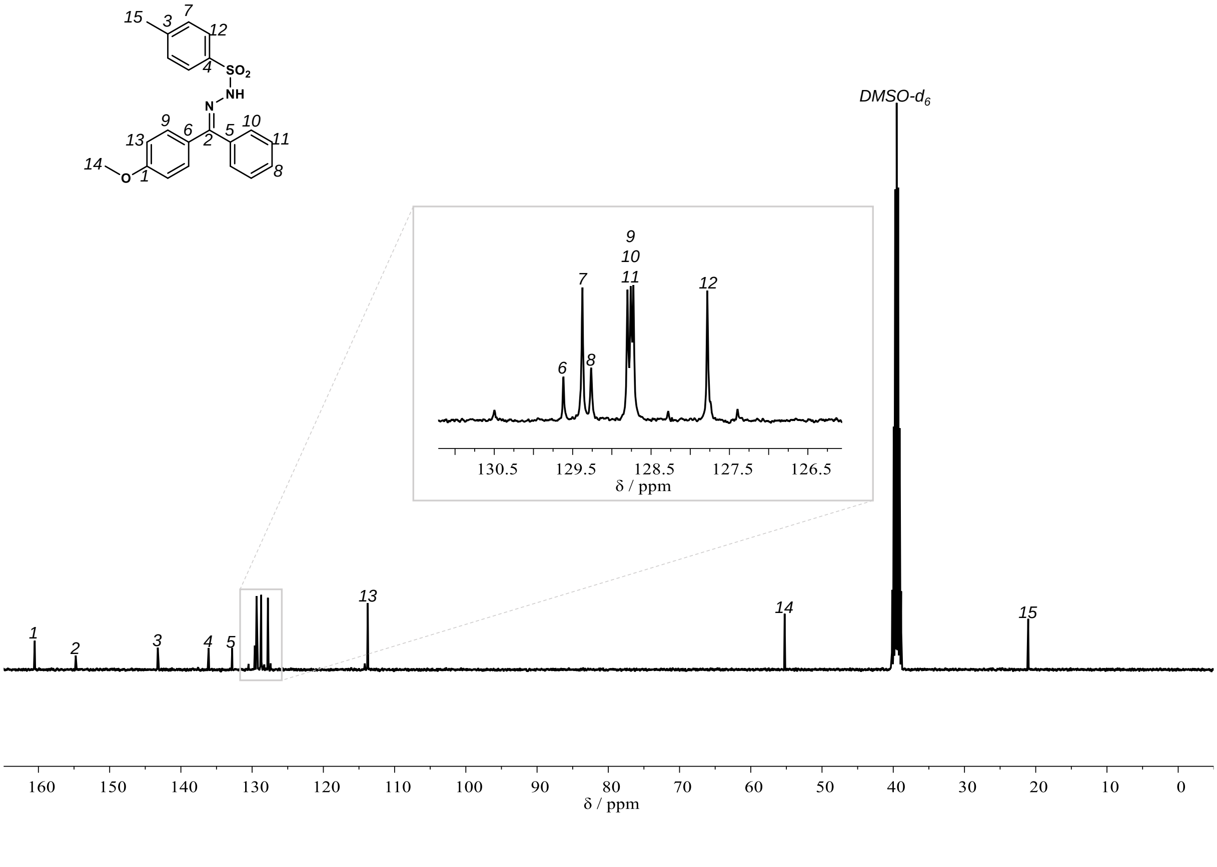

Supplementary Figure 22: ^{13}C-NMR spectrum of 1f.

***Synthesis of N'-(4-bromobenzhydrylidene)-p-toluenesulfonylhydrazide* (1g)**

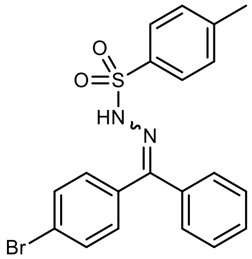

Prepared from **3g** according to general procedure A. Obtained as a white solid in a yield of 59% (1.27 g, 2.96 mmol). The isolated product consisted of a mixture of the *(E)-* and *(Z)-*stereoisomers in a ratio of 65:35.

^{1}H NMR (400 MHz, DMSO-d_6) δ/ppm = 10.59 (s, 1H, *(Z)*), 10.55 (s, 1H, *(E)*), 7.83 – 7.79 (m, 2H, *(E),* 2H, *(Z)*), 7.75 – 7.71 (m, 2H, *(Z)*), 7.58 – 7.51 (m, 5H, *(E)*), 7.46 – 7.42 (m, 2H, *(E),* 2H, *(Z)*), 7.40 – 7.32 (m, 3H, *(Z)*), 7.29 – 7.21 (m, 2H, *(E),* 2H, *(Z)*), 7.21 – 7.16 (m, 2H, *(E),* 2H, *(Z)*), 2.40 (s, 3H, *(E),* 3H, *(Z)*).

^{13}C NMR (101 MHz, DMSO-d_6) δ/ppm = 153.30, 153.03, 143.41, 136.69, 136.37, 136.05, 136.01, 132.05, 131.90, 131.69, 131.41, 131.09, 129.80, 129.55, 129.50, 129.48, 129.07, 128.92, 128.79, 128.43, 127.70, 127.62, 127.15, 123.22, 122.90, 21.06.

IR (ATR platinum diamond): v/cm^{-1} = 3275 (vw), 3209 (w), 3052 (vw), 3028 (vw), 2923 (vw), 2855 (vw), 1596 (w), 1491 (w), 1483 (w), 1444 (w), 1393 (s), 1349 (s), 1318 (s), 1306 (m), 1296 (w), 1185 (w), 1162 (vs), 1098 (w), 1094 (w), 1070 (vs), 1028 (w), 1022 (w), 1010 (m), 1000 (w), 981 (s), 950 (w), 924 (w), 882 (m), 866 (m), 847 (w), 825 (s), 810 (s), 773 (s), 718 (w), 701 (s), 693 (m), 679 (vs), 664 (vs), 652 (m), 625 (w), 607 (m), 555 (vs), 545 (vs), 483 (m), 446 (w).

ESI-HRMS m/z: $[M+H]^+$ calculated for $C_{20}H_{17}N_2O_2S^{79}Br$ = 429.0267, found 429.0263.

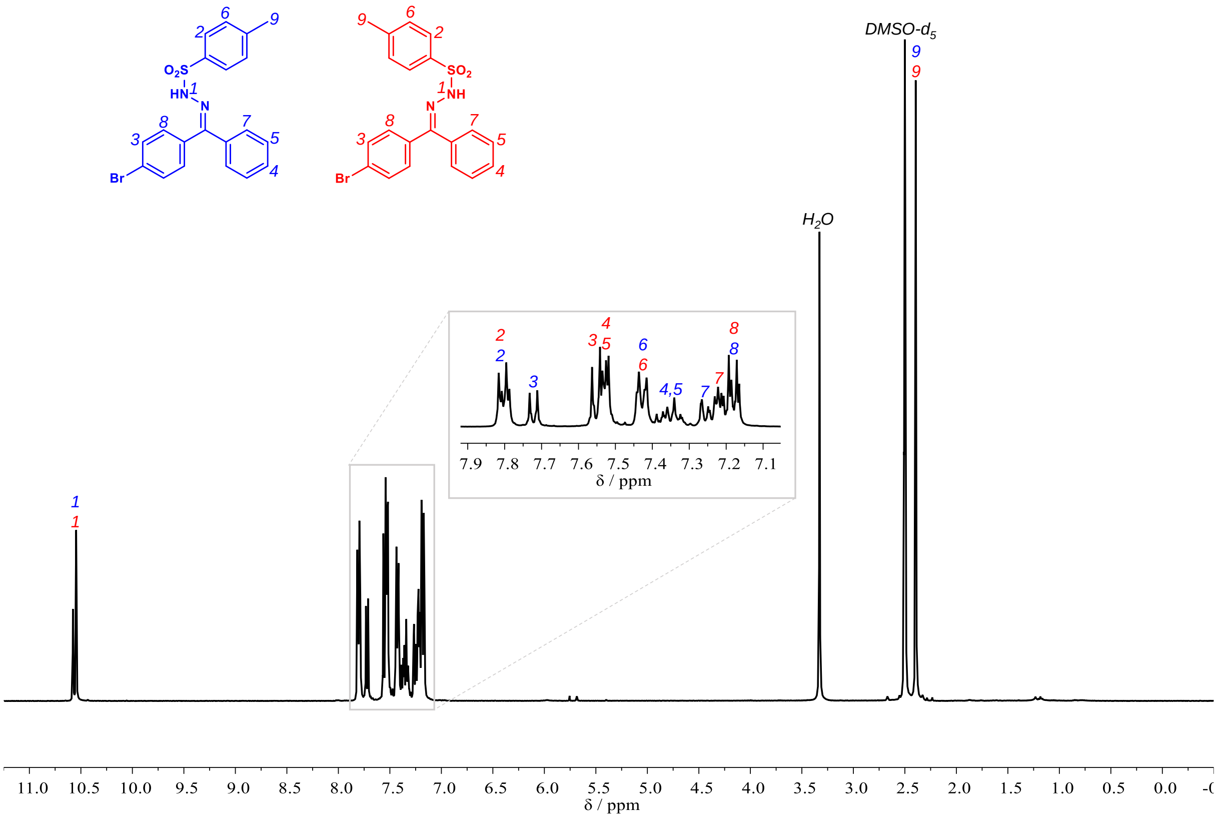

Supplementary Figure 23: ^{1}H-NMR spectrum of 1g.

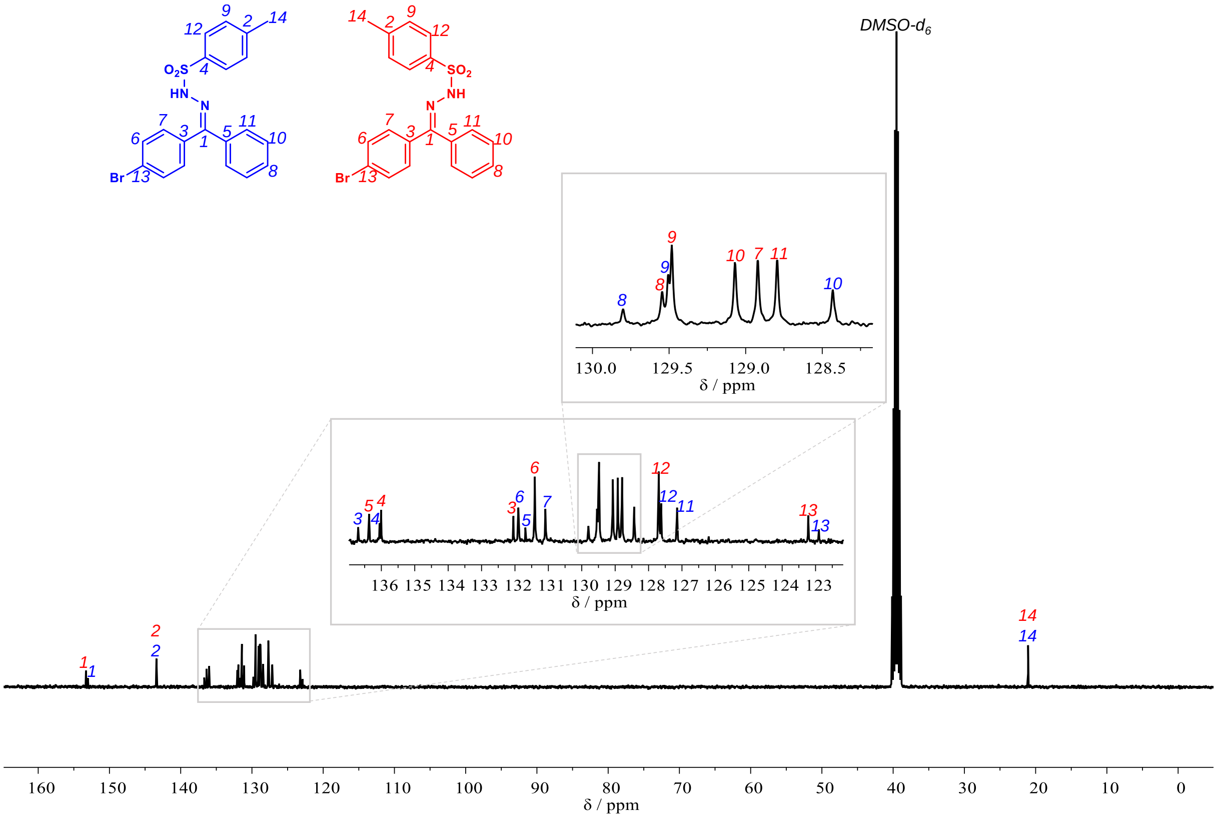

Supplementary Figure 24: ^{13}C-NMR spectrum of 1g.

***Synthesis of N'-(4-nitrobenzhydrylidene)-p-toluenesulfonylhydrazide* (1h)**

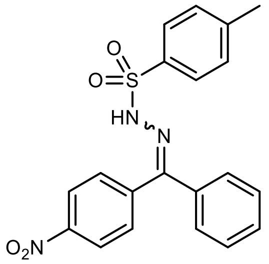

Prepared from **3h** according to general procedure A. Obtained as a slightly yellow solid in a yield of 75% (1.49 g, 3.77 mmol). The isolated product consisted only of the *(Z)*-stereoisomer.

^{1}H NMR (400 MHz, DMSO-d_6) δ/ppm = 10.67 (s, 1H), 8.39 – 8.34 (m, 2H), 7.83 – 7.78 (m, 2H), 7.55 – 7.50 (m, 2H), 7.46 – 7.42 (m, 2H), 7.42 – 7.33 (m, 3H), 7.29 – 7.24 (m, 2H), 2.40 (s, 3H).

^{13}C NMR (101 MHz, DMSO-d_6) δ/ppm = 152.04, 148.00, 143.55, 139.28, 136.15, 135.97, 130.67, 130.02, 129.60, 128.56, 127.58, 127.04, 124.08, 21.06.

IR (ATR platinum diamond): *v*/cm^{-1} = 3168 (w), 3063 (vw), 2915 (vw), 1596 (w), 1520 (s), 1448 (w), 1401 (w), 1347 (s), 1321 (m), 1308 (m), 1292 (w), 1185 (w), 1160 (vs), 1119 (w), 1107 (w), 1090 (w), 1049 (w), 1014 (w), 975 (w), 884 (m), 854 (s), 847 (s), 817 (w), 771 (m), 765 (m), 738 (w), 695 (vs), 671 (vs), 627 (m), 617 (w), 547 (vs), 500 (m), 479 (w), 428 (vw).

ESI-HRMS m/z: $[M+H]^+$ calculated for $C_{20}H_{17}N_3O_4S$ = 396.1008, found 396.1013.

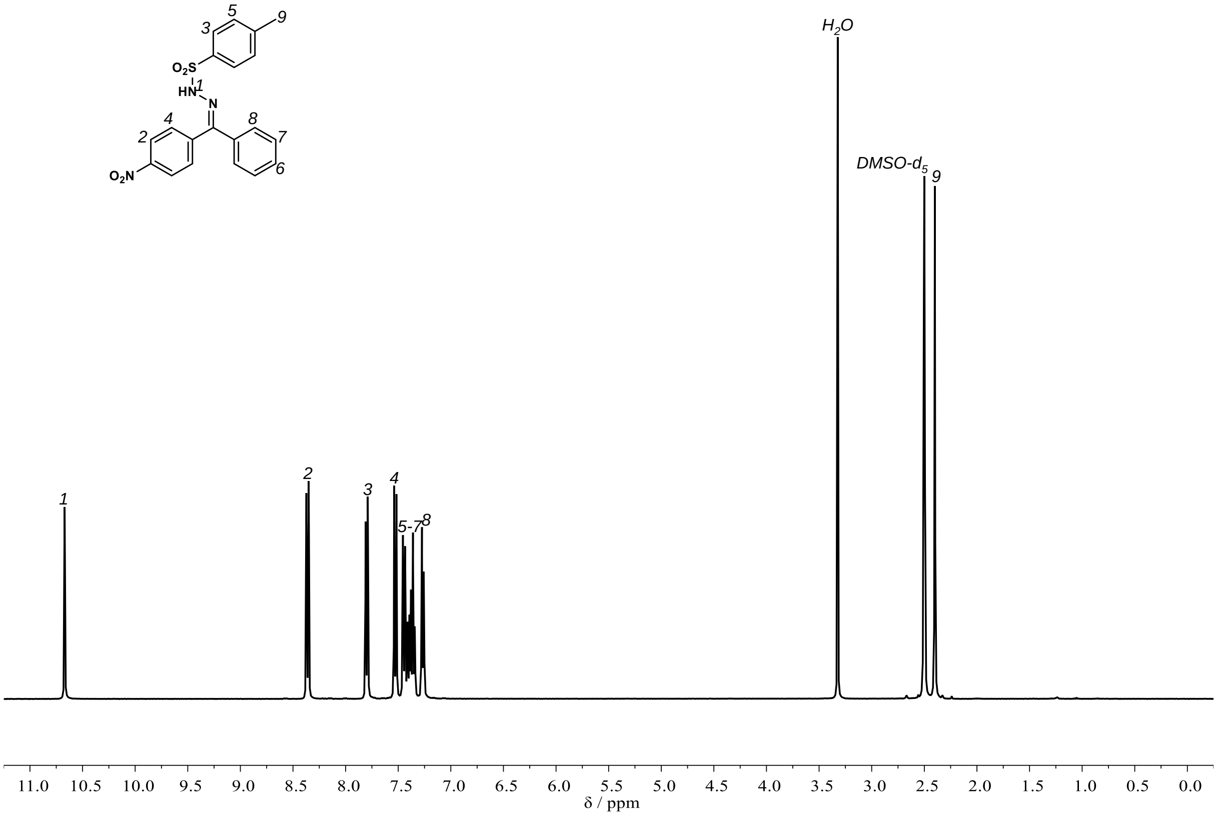

Supplementary Figure 25: ^{1}H-NMR spectrum of 1h.

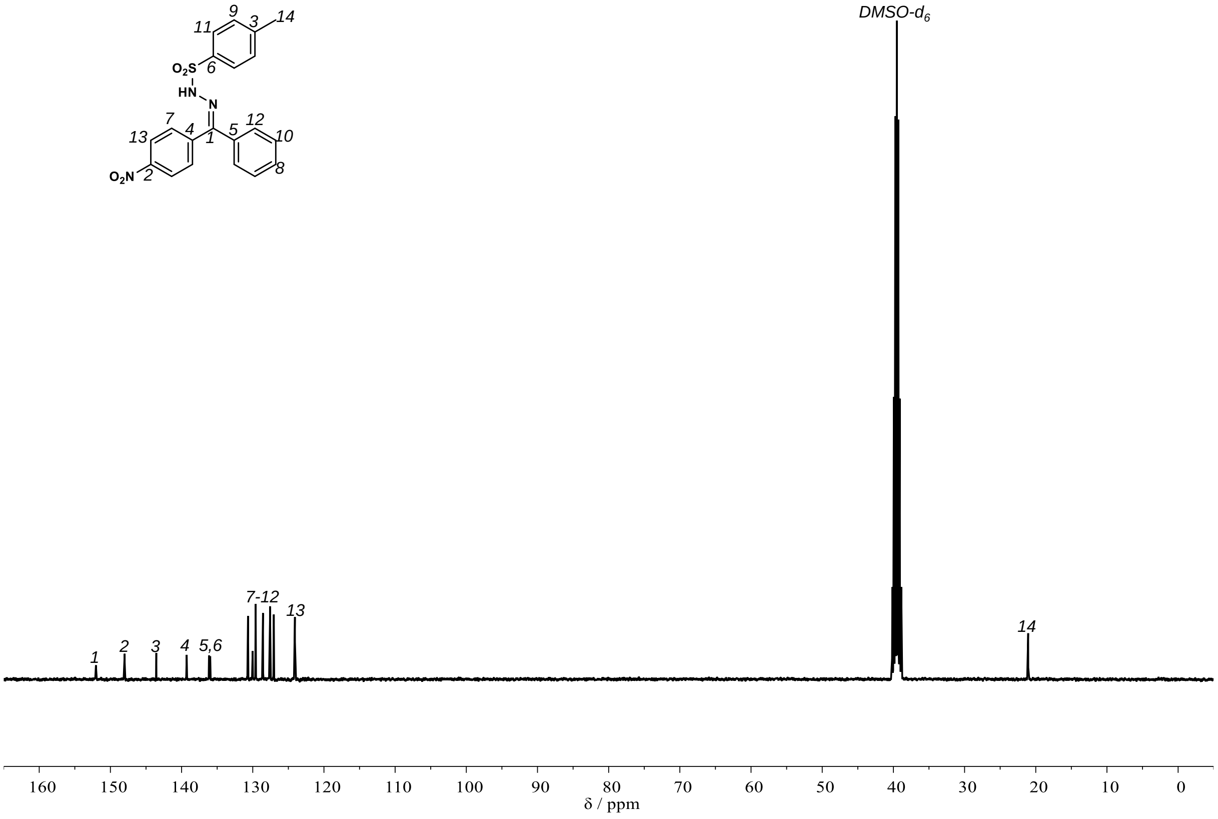

Supplementary Figure 26: ^{13}C-NMR spectrum of 1h.

Synthesis of N’-(4-methoxybenzylidene)-p-toluenesulfonylhydrazide **(1i)**

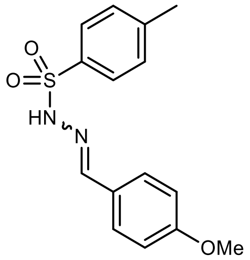

Prepared from **3i** according to general procedure B. Obtained as a white solid in a yield of 98% (1.49 g, 4.90 mmol). The isolated product consisted only of the *(E)*-stereoisomer.

^{1}H NMR (400 MHz, DMSO-d_6) δ/ppm = 11.21 (s, 1H), 7.85 (s, 1H), 7.79 – 7.72 (m, 2H), 7.53 – 7.45 (m, 2H), 7.43 – 7.36 (m, 2H), 6.98 – 6.90 (m, 2H), 3.76 (s, 3H), 2.35 (s, 3H).

^{13}C NMR (101 MHz, DMSO-d_6) δ/ppm = 160.77, 147.02, 143.33, 136.21, 129.60, 128.35, 127.24, 126.27, 114.25, 55.27, 20.99.

IR (ATR platinum diamond): ν/cm^{-1} = 3219 (m), 3020 (vw), 2968 (vw), 2839 (vw), 1609 (m), 1594 (w), 1574 (w), 1518 (m), 1462 (w), 1436 (w), 1425 (m), 1382 (w), 1360 (m), 1323 (m), 1300 (s), 1257 (vs), 1172 (m), 1158 (vs), 1117 (w), 1107 (m), 1088 (m), 1033 (vs), 1020 (m), 950 (vs), 932 (w), 837 (w), 827 (vs), 810 (s), 767 (w), 701 (w), 662 (vs), 642 (w), 570 (vs), 547 (vs), 529 (vs), 506 (vs), 442 (w).

ESI-HRMS m/z: [M+H]$^+$ calculated for $C_{15}H_{16}N_2O_3S$ = 305.0948, found 305.0954.

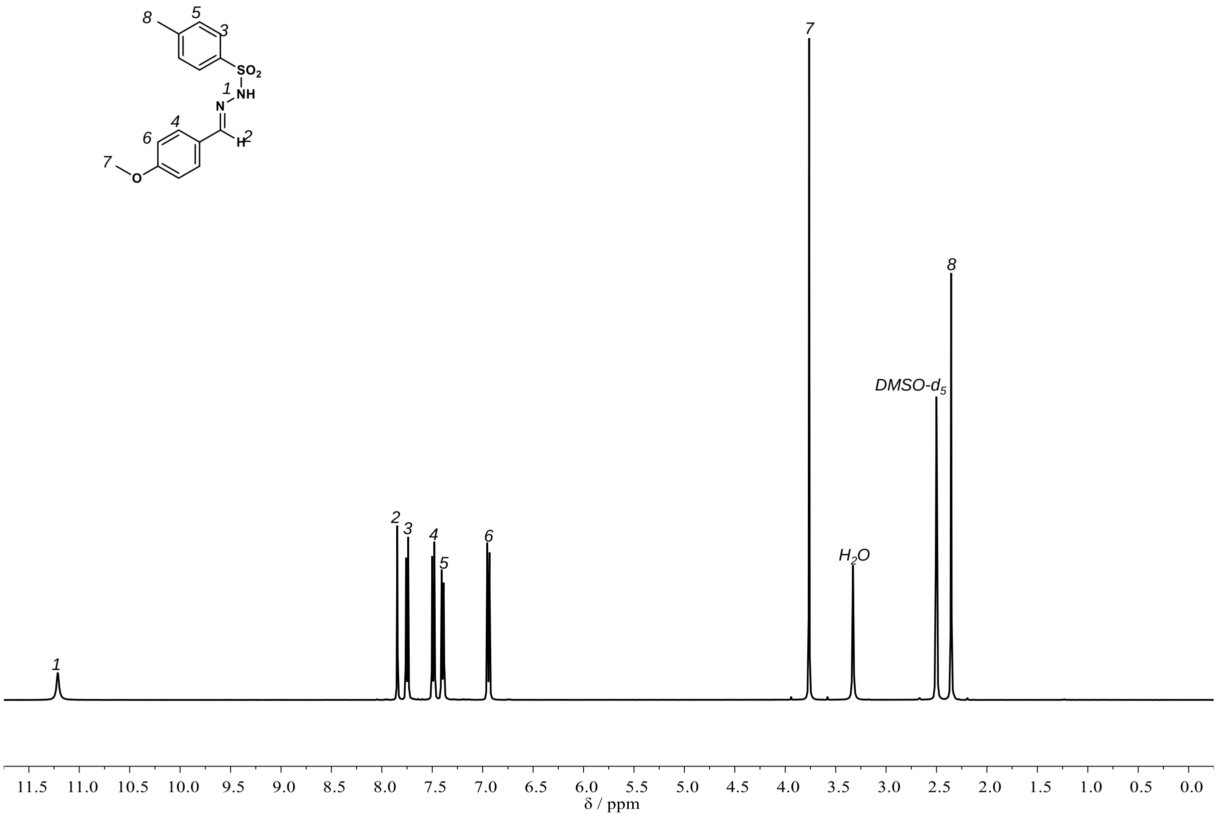

Supplementary Figure 27: ^{1}H-NMR spectrum of 1i.

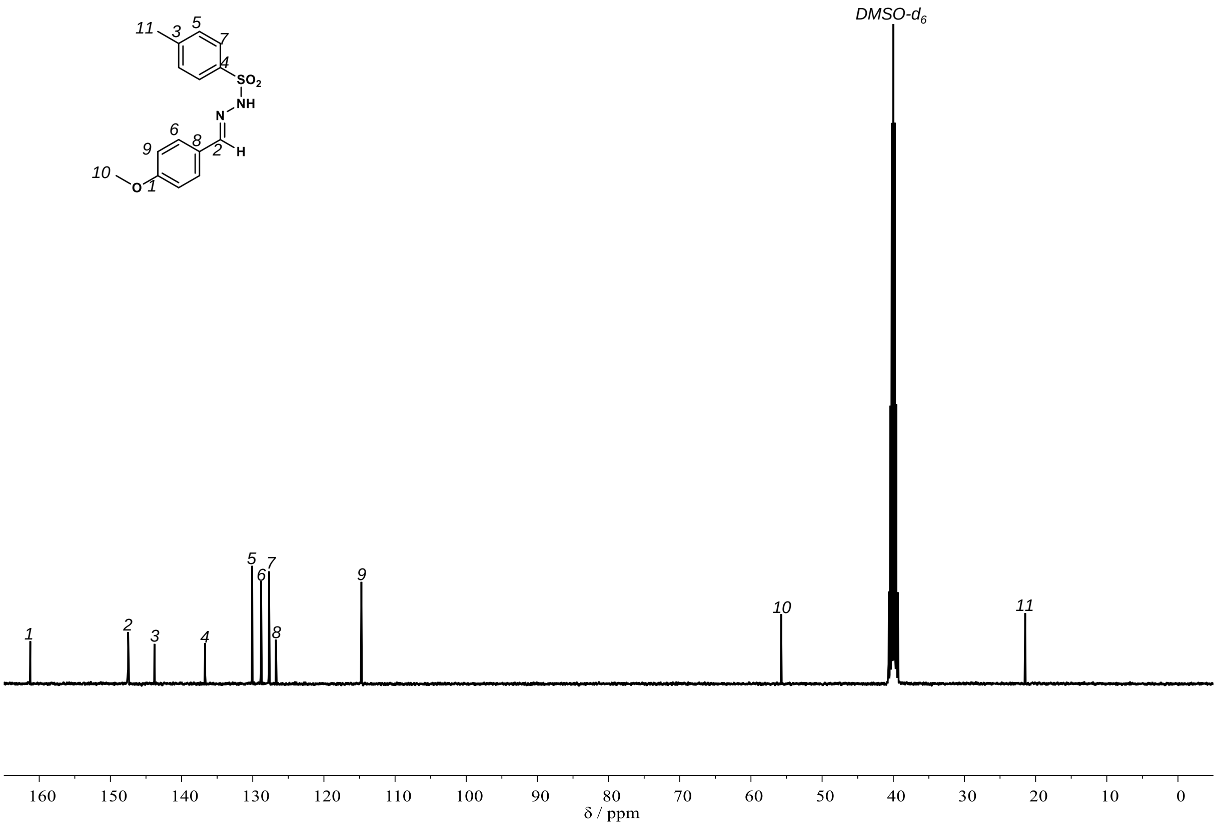

Supplementary Figure 28: ^{13}C-NMR spectrum of 1i.

***Synthesis of N'-(1-(4-methoxyphenyl)ethylidene)-p-toluenesulfonylhydrazide* (1j)**

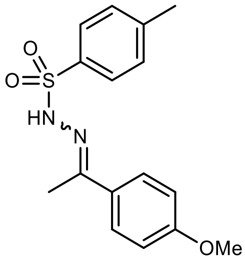

Prepared from **3j** according to general procedure A. Obtained as a white solid in a yield of 85% (1.35 g, 4.23 mmol). The isolated product consisted only of the *(E)*-stereoisomer.

^{1}H NMR (400 MHz, DMSO-d_6) δ/ppm = 10.33 (s, 1H), 7.84 – 7.77 (m, 2H), 7.61 – 7.53 (m, 2H), 7.44 – 7.37 (m, 2H), 6.96 – 6.87 (m, 2H), 3.76 (s, 3H), 2.37 (s, 3H), 2.14 (s, 3H).

^{13}C NMR (101 MHz, DMSO-d_6) δ/ppm = 160.28, 153.16, 143.25, 136.27, 129.83, 129.41, 127.60, 127.45, 113.70, 55.20, 21.01, 14.14.

IR (ATR platinum diamond): v/cm^{-1} = 3221 (w), 3003 (vw), 2962 (vw), 2933 (vw), 2834 (vw), 1615 (w), 1598 (m), 1516 (m), 1456 (w), 1440 (w), 1399 (m), 1374 (w), 1337 (w), 1312 (s), 1302 (vs), 1267 (vs), 1177 (s), 1162 (vs), 1121 (m), 1096 (w), 1084 (m), 1045 (m), 1031 (s), 1020 (m), 965 (w), 911 (s), 837 (s), 812 (vs), 798 (w), 716 (vs), 681 (vs), 634 (w), 590 (s), 562 (m), 553 (vs), 539 (vs), 527 (vs), 506 (w), 479 (w), 438 (w).

ESI-HRMS m/z: [M+H]$^+$ calculated for $C_{16}H_{18}N_2O_3S$ = 319.1111, found 319.1104.

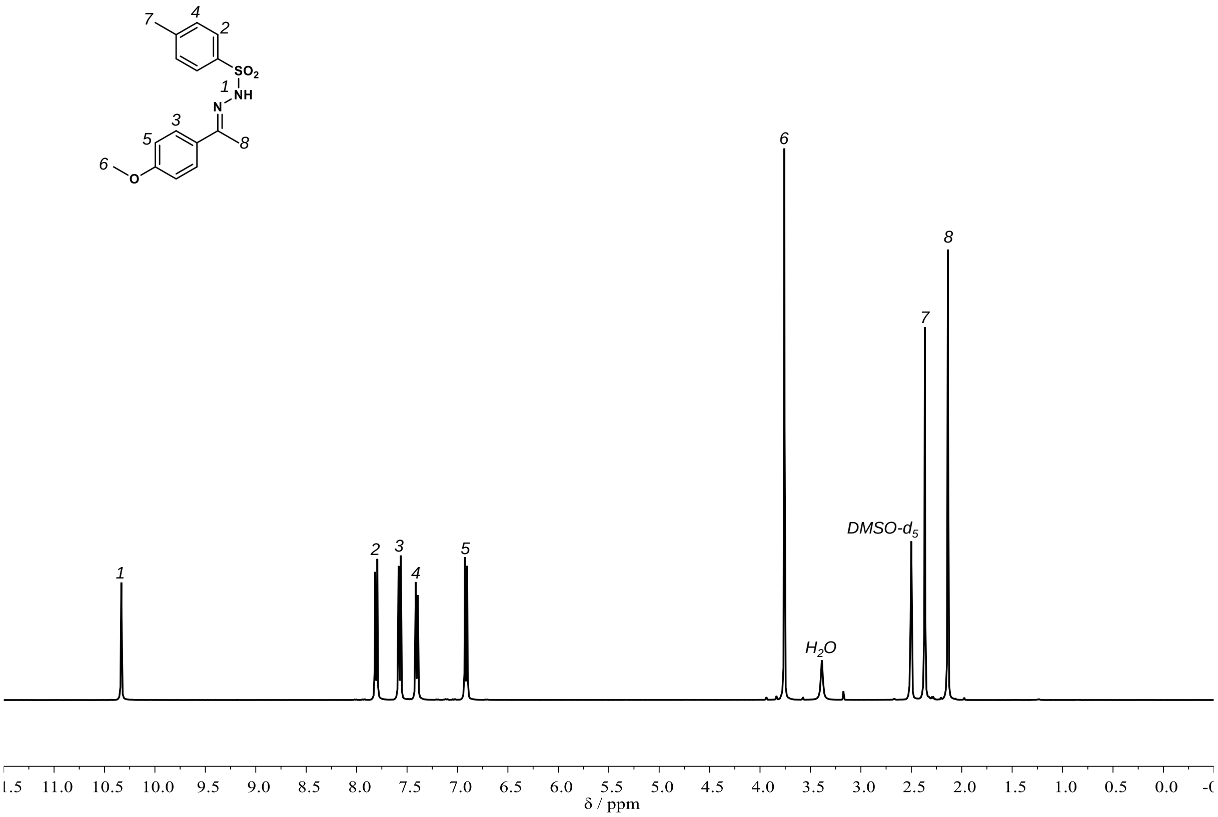

Supplementary Figure 29: ^{1}H-NMR spectrum of 1j.

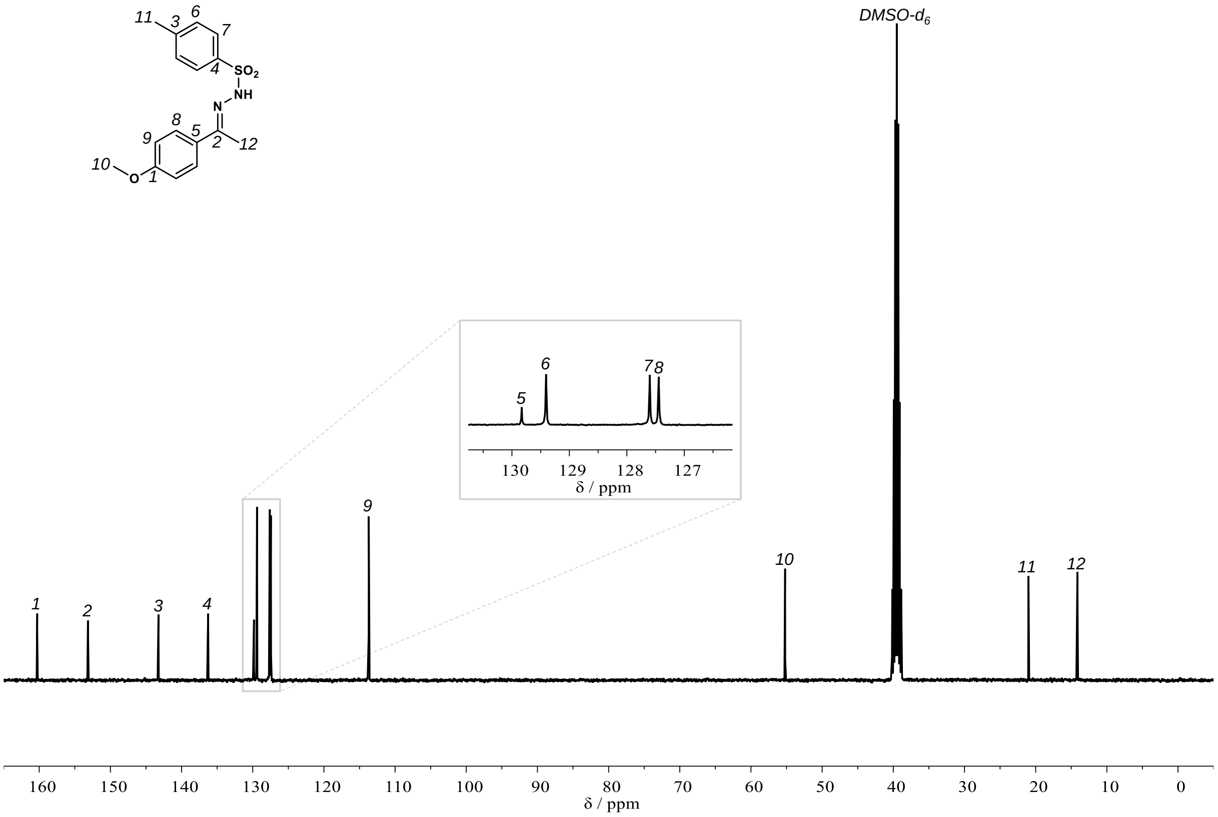

Supplementary Figure 30: ^{13}C-NMR spectrum of 1j.

***Synthesis of N'-(2-adamantylidene)-p-toluenesulfonylhydrazide* (1k)**

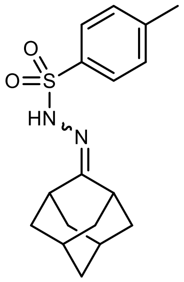

Prepared from **3k** according to general procedure A. Obtained as a white solid in a yield of 64% (1.35 g, 4.23 mmol).

^{1}H NMR (400 MHz, DMSO-*d*$_6$) δ/ppm = 10.06 (s, 1H), 7.76 – 7.69 (m, 2H), 7.42 – 7.36 (m, 2H), 3.14 (s, 1H), 2.38 (s, 4H), 1.91 – 1.80 (m, 6H), 1.80 – 1.74 (m, 2H), 1.67 – 1.56 (m, 4H).

^{13}C NMR (101 MHz, DMSO-*d*$_6$) δ/ppm = 168.82, 142.82, 136.54, 129.30, 127.40, 38.61, 38.51, 37.11, 35.67, 31.33, 26.95, 21.00.

IR (ATR platinum diamond): v/cm^{-1} = 3221 (w), 2917 (w), 2853 (w), 1646 (vw), 1596 (w), 1495 (vw), 1446 (w), 1403 (w), 1345 (w), 1321 (m), 1304 (w), 1294 (w), 1166 (vs), 1158 (vs), 1090 (w), 1084 (w), 1035 (w), 1008 (m), 989 (m), 950 (w), 921 (s), 887 (w), 843 (w), 812 (m), 800 (w), 780 (vw), 732 (w), 703 (w), 675 (vs), 644 (s), 627 (w), 613 (w), 603 (w), 557 (vs), 543 (vs), 514 (w), 481 (w), 438 (w).

ESI-HRMS m/z: [M+H]$^+$ calculated for $C_{17}H_{22}N_2O_2S$ = 319.1475, found 319.1471.

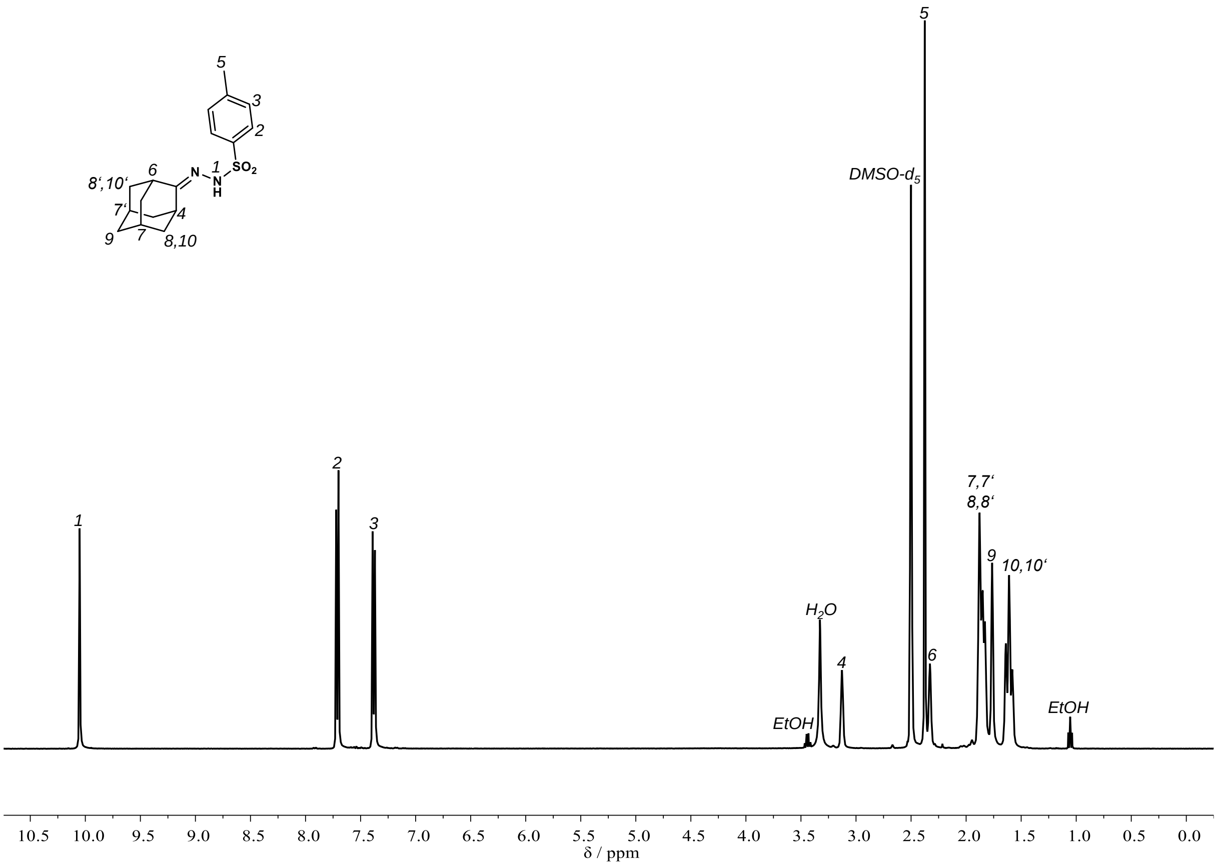

Supplementary Figure 31: ^{1}H-NMR spectrum of 1k.

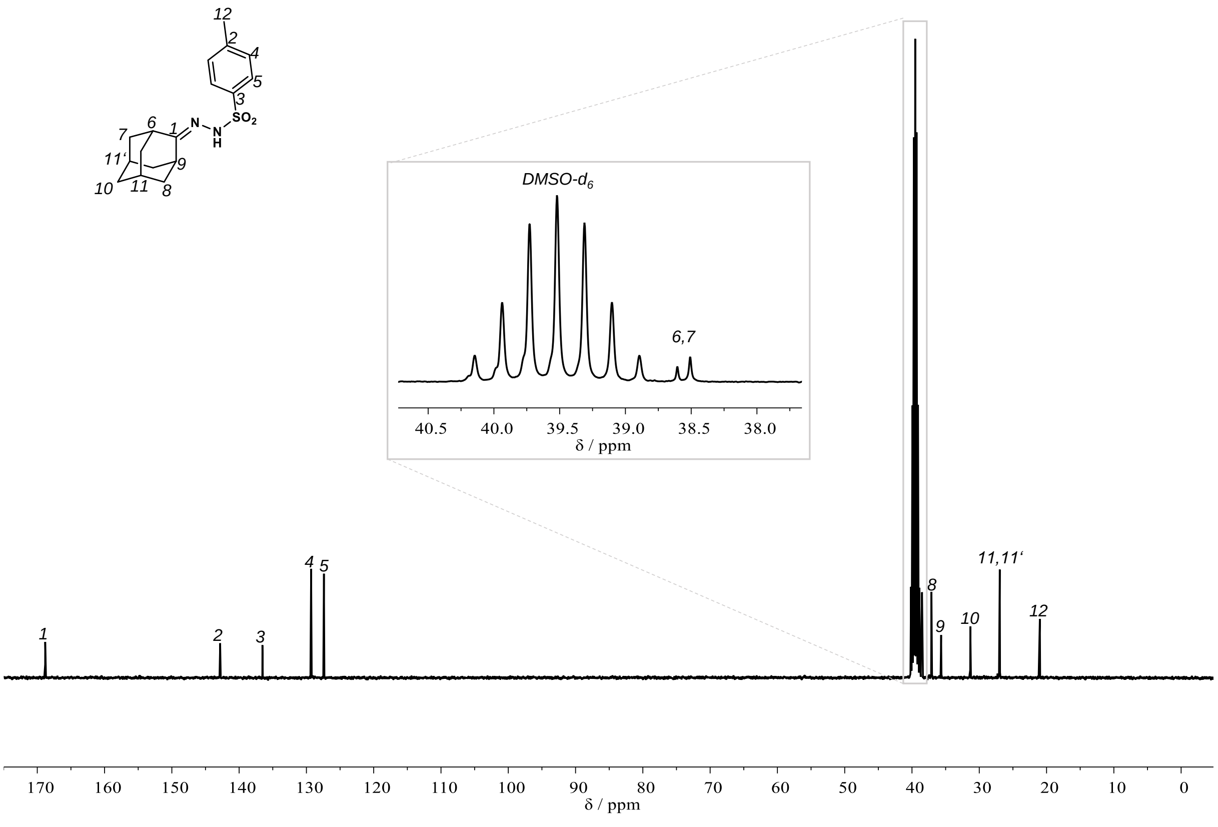

Supplementary Figure 32: ^{13}C-NMR spectrum of 1k.

***Synthesis of N'-(3-pentylidene)-p-toluenesulfonylhydrazide* (1l)**

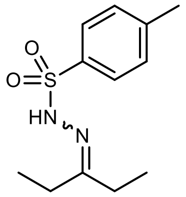

Prepared from **3l** according to general procedure B. Obtained as a white solid in quantitative yield (1.27 g, 5.00 mmol).

^{1}H NMR (400 MHz, DMSO-d_6) δ/ppm = 9.96 (s, 1H), 7.78 – 7.64 (m, 2H), 7.44 – 7.30 (m, 2H), 2.37 (s, 3H), 2.19 (q, *J* = 7.6 Hz, 2H), 2.12 (q, *J* = 7.3 Hz, 2H), 0.93 (t, *J* = 7.6 Hz, 3H), 0.87 (t, *J* = 7.3 Hz, 3H).

^{13}C NMR (101 MHz, DMSO-d_6) δ/ppm =163.61, 142.94, 136.27, 129.20, 127.54, 28.33, 22.62, 20.99, 10.29, 9.44.

IR (ATR platinum diamond): v/cm^{-1} = 3217 (w), 2980 (w), 2941 (vw), 2904 (vw), 2884 (vw), 1637 (vw), 1596 (w), 1495 (vw), 1465 (w), 1390 (w), 1376 (w), 1362 (w), 1335 (vs), 1308 (w), 1292 (w), 1189 (w), 1162 (vs), 1090 (m), 1020 (w), 993 (w), 950 (w), 909 (m), 812 (m), 767 (w), 706 (m), 679 (vs), 629 (w), 582 (vs), 555 (vs), 537 (s), 487 (w), 455 (w).

ESI-HRMS m/z: [M]$^{+}$ calculated for $C_{12}H_{18}N_2O_2S$ = 255.1084, found 255.1805.

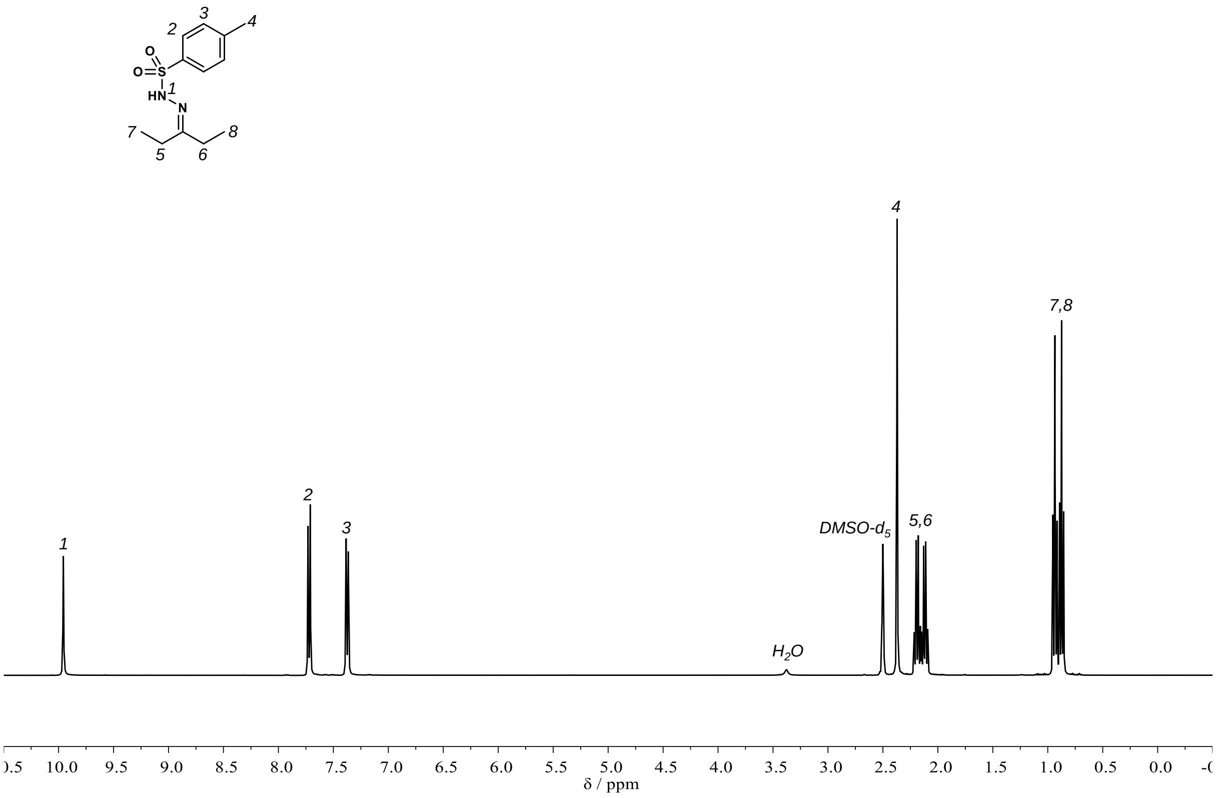

Supplementary Figure 33: ^{1}H-NMR spectrum of 1l.

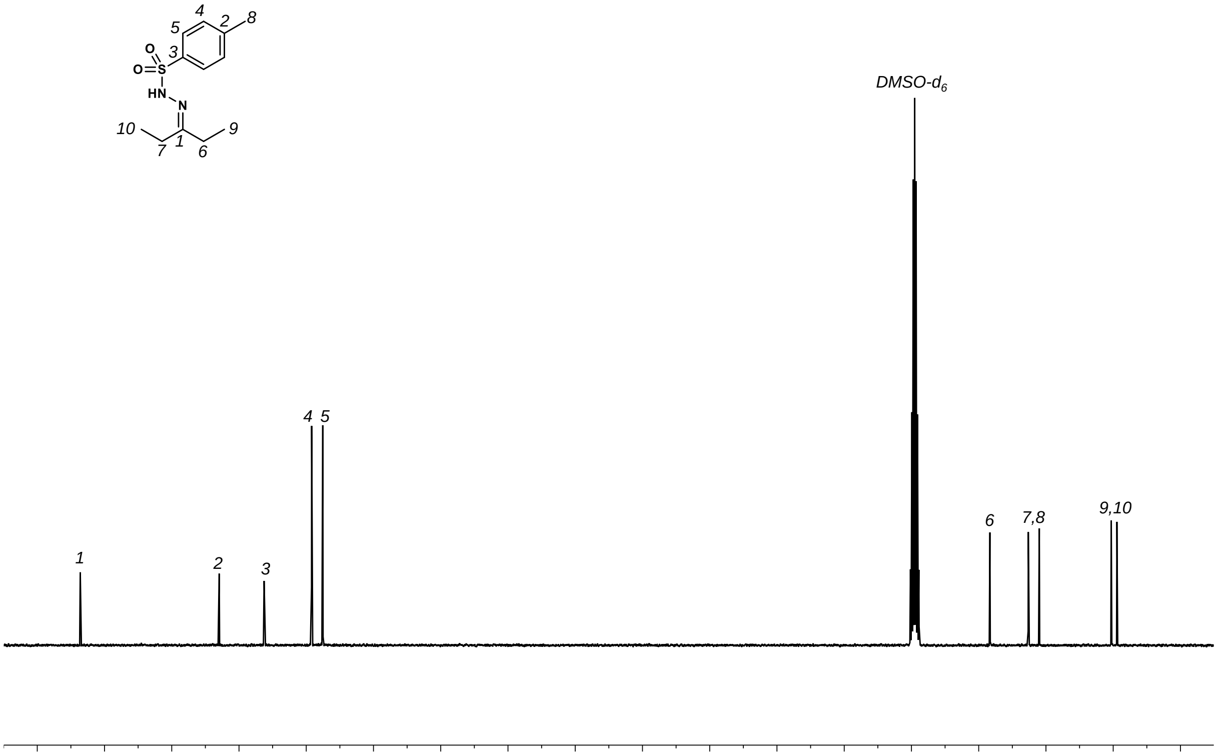

Supplementary Figure 34: ^{13}C-NMR spectrum of 1l.

1.3.3 Thioketones

Synthesis of 4,4-dimethoxythiobenzophenone **(5a)**

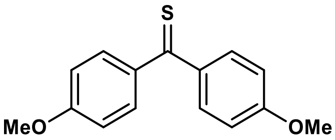

In a flame-dried Schlenk flask under argon atmosphere, 1.00 g (4.13 mmol, 1.00 eq.) of 4,4'-dimethoxybenzophenone (**3a**) were dispersed in 40 mL of dry toluene and Lawesson's reagent (1.34 g, 3.30 mmol, 0.80 eq.) was added. The mixture was refluxed for 16 hours under light exclusion. Afterwards, the crude reaction mixture was transferred onto a short filter column (cyclohexane with 3 vol% NEt_3) and all blue fractions were collected. After removal of the solvent under reduced pressure, the crude product was purified *via* another subsequent column chromatography (cyclohexane/ethyl acetate 30:1 with 1 vol% NEt_3). The product was isolated as a blue solid in a yield of 70% (860 mg, 3.33 mmol).

^{1}H NMR (400 MHz, DMSO-d_6) δ/ppm = 7.70 – 7.62 (m, 2H), 7.06 – 6.98 (m, 2H), 3.86 (s, 3H).

^{13}C NMR (101 MHz, DMSO-d_6) δ/ppm = 233.15, 163.57, 140.47, 132.44, 114.02, 56.15.

IR (ATR platinum diamond): v/cm^{-1} = 3007 (vw), 2968 (vw), 2929 (vw), 2834 (w), 1588 (s), 1567 (m), 1502 (m), 1454 (m), 1440 (m), 1417 (w), 1300 (s), 1249 (vs), 1216 (s), 1162 (vs), 1117 (s), 1113 (s), 1045 (s), 1020 (vs), 963 (m), 897 (w), 833 (vs), 804 (s), 775 (w), 738 (w), 644 (w), 617 (vs), 543 (s), 508 (m).

ESI-HRMS m/z: $[M+H]^+$ calculated for $C_{15}H_{15}O_2S$ = 259.0787, found 259.0784

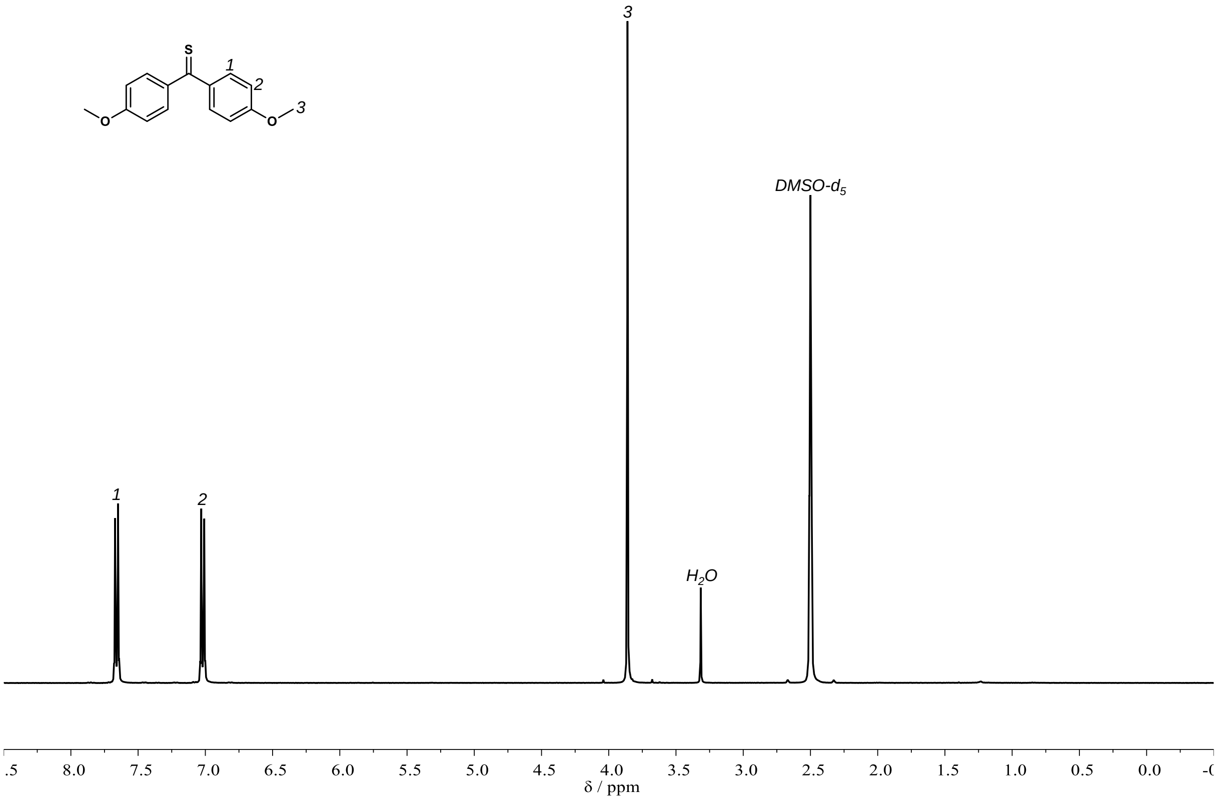

Supplementary Figure 35: ^{1}H-NMR spectrum of 5a.

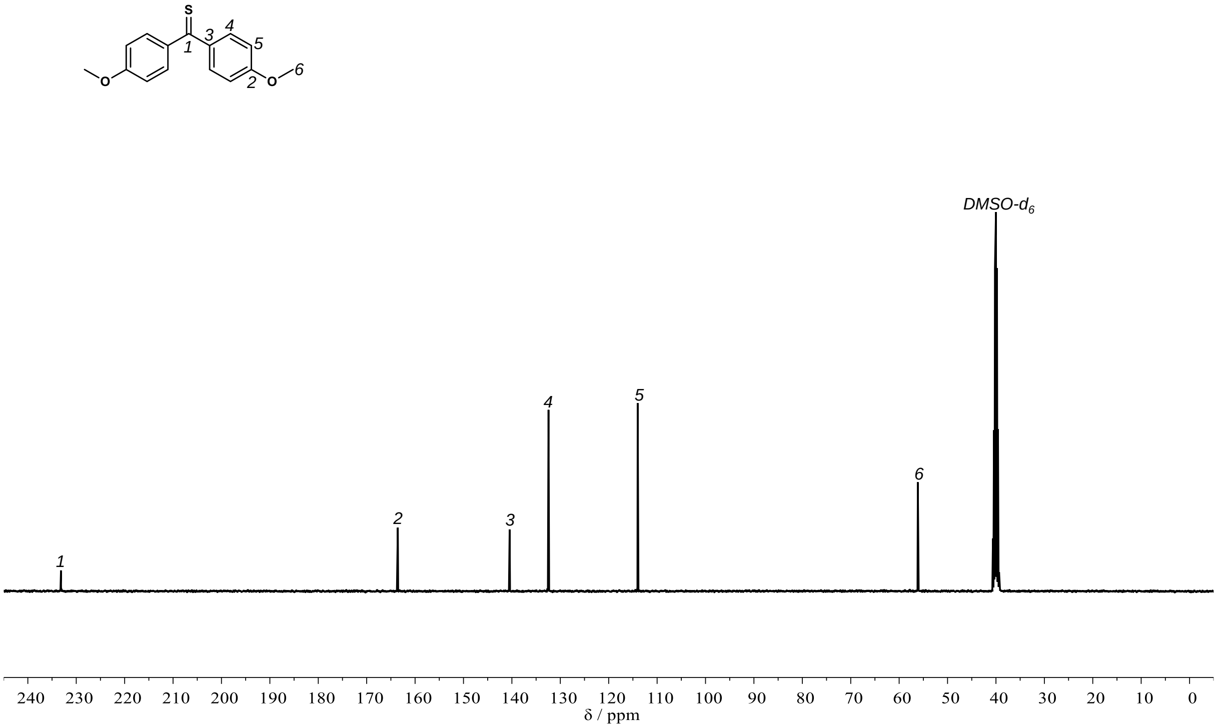

Supplementary Figure 36: ^{13}C-NMR spectrum of 5a.

***Synthesis of thioxanthene-9-thione* (5b)**

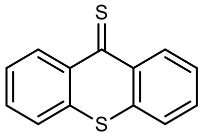

In a flame-dried Schlenk flask under argon atmosphere, 1.06 g (5.00 mmol, 1.00 eq.) of thioxanthene-9-one (**3m**) were dispersed in 50 mL of dry toluene and Lawesson's reagent (1.62 g, 4.00 mmol, 0.80 eq.) was added. The mixture was refluxed for 16 hours under light exclusion. Afterwards, the crude reaction mixture was transferred onto a short filter column (cyclohexane with 3 vol% NEt_3) and all dark green fractions were collected. The product was obtained as a dark green solid in a yield 94% (1.07 g, 4.70 mmol).

^{1}H NMR (400 MHz, DMSO-d_6) δ/ppm = 8.91 (dd, *J* = 8.4, 1.4 Hz, 1H), 7.90 (dd, *J* = 8.0, 1.4 Hz, 1H), 7.81 (ddd, *J* = 8.2, 7.0, 1.4 Hz, 1H), 7.59 (ddd, *J* = 8.4, 7.0, 1.4 Hz, 1H).

^{13}C NMR (101 MHz, DMSO-d_6) δ/ppm = 210.34, 136.94, 133.16, 133.11, 132.06, 128.10, 127.11.

IR (ATR platinum diamond): ν/cm^{-1} = 3048 (vw), 1586 (w), 1555 (w), 1456 (w), 1444 (w), 1417 (m), 1314 (w), 1284 (w), 1261 (m), 1187 (s), 1156 (m), 1113 (w), 1096 (m), 1072 (w), 1033 (w), 1006 (s), 959 (w), 952 (w), 897 (w), 854 (w), 769 (vs), 747 (w), 720 (vs), 660 (w), 621 (m), 607 (m), 529 (w), 475 (w) cm^{-1}.

ESI-HRMS m/z: $[M+H]^+$ calculated for $C_{13}H_8S_2$ = 229.0141, found 229.0141.

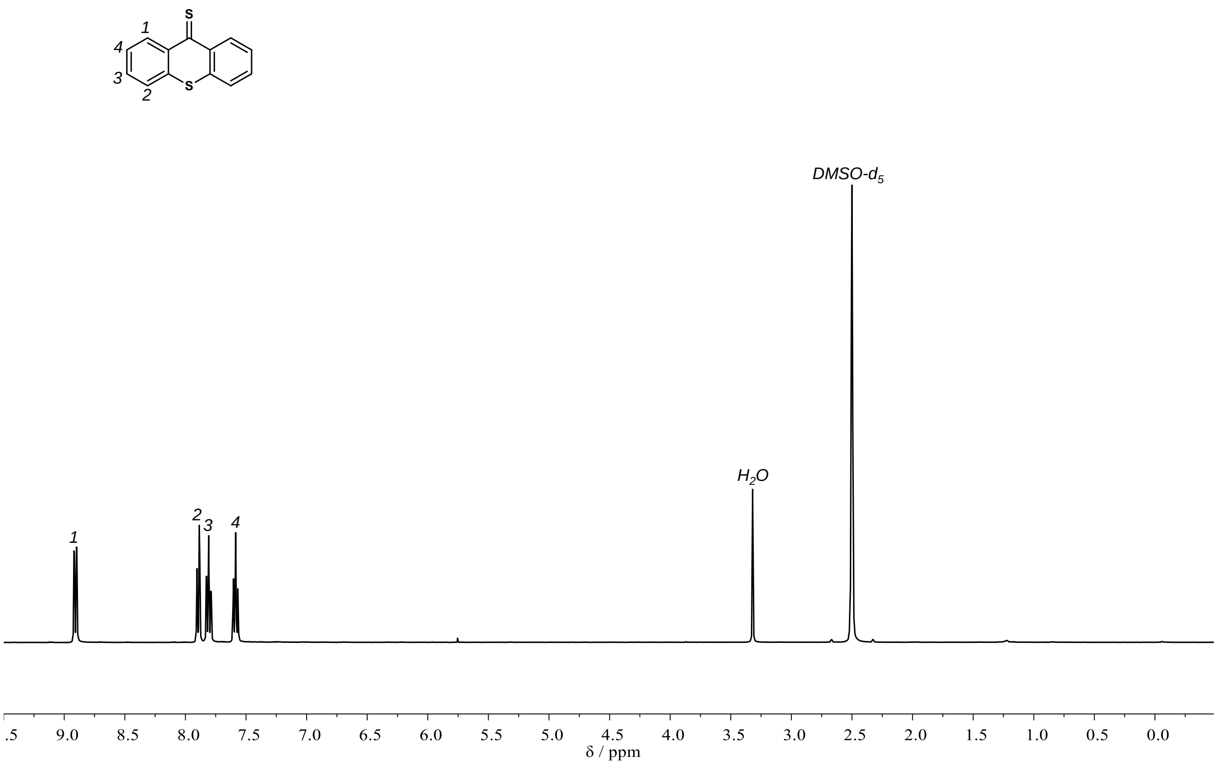

Supplementary Figure 37: ^{1}H-NMR spectrum of 5b.

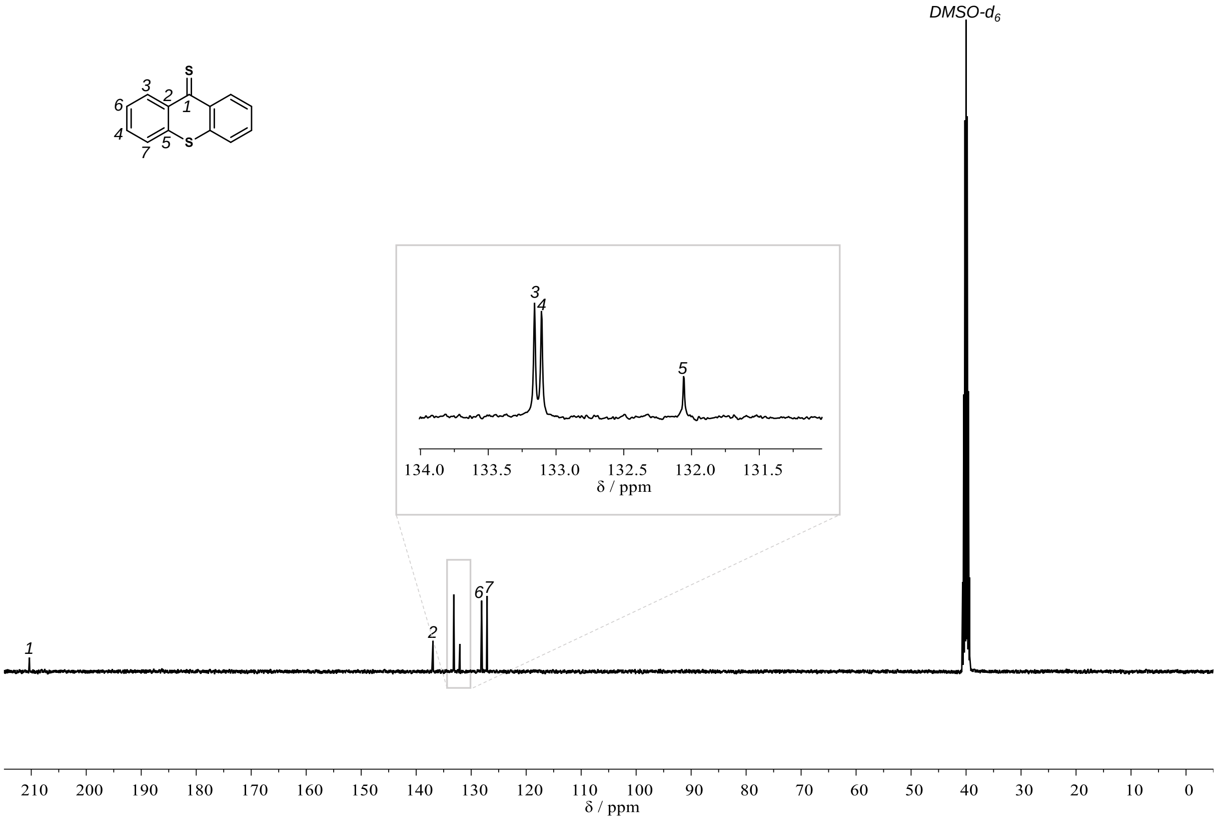

Supplementary Figure 38: ^{13}C-NMR spectrum of 5b.

***Synthesis of 4,4'-dibromothiobenzophenone* (5c)**

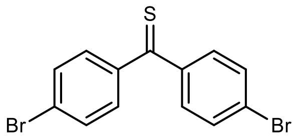

In a flame-dried Schlenk flask under argon atmosphere, 1.06 g (5.00 mmol, 1.00 eq.) of 4,4'-dibromobenzophenone (**3c**) were dispersed in 50 mL of dry toluene and Lawesson's reagent (1.62 g, 4.00 mmol, 0.80 eq.) was added. The mixture was refluxed for 16 hours under light exclusion. Afterwards, the crude reaction mixture was transferred onto a short filter column (cyclohexane with 3 vol% NEt_3) and all blue fractions were collected. The product was obtained as a blue solid in a yield of 83% (1.48 g, 4.15 mmol). Impurities in the ^{1}H-NMR spectra are due to contamination with ketone **3c** present caused by partial decomposition of the product before the measurements.

^{1}H NMR (400 MHz, DMSO-d_6) δ/ppm = 7.76 – 7.66 (m, 4H), 7.64 – 7.53 (m, 4H).

^{13}C NMR (101 MHz, DMSO-d_6) δ/ppm = 235.07, 145.16, 131.46, 131.06, 127.05.

IR (ATR platinum diamond): v/cm^{-1} = 3057 (w), 2917 (w), 2849 (w), 1643 (vs), 1574 (vs), 1481 (s), 1417 (w), 1393 (s), 1316 (w), 1302 (m), 1286 (s), 1273 (s), 1263 (vs), 1207 (s), 1174 (s), 1146 (w), 1123 (w), 1105 (w), 1070 (vs), 1051 (s), 1004 (vs), 967 (m), 924 (s), 889 (m), 854 (m), 825 (vs), 749 (vs), 722 (s), 695 (w), 662 (m), 629 (w), 611 (m), 520 (w), 477 (w), 469 (s), 455 (w), 436 (w).

ESI-HRMS m/z: $[M+H]^+$ calculated for $C_{13}H_8S^{79}Br^{81}Br$ = 356.8766, found 356.8762.

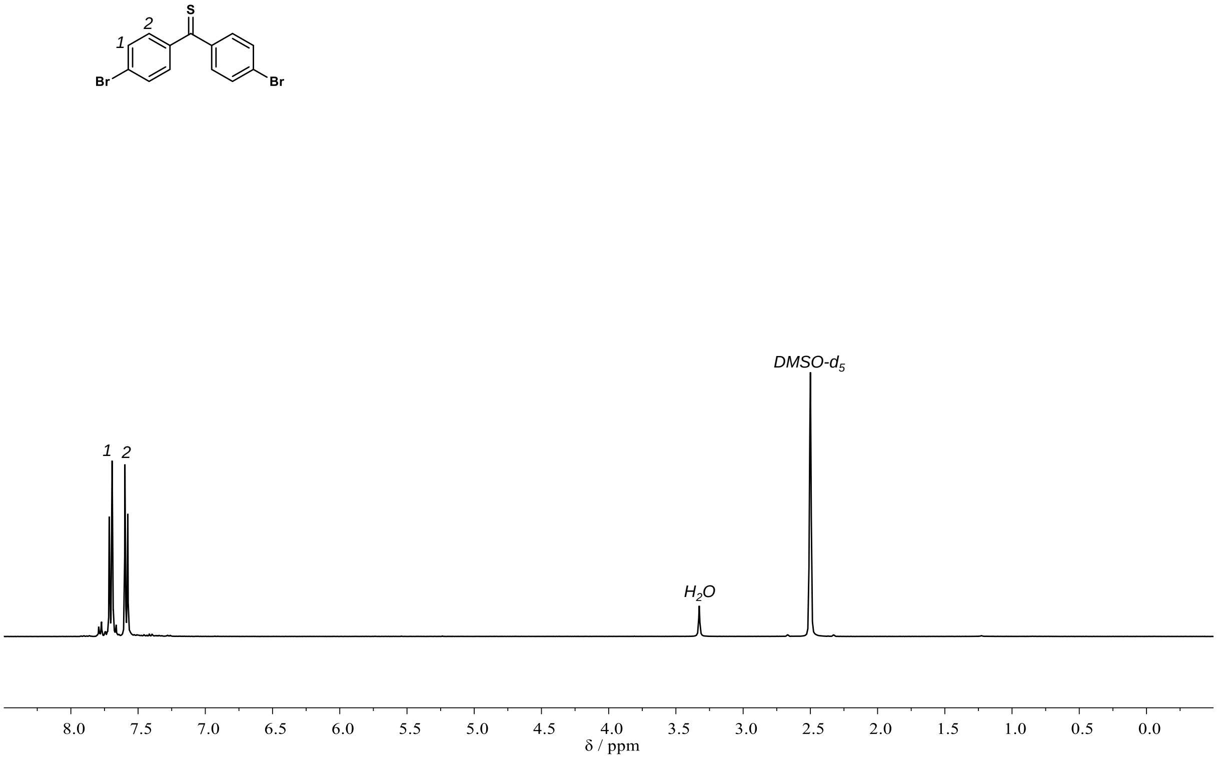

Supplementary Figure 39: ^{1}H-NMR spectrum of 5c.

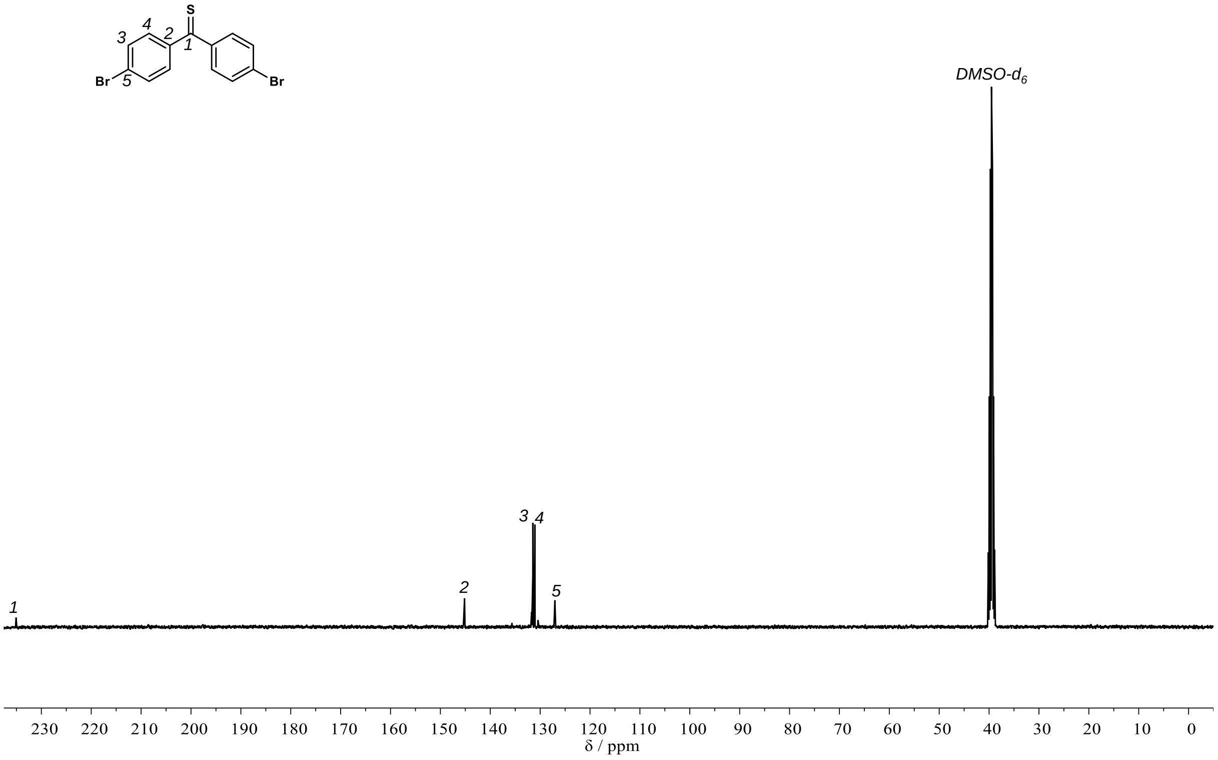

Supplementary Figure 40: ^{13}C-NMR spectrum of 5c.

1.3.4 Mechanistic intermediates

***Synthesis of 4,4'-dimethoxybenzhydrylidenehydrazine* (9)**

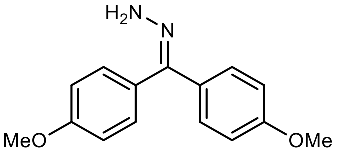

2.00 g (8.26 mmol, 1.00 eq.) 4,4'-dimethoxybenzophenone (**3a**) were suspended in 12.5 mL of ethanol and hydrazine hydrate (4.10 mL. 81.8 mmol, 9.90 eq.) was added. The mixture was refluxed for 64 hours. Afterwards, the crude reaction mixture was stored at -20 °C for 1 hour. The precipitate was filtered off, washed with cold ethanol and dried under vacuum. The product was obtained as a colorless solid in a yield of 79% (1.69 g, 6.58 mmol).

^{1}H NMR (400 MHz, DMSO-d_6) δ/ppm = 7.29 – 7.23 (m, 2H), 7.17 – 7.13 (m, 2H), 7.13 – 7.08 (m, 2H), 6.88 – 6.82 (m, 2H), 6.01 (s, 2H), 3.82 (s, 3H), 3.73 (s, 3H).

^{13}C NMR (101 MHz, DMSO-d_6) δ/ppm = 160.77, 147.02, 143.33, 136.21, 129.60, 128.35, 127.24, 126.27, 114.25, 55.27, 20.99.

IR (ATR platinum diamond): ν/cm^{-1} = 3369 (w), 3279 (w), 3215 (vw), 3194 (vw), 3059 (vw), 3013 (vw), 2964 (vw), 2841 (vw), 1606 (s), 1574 (w), 1508 (vs), 1471 (w), 1458 (m), 1442 (m), 1411 (w), 1335 (w), 1300 (w), 1288 (w), 1240 (vs), 1179 (s), 1166 (vs), 1109 (w), 1072 (w), 1022 (vs), 965 (w), 954 (w), 934 (w), 837 (vs), 827 (s), 819 (vs), 804 (m), 786 (w), 736 (w), 695 (w), 677 (m), 648 (w), 627 (w), 617 (w), 601 (s), 555 (m), 529 (w), 504 (m), 485 (w), 461 (m).

ESI-HRMS m/z: $[M+H]^+$ calculated for $C_{15}H_{16}N_2O_2$ = 257.1285, found 257.1282.

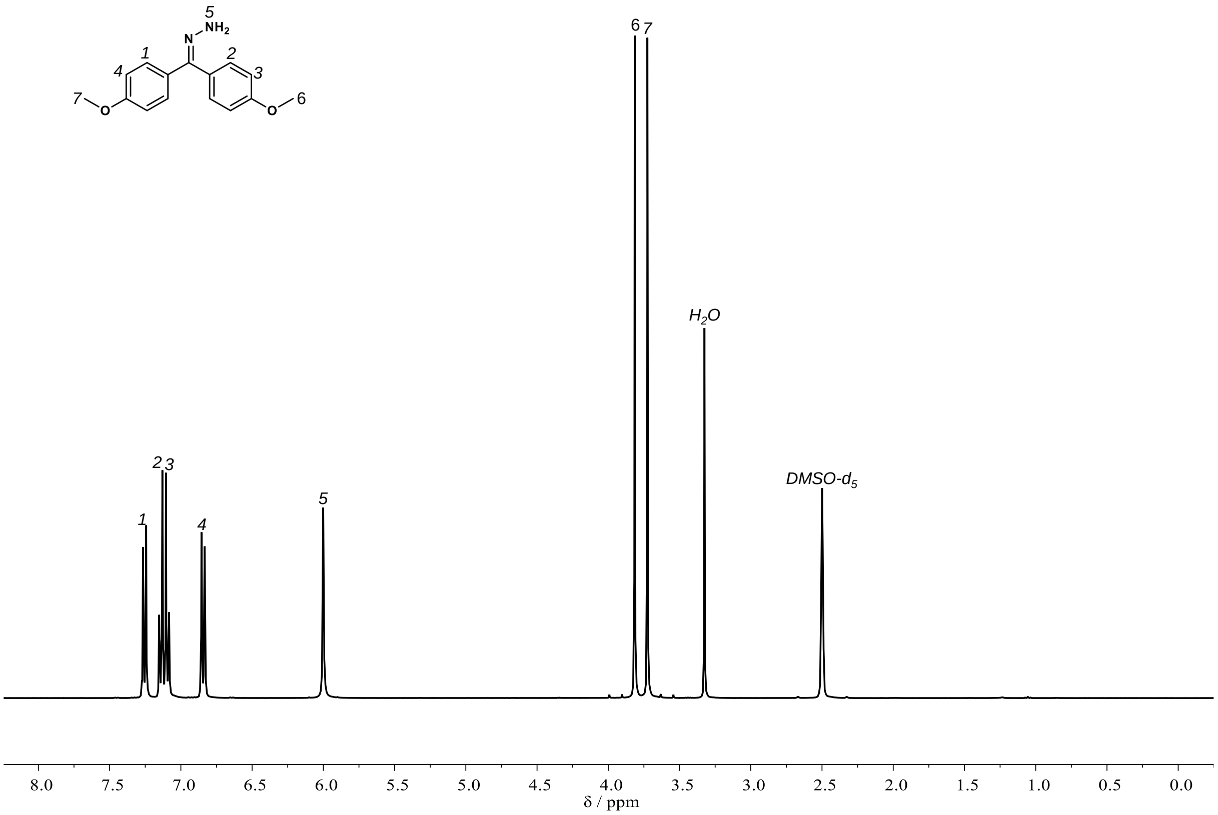

Supplementary Figure 41: ^{1}H-NMR spectrum of 9.

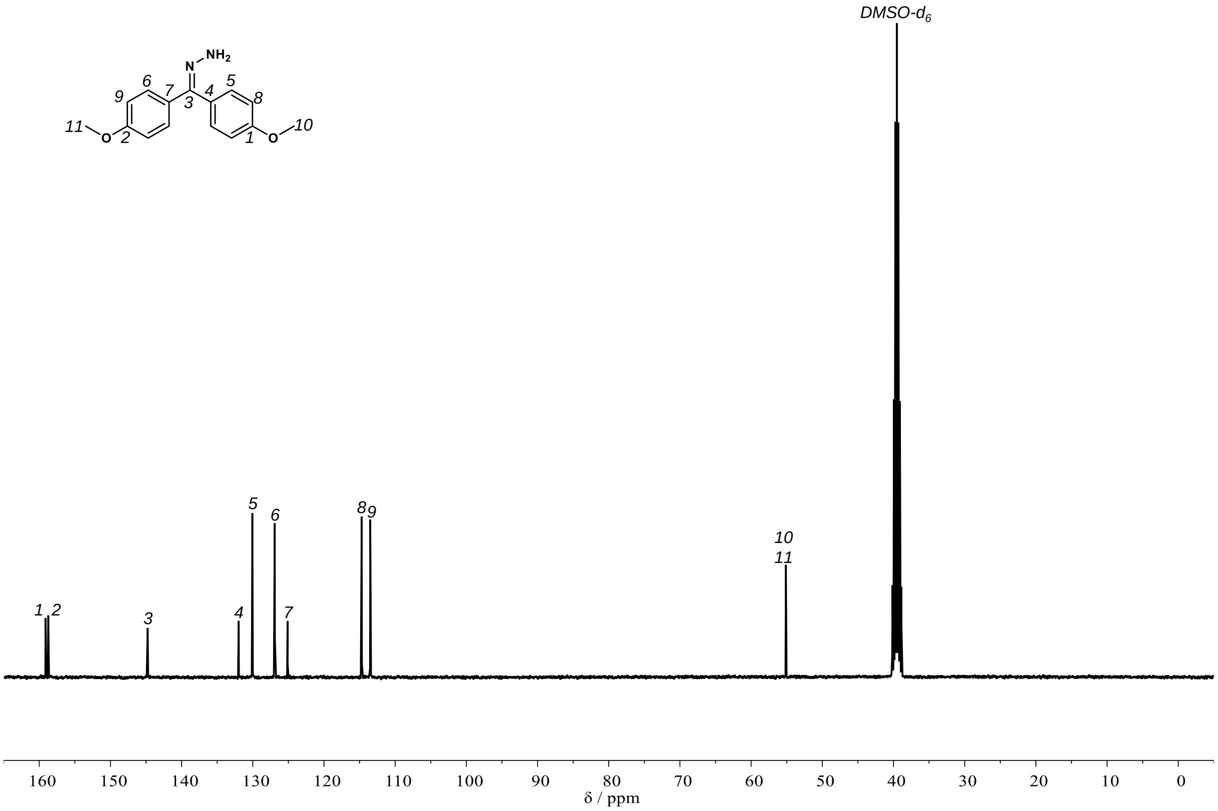

Supplementary Figure 42: ^{13}C-NMR spectrum of 9.

***Synthesis of bis(4-methoxyphenyl)diazomethane* (4a)**

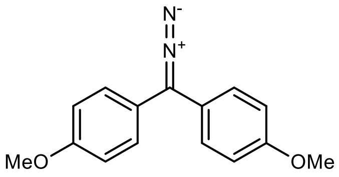

In a flame-dried Schlenk flask under argon atmosphere, 256 mg (1.00 mmol, 1.00 eq.) 4,4'-dimethoxybenzophenone hydrazine (**9**) was dissolved in 5 mL of dry THF and 217 mg (2.50 mmol, 2.50 mmol) manganese dioxide along with 142 mg (1.00 mmol, 1.00 eq.) anhydrous sodium sulfate and 100 µL of saturated ethanolic potassium hydroxide solution were added. The mixture was stirred under light exclusion for 3 hours. Afterwards, the mixture was filtere through Celite® and the solvent was removed under reduced pressure. The product was obtained as a purple crystalline solid in quantitative yield (254 mg, 1.00 mmol). Impurities in the NMR spectra are due to the instability of the compound in solution.

^{1}H NMR (400 MHz, DMSO-d_6) δ/ppm = 7.20 – 7.14 (m, 4H), 7.05 – 7.01 (m, 4H), 3.77 (s, 4H).

^{13}C NMR (101 MHz, DMSO-d_6) δ/ppm = 157.59, 126.31, 120.26, 115.10, 60.05, 55.21.

IR (ATR platinum diamond): v/cm^{-1} = 3063 (vw), 3034 (vw), 3017 (vw), 2952 (w), 2935 (w), 2904 (w), 2834 (w), 2026 (vs), 1650 (vw), 1604 (m), 1576 (w), 1506 (vs), 1465 (m), 1440 (m), 1415 (w), 1288 (w), 1267 (m), 1240 (vs), 1174 (vs), 1152 (m), 1113 (m), 1024 (vs), 961 (w), 940 (w), 930 (w), 854 (w), 827 (vs), 780 (m), 716 (w), 638 (w), 609 (s), 545 (m), 508 (w), 483 (m), 420 (w).

Mass spectrometry was not conclusive due to the instability of the compound.

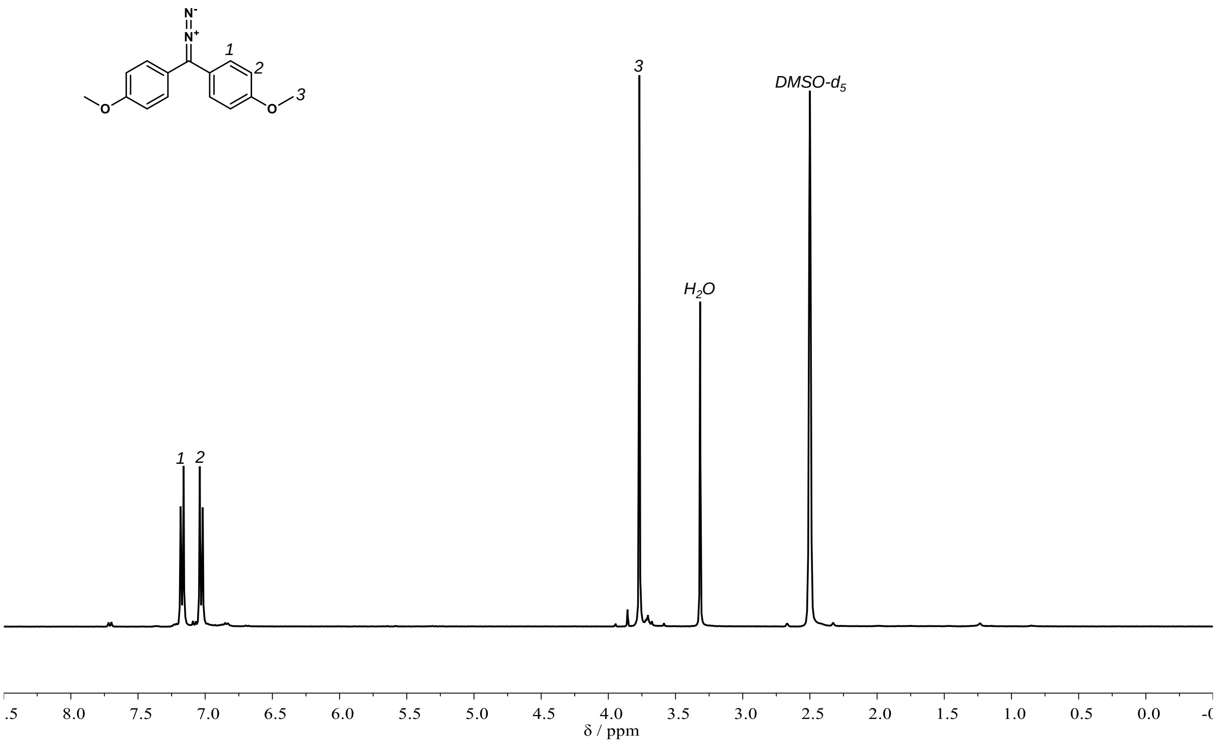

Supplementary Figure 43: ^{1}H-NMR spectrum of 4a.

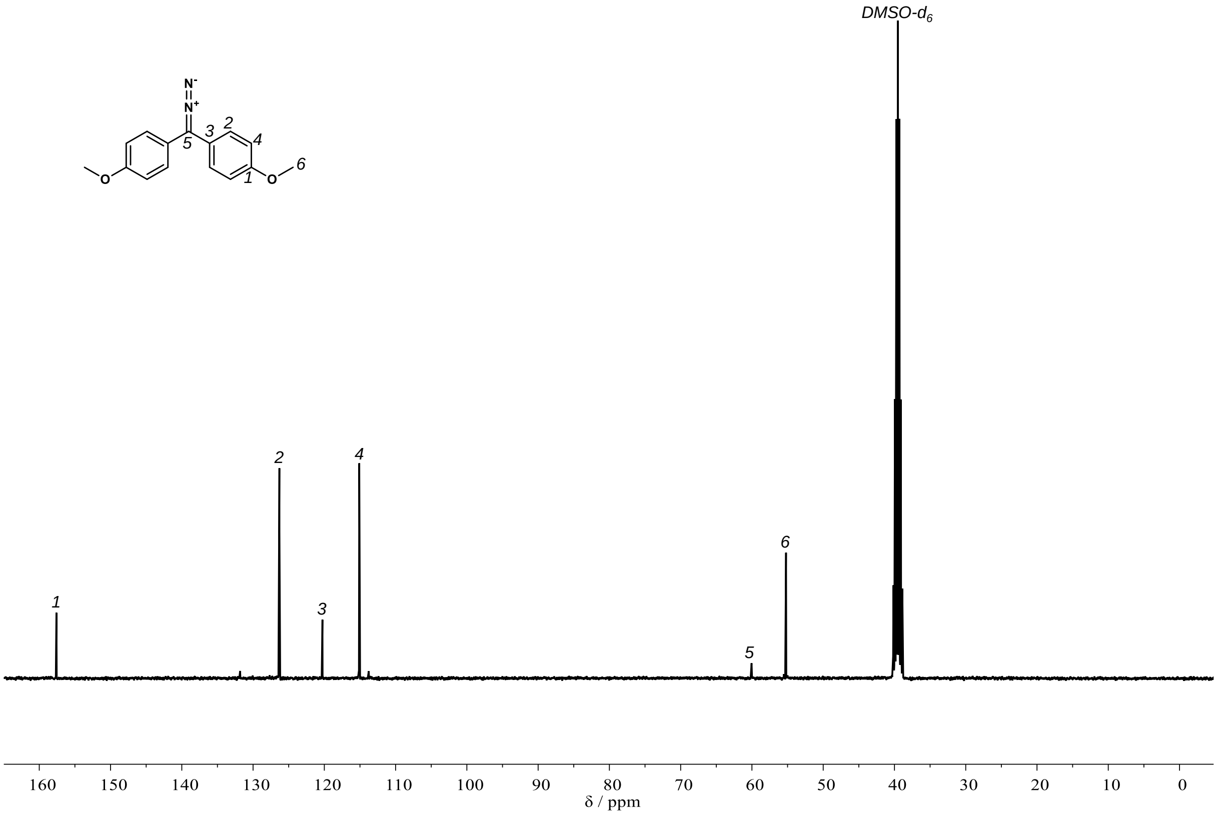

Supplementary Figure 44: ^{13}C-NMR spectrum of 4a.

***Synthesis of 2,2,3,3-tetrakis(4-methoxyphenyl)thiirane* (6a)**

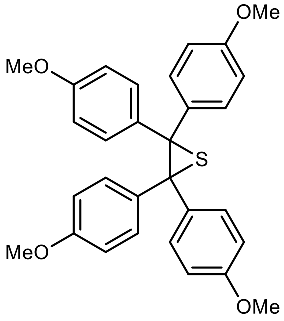

In a round-bottom flask, 264 mg (70 wt% NMR purity, 0.726 mg, 1.00 eq.) bis(4-methoxyphenyl)diazomethane (**4a**) were dissolved in 5 mL of dry DMSO and a solution of 188 mg (0.726 mmol, 1.00 eq.) 4,4'-dimethoxythiobenzophenone (**5a**) was added dropwise. The mixture was stirred for 5 minutes at room temperature. Afterwards, 20 mL of diethyl ether were added and the precipitate was filtered off, washed with diethyl ether and dried under vacuum. The product was obtained as a colorless solid in a yield of 76% (268 mg, 0.554 mmol).

^{1}H NMR (400 MHz, DMSO-d_6) δ/ppm = 7.17 – 7.08 (m, 8H), 6.69 – 6.60 (m, 8H), 3.65 (s, 12H).

^{13}C NMR (101 MHz, DMSO-d_6) δ/ppm = 157.63, 132.70, 131.56, 112.50, 66.93, 54.95.

IR (ATR platinum diamond): v/cm^{-1} = 3071 (vw), 3030 (vw), 3011 (vw), 2948 (vw), 2927 (vw), 2896 (vw), 2834 (w), 1604 (m), 1576 (w), 1504 (vs), 1460 (s), 1440 (w), 1411 (w), 1294 (m), 1242 (vs), 1177 (s), 1166 (s), 1113 (m), 1031 (vs), 1008 (w), 874 (vw), 839 (m), 825 (s), 812 (vs), 786 (w), 767 (m), 753 (w), 732 (w), 699 (w), 687 (w), 621 (w), 586 (s), 553 (s), 522 (m) cm-1

EI-HRMS m/z: $[M]^+$ calculated for $C_{30}H_{28}O_4S$ = 484.1703, found 484.1701

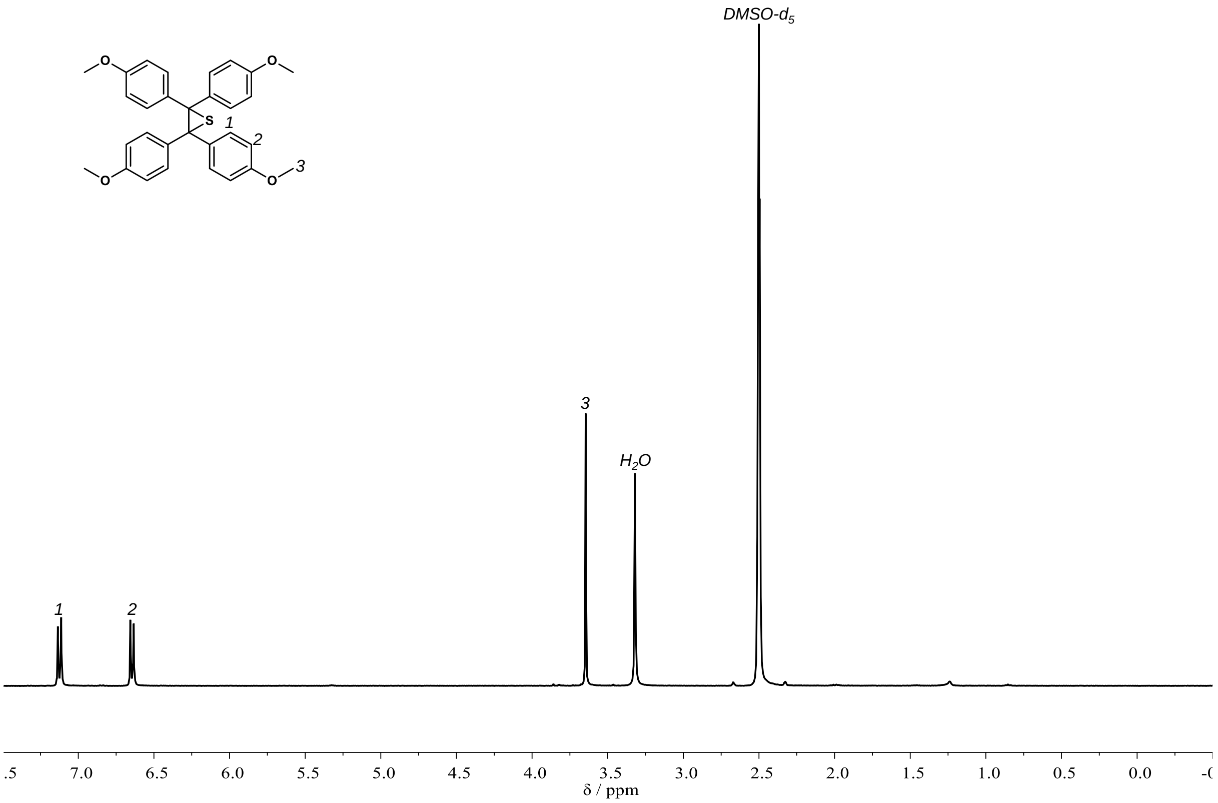

Supplementary Figure 45: ^{1}H-NMR spectrum of 6a.

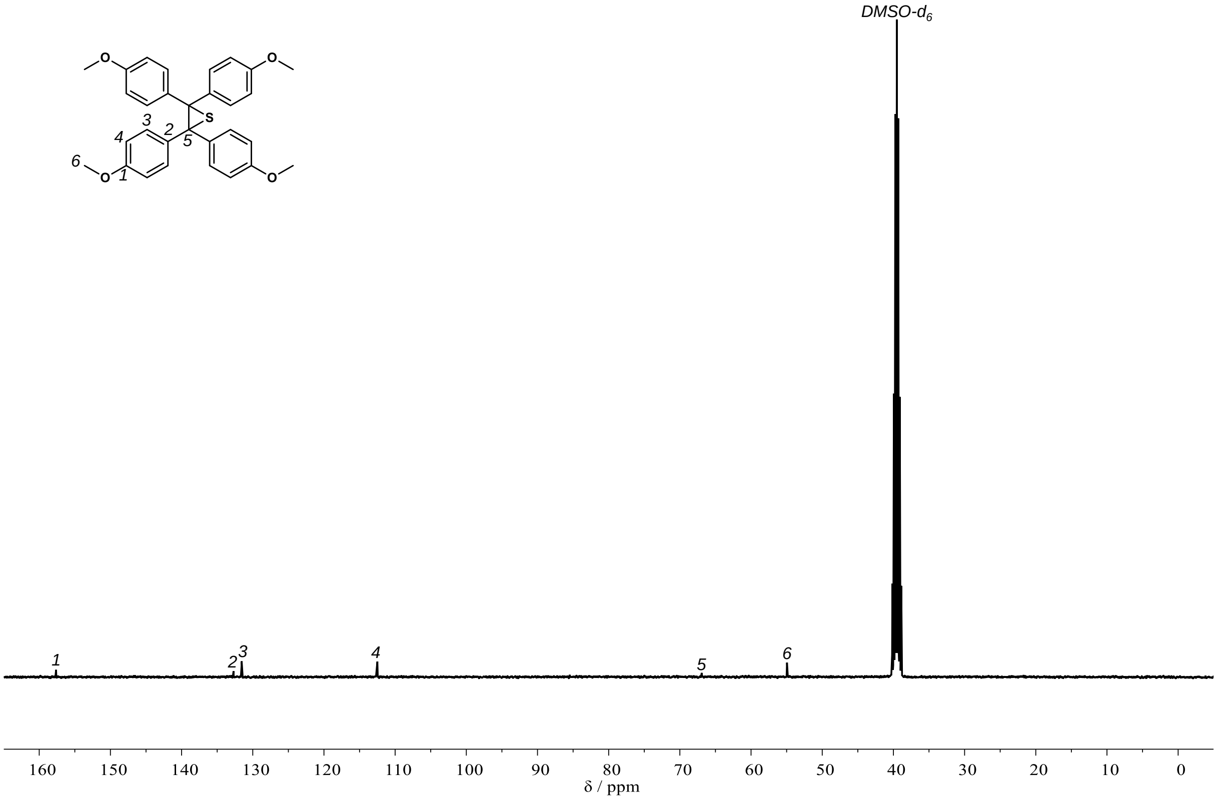

Supplementary Figure 46: ^{13}C-NMR spectrum of 6a.

1.3.5 Homocoupling products

General procedure for alkylidene-homocouplings of N-tosylhydrazones:

1.00 mmol of the *N*-tosylhydrazone was dispersed in 2.00 mL of dry DMSO and 64.1 mg of elemental sulfur (0.25 mmol, 0.25 eq.) was added. The mixture was heated to 100°C and 166 mg of anhydrous potassium carbonate (1.20 mmol, 1.20 eq.) were added. The mixture was stirred at 100°C for 30 minutes. Afterwards, the crude mixture was transferred directly onto a column (packed in pure cyclohexane) and the product was isolated *via* column chromatography.

***Synthesis of 1,1,2,2-tetrakis(4-methoxyphenyl)ethylene* (2a)**

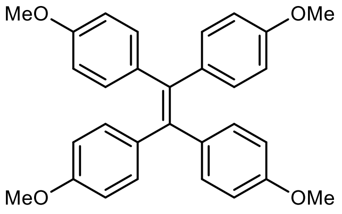

1. Prepared from **1a** according to the general procedure. The product was isolated *via* column chromatography (cyclohexane/ethyl acetate 30:1) as a colorless solid in a yield of 99% (225 mg, 0.497 mmol)

2. Upscaled synthesis:
Prepared from **1a** according to the general procedure in a larger scale of 5 mmol NTH. The product was isolated *via* column chromatography (cyclohexane/ethyl acetate 30:1) as a colorless solid in a yield of 95% (1.08 g, 2.38 mmol).

3. One-Pot synthesis:
485 mg 4,4'-dimethoxybenzophenone (**3a**) (2.00 mmol, 1.00 eq.) was dispersed in 4 mL of ethanol and 373 mg *p*-toluenesulfonyl hydrazide (2.00 mmol, 1.00 eq.) along with 19.0 mg *p*-toluenesulfonic acid monohydrate (0.10 mmol, 0.05 eq.) were added. The mixture was refluxed for 12 hours. The solvent was removed *in vacuo* and the residue was dispersed in 4 mL of dry DMSO. 128 mg of elemental sulfur (0.50 mmol, 0.25 eq.) were added and the mixture was heated to 100°C. 346 mg of potassium carbonate (2.50 mmol, 1.25 eq.) were added and the mixture was stirred at 100°C for 30 minutes. The crude mixture was transferred to a column (packed in cyclohexane) and the product was isolated *via* column chromatography (cyclohexane/ethyl acetate 30:1). The product was obtained as a colorless solid in a yield of 85% (385 mg, 0.851 mmol).

^{1}H NMR (400 MHz, DMSO-d_6) δ/ppm = 6.89 – 6.80 (m, 8H), 6.73 – 6.65 (m, 8H), 3.68 (s, 12H).

^{13}C NMR (101 MHz, DMSO-d_6) δ/ppm = 157.43, 137.98, 136.24, 131.97, 113.19, 54.88.

IR (ATR platinum diamond): v/cm^{-1} = 3052 (vw), 3003 (vw), 2952 (vw), 2896 (vw), 2830 (vw), 1604 (m), 1571 (w), 1506 (vs), 1454 (m), 1440 (m), 1411 (w), 1296 (s), 1271 (w), 1240 (vs), 1183 (w), 1168 (vs), 1105 (m), 1033 (vs), 1012 (m), 977 (w), 862 (w), 827 (vs), 808 (vs), 765 (m), 745 (w), 588 (vs), 568 (w), 525 (m).

ESI-HRMS m/z: [M]$^+$ calculated for $C_{30}H_{28}O_4$ = 452.1982, found 452.1980.

Fluorescence: λ_{em}(max) = 490 nm with λ_{ex} = 327 nm

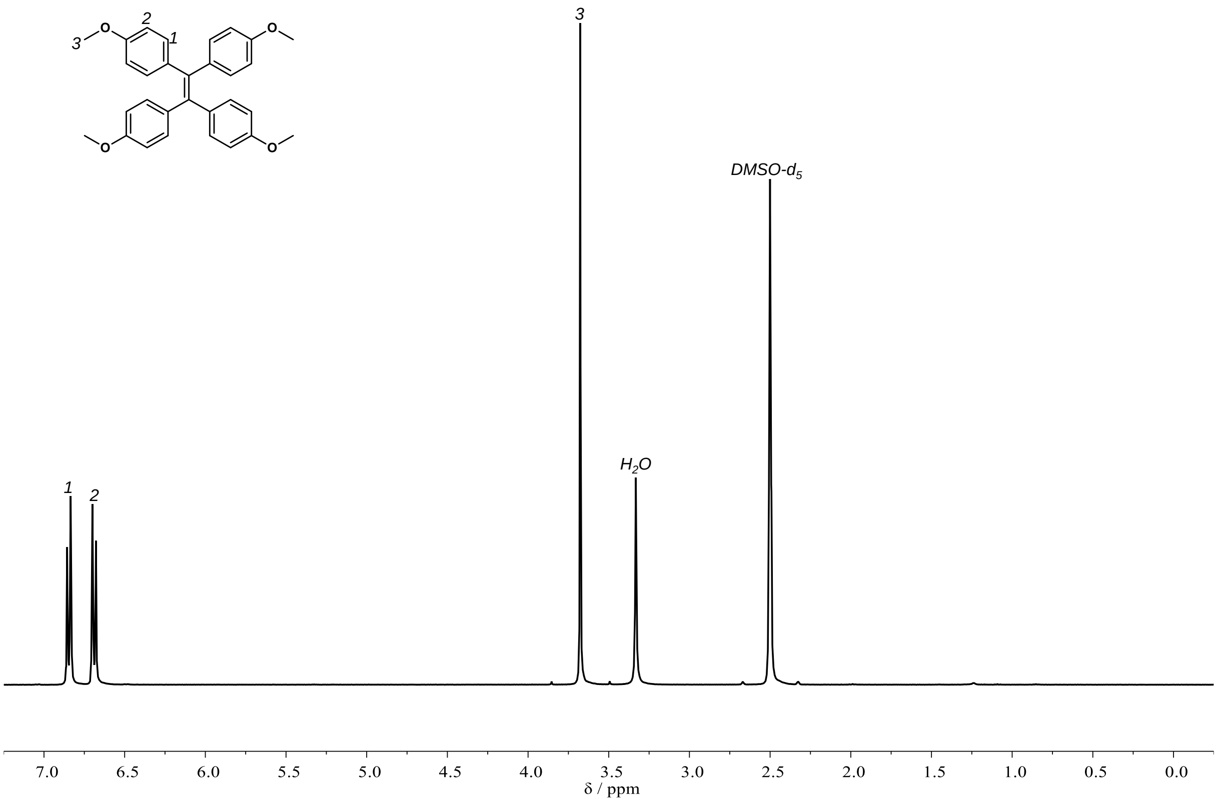

Supplementary Figure 47: ^{1}H-NMR spectrum of 2a.

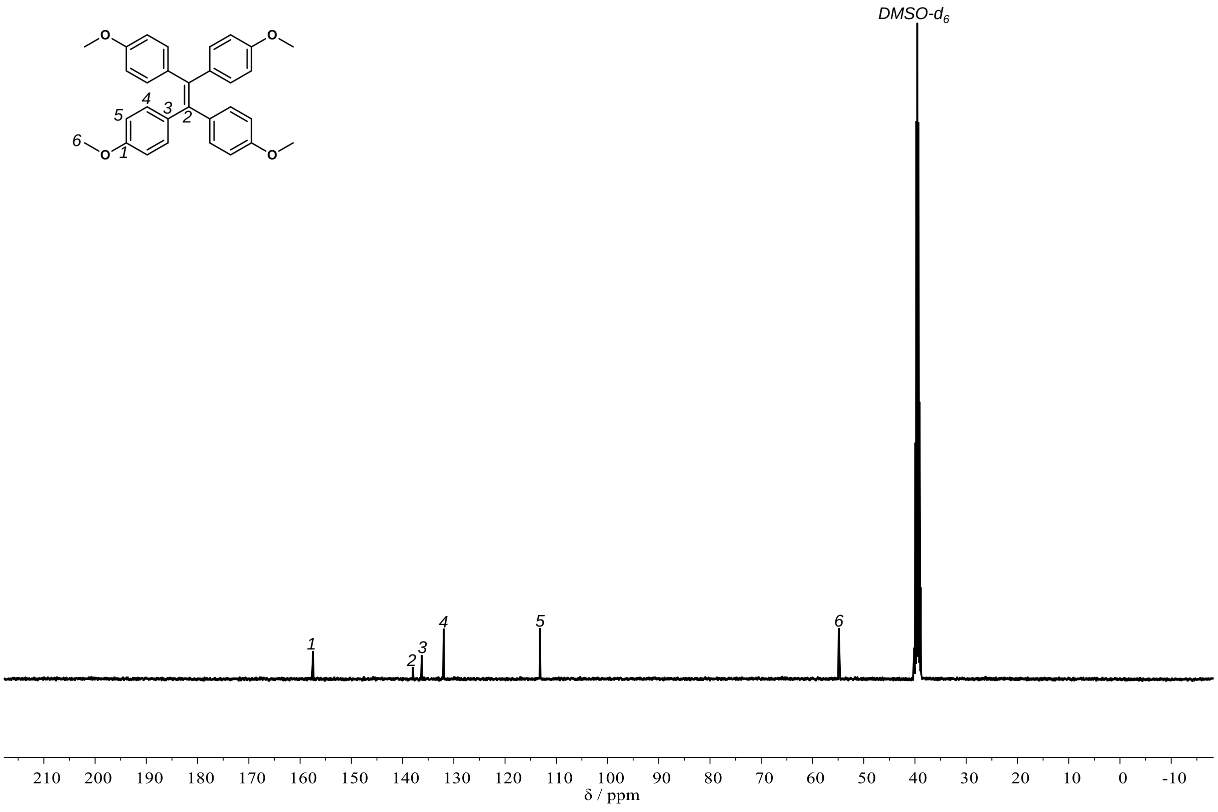

Supplementary Figure 48: ^{13}C-NMR spectrum of 2a.

***Synthesis of 1,1,2,2-tetraphenylethylene* (2b)**

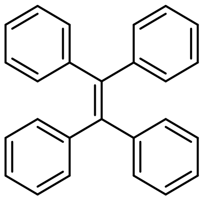

Prepared from **1b** according to the general procedure. The product was isolated *via* column chromatography (cyclohexane/ethyl acetate 50:1) as a colorless solid in a yield of 99% (164 mg, 0.493 mmol).

^{1}H NMR (400 MHz, DMSO-d_6) δ/ppm = 7.18 – 7.05 (m, 12H), 7.00 – 6.93 (m, 8H).

^{13}C NMR (101 MHz, DMSO-d_6) δ/ppm = 143.14, 140.55, 130.60, 127.81, 126.52.

IR (ATR platinum diamond): v/cm^{-1} = 3075 (vw), 3050 (vw), 3022 (vw), 2923 (vw), 2855 (vw), 1594 (w), 1574 (vw), 1489 (w), 1442 (m), 1275 (vw), 1181 (vw), 1154 (w), 1074 (w), 1026 (w), 1000 (w), 981 (w), 919 (vw), 913 (w), 909 (w), 778 (w), 761 (m), 745 (s), 693 (vs), 625 (s), 613 (m), 568 (m), 471 (w), 465 (w), 440 (vw).

EI-HRMS m/z: [M]$^+$ calculated for $C_{26}H_{20}$ = 332.1560, found 332.1557.

Fluorescence: λ_{em}(max) = 469 nm with λ_{ex} = 309 nm

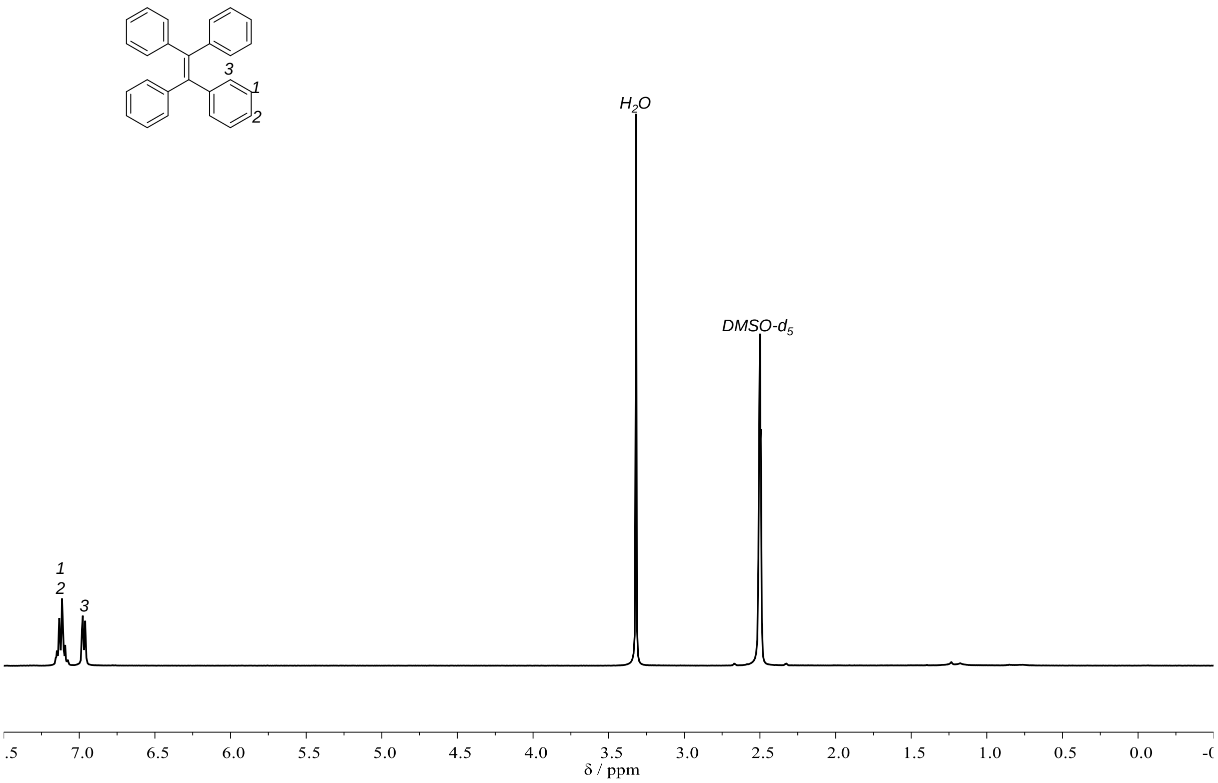

Supplementary Figure 49: ^{1}H-NMR spectrum of 2b.

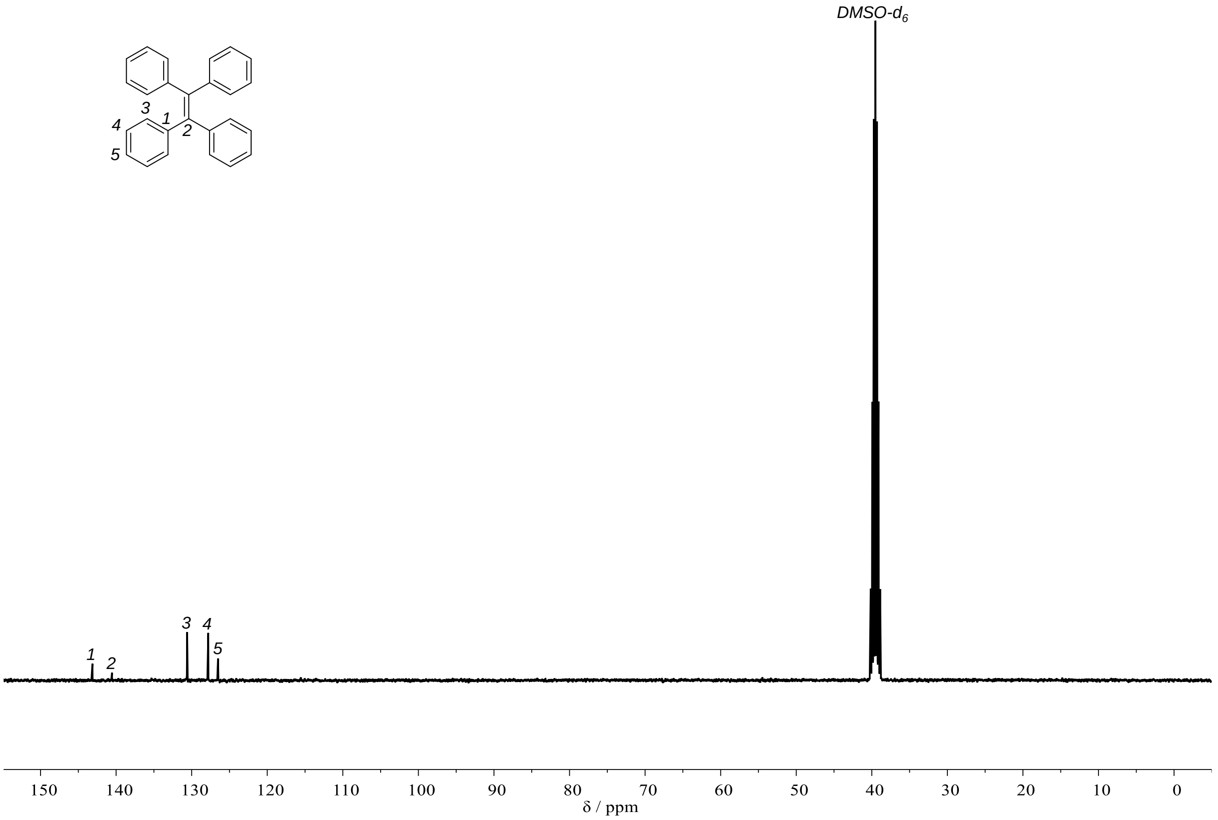

Supplementary Figure 50: ^{13}C-NMR spectrum of 2b.

***Synthesis of 1,1,2,2-tetrakis(4-bromophenyl)ethylene* (2c)**

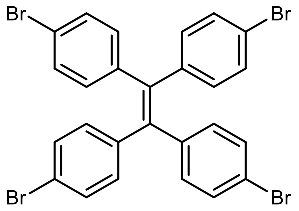

Prepared from **1c** according to the general procedure. The product was isolated *via* column chromatography (cyclohexane/ethyl acetate 50:1) as a colorless solid in a yield of 95% (307 mg, 0.474 mmol).

^{1}H NMR (400 MHz, DMSO-d_6) δ/ppm = 7.43 – 7.35 (m, 8H), 6.96 – 6.87 (m, 8H).

^{13}C NMR (101 MHz, DMSO-d_6) δ/ppm = 141.44, 139.32, 132.73, 131.13, 120.42.

IR (ATR platinum diamond): v/cm^{-1} = 1582 (w), 1483 (vs), 1393 (m), 1103 (w), 1070 (s), 1008 (vs), 973 (w), 963 (w), 862 (w), 817 (s), 792 (vs), 736 (s), 716 (w), 685 (w), 652 (w), 631 (w), 623 (w), 566 (w), 502 (s), 490 (s), 477 (m).

ESI-HRMS m/z: [M]$^+$ calculated for $C_{26}H_{16}{}^{79}Br_2{}^{81}Br_2$ = 647.7939, found 647.7936.

Fluorescence: λ_{em}(max) = 476 nm with λ_{ex} = 321 nm

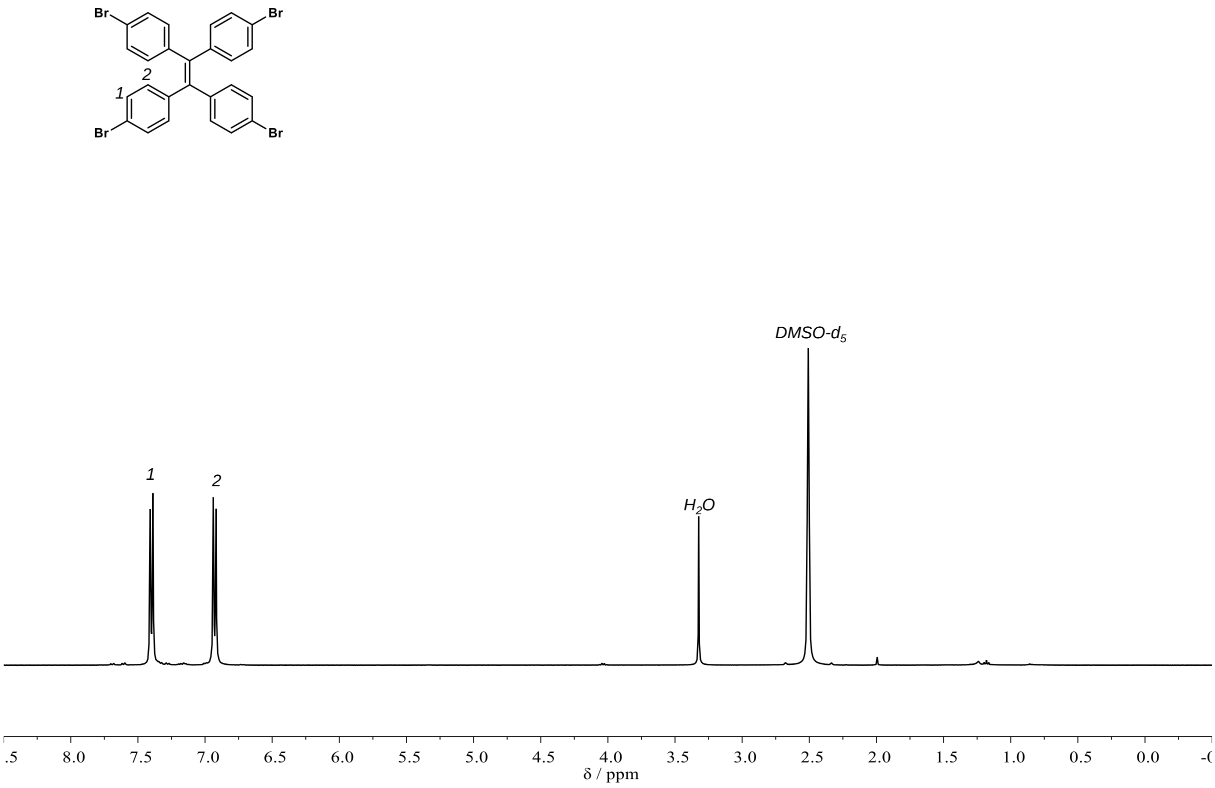

Supplementary Figure 51: ^{1}H-NMR spectrum of 2c.

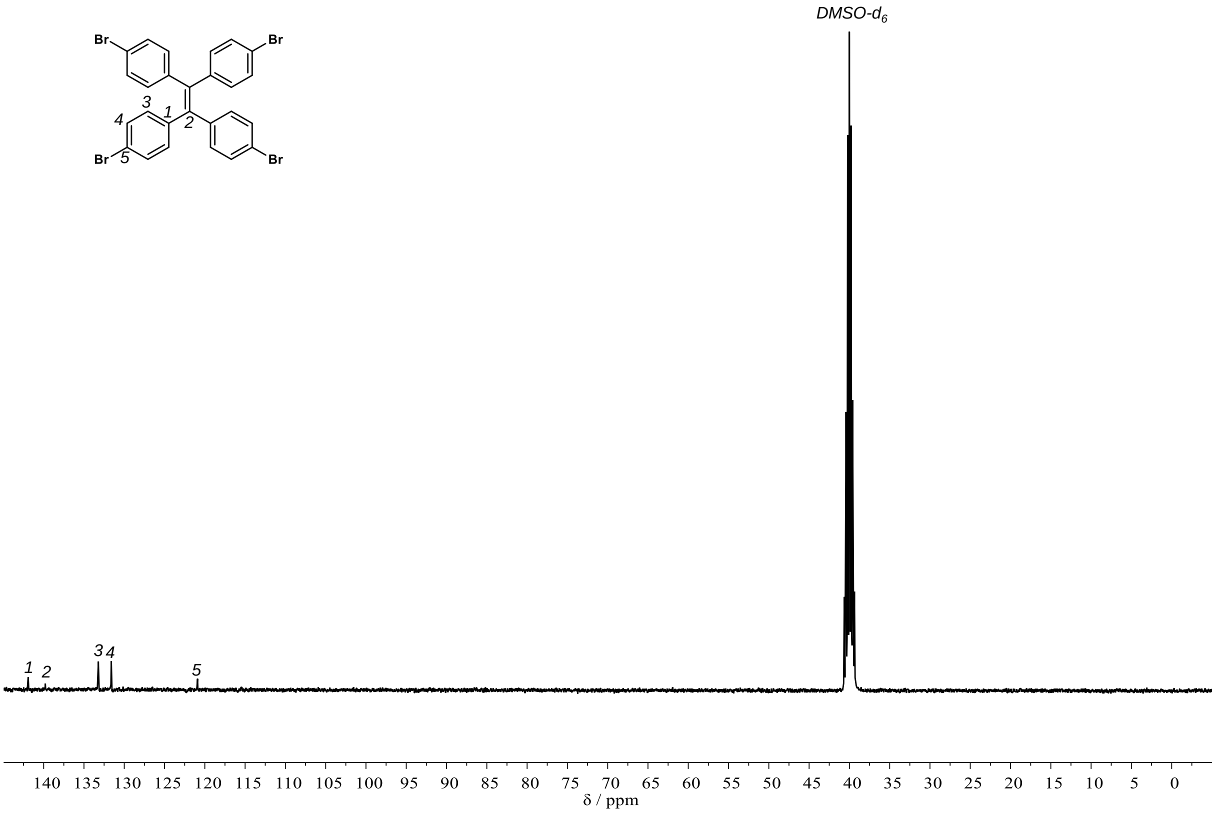

Supplementary Figure 52: ^{13}C-NMR spectrum of 2c.

Synthesis of 1,1,2,2-tetrakis(4-nitrophenyl)ethylene **(2d)**

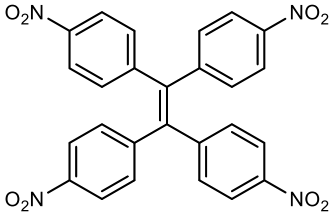

Prepared from **1d** according to the general procedure. The product was isolated *via* column chromatography (cyclohexane/ethyl acetate 10:1) as an orange solid in a yield of 83% (212 mg, 414 mmol).

^{1}H NMR (400 MHz, DMSO-d_6) δ/ppm = 8.36 – 7.98 (m, 8H), 7.54 – 7.26 (m, 8H).

^{13}C NMR (101 MHz, DMSO-d_6) δ/ppm = 148.08, 147.06, 141.29, 132.53, 124.06.

IR (ATR platinum diamond): v/cm^{-1} = 3106 (vw), 2933 (vw), 2849 (vw), 1594 (w), 1512 (vs), 1407 (vw), 1341 (vs), 1281 (w), 1273 (w), 1179 (w), 1107 (w), 1014 (w), 880 (w), 854 (w), 847 (w), 835 (s), 810 (m), 747 (s), 720 (w), 703 (s), 689 (w), 650 (w), 504 (w), 487 (w), 471 (w).

FAB-HRMS m/z: $[M+H]^+$ calculated for $C_{26}H_{16}N_4O_8$ = 513.1040, found 513.1041.

Fluorescence: λ_{em}(max) = 509 nm with λ_{ex} = 325 nm.

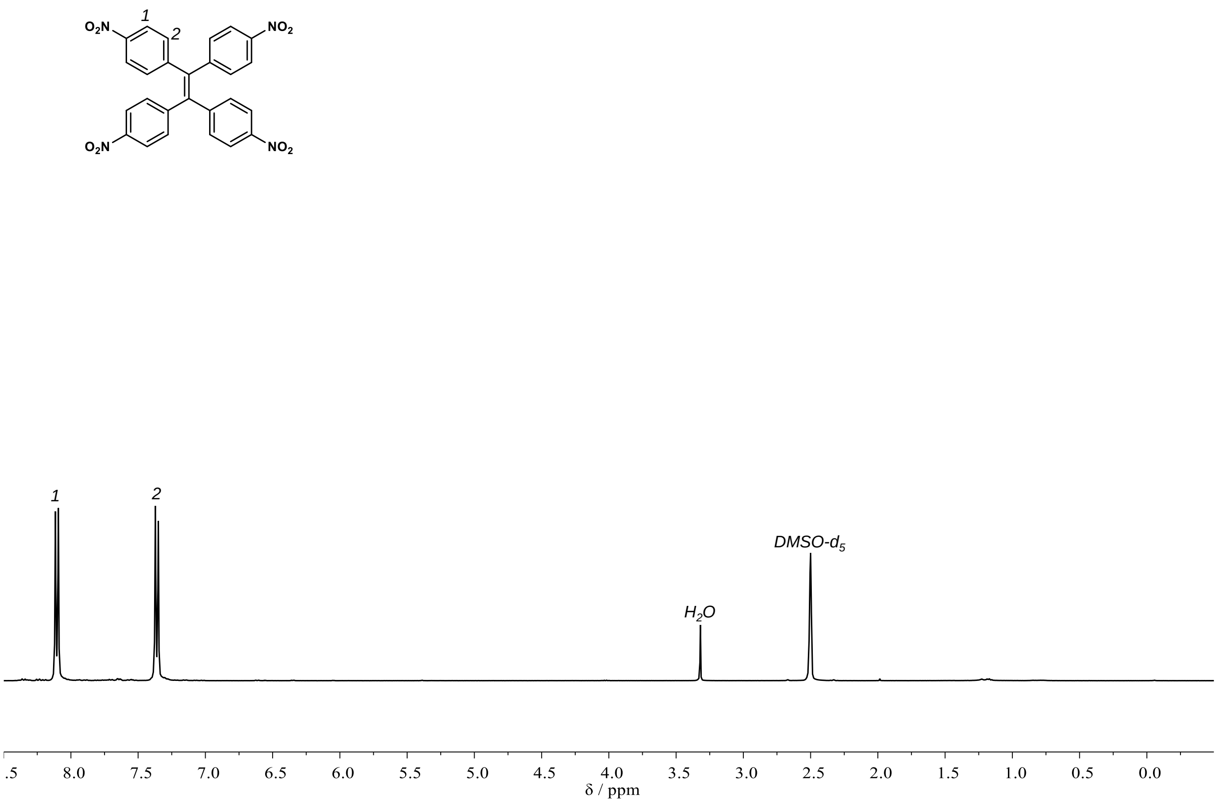

Supplementary Figure 53: ^{1}H-NMR spectrum of 2d.

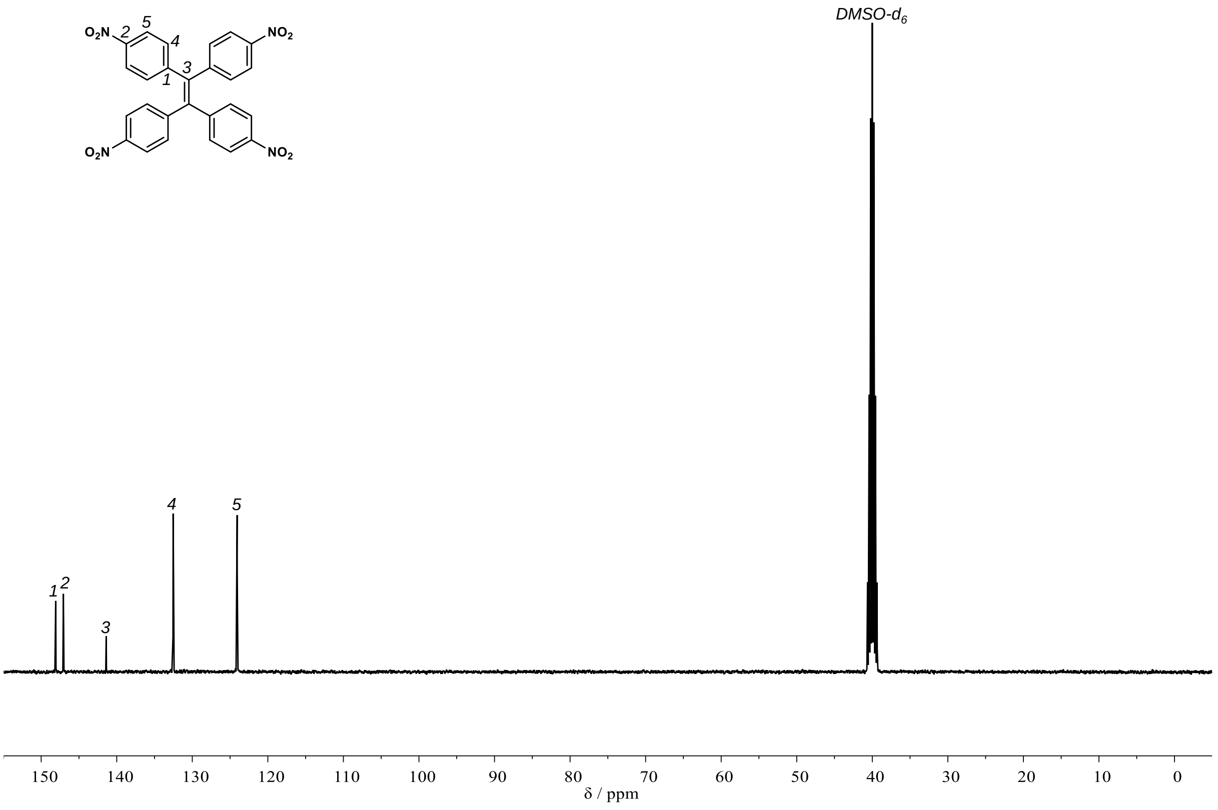

Supplementary Figure 54: ^{13}C-NMR spectrum of 2d.

***Synthesis of 9,9'-bifluorenylidene* (2e)**

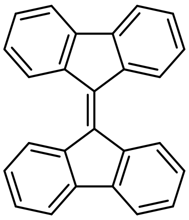

Prepared from **1e** according to the general procedure. The product was isolated *via* column chromatography (cyclohexane/ethyl acetate 50:1) as a red solid in a yield of 83% (137 mg, 0.417 mmol).

^{1}H NMR (400 MHz, DMSO-d_6) δ/ppm = 8.33 – 8.26 (m, 4H), 7.94 – 7.87 (m, 4H), 7.45 – 7.34 (m, 4H), 7.34 – 7.25 (m, 4H).

^{13}C NMR (101 MHz, DMSO-d_6) δ/ppm = 140.72, 140.21, 137.18, 129.71, 127.31, 126.21, 120.48.

IR (ATR platinum diamond): *v*/cm^{-1} = 3077 (vw), 3052 (vw), 3030 (vw), 3013 (vw), 1604 (w), 1571 (vw), 1475 (w), 1440 (m), 1347 (w), 1310 (w), 1304 (w), 1279 (w), 1230 (w), 1205 (w), 1150 (w), 1100 (w), 1035 (w), 938 (w), 874 (w), 786 (w), 761 (s), 743 (m), 718 (vs), 638 (w), 617 (w), 586 (w), 422 (w).

EI-HRMS m/z: [M]$^{+}$ calculated for $C_{26}H_{16}$ = 328.1247, found 328.1245.

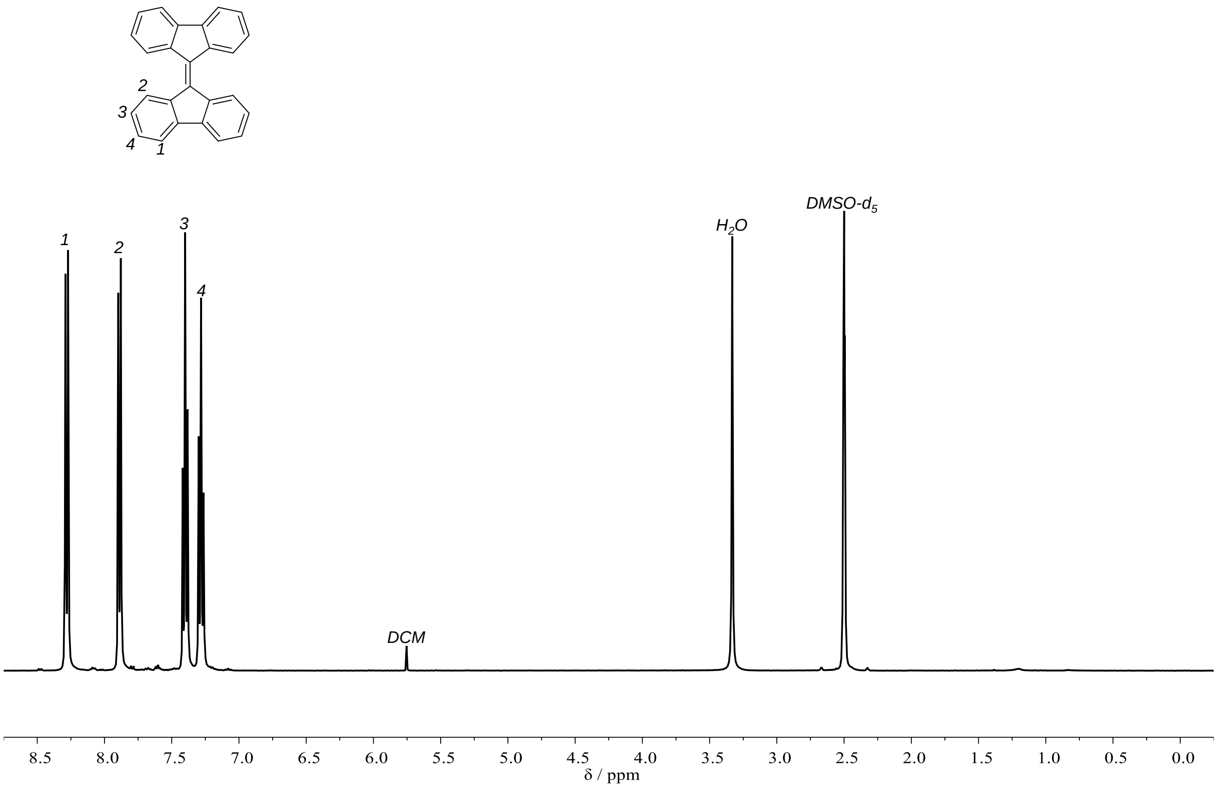

Supplementary Figure 55: ^{1}H-NMR spectrum of 2e.

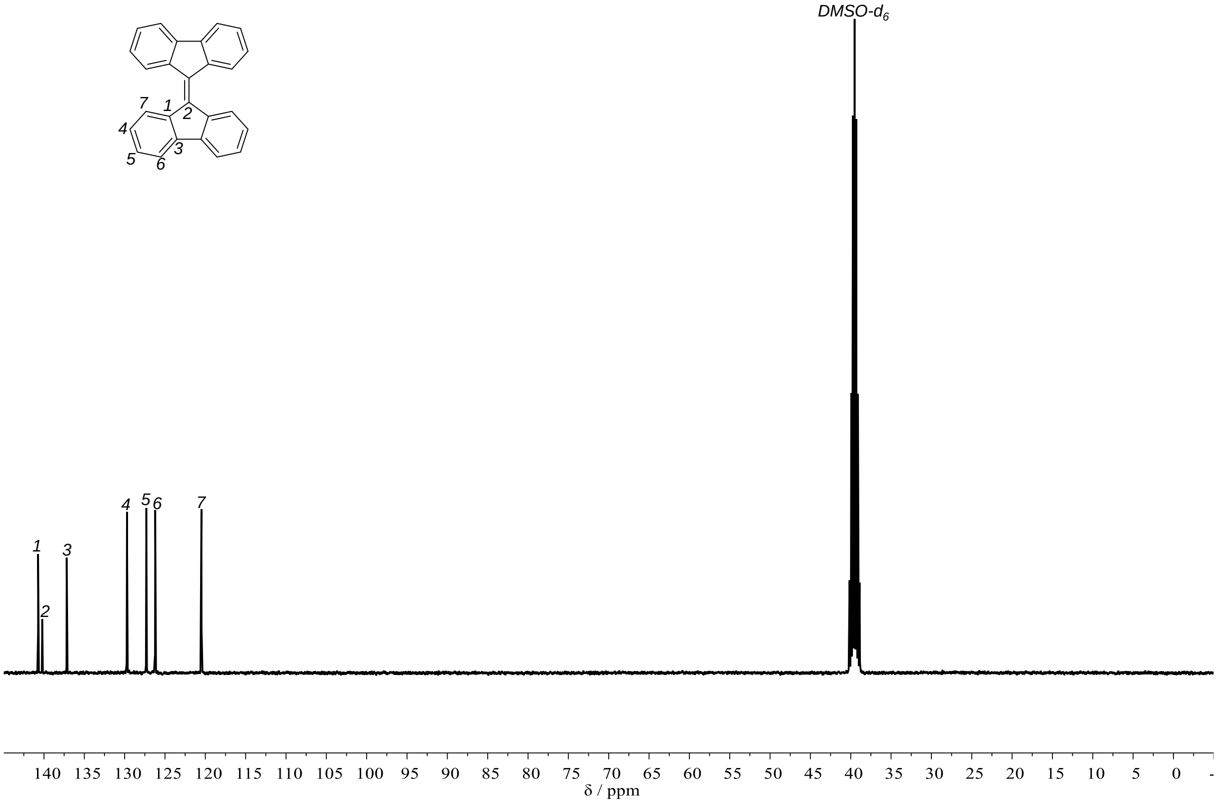

Supplementary Figure 56: ^{13}C-NMR spectrum of 2e.

***Synthesis of 1,2-bis(4-methoxyphenyl)-1,2-diphenylethylene* (2f)**

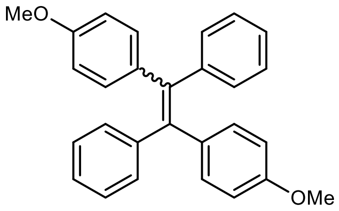

Prepared from **1f** according to the general procedure. The product was isolated *via* column chromatography (cyclohexane/ethyl acetate 30:1) as a colorless solid in a yield of 83% (163 mg, 0.415 mmol). According to ^{1}H-NMR, the isolated products consists of a 55:45 mixture of the *(E)*- and *(Z)*-isomers. With the employed methods, assignment of the signals to respective isomers was not possible.

^{1}H NMR (400 MHz, DMSO-d_6) δ/ppm = 7.16 – 7.04 (m, 6H), 7.01 – 6.91 (m, 4H), 6.91 – 6.80 (m, 4H), 6.75 – 6.63 (m, 4H), 3.68 (s, 6H, isomer 1), 3.66 (s, 6H, isomer 2).

^{13}C NMR (101 MHz, DMSO-d_6) δ/ppm = 157.61, 157.58, 143.79, 143.73, 139.28, 135.68, 135.62, 131.91, 131.89, 130.74, 130.71, 127.80, 127.70, 126.32, 126.26, 113.28, 113.18, 54.90, 54.88.

IR (ATR platinum diamond): ν/cm^{-1} = 3075 (vw), 3034 (vw), 3013 (vw), 2993 (vw), 2950 (w), 2929 (w), 2908 (vw), 2834 (w), 1746 (vw), 1606 (m), 1574 (w), 1508 (vs), 1462 (m), 1440 (m), 1411 (w), 1290 (m), 1242 (vs), 1172 (vs), 1133 (w), 1107 (m), 1072 (w), 1035 (vs), 975 (w), 852 (w), 843 (w), 833 (m), 819 (m), 806 (s), 786 (w), 780 (m), 757 (s), 738 (m), 695 (vs), 660 (vw), 627 (w), 607 (s), 584 (w), 570 (w), 545 (s), 516 (w), 494 (w).

ESI-HRMS m/z: [M]$^{+}$ calculated for $C_{28}H_{24}O_2$ = 392.1771, found 392.1764.

Fluorescence: λ_{em}(max) = 486 nm with λ_{ex} = 321 nm

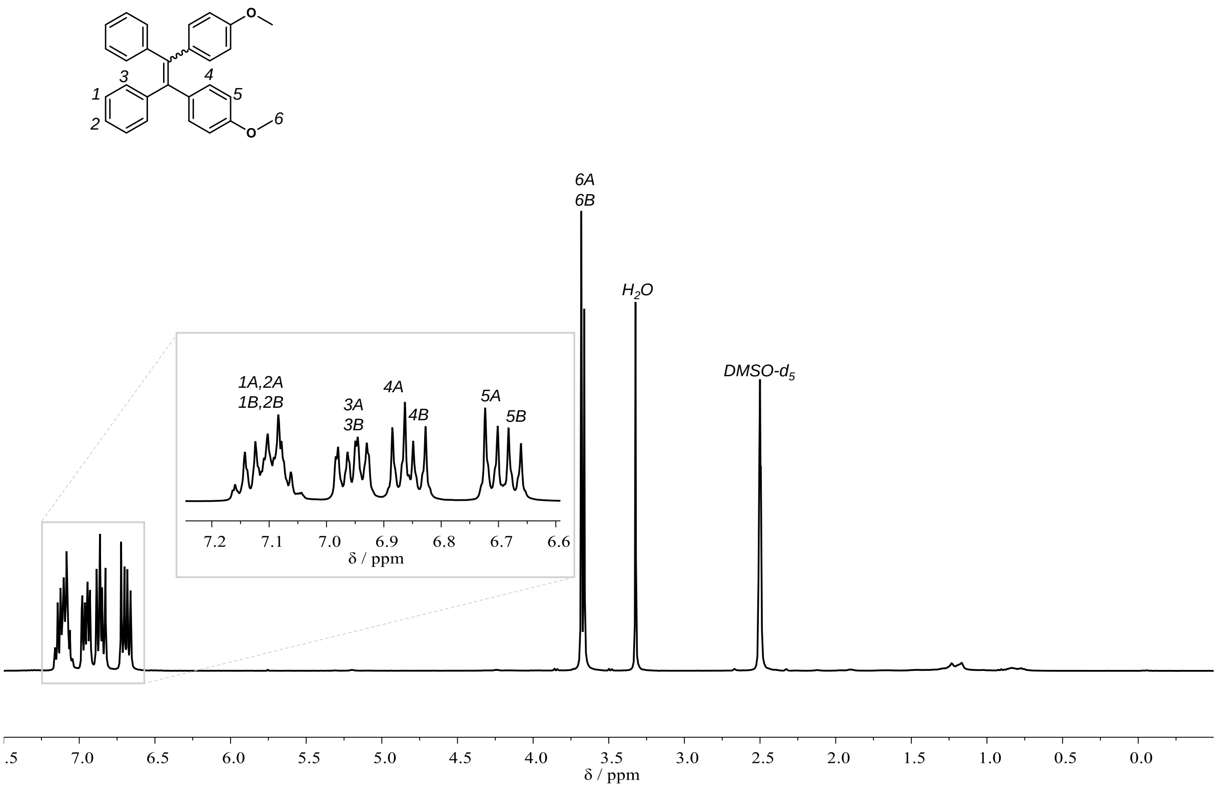

Supplementary Figure 57: ^{1}H-NMR spectrum of 2f.

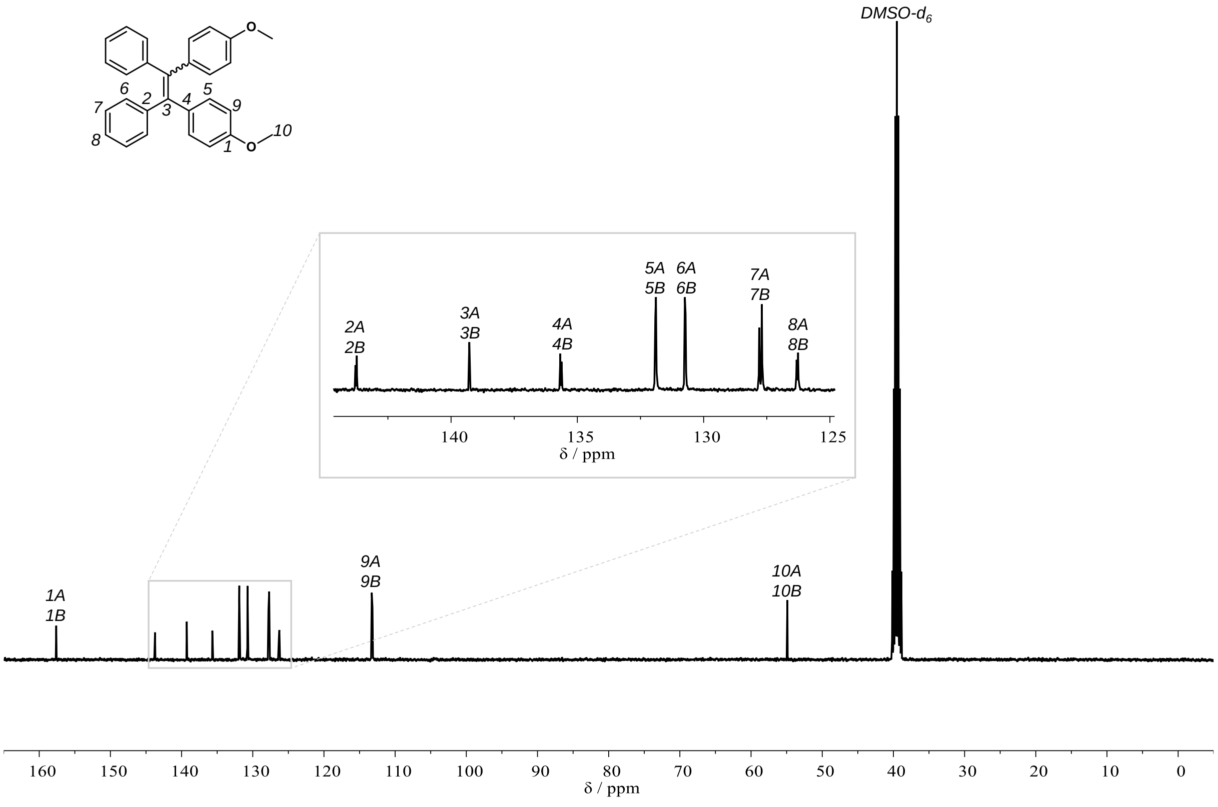

Supplementary Figure 58: ^{13}C-NMR spectrum of 2f.

Synthesis of 1,2-bis(4-bromophenyl)-1,2-diphenylethylene (2g)

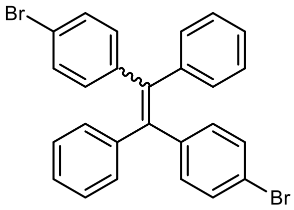

Prepared from **1g** according to the general procedure. The product was isolated *via* column chromatography (cyclohexane/ethyl acetate 50:1) as a colorless solid in a yield of 85% (208 mg, 0.424 mmol). According to ^{1}H-NMR, the isolated products consists of a 50:50 mixture of the *(E)*- and *(Z)*-isomers. With the employed methods, assignment of the signals to respective isomers was not possible.

^{1}H NMR (400 MHz, DMSO-d_6) δ/ppm = 7.42 – 7.29 (m, 4H), 7.23 – 7.07 (m, 6H), 7.02 – 6.86 (m, 8H).
^{13}C NMR (101 MHz, DMSO-d_6) δ/ppm = 142.93, 142.83, 142.67, 142.60, 140.46, 140.42, 133.23, 133.16, 131.48, 131.31, 131.10, 131.04, 128.60, 128.43, 127.49, 127.36, 120.53, 120.40, 40.68.
IR (ATR platinum diamond): ν/cm^{-1} = 3061 (vw), 3020 (vw), 2921 (vw), 1580 (vw), 1485 (s), 1442 (w), 1390 (w), 1349 (vw), 1177 (vw), 1103 (w), 1070 (s), 1028 (w), 1010 (vs), 971 (w), 915 (vw), 850 (w), 831 (w), 821 (m), 806 (s), 755 (vs), 730 (w), 716 (w), 697 (vs), 621 (w), 590 (w), 572 (w), 483 (m), 469 (w), 461 (w).
ESI-HRMS m/z: $[M]^+$ calculated for $C_{26}H_{18}{}^{79}Br^{81}Br$ = 489.9749, found 489.9743.
Fluorescence: λ_{em}(max) = 477 nm with λ_{ex} = 315 nm

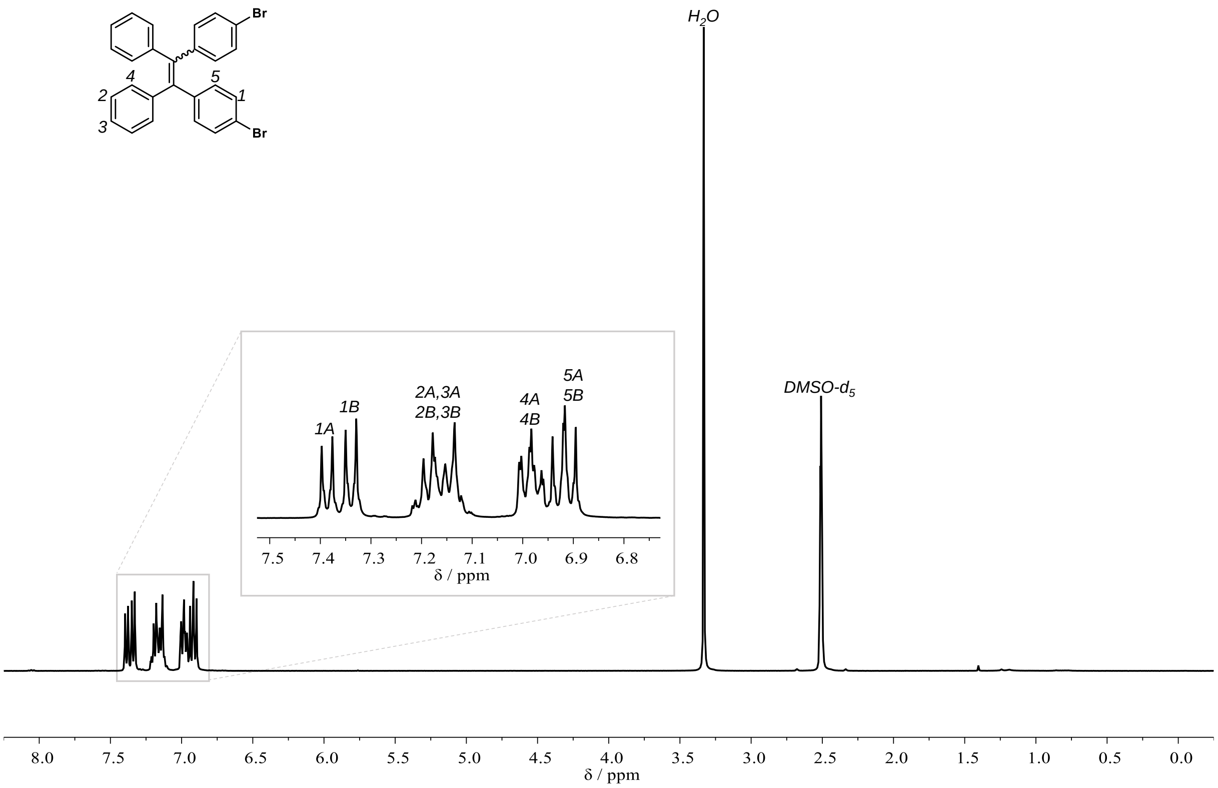

Supplementary Figure 59: ^{1}H-NMR spectrum of 2g.

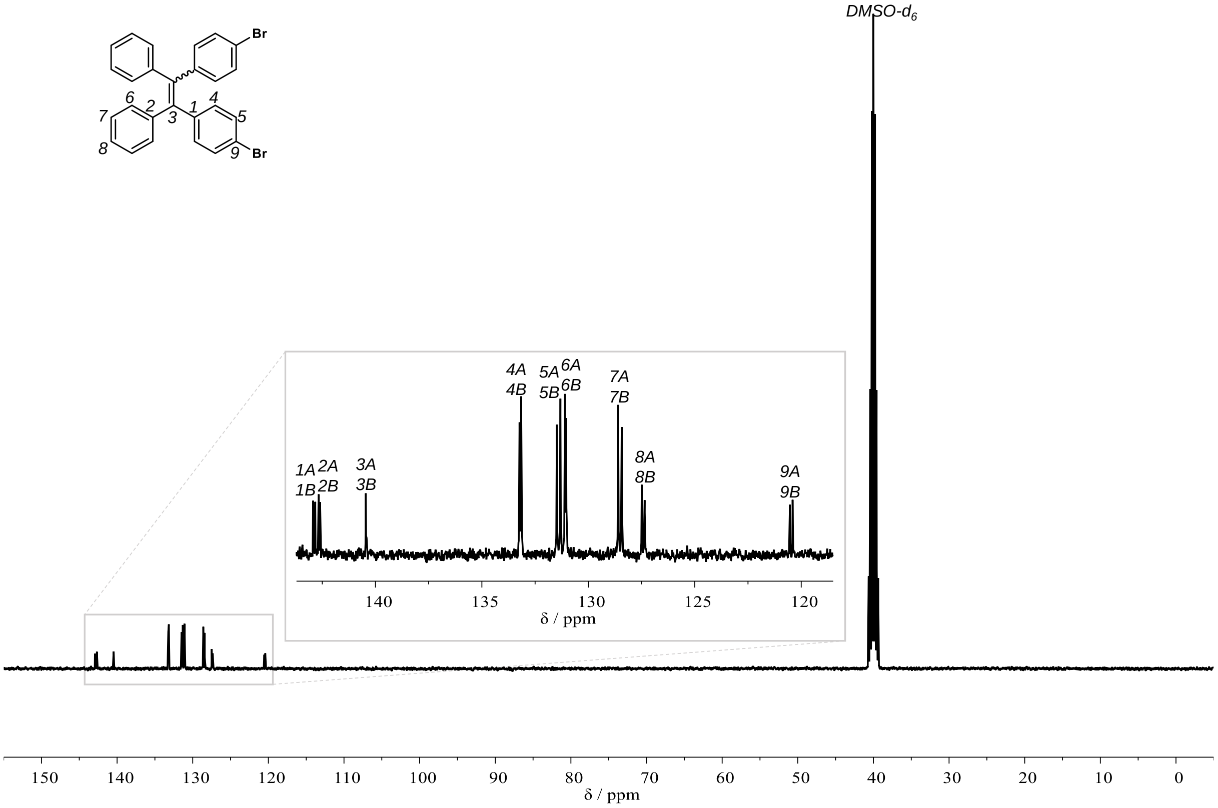

Supplementary Figure 60: ^{13}C-NMR spectrum of 2g.

***Synthesis of 1,2-bis(4-nitrophenyl)-1,2-diphenylethylene* (2h)**

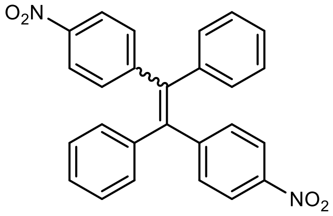

Prepared from **1h** according to the general procedure. The product was isolated *via* column chromatography (cyclohexane/ethyl acetate 40:1) as a yellow solid in a yield of 99% (210 mg, 0.498 mmol). According to ^{1}H-NMR, the isolated products consists of a 55:45 mixture of the *(E)*- and *(Z)*-isomers. With the employed methods, assignment of the signals to respective isomers was not possible.

^{1}H NMR (400 MHz, DMSO-d_6) δ/ppm = 8.09 – 8.00 (m, 4H), 7.32 – 7.23 (m, 4H), 7.27 – 7.13 (m, 6H), 7.07 – 6.96 (m, 4H).

^{13}C NMR (101 MHz, DMSO-d_6) δ/ppm 149.65, 149.41, 146.06, 145.92, 141.51, 141.25, 141.03, 140.96, 131.97, 131.86, 130.68, 130.57, 128.40, 128.18, 127.69, 127.47, 123.36, 123.14.

IR (ATR platinum diamond): ν/cm^{-1} = 3079 (vw), 3059 (vw), 3017 (vw), 2927 (vw), 2847 (vw), 1590 (m), 1512 (vs), 1491 (m), 1444 (w), 1407 (vw), 1337 (vs), 1269 (w), 1246 (w), 1179 (w), 1156 (vw), 1107 (w), 1074 (w), 1028 (w), 1014 (w), 977 (vw), 917 (vw), 870 (w), 856 (w), 843 (m), 821 (w), 771 (w), 749 (s), 697 (vs), 640 (w), 619 (vw), 594 (w), 570 (vw), 490 (w).

EI-HRMS m/z: $[M]^+$ calculated for $C_{26}H_{18}N_2O_4$ = 422.1261, found 422.1262.

Fluorescence: λ_{em}(max) = 534 nm with λ_{ex} = 456 nm

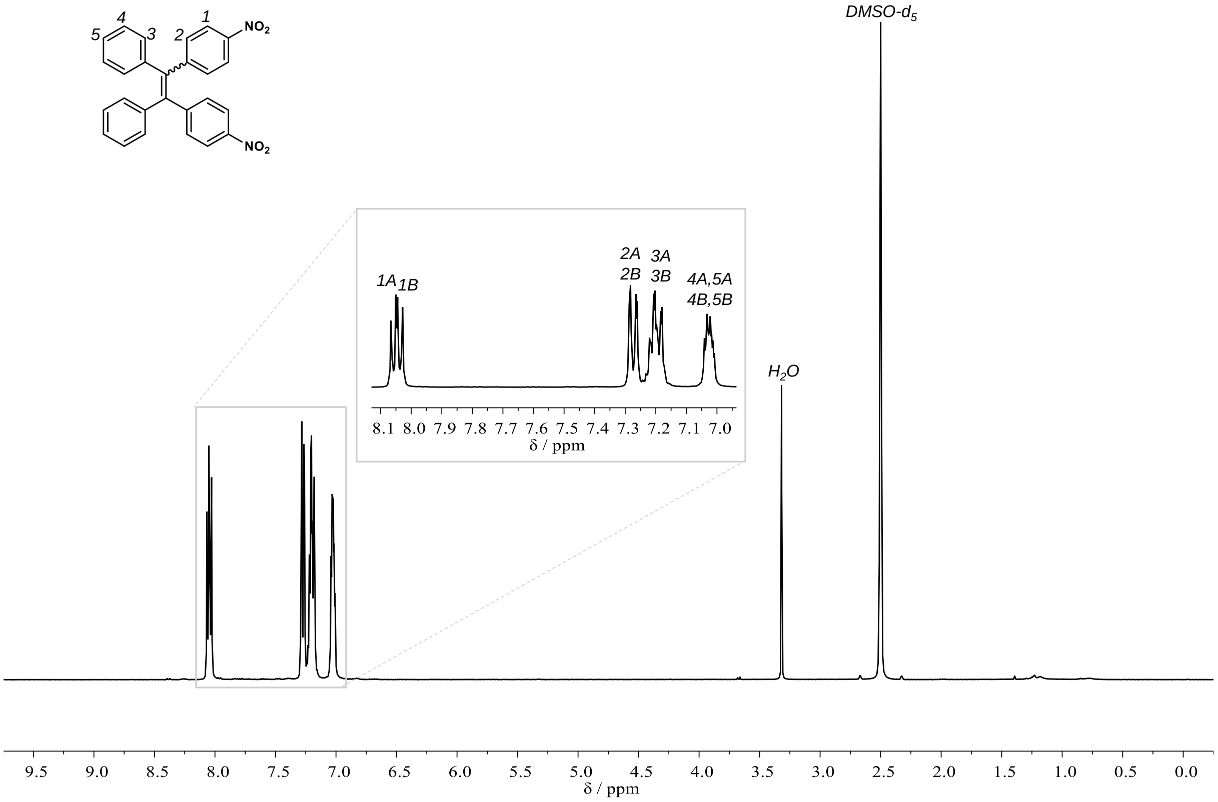

Supplementary Figure 61: ^{1}H-NMR spectrum of 2h.

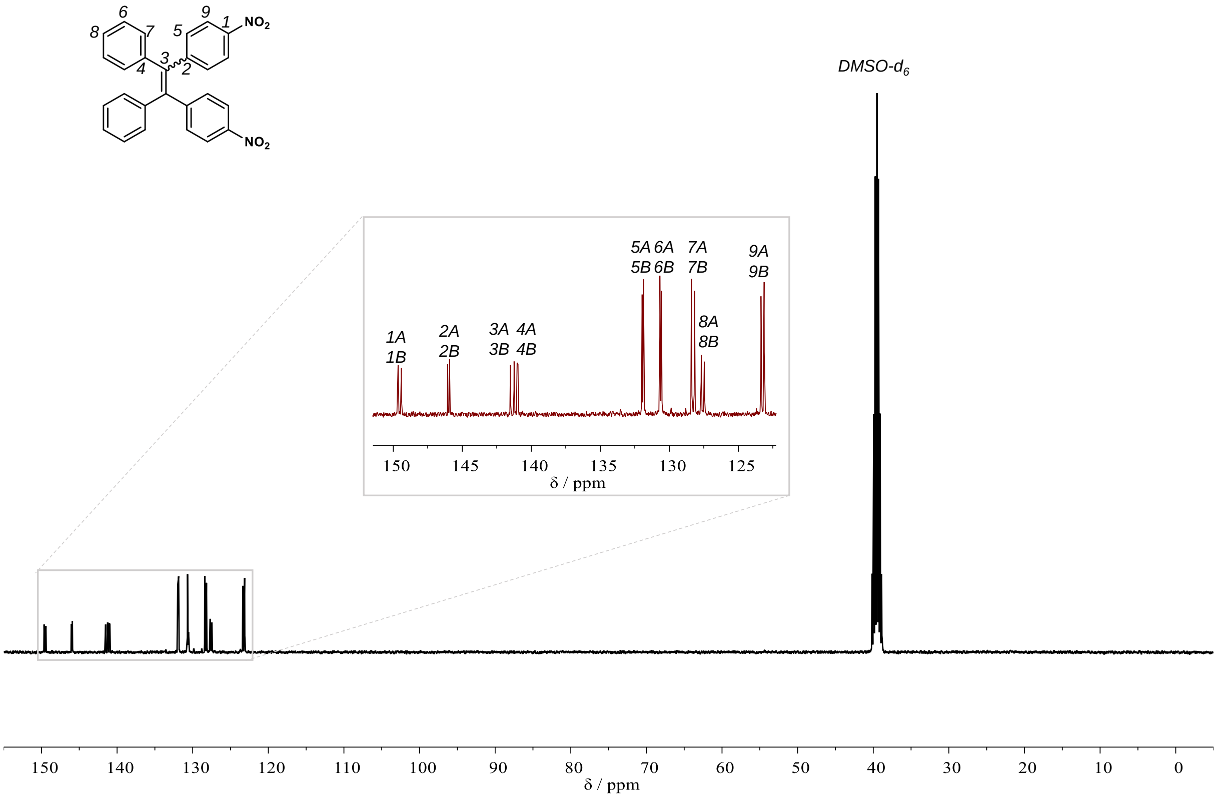

Supplementary Figure 62: ^{13}C-NMR spectrum of 2h.

1.3.6 Cross-coupling products

General procedure for the alkylidene cross-coupling of N-tosylhydrazones and thioketones:

0.50 mmol (1.00 eq.) of the *N*-tosylhydrazone were dissolved in 2 mL of dry DMSO and 0.51 mmol of the thioketone (1.02 eq.) were added. The mixture was heated to 100 °C and 82.92 mg (0.60 mmol, 1.20 eq.) of potassium carbonate were added. After stirring at 100 °C for 30 minutes, 32.1 mg (0.125 mmol, 0.25 eq.) of elemental sulfur were added and stirring at 100°C was continued for another 30 minutes. Afterwards, the crude mixture was transferred directly onto a column (packed in pure cyclohexane) and the product was isolated *via* column chromatography.

***Synthesis of 1,1-bis(4-methoxyphenyl)-2,2-diphenylethylene* (7a)**

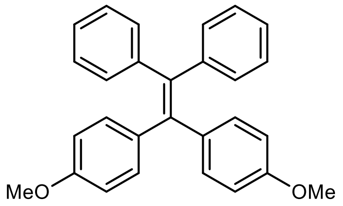

Prepared from NTH **1b** and thioketone **5a** according to general procedure. The product was isolated via column chromatography (cyclohexane/ethyl acetate 40:1). Obtained as a white solid in a yield of 85% (167 mg, 0.426 mmol).

^{1}H NMR (400 MHz, DMSO-d_6) δ/ppm = 7.18 – 7.09 (m, 4H), 7.13 – 7.04 (m, 2H), 6.99 – 6.91 (m, 4H), 6.90 – 6.81 (m, 4H), 6.72 – 6.64 (m, 4H), 3.67 (s, 6H).

^{13}C NMR (101 MHz, DMSO-d_6) δ/ppm = 157.69, 143.79, 139.81, 138.75, 135.62, 131.98, 130.69, 127.82, 126.21, 113.16, 54.90.

IR (ATR platinum diamond): v/cm^{-1} = 3005 (w), 2964 (w), 2931 (vw), 2836 (w), 1604 (m), 1574 (w), 1506 (vs), 1462 (m), 1442 (m), 1413 (w), 1296 (m), 1240 (vs), 1174 (vs), 1158 (m), 1129 (w), 1113 (w), 1074 (w), 1026 (vs), 835 (s), 825 (s), 806 (m), 767 (s), 753 (s), 736 (m), 695 (vs), 660 (w), 640 (vw), 615 (vw), 592 (vs), 572 (w), 545 (s), 518 (w), 467 (w).

ESI-HRMS m/z: $[M]^+$ calculated for $C_{28}H_{24}O_2$ = 392.1771, found 392.1769.

Fluorescence: λ_{em}(max) = 480 nm with λ_{ex} = 321 nm

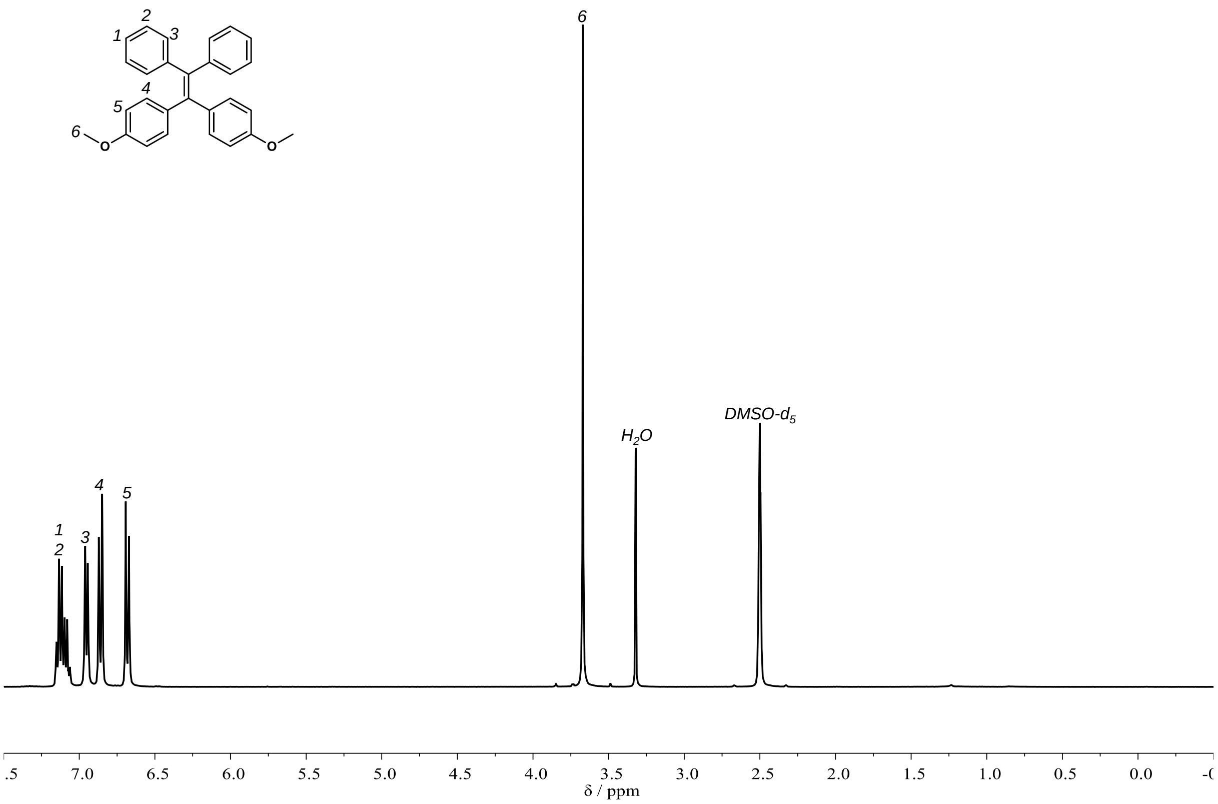

Supplementary Figure 63: ^{1}H-NMR spectrum of 7a.

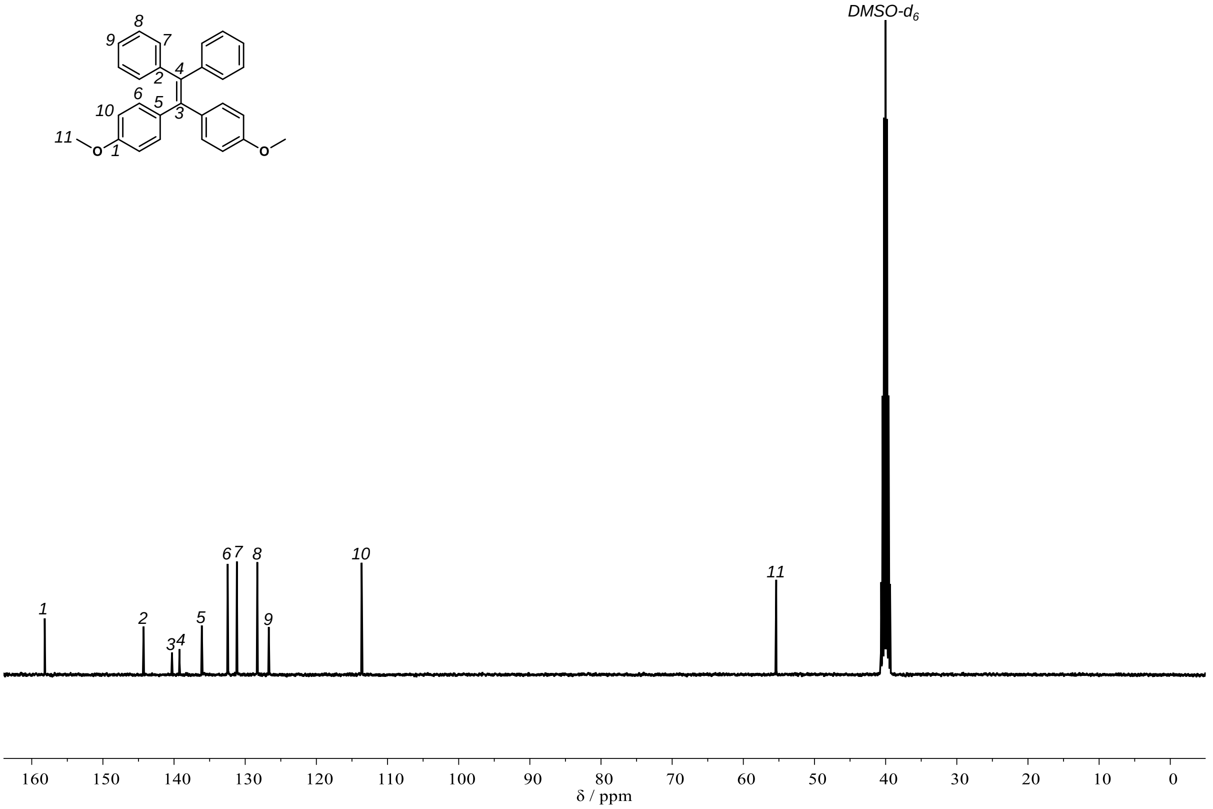

Supplementary Figure 64: ^{13}C-NMR spectrum of 7a.

***Synthesis of 1,1-bis(4-bromophenyl)-2,2-bis(4-methoxyphenyl)ethylene* (7b)**

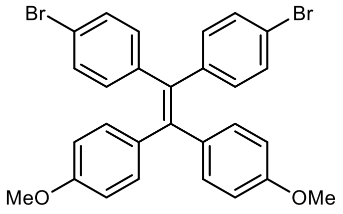

Method A:
Prepared from NTH **1c** and thioketone **5a** according to general procedure.. The product was isolated via column chromatography (cyclohexane/ethyl acetate 30:1). Obtained as a slightly yellow solid in a yield of 85% (233 mg, 0.423 mmol).

Method B:
Prepared from NTH **1a** and thioketone **5c** according to general procedure in a smaller scale of 0.4 mmol. The product isolated via column chromatography (cyclohexane/ethyl acetate 30:1). Obtained as a slightly yellow solid in a yield of 83% (182 mg, 0.331 mmol).

^{1}H NMR (400 MHz, DMSO-d_6) δ/ppm = 7.41 – 7.32 (m, 4H), 6.95 – 6.83 (m, 8H), 6.80 – 6.62 (m, 4H), 3.69 (s, 6H).

^{13}C NMR (101 MHz, DMSO-d_6) δ/ppm = 158.02, 142.66, 141.23, 136.18, 135.03, 132.89, 132.00, 130.94, 119.58, 113.38, 54.95.

IR (ATR platinum diamond): ν/cm^{-1} = 3007 (vw), 2956 (vw), 2931 (vw), 2836 (vw), 1905 (vw), 1604 (m), 1586 (w), 1508 (vs), 1481 (m), 1458 (w), 1440 (w), 1388 (w), 1294 (w), 1281 (w), 1242 (vs), 1177 (s), 1127 (w), 1103 (w), 1068 (m), 1026 (vs), 1008 (vs), 975 (w), 862 (w), 829 (vs), 808 (vs), 780 (s), 740 (w), 726 (w), 666 (w), 592 (m), 570 (w), 559 (m), 508 (w), 483 (s), 465 (w).

ESI-HRMS m/z: $[M]^+$ calculated for $C_{28}H_{22}O_2{}^{79}Br^{81}Br$ = 549.9966, found 549.9963

Fluorescence: λ_{em}(max) = 493 nm with λ_{ex} = 332 nm

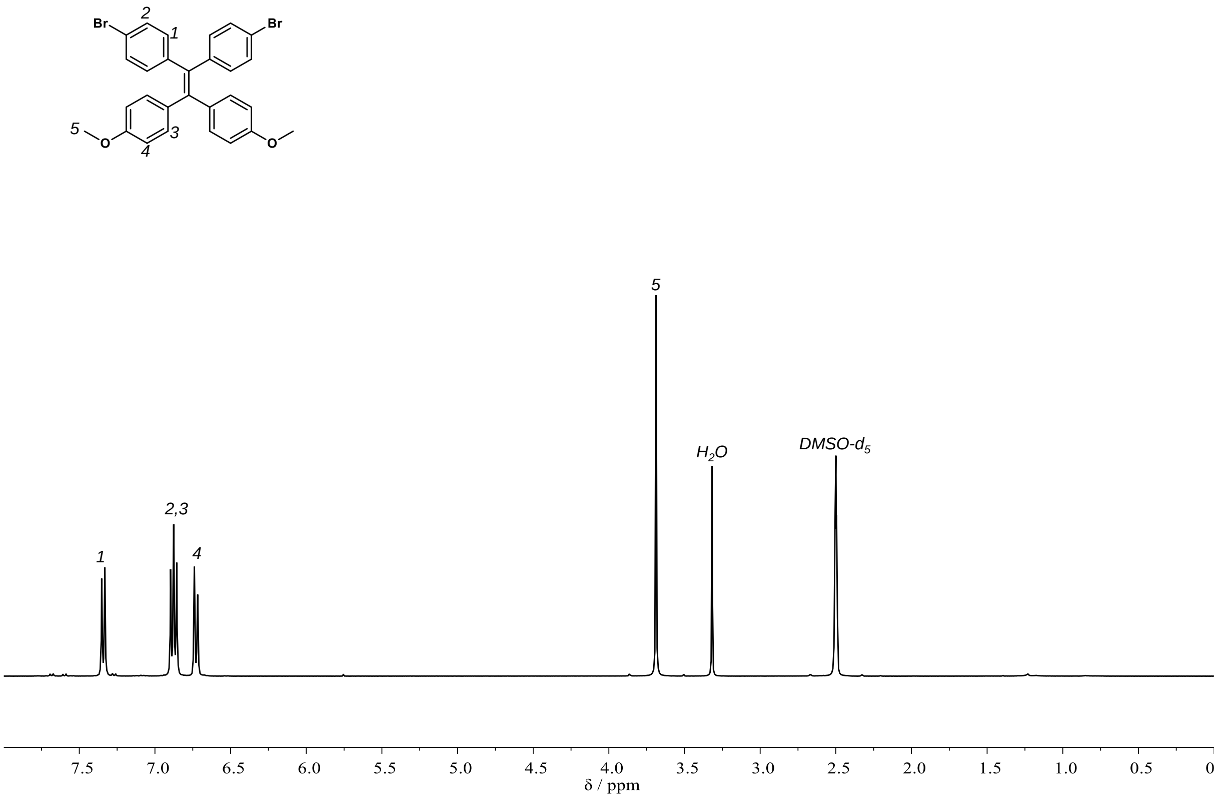

Supplementary Figure 65: ^{1}H-NMR spectrum of 7b.

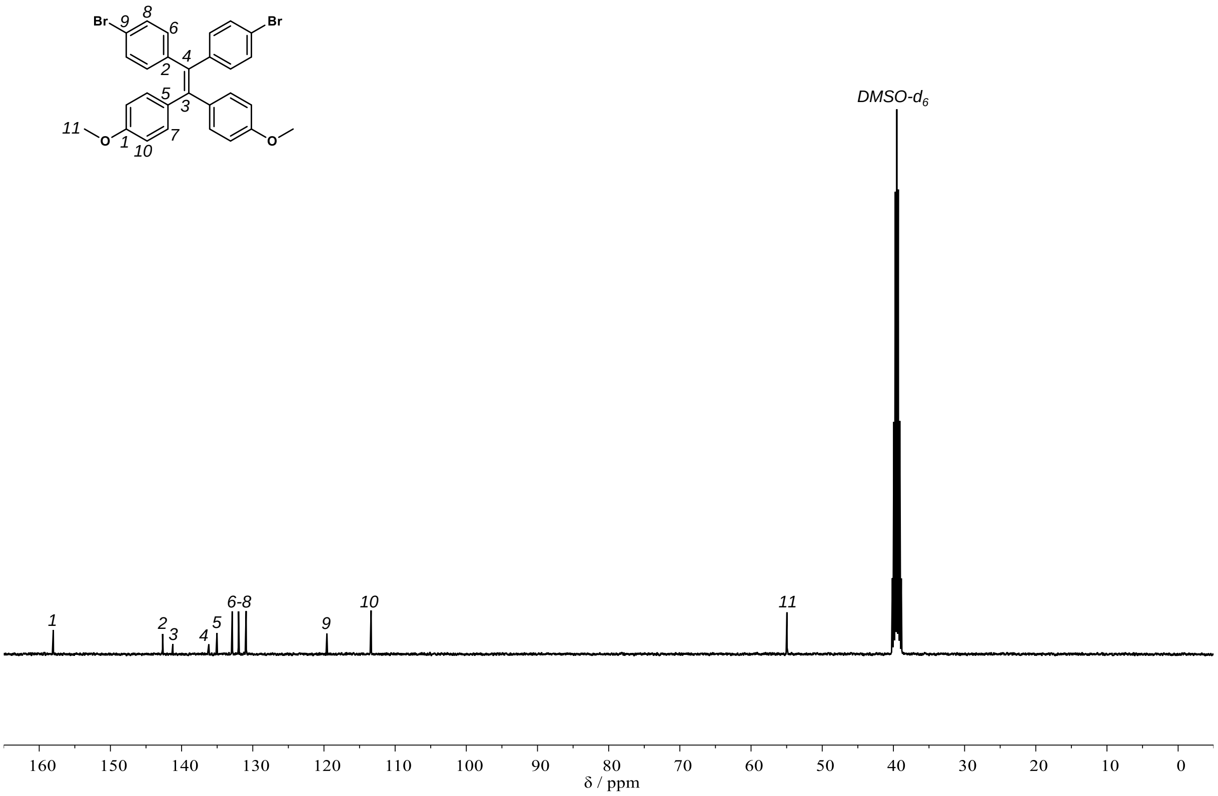

Supplementary Figure 66: ^{13}C-NMR spectrum of 7b.

***Synthesis of 1,1-bis(4-methoxyphenyl)-2,2-bis(4-nitrophenyl)ethylene* (7c)**

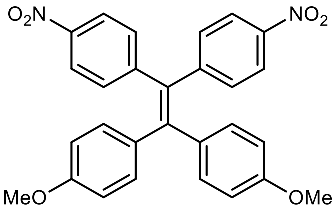

Prepared from NTH **1d** and thioketone **5a** according to general procedure.. The product was isolated via column chromatography (cyclohexane/ethyl acetate 20:1). Obtained as an orange solid in a yield of 79% (190 mg, 0.394 mmol).

^{1}H NMR (400 MHz, DMSO-d_6) δ/ppm = 8.11 – 8.00 (m, 4H), 7.29 – 7.17 (m, 4H), 6.98 – 6.88 (m, 4H), 6.81 – 6.72 (m, 4H), 3.69 (s, 6H).

^{13}C NMR (101 MHz, DMSO-d_6) δ/ppm = 159.19, 150.77, 146.09, 145.67, 135.30, 134.56, 132.83, 132.68, 123.85, 114.05, 55.51.

IR (ATR platinum diamond): ν/cm^{-1} = 3073 (vw), 2958 (vw), 2933 (vw), 2836 (w), 1590 (m), 1506 (vs), 1462 (w), 1440 (w), 1337 (vs), 1292 (m), 1244 (vs), 1172 (vs), 1131 (w), 1107 (s), 1028 (s), 1014 (m), 977 (w), 961 (w), 874 (w), 856 (w), 841 (s), 833 (s), 815 (s), 784 (w), 753 (m), 736 (w), 703 (m), 689 (w), 668 (w), 594 (w), 570 (w), 559 (w), 514 (w).

ESI-HRMS m/z: $[M+H]^+$ calculated for $C_{28}H_{22}N_2O_6$ = 483.1551, found 483.1543.

Fluorescence: λ_{em}(max) = 581 nm with λ_{ex} = 383 nm.

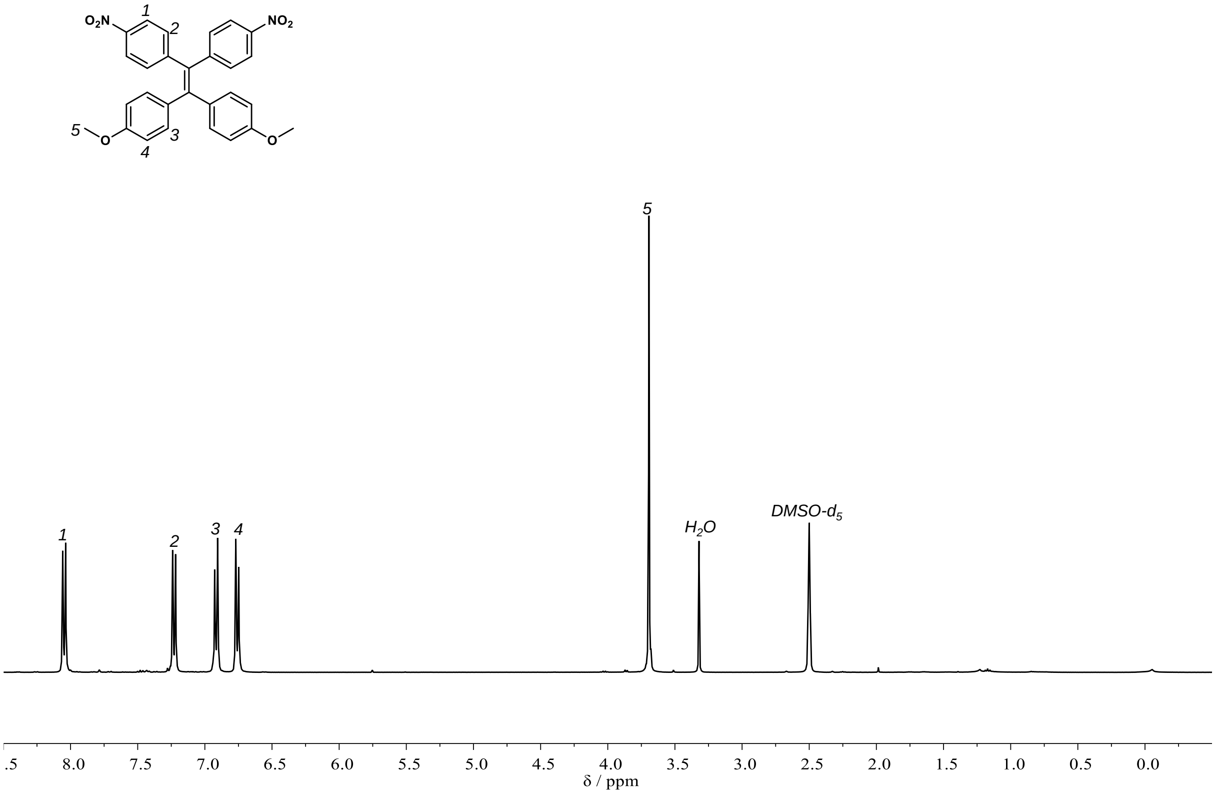

Supplementary Figure 67: ^{1}H-NMR spectrum of 7c.

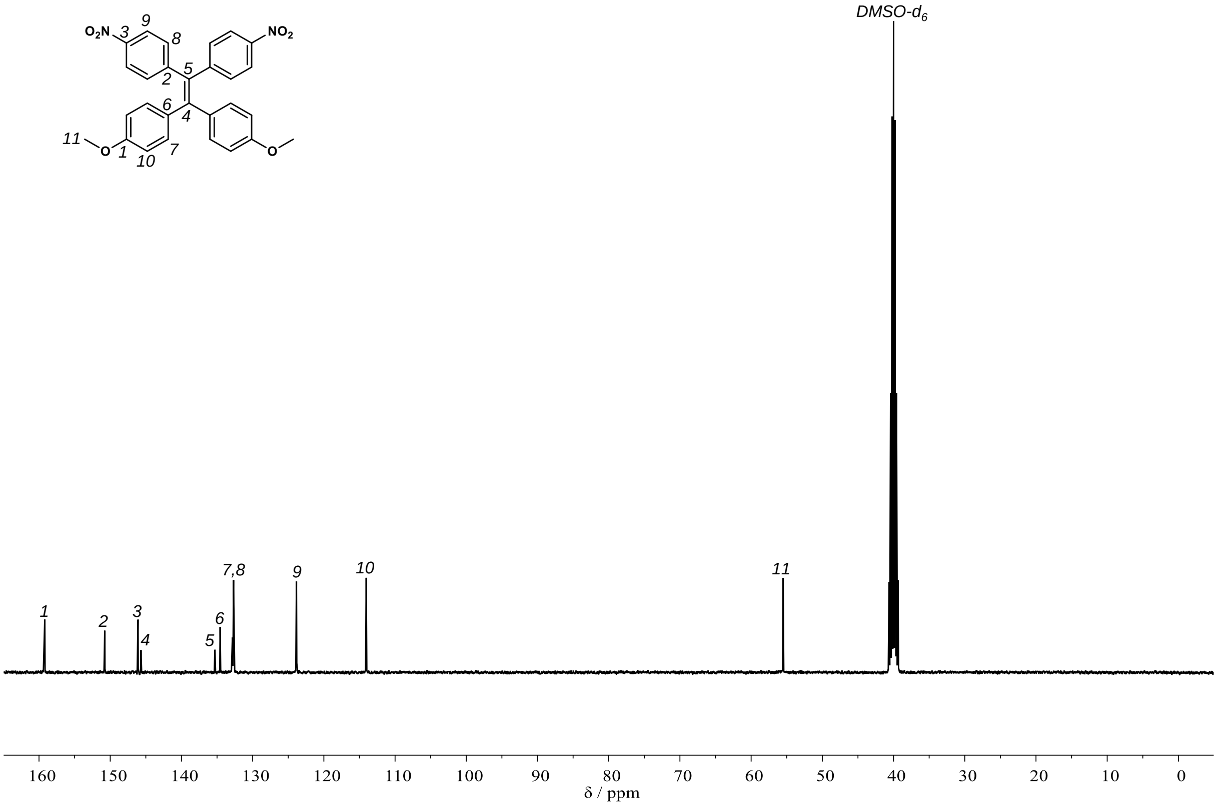

Supplementary Figure 68: ^{13}C-NMR spectrum of 7c.

***Synthesis of 1,1,2-tris(4-methoxyphenyl)ethylene* (7d)**

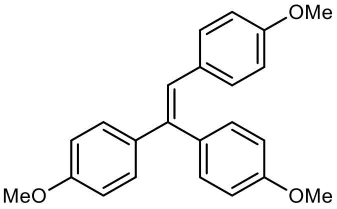

Prepared from NTH **1i** and thioketone **5a** according to general procedure.. The product was isolated via column chromatography (cyclohexane/ethyl acetate 30:1). Obtained as a colorless, wax-like solid in a yield of 56% (190 mg, 0.394 mmol).

^{1}H NMR (400 MHz, DMSO-d_6) δ/ppm = 7.23 – 7.14 (m, 2H), 7.07 – 6.99 (m, 2H), 6.99 – 6.91 (m, 4H), 6.94 – 6.83 (m, 3H), 6.75 – 6.67 (m, 2H), 3.78 (s, 3H), 3.74 (s, 3H), 3.68 (s, 3H).

^{13}C NMR (101 MHz, DMSO-d_6) δ/ppm = 158.66, 158.45, 157.82, 138.93, 135.62, 132.35, 131.00, 130.32, 129.88, 128.12, 125.21, 114.32, 113.66, 113.45, 55.11, 55.01, 54.97.

IR (ATR platinum diamond): v/cm^{-1} = 3032 (vw), 2999 (vw), 2954 (w), 2927 (w), 2871 (vw), 2851 (w), 2834 (w), 1695 (vw), 1635 (w), 1600 (vs), 1574 (w), 1508 (vs), 1460 (m), 1440 (m), 1415 (w), 1374 (vw), 1302 (m), 1292 (m), 1240 (vs), 1170 (vs), 1109 (m), 1026 (vs), 967 (w), 948 (w), 930 (w), 884 (w), 850 (w), 829 (vs), 804 (m), 786 (w), 765 (m), 755 (w), 736 (w), 685 (w), 629 (vw), 613 (w), 592 (w), 562 (w), 533 (m), 508 (w).

ESI-HRMS m/z: [M]$^+$ calculated for $C_{23}H_{22}O_3$ = 346.1563, found 346.1557.

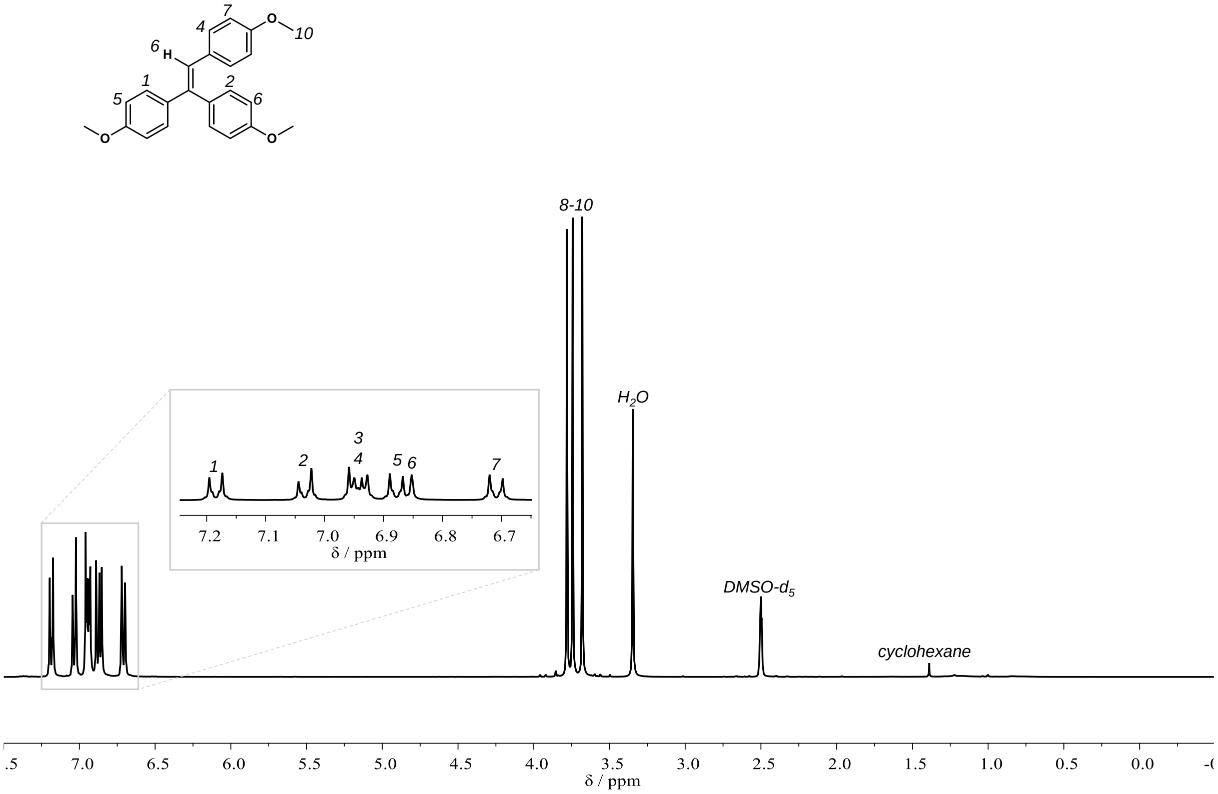

Supplementary Figure 69: ¹H-NMR spectrum of 7d.

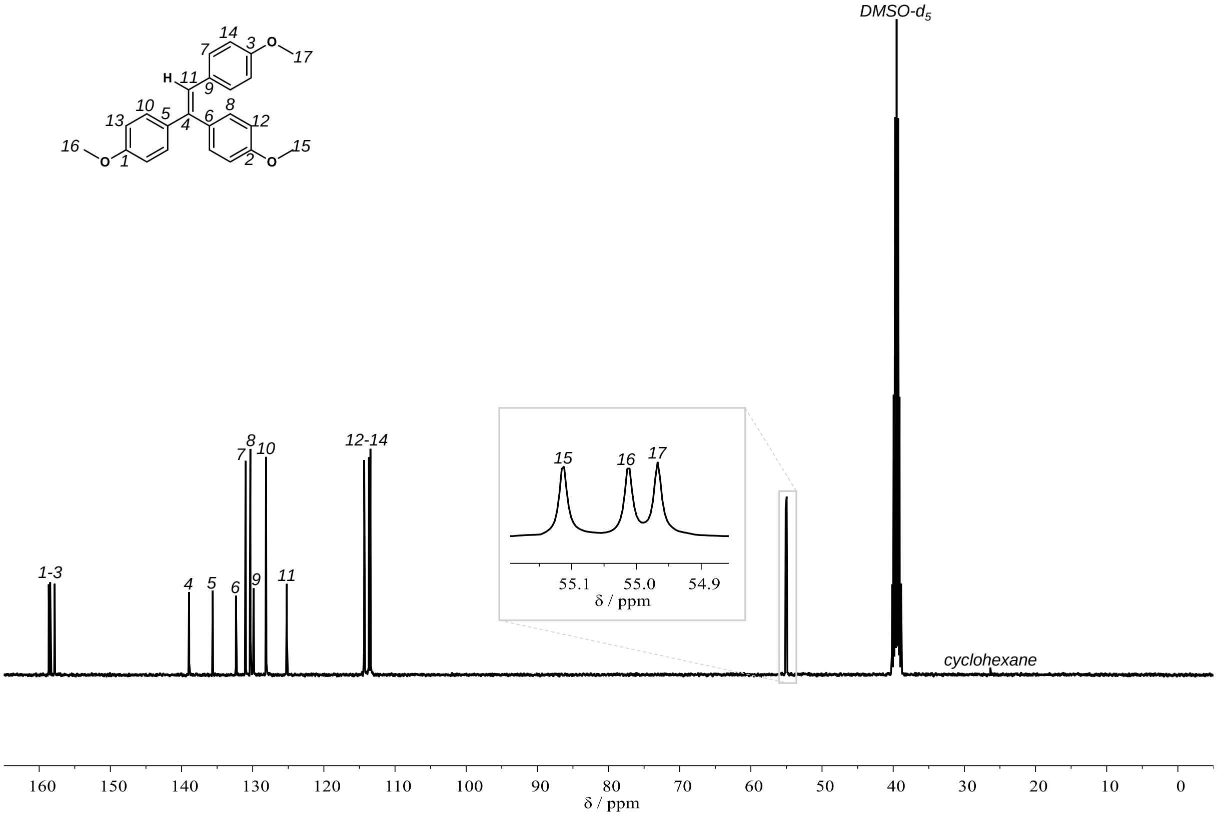

Supplementary Figure 70: ¹³C-NMR spectrum of 7d.

***Synthesis of 1,1,2-tris(4-methoxyphenyl)propylene* (7e)**

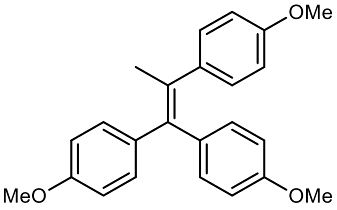

Prepared from NTH **1j** and thioketone **5a** according to general procedure in a smaller scale using 0.25 mmol NTH. The product was isolated via column chromatography (cyclohexane/ethyl acetate 30:1). Obtained as a colorless solid in a yield of 93% (84.2 mg, 0.234 mmol).

^{1}H NMR (400 MHz, DMSO-d_6) δ/ppm = 7.14 – 7.01 (m, 4H), 6.95 – 6.87 (m, 2H), 6.80 – 6.70 (m, 4H), 6.66 – 6.58 (m, 2H), 3.76 (s, 3H), 3.68 (s, 3H), 3.64 (s, 3H), 2.02 (s, 3H).

^{13}C NMR (101 MHz, DMSO-d_6) δ/ppm = 157.75, 157.37, 157.08, 137.27, 135.92, 135.81, 135.63, 133.25, 131.48, 130.78, 130.15, 113.53, 113.29, 112.97, 54.99, 54.88, 54.80, 23.20.

IR (ATR platinum diamond): ν/cm^{-1} = 3005 (vw), 2954 (w), 2929 (w), 2906 (w), 2853 (vw), 2834 (w), 1604 (m), 1571 (w), 1506 (vs), 1462 (m), 1442 (m), 1409 (vw), 1292 (m), 1273 (w), 1236 (vs), 1170 (vs), 1146 (w), 1107 (w), 1078 (w), 1028 (vs), 1002 (m), 954 (vw), 911 (w), 829 (vs), 808 (vs), 782 (w), 749 (w), 734 (w), 716 (w), 574 (s), 545 (w), 522 (w), 494 (w), 477 (w).

ESI-HRMS m/z: $[M]^+$ calculated for $C_{24}H_{24}O_3$ = 360.1720, found 360.1714.

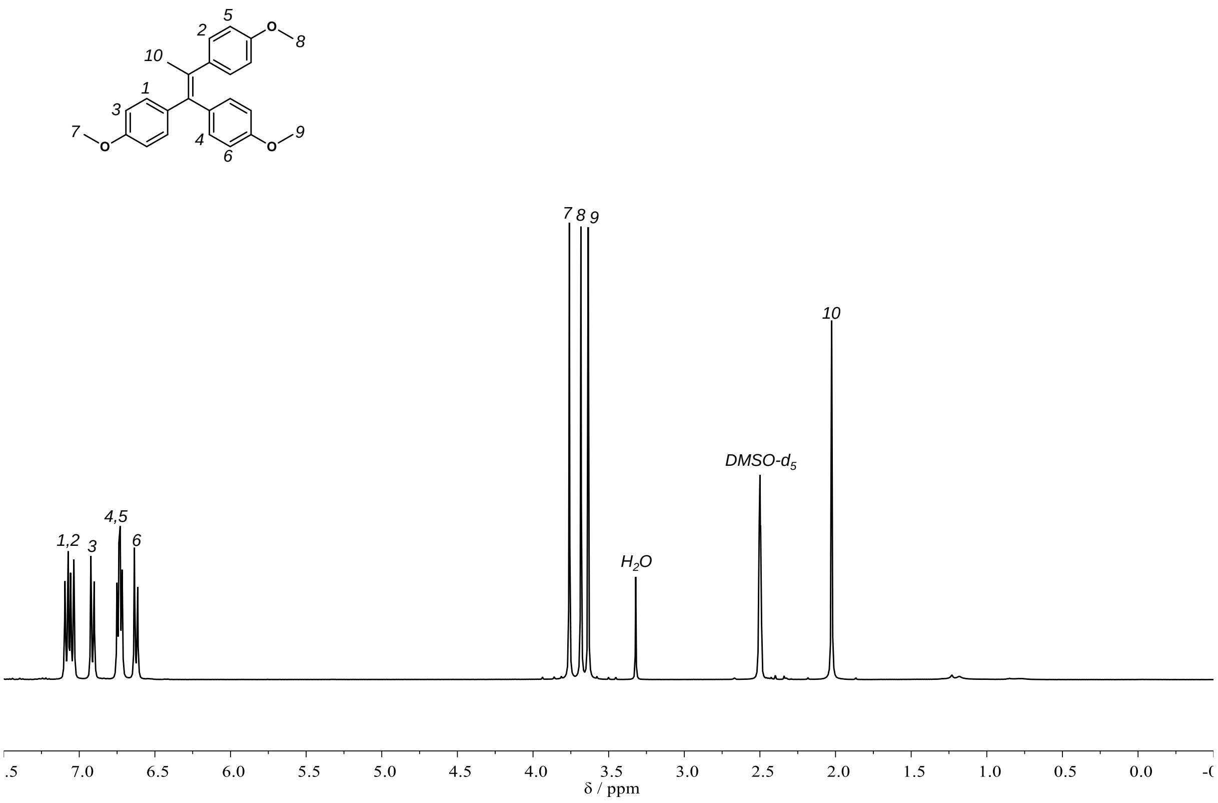

Supplementary Figure 71: ^{1}H-NMR spectrum of 7e.

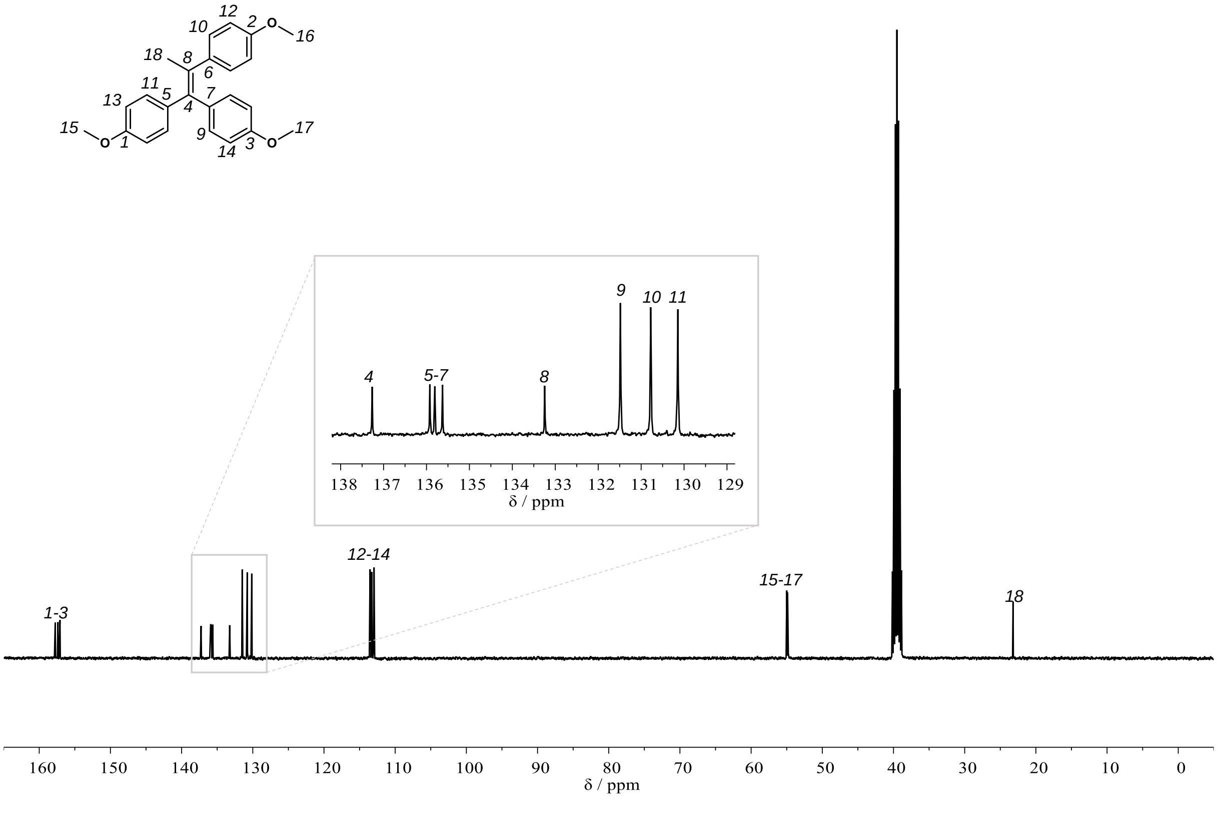

Supplementary Figure 72: ^{13}C-NMR spectrum of 7e.

***Synthesis of 3-(4,4'-dimethoxybenzhydrylidene)pentane* (7f)**

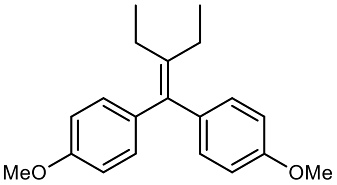

Prepared from NTH **1l** and thioketone **5a** according to general procedure in a smaller scale using 0.25 mmol NTH. The product was isolated via column chromatography (cyclohexane/ethyl acetate 30:1). Obtained as a colorless solid in a yield of 79% (117 mg, 0.395 mmol).

^{1}H NMR (400 MHz, DMSO-d_6) δ/ppm = 7.04 – 6.96 (m, 4H), 6.90 – 6.80 (m, 4H), 3.72 (s, 6H), 2.06 (q, J = 7.4 Hz, 4H), 0.97 (t, J = 7.4 Hz, 6H).

^{13}C NMR (101 MHz, DMSO-d_6) δ/ppm = 157.94, 140.99, 136.53, 136.04, 130.29, 113.93, 55.41, 24.43, 13.72.

IR (ATR platinum diamond): ν/cm^{-1} = 3026 (vw), 3013 (vw), 2964 (w), 2931 (w), 2869 (w), 2841 (w), 1604 (m), 1574 (w), 1506 (vs), 1467 (m), 1454 (m), 1444 (m), 1378 (w), 1329 (w), 1302 (w), 1273 (m), 1261 (m), 1234 (vs), 1174 (vs), 1152 (w), 1107 (w), 1092 (w), 1031 (vs), 897 (w), 833 (vs), 825 (vs), 812 (vs), 792 (w), 780 (w), 730 (w), 634 (w), 621 (w), 578 (s), 570 (s), 551 (m), 522 (m).

ESI-HRMS m/z: $[M+H]^+$ calculated for $C_{20}H_{24}O_2$ = 297.1850, found 297.1847.

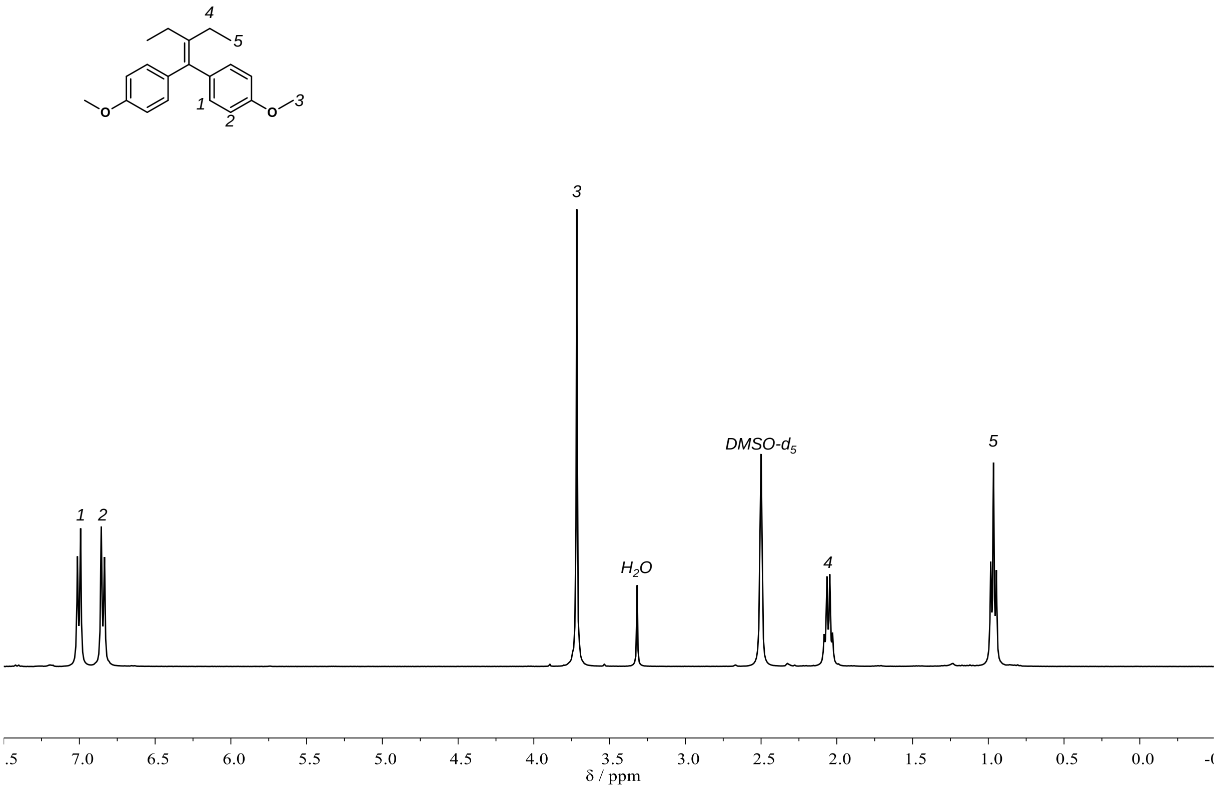

Supplementary Figure 73: ^{1}H-NMR spectrum of 7f.

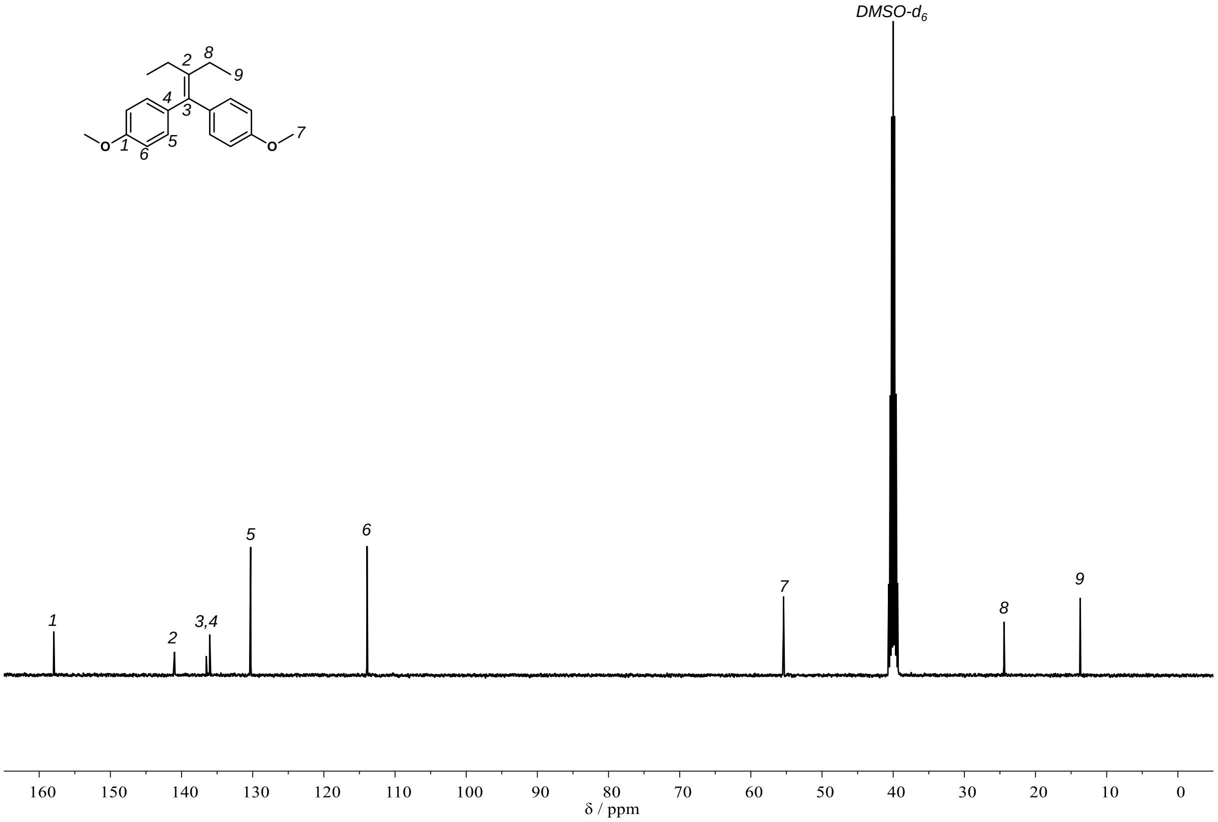

Supplementary Figure 74: ^{13}C-NMR spectrum of 7f.

***Synthesis of 9-(4,4'-dimethoxybenzhydrylidene)thioxanthene* (7g)**

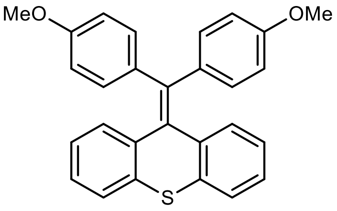

Prepared from NTH **1a** and thioketone **5b** according to general procedure. The product was isolated via column chromatography (cyclohexane/ethyl acetate 30:1). Obtained as a colorless solid in a yield of 95% (200 mg, 0.474 mmol).

^{1}H NMR (400 MHz, DMSO-d_6) δ/ppm = 7.54 – 7.47 (m, 2H), 7.16 – 7.04 (m, 8H), 7.01 – 6.94 (m, 2H), 6.80 – 6.72 (m, 4H), 3.67 (s, 6H).

^{13}C NMR (101 MHz, DMSO-d_6) δ/ppm = 158.25, 142.26, 137.13, 134.60, 134.48, 134.27, 130.84, 129.88, 127.00, 126.92, 126.35, 113.95, 55.43.

IR (ATR platinum diamond): v/cm^{-1} = 3055 (vw), 2999 (vw), 2950 (vw), 2927 (w), 2902 (vw), 2834 (w), 1602 (m), 1571 (w), 1506 (vs), 1456 (m), 1436 (m), 1286 (m), 1267 (w), 1240 (vs), 1172 (vs), 1144 (w), 1109 (w), 1065 (w), 1031 (vs), 973 (w), 940 (w), 852 (vw), 821 (s), 769 (vs), 743 (vs), 736 (vs), 699 (w), 654 (w), 592 (m), 580 (m), 547 (m), 525 (w), 450 (w).

ESI-HRMS m/z: [M]$^+$ calculated for $C_{28}H_{22}O_2S$ = 422.1336, found 422.1332

Fluorescence: λ_{em}(max) = 448 nm with λ_{ex} = 328 nm

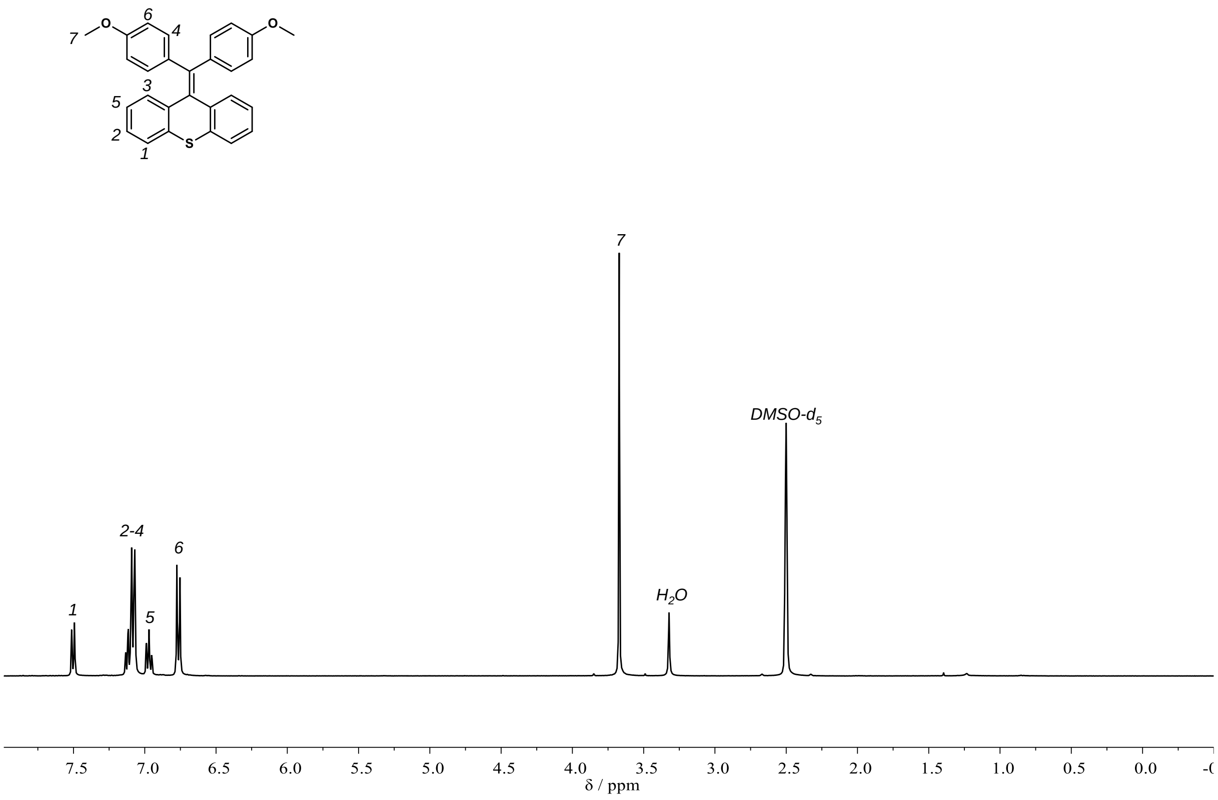

Supplementary Figure 75: ^{1}H-NMR spectrum of 7g.

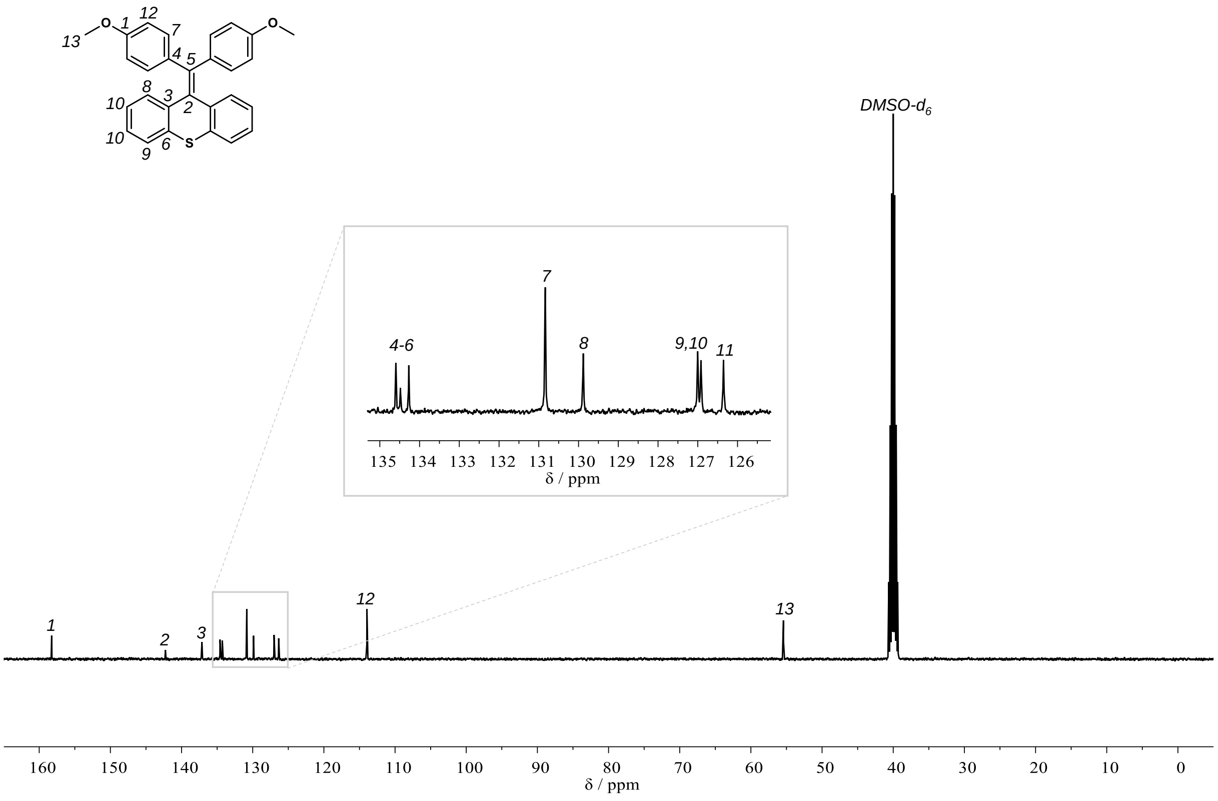

Supplementary Figure 76: ^{13}C-NMR spectrum of 7g.

Synthesis of 9-benzhydrylidenethioxanthene (7h)

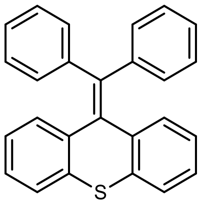

Prepared from NTH **1b** and thioketone **5b** according to general procedure. The product was isolated via column chromatography (cyclohexane/ethyl acetate 30:1). Obtained as a colorless solid in a yield of 99% (179 mg, 0.494 mmol).

^{1}H NMR (400 MHz, DMSO-d_6) δ/ppm = 7.54 – 7.50 (m, 2H), 7.25 – 7.18 (m, 8H), 7.17 – 7.09 (m, 4H), 7.08 – 7.04 (m, 2H), 6.96 – 6.90 (m, 2H).

^{13}C NMR (101 MHz, DMSO-d_6) δ/ppm = 142.96, 142.10, 136.59, 134.84, 134.19, 129.84, 129.56, 128.64, 127.17, 127.10, 126.97, 126.31.

IR (ATR platinum diamond): ν/cm^{-1} = 3044 (vw), 3020 (vw), 2962 (vw), 2921 (w), 2855 (vw), 1738 (w), 1594 (w), 1574 (w), 1555 (w), 1489 (w), 1454 (w), 1438 (s), 1316 (w), 1263 (w), 1240 (w), 1212 (w), 1162 (w), 1156 (w), 1117 (w), 1078 (w), 1065 (w), 1026 (m), 1002 (w), 981 (w), 973 (w), 944 (w), 919 (w), 903 (w), 868 (w), 761 (vs), 755 (vs), 745 (vs), 701 (vs), 631 (m), 613 (s), 580 (m), 539 (m), 518 (w), 485 (w), 475 (w), 448 (w), 409 (w).

ESI-HRMS m/z: [M]$^+$ calculated for $C_{26}H_{18}S$ = 362.1124, found 362.1121.

Fluorescence: λ_{em}(max) = 421 nm with λ_{ex} = 322 nm

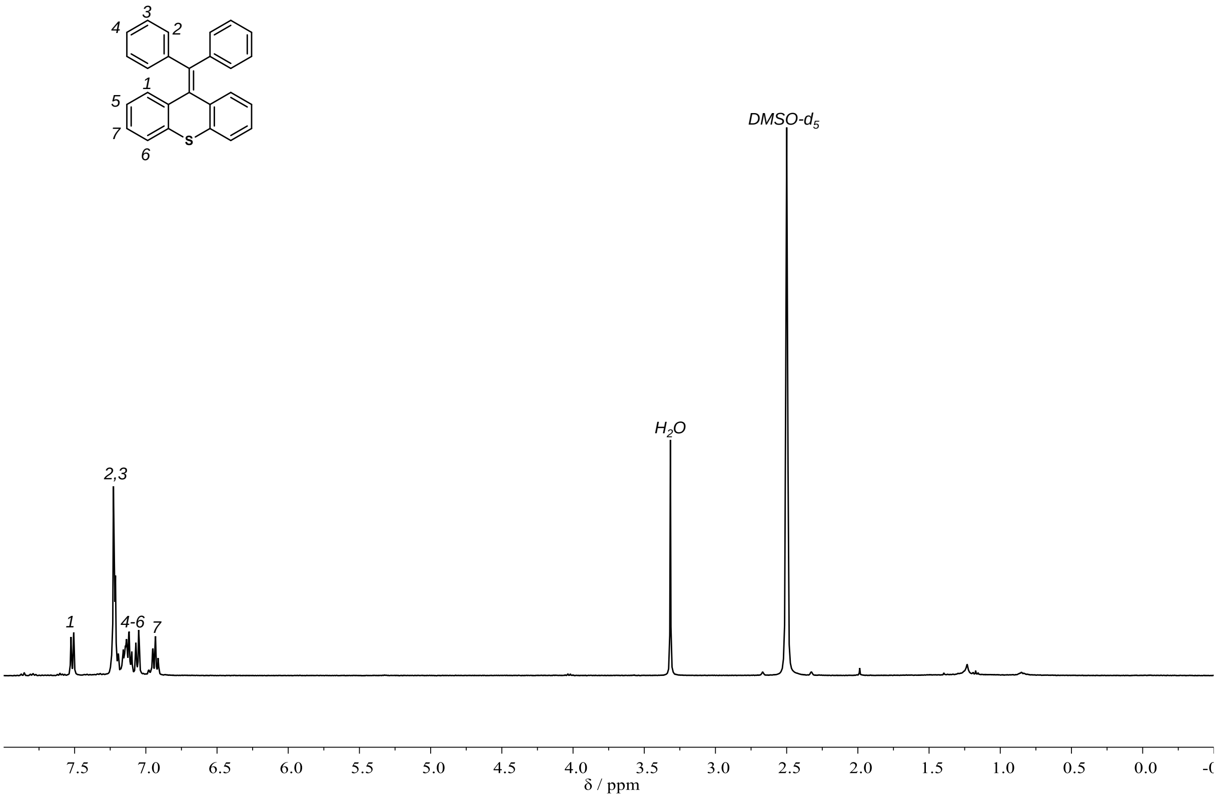

Supplementary Figure 77: ^{1}H-NMR spectrum of 7h.

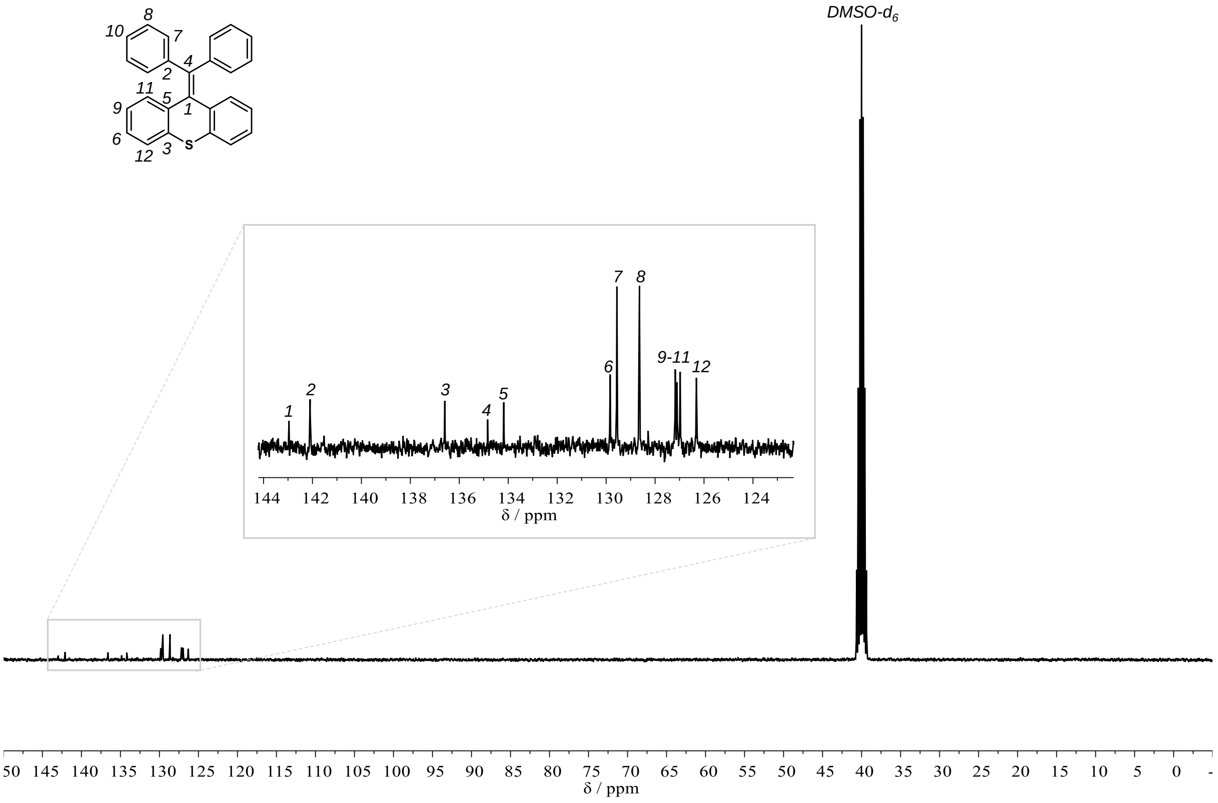

Supplementary Figure 78: ^{13}C-NMR spectrum of 7h.

***Synthesis of 9-benzhydrylidenethioxanthene* (7i)**

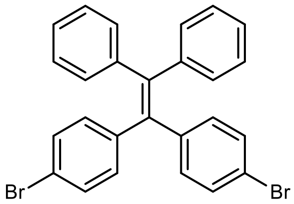

Prepared from NTH **1b** and thioketone **5c** according to general procedure. The product was isolated via column chromatography (cyclohexane/ethyl acetate 30:1). Obtained as a colorless solid in a yield of 99% (220 mg, 0.494 mmol).

^{1}H NMR (400 MHz, DMSO-d_6) δ/ppm = 7.38 – 7.29 (m, 4H), 7.22 – 7.10 (m, 7H), 7.02 – 6.95 (m, 5H), 6.94 – 6.88 (m, 4H).

^{13}C NMR (101 MHz, DMSO-d_6) δ/ppm =143.05, 142.45, 142.37, 138.56, 133.26, 131.40, 131.01, 128.50, 127.44, 120.45.

IR (ATR platinum diamond): ν/cm^{-1} = 3052 (vw), 3026 (vw), 2923 (vw), 2853 (vw), 1660 (vw), 1586 (w), 1487 (m), 1442 (w), 1390 (w), 1275 (vw), 1179 (vw), 1105 (w), 1070 (m), 1028 (vw), 1010 (s), 971 (w), 924 (vw), 864 (vw), 831 (s), 810 (w), 767 (m), 757 (s), 726 (w), 697 (vs), 673 (w), 638 (w), 623 (w), 617 (w), 570 (w), 485 (m), 455 (w).

EI-HRMS m/z: [M]$^+$ calculated for $C_{26}H_{18}{}^{79}Br^{81}Br$ = 489.9749, found 489.9751.

Fluorescence: λ_{em}(max) = 476 nm with λ_{ex} = 313 nm.

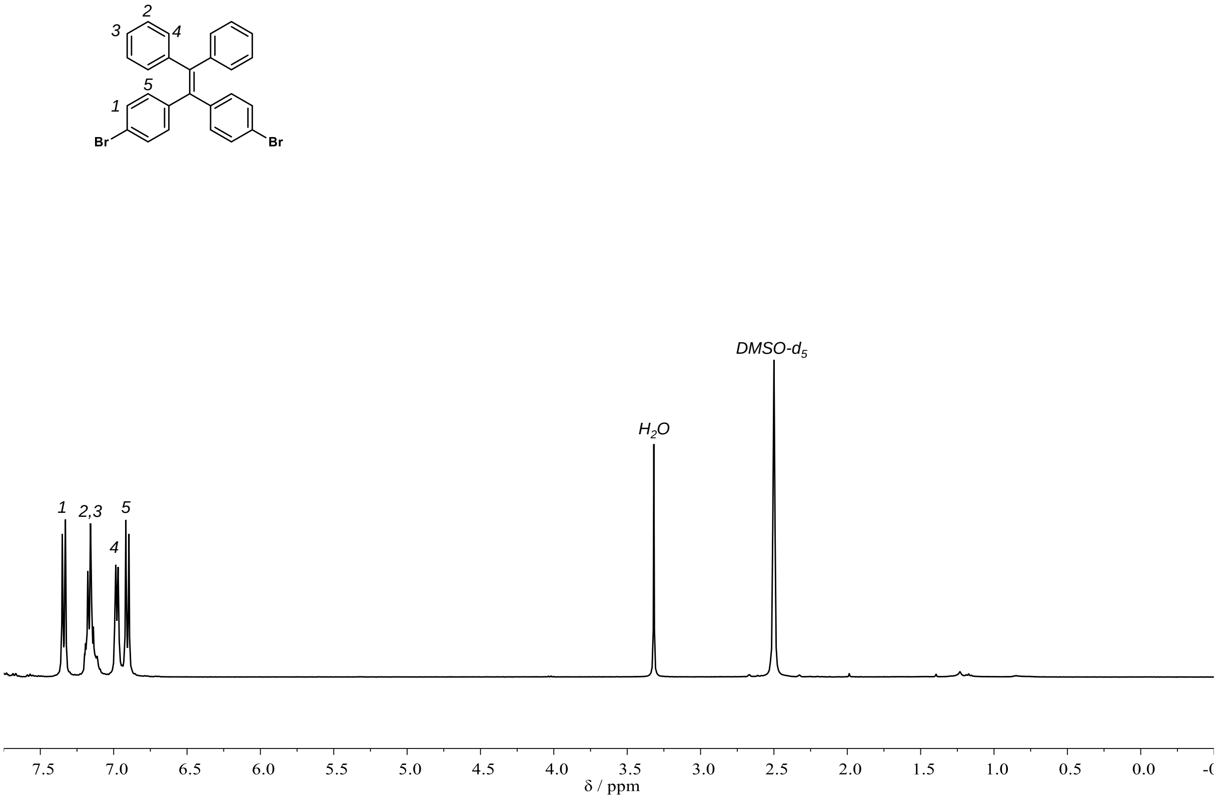

Supplementary Figure 79: ^{1}H-NMR spectrum of 7i.

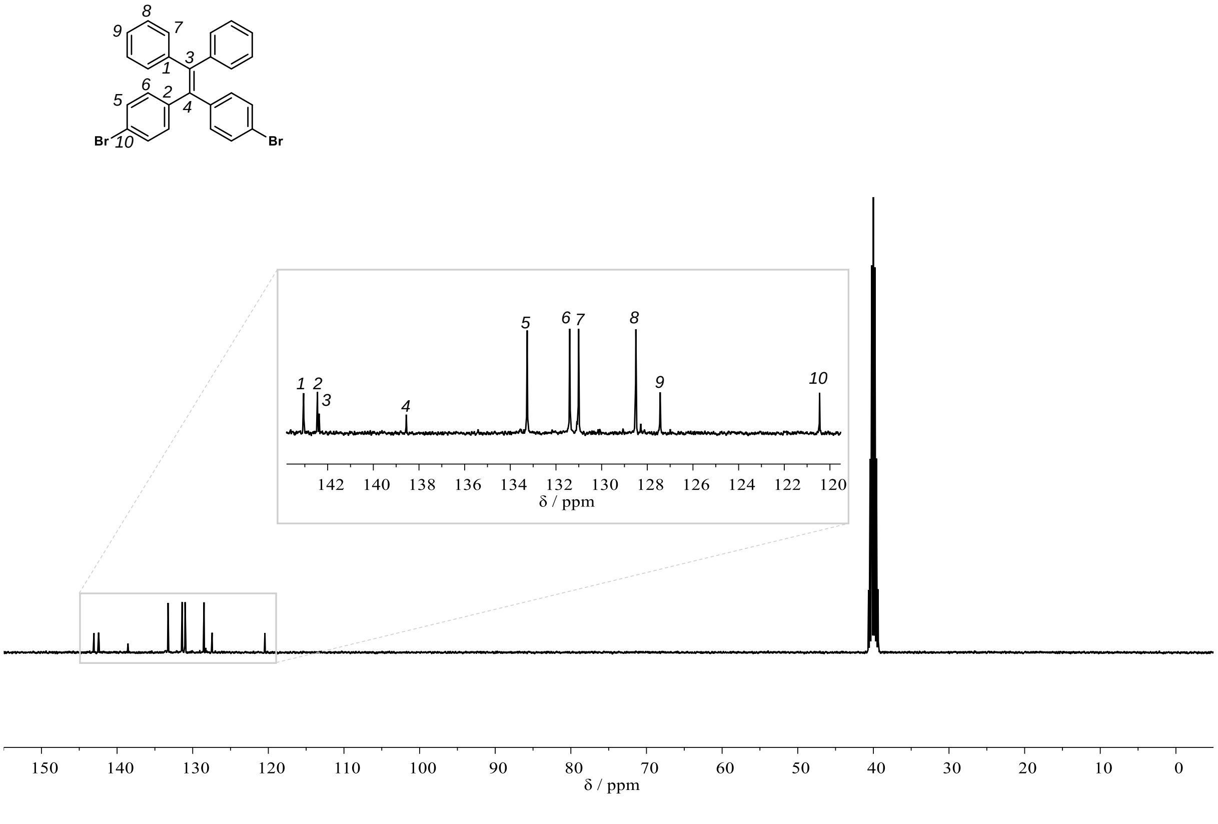

Supplementary Figure 80: ^{13}C-NMR spectrum of 7i.

1.4 Fluorescence Measurements

Most TPE derivatives synthesized in this work exhibited characteristic AIE properties, as was apparent by their lack of fluorescence in solute state and a strong emission in aggregated states. Fluorescence measurements were conducted for all compounds exhibiting this behavior, namely all homo- and cross-coupling products except **2e**, **7d**, **7e** and **7f**.

Since the measured TPE derivatives were not emissive in solution, fluorescence was turned on by adding to a solution of the analytes in THF to an antisolvent (water/THF 9:1). For the measurements, a concentration of approximately 20 µM in water/THF was used (10 µL of a 10 µM analyte solution was added to 5 mL of water/THF). Under these conditions, the formation of aggregates was visible by slight turbidity of the sample, turning on the fluorescence of the TPE derivatives.

The excitation wavelengths used for each TPE were determined by recording an absorption spectrum (not depicted) and using the wavelength with the highest absorption for fluorescence excitation.

The measured excitation and maximum emission wavelengths for each AIE-active TPE synthesized in this work are compiled in Supplementary Table S1.

Supplementary Table 1: Excitation and Emission wavelengths for each AIE-active TPE synthesized in this work.

Compound	λ (excitation) / nm	λ (emission) / nm
**2a**	327	490
**2b**	305	469
**2c**	321	476
**2d**	325	509
**2f**	321	486
**2g**	315	477
**2h**	456	534
**7a**	321	480
**7b**	332	493
**7c**	383	571
**7g**	328	448
**7h**	322	421
**7i**	313	476

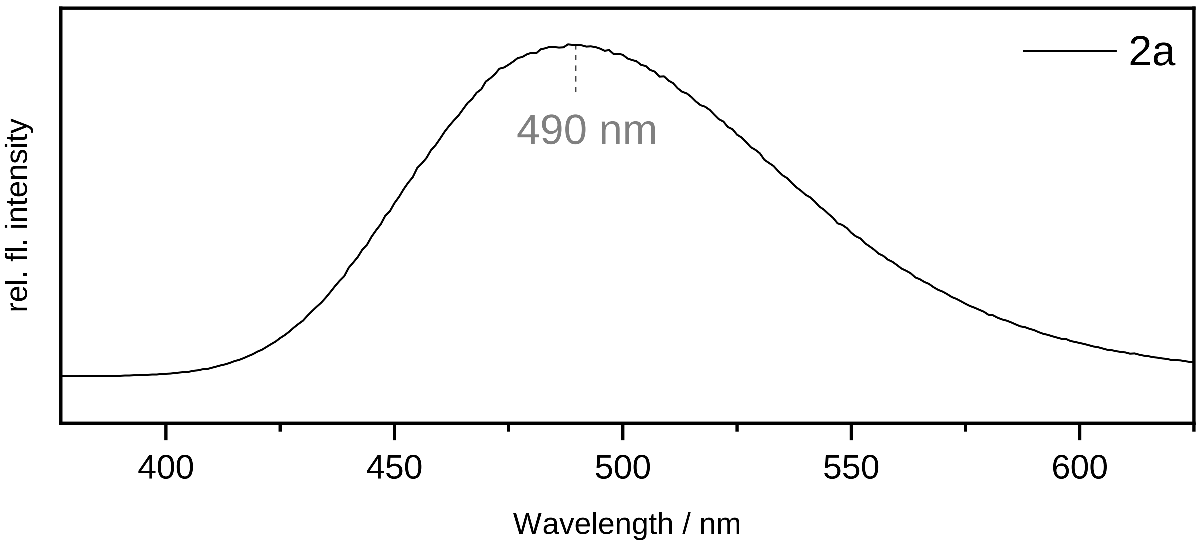

Supplementary Figure 81: Fluorescence spectrum of 2a.

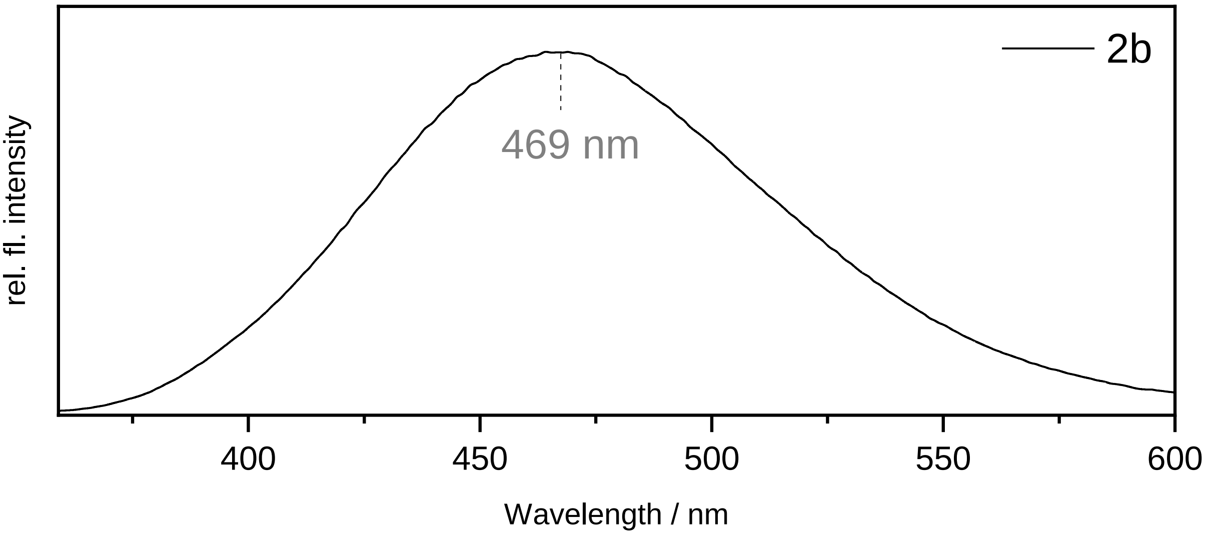

Supplementary Figure 82: Fluorescence spectrum of 2b.

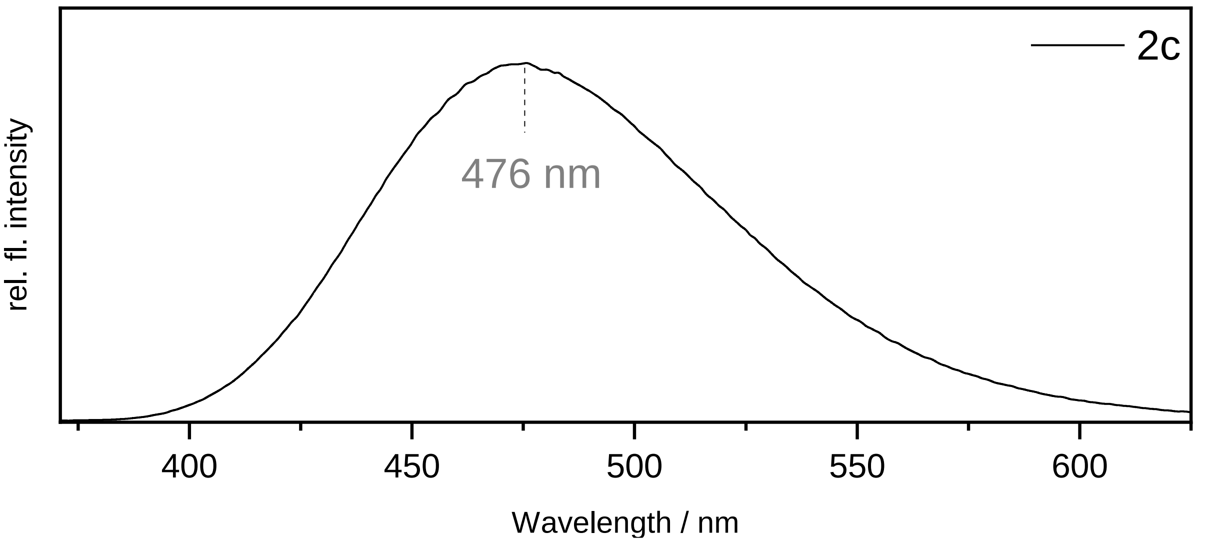

Supplementary Figure 83: Fluorescence spectrum of 2c.

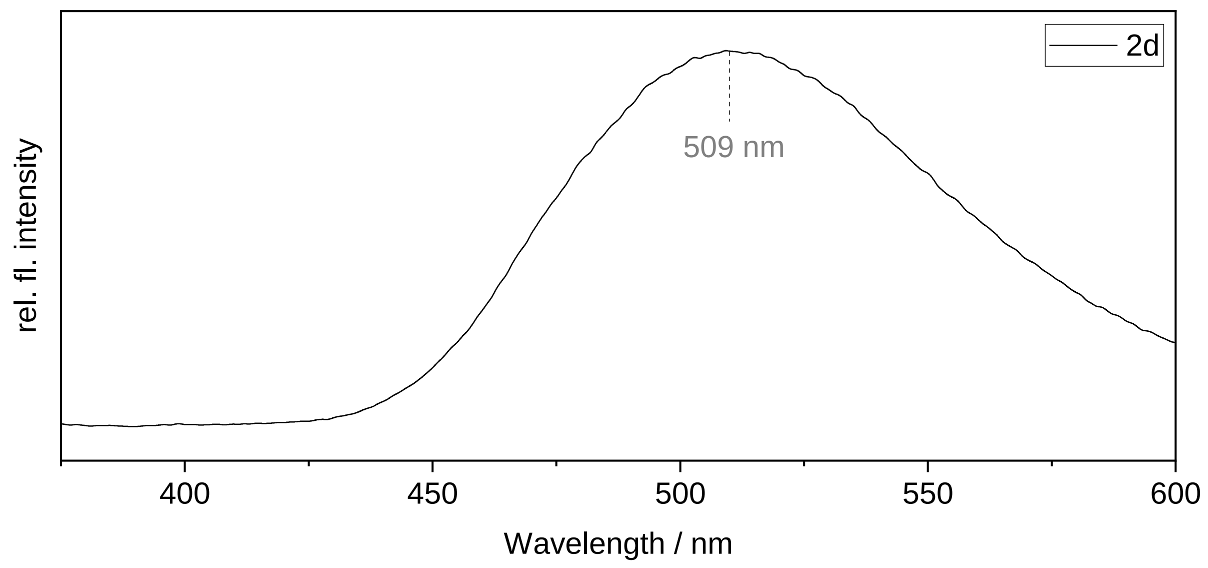

Supplementary Figure 84: Fluorescence spectrum of 2d.

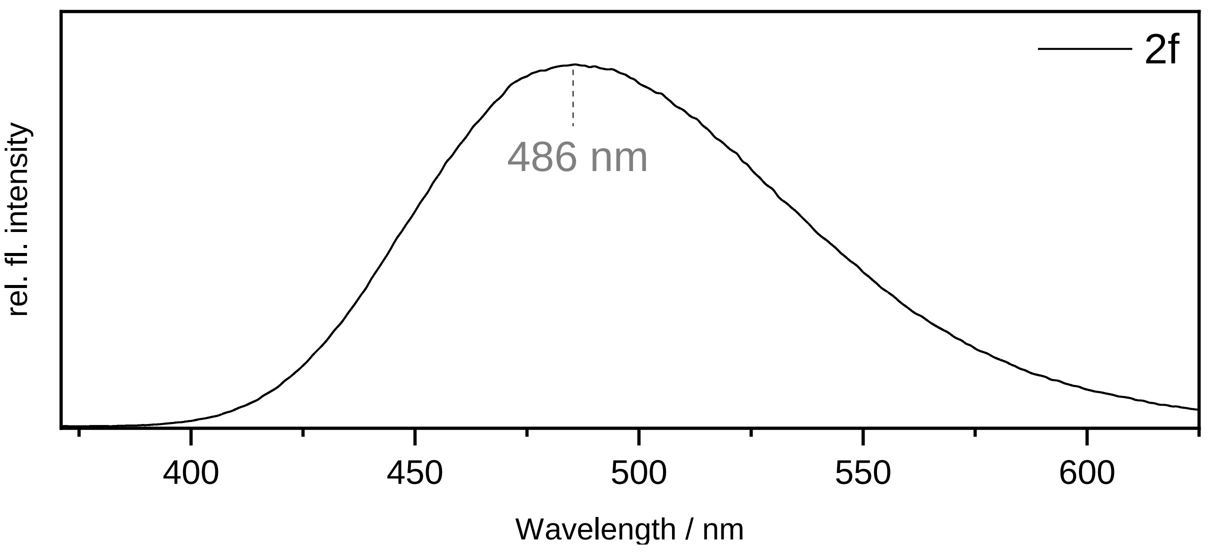

Supplementary Figure 85: Fluorescence spectrum of 2f.

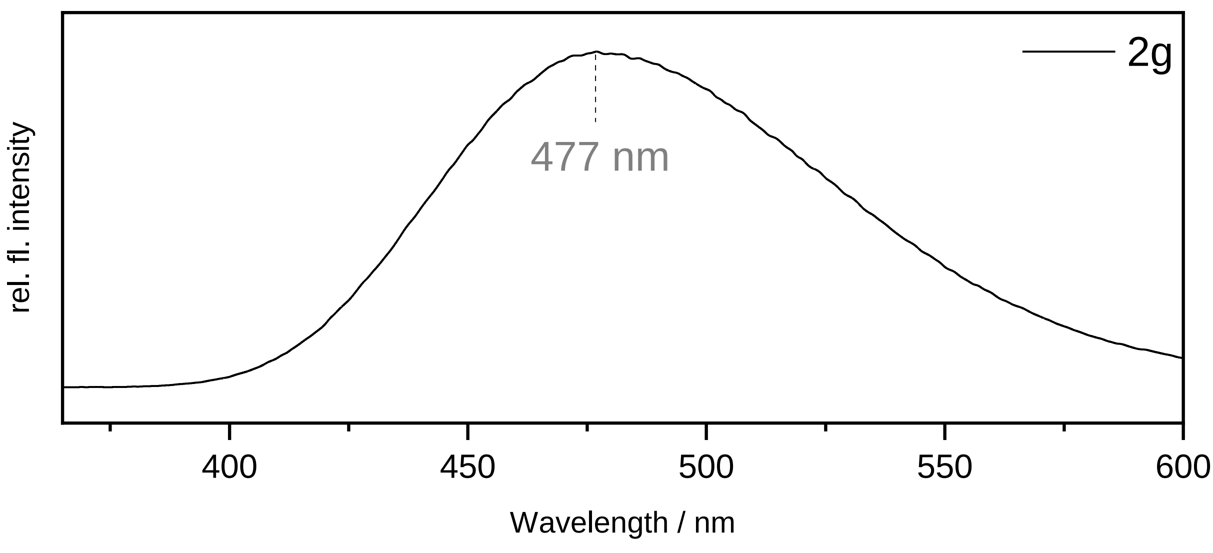

Supplementary Figure 86: Fluorescence spectrum of 2g.

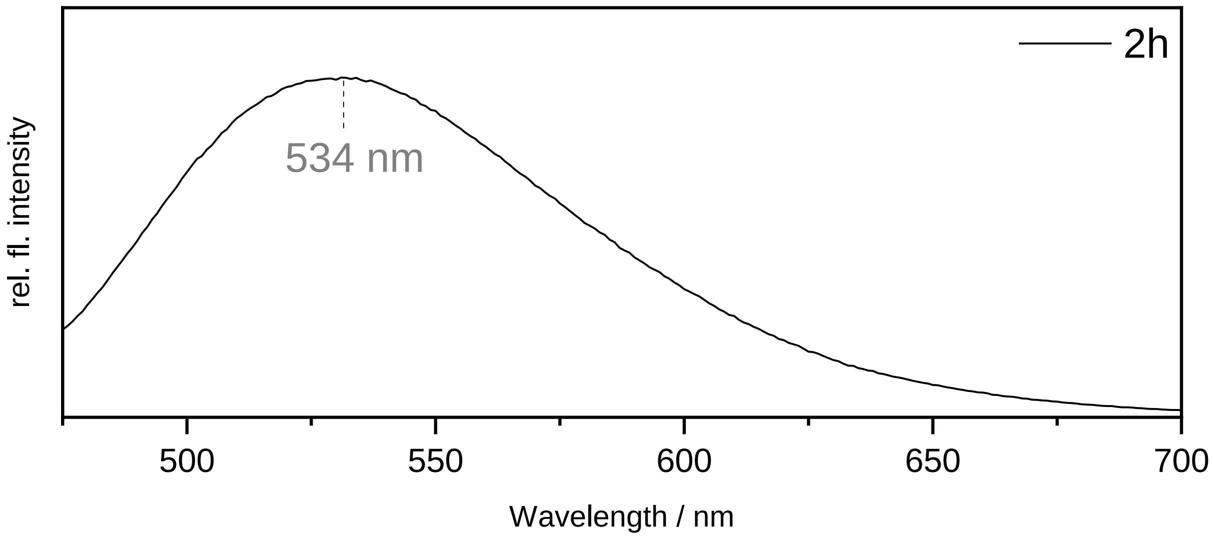

Supplementary Figure 87: Fluorescence spectrum of 2h.

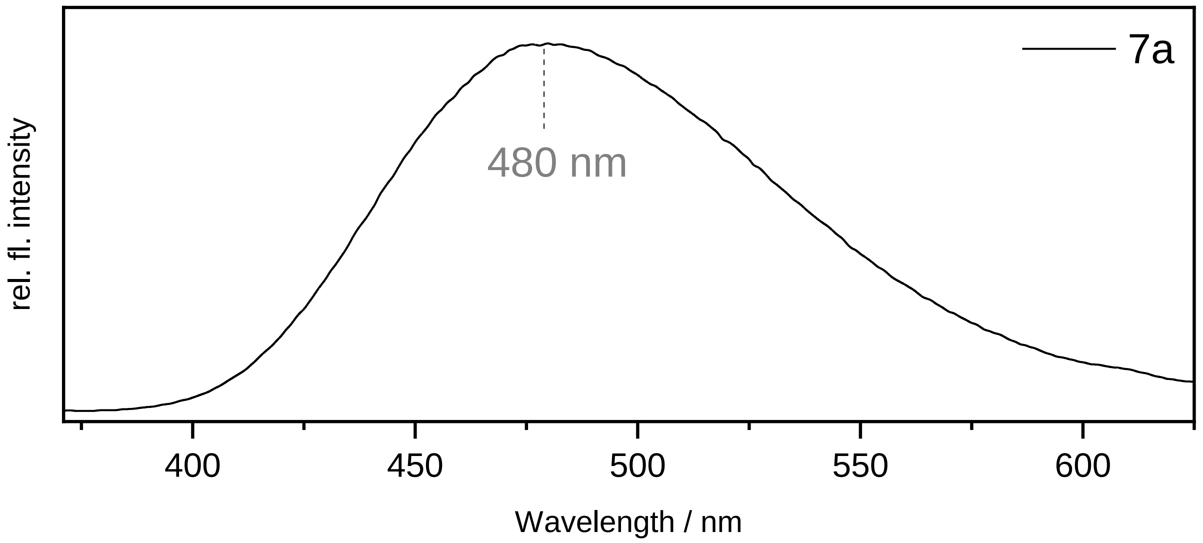

Supplementary Figure 88: Fluorescence spectrum of 7a.

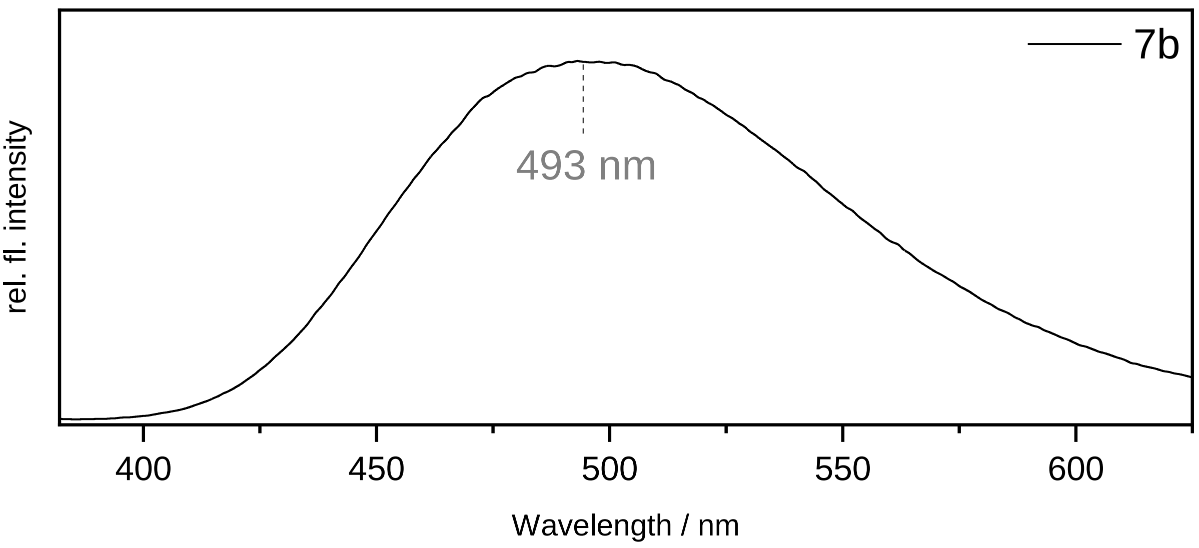

Supplementary Figure 89: Fluorescence spectrum of 7b.

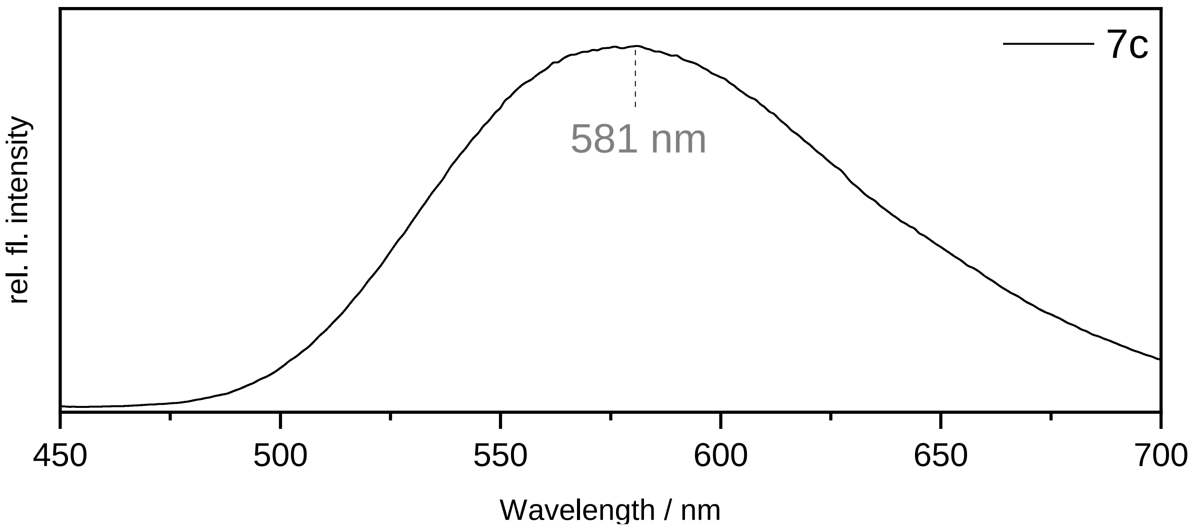

Supplementary Figure 90: Fluorescence spectrum of 7c.

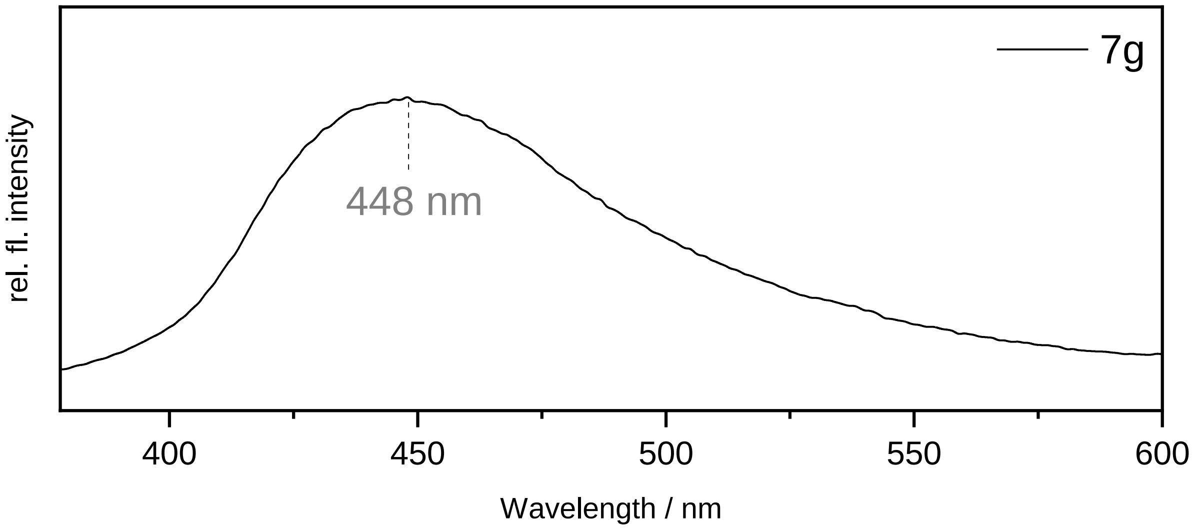

Supplementary Figure 91: Fluorescence spectrum of 7g.

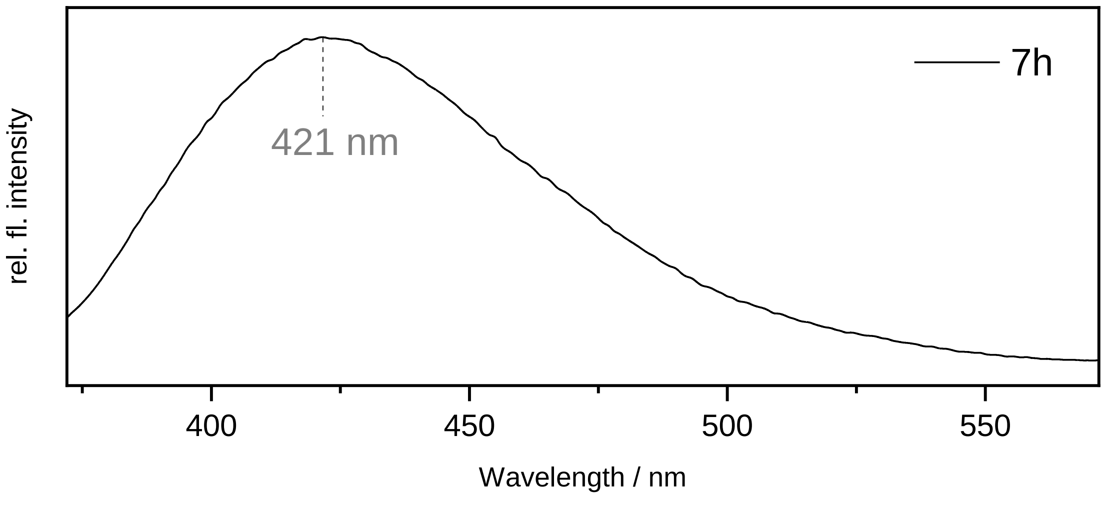

Supplementary Figure 92: Fluorescence spectrum of 7h.

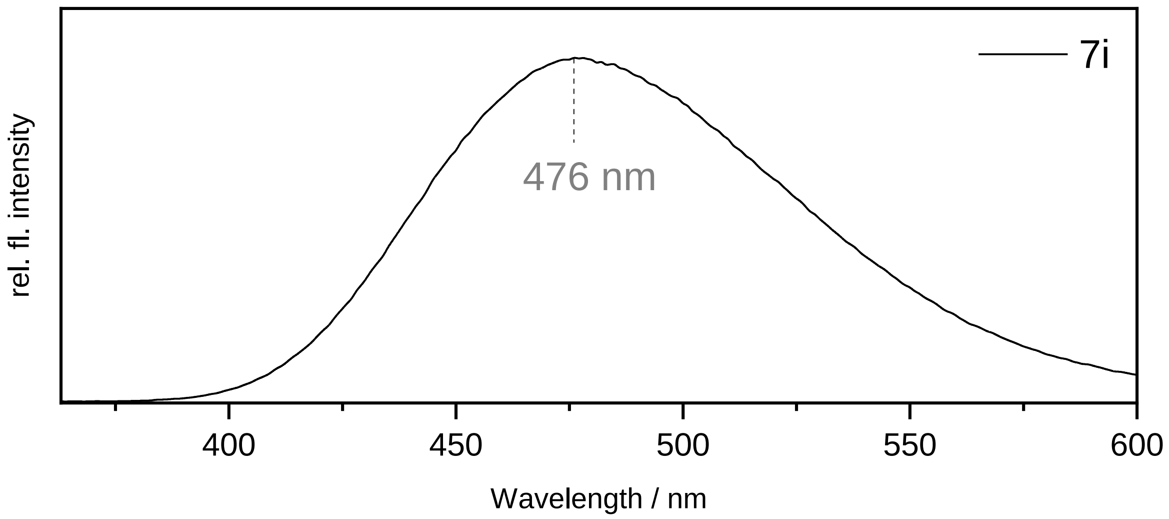

Supplementary Figure 93: Fluorescence spectrum of 7i.

1.5 Mechanistic Study

The mechanistic study was conducted by synthesizing intermediates postulated to be involved in the mechanism of the alkylidene-homocoupling reaction and subjecting them to control experiments with modified reaction conditions. The conditions used for the control experiments were as follows:

Conditions **A**: 500 µL DMSO-d_6, 100 °C, 1.20 eq. K_2CO_3, 0.25 eq. S_8,
Conditions **B**: 500 µL DMSO-d_6, 100 °C, 1.20 eq. K_2CO_3,
Conditions **C**: 500 µL DMSO-d_6, 100 °C, 0.25 eq. S_8,
Conditions **D**: 500 µL DMSO-d_6, 100 °C.
Conditions **E**: 500 µL DMSO-d_6, 100 °C, 1.20 eq. K_2CO_3, 0.25 eq. S_8 (Sulfur was added after a reaction time of 30 minutes).
Conditions **F**: 500 µL DMSO-d_6, 100 °C, 2.00 eq sodium *p*-toluenesulfinate (NaTs).

The substrates for these test reactions were NTH **1a**, diazo compound **4a**, thioketone **5a** and thiirane **6a**. In total, 5 series of test reactions were performed by subjecting one or more of these substrates to control experiment conditions:

Series 1: NTH **1a** under conditions **A-D** (Supplementary Figure 94).
Series 2: Diazo compound **4a** under conditions **A-D** (Supplementary Figure 95).
Series 3: Thioketone **5a** under conditions **A-D** (Supplementary Figure 96).
Series 4: NTH **1a** and thioketone **5a** (equal stoichiometry) under conditions **B** and **E** (Supplementary Figure 97).
Series 5: Thiirane **6a** under conditions **A-D** and **F** (Supplementary Figures 98 and 99).

All reactions were carried out in 10 mL glass vials on a 0.25 mmol scale. Heating of the reactions was performed inside an aluminium block with indentations for 10 mL glass vials. After 30 minutes (also 1 hour in Series 4), an NMR sample was taken by removing 50 µL of the crude mixture with a pipette and adding it to 400 µL of DMSO-d_6.

In the following, the ^{1}H-NMR spectra obtained from each series of control experiments are shown. The reaction scheme above shows the used substrates on the left side of the arrow and identified products of any test reaction on the right side of the arrow. In the spectra, the signals of known compounds are marked with color-coded boxes (dark blue for **1a**, red for **2a**, orange for **3a**, pink for **4a**, light blue for **5a**, light green for **6a**, dark green for *p*-toluenethiosulfonate (TsS^-), purple for *p*-toluenesulfinate (Ts^-)).

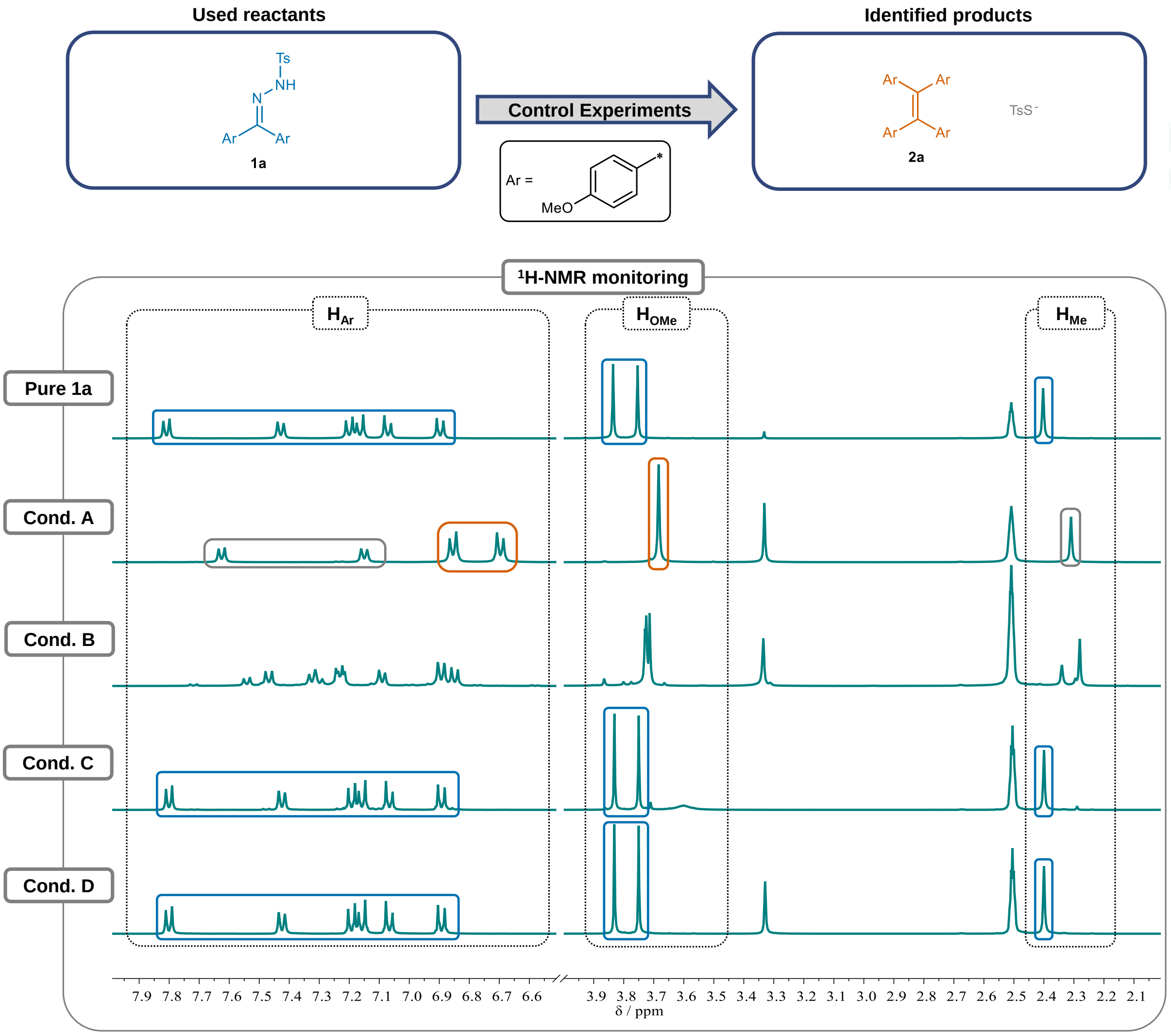

Supplementary Figure 94: ^{1}H-NMR spectra obtained from Series 1 of control experiments.

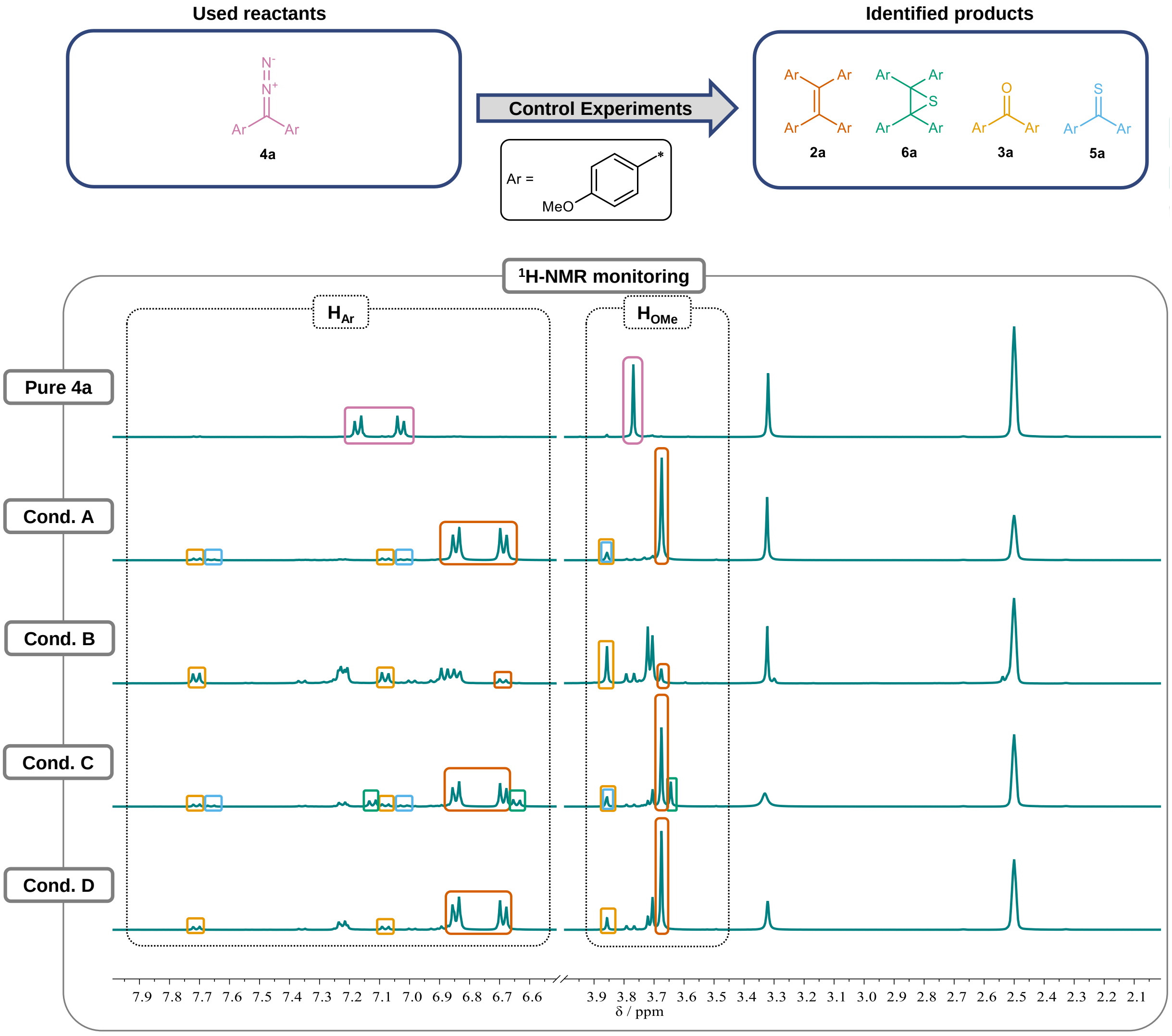

Supplementary Figure 95: ^{1}H-NMR spectra obtained from Series 2 of control experiments.

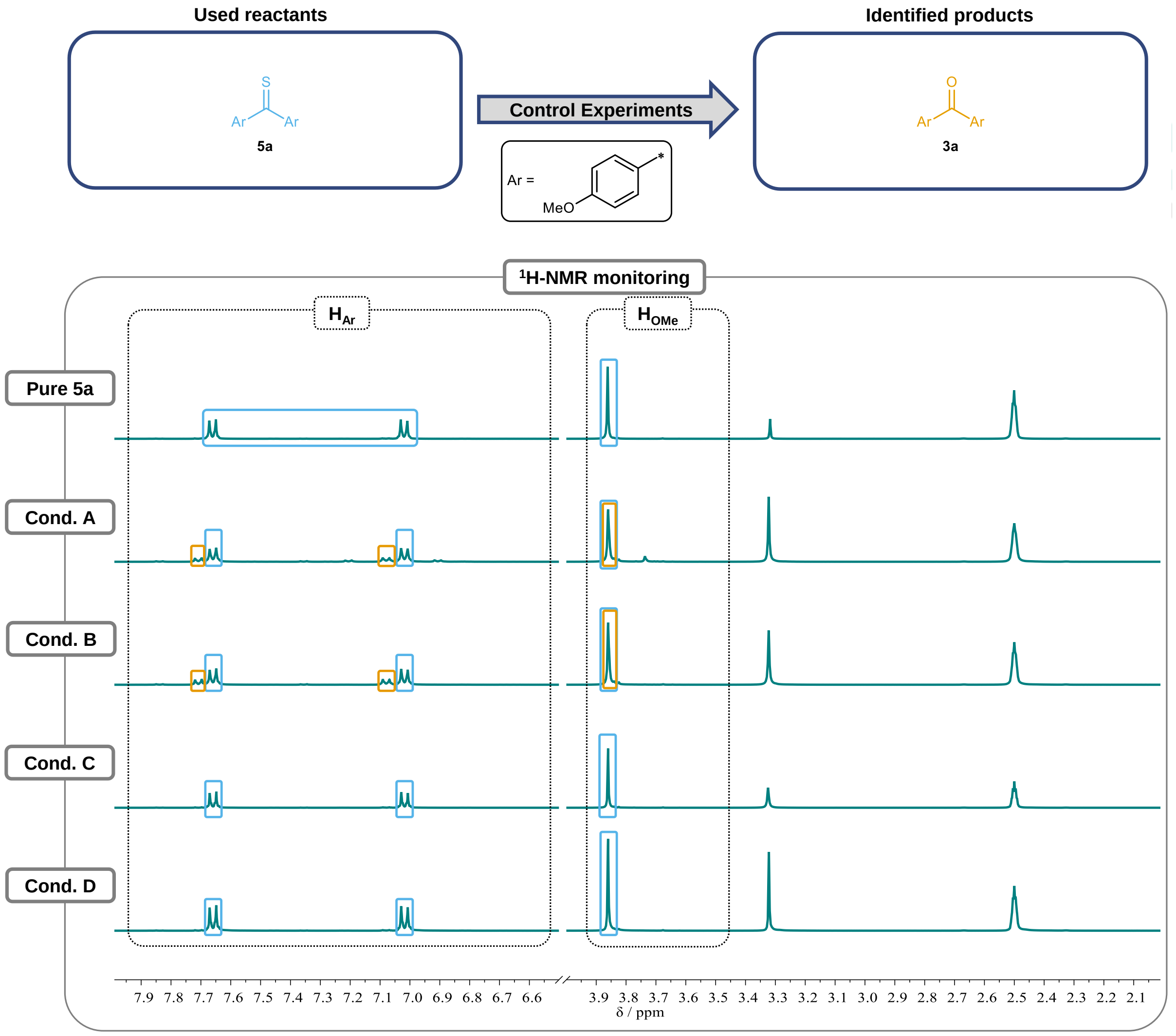

Supplementary Figure 96: ^{1}H-NMR spectra obtained from Series 3 of control experiments.

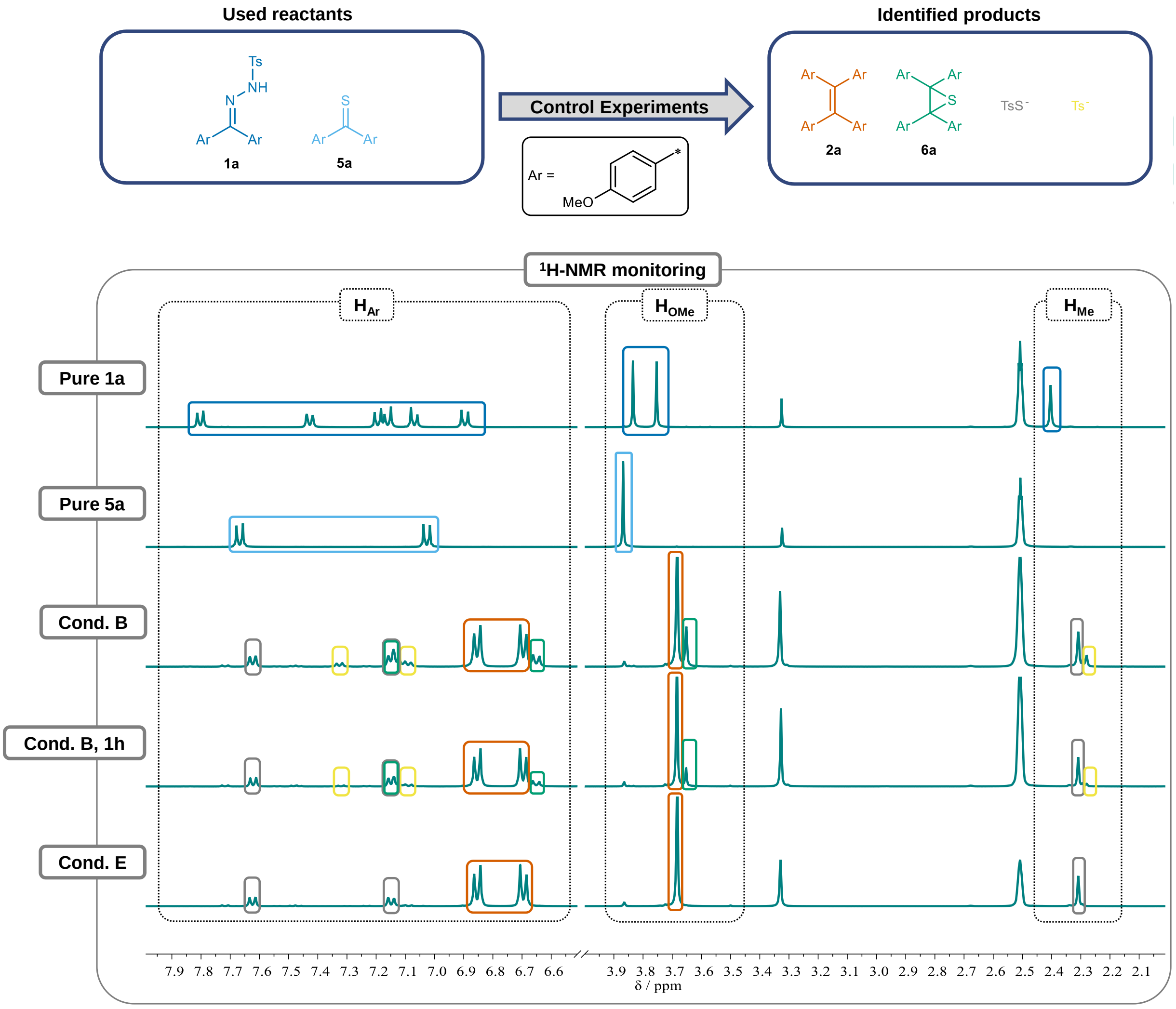

Supplementary Figure 97: ¹H-NMR spectra obtained from Series 4 of control experiments.

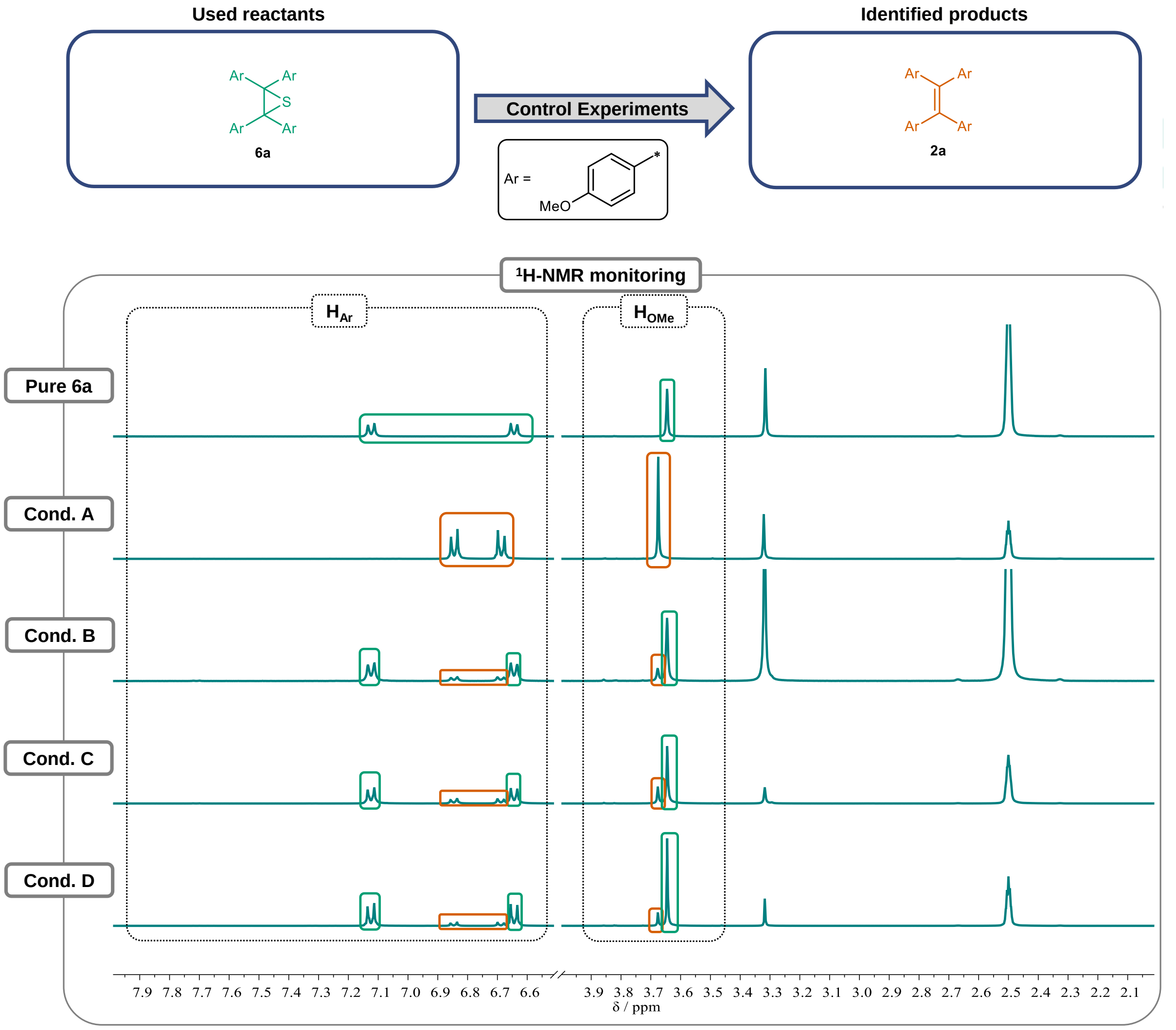

Supplementary Figure 98: ^{1}H-NMR spectra obtained from Series 5 of control experiments (conditions A-D).

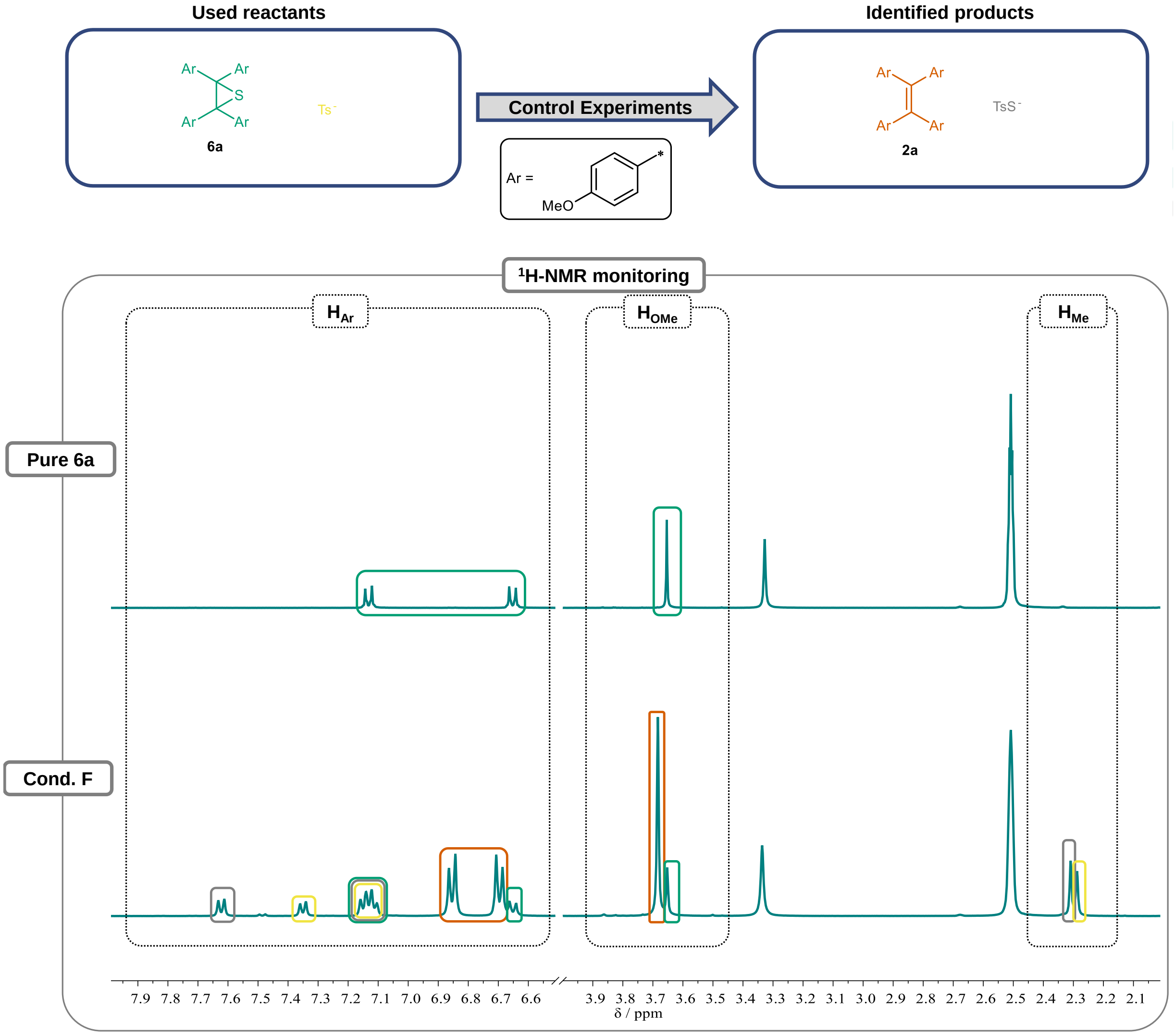

Supplementary Figure 99: ^{1}H-NMR spectra obtained from Series 5 of control experiments (conditions F).

2. Supplementary References

1. Erko, F. G., Cseh, L., Berthet, J., Mehl, G. H. & Delbaere, S. Synthesis and photochromic properties of a bis(diarylethene)-naphthopyran hybrid. *Dyes Pigm.* **115,** 102–109; 10.1016/j.dyepig.2014.12.015 (2015).
2. Tan, L.-S. & Yu, Z. *Bis(aniline) compounds containing multiple substituents with carbon-carbon triple-bonded groups*. Available at https://ppubs.uspto.gov/pubwebapp/external.html?q=(10988437).pn.&db=USPAT, US-PGPUB (2021).
3. Gannon, S. M. & Krause, J. G. Phase-Transfer Permanganate Oxidation of Unfunctionalized Benzylic Positions. *Synthesis* **1987,** 915–917; 10.1055/s-1987-28121 (1987).
4. Tomasulo, M., Kaanumal, S. L., Sortino, S. & Raymo, F. M. Synthesis and properties of benzophenone-spiropyran and naphthalene-spiropyran conjugates. *J. Org. Chem.* **72,** 595–605; 10.1021/jo062004d (2007).
