# Supplementary material for: Synthesis of highly substituted alkenes by sulfur-mediated olefination of N-tosylhydrazones
Source: Commun Chem. 2023 Nov 18;6:255. doi: 10.1038/s42004-023-01058-2 (PMC10657425; doi:10.1038/s42004-023-01058-2)
Supplement: Supplementary file 1 — Peer Review File [file 42004_2023_1058_MOESM1_ESM.pdf]

nature portfolio

Peer Review File

Synthesis of highly substituted alkenes by sulfur-mediated olefination of N-tosylhydrazones

Open Access This file is licensed under a Creative Commons Attribution 4.0 International License, which permits use, sharing, adaptation, distribution and reproduction in any medium or format, as long as you give appropriate credit to the original author(s) and the source, provide a link to the Creative Commons license, and indicate if changes were made. In the cases where the authors are anonymous, such as is the case for the reports of anonymous peer reviewers, author attribution should be to 'Anonymous Referee' followed by a clear attribution to the source work. The images or other third party material in this file are included in the article's Creative Commons license, unless indicated otherwise in a credit line to the material. If material is not included in the article's Creative Commons license and your intended use is not permitted by statutory regulation or exceeds the permitted use, you will need to obtain permission directly from the copyright holder. To view a copy of this license, visit http://creativecommons.org/licenses/by/4.0/.

This manuscript has been previously reviewed at another Nature Portfolio journal. This document only contains reviewer comments and rebuttal letters for versions considered at Communications Chemistry.

REVIEWERS' COMMENTS:

Reviewer #1 (Original Reviewer #2, Remarks to the Author):

My concerns have been well addressed by the authors, the topic of submitted research meet the readership of Communications Chemistry. In addition, after reviewing of the revised manuscript and the replies to other reviewers, this reviewer agree that the manuscript is carefully written, all important aspects are convincingly discussed in the manuscript. Overall, the manuscript is recommended to publish in Communications Chemistry in current form.
